# Conservation of copy number profiles during engraftment and passaging of patient-derived cancer xenografts

**DOI:** 10.1038/s41588-020-00750-6

**Published:** 2021-01-07

**Authors:** Xing Yi Woo, Jessica Giordano, Anuj Srivastava, Zi-Ming Zhao, Michael W. Lloyd, Roebi de Bruijn, Yun-Suhk Suh, Rajesh Patidar, Li Chen, Sandra Scherer, Matthew H. Bailey, Chieh-Hsiang Yang, Emilio Cortes-Sanchez, Yuanxin Xi, Jing Wang, Jayamanna Wickramasinghe, Andrew V. Kossenkov, Vito W. Rebecca, Hua Sun, R. Jay Mashl, Sherri R. Davies, Ryan Jeon, Christian Frech, Jelena Randjelovic, Jacqueline Rosains, Francesco Galimi, Andrea Bertotti, Adam Lafferty, Alice C. O’Farrell, Elodie Modave, Diether Lambrechts, Petra ter Brugge, Violeta Serra, Elisabetta Marangoni, Rania El Botty, Hyunsoo Kim, Jong-Il Kim, Han-Kwang Yang, Charles Lee, Dennis A. Dean, Brandi Davis-Dusenbery, Yvonne A. Evrard, James H. Doroshow, Alana L. Welm, Bryan E. Welm, Michael T. Lewis, Bingliang Fang, Jack A. Roth, Funda Meric-Bernstam, Meenhard Herlyn, Michael A. Davies, Li Ding, Shunqiang Li, Ramaswamy Govindan, Claudio Isella, Jeffrey A. Moscow, Livio Trusolino, Annette T. Byrne, Jos Jonkers, Carol J. Bult, Enzo Medico, Jeffrey H. Chuang, PDXNET Consortium, Matthew H. Bailey, Vito W. Rebecca, Michael A. Davies, Peter N. Robinson, Brian J. Sanderson, Steven B. Neuhauser, Lacey E. Dobrolecki, Xiaofeng Zheng, Mourad Majidi, Ran Zhang, Xiaoshan Zhang, Argun Akcakanat, Kurt W. Evans, Timothy A. Yap, Dali Li, Erkan Yucan, Christopher D. Lanier, Turcin Saridogan, Bryce P. Kirby, Min Jin Ha, Huiqin Chen, Scott Kopetz, David G. Menter, Jianhua Zhang, Shannon N. Westin, Michael P. Kim, Bingbing Dai, Don L. Gibbons, Coya Tapia, Vanessa B. Jensen, Gao Boning, John D. Minna, Hyunsil Park, Brenda C. Timmons, Luc Girard, Dylan Fingerman, Qin Liu, Rajasekharan Somasundaram, Min Xiao, Vashisht G. Yennu-Nanda, Michael T. Tetzlaff, Xiaowei Xu, Katherine L. Nathanson, Song Cao, Feng Chen, John F. DiPersio, Kian H. Lim, Cynthia X. Ma, Fernanda M. Rodriguez, Brian A. Van Tine, Andrea Wang-Gillam, Michael C. Wendl, Yige Wu, Matthew A. Wyczalkowski, Lijun Yao, Reyka Jayasinghe, Rebecca L. Aft, Ryan C. Fields, Jingqin Luo, Katherine C. Fuh, Vicki Chin, John DiGiovanna, Jeffrey Grover, Soner Koc, Sara Seepo, Tiffany Wallace, Chong-Xian Pan, Moon S. Chen, Luis G. Carvajal-Carmona, Amanda R. Kirane, May Cho, David R. Gandara, Jonathan W. Riess, Tiffany Le, Ralph W. deVere White, Clifford G. Tepper, Hongyong Zhang, Nicole B. Coggins, Paul Lott, Ana Estrada, Ted Toal, Alexa Morales Arana, Guadalupe Polanco-Echeverry, Sienna Rocha, Ai-Hong Ma, Nicholas Mitsiades, Salma Kaochar, Bert W. O’Malley, Matthew J. Ellis, Susan G. Hilsenbeck, Michael Ittmann, EurOPDX Consortium, Roebi de Bruijn, Petra ter Brugge, Simona Corso, Alessandro Fiori, Silvia Giordano, Marieke van de Ven, Daniel S. Peeper, Ian Miller, Cristina Bernadó, Beatriz Morancho, Lorena Ramírez, Joaquín Arribas, Héctor G. Palmer, Alejandro Piris-Gimenez, Laura Soucek, Ahmed Dahmani, Elodie Montaudon, Fariba Nemati, Virginie Dangles-Marie, Didier Decaudin, Sergio Roman-Roman, Denis G. Alférez, Katherine Spence, Robert B. Clarke, Mohamed Bentires-Alj, David K. Chang, Andrew V. Biankin, Alejandra Bruna, Martin O’Reilly, Carlos Caldas, Oriol Casanovas, Eva Gonzalez-Suarez, Purificacíon Muñoz, Alberto Villanueva, Nathalie Conte, Jeremy Mason, Ross Thorne, Terrence F. Meehan, Helen Parkinson, Zdenka Dudova, Ales Křenek, Dalibor Stuchlík, Olivier Elemento, Giorgio Inghirami, Anna Golebiewska, Simone P. Niclou, G. Bea A. Wisman, Steven de Jong, Petra Kralova, Radislav Sedlacek, Elisa Claeys, Eleonora Leucci, Massimiliano Borsani, Luisa Lanfrancone, Pier Giuseppe Pelicci, Gunhild Mari Mælandsmo, Jens Henrik Norum, Emilie Vinolo

**Affiliations:** 1 The Jackson Laboratory for Genomic Medicine, Farmington, CT USA; 2 Department of Oncology, University of Turin, Turin, Italy; 3 Candiolo Cancer Institute, FPO-IRCCS, Turin, Italy; 4 The Jackson Laboratory for Mammalian Genetics, Bar Harbor, ME USA; 5 Netherlands Cancer Institute, Amsterdam, the Netherlands; 6 College of Medicine, Seoul National University, Seoul, Republic of Korea; 7 Frederick National Laboratory for Cancer Research, Frederick, MD USA; 8 Department of Oncological Sciences, Huntsman Cancer Institute, University of Utah, Salt Lake City, UT USA; 9 Department of Human Genetics, University of Utah, Salt Lake City, UT USA; 10 Department of Bioinformatics and Computational Biology, The University of Texas MD Anderson Cancer Center, Houston, TX USA; 11 The Wistar Institute, Philadelphia, PA USA; 12 Department of Medicine, Washington University School of Medicine in St. Louis, St. Louis, MO USA; 13 Seven Bridges Genomics, Charlestown, MA USA; 14 Department of Physiology and Medical Physics, Centre for Systems Medicine, Royal College of Surgeons in Ireland, Dublin, Ireland; 15 Center for Cancer Biology, VIB, Leuven, Belgium; 16 Laboratory of Translational Genetics, Department of Human Genetics, KU Leuven, Leuven, Belgium; 17 Vall d´Hebron Institute of Oncology, Barcelona, Spain; 18 Department of Translational Research, Institut Curie, PSL Research University, Paris, France; 19 Precision Medicine Center, The First Affiliated Hospital of Xi’an Jiaotong University, Xi’an, People’s Republic of China; 20 Department of Life Sciences, Ewha Womans University, Seoul, Republic of Korea; 21 Division of Cancer Treatment and Diagnosis, National Cancer Institute, Bethesda, MD USA; 22 Department of Surgery, Huntsman Cancer Institute, University of Utah, Salt Lake City, UT USA; 23 Lester and Sue Smith Breast Center, Baylor College of Medicine, Houston, TX USA; 24 Department of Thoracic and Cardiovascular Surgery, The University of Texas MD Anderson Cancer Center, Houston, TX USA; 25 Department of Investigational Cancer Therapeutics, The University of Texas MD Anderson Cancer Center, Houston, TX USA; 26 Department of Melanoma Medical Oncology, The University of Texas MD Anderson Cancer Center, Houston, TX USA; 27 Investigational Drug Branch, National Cancer Institute, Bethesda, MD USA; 28 Department of Biostatistics, The University of Texas MD Anderson Cancer Center, Houston, TX USA; 29 Department of Gastrointestinal Medical Oncology, The University of Texas MD Anderson Cancer Center, Houston, TX USA; 30 Department of Genomic Medicine, The University of Texas MD Anderson Cancer Center, Houston, TX USA; 31 Department of Gynecologic Oncology and Reproductive Medicine, The University of Texas MD Anderson Cancer Center, Houston, TX USA; 32 Department of Surgical Oncology, The University of Texas MD Anderson Cancer Center, Houston, TX USA; 33 Department of Thoracic/Head and Neck Medical Oncology, The University of Texas MD Anderson Cancer Center, Houston, TX USA; 34 Department of Translational Molecular Pathology, The University of Texas MD Anderson Cancer Center, Houston, TX USA; 35 Department of Veterinary Medicine and Surgery, The University of Texas MD Anderson Cancer Center, Houston, TX USA; 36 Hamon Center For Therapeutic Oncology, UT Southwestern Medical Center, Dallas, TX USA; 37 Department of Pathology and Laboratory Medicine, Hospital of the University of Pennsylvania, Philadelphia, PA USA; 38 Abramson Cancer Center, University of Pennsylvania, Philadelphia, PA USA; 39 Department of Surgery, Washington University School of Medicine in St. Louis, St. Louis, MO USA; 40 Division of Gynecologic Oncology, Washington University School of Medicine in St. Louis, St. Louis, MO USA; 41 Center to Reduce Cancer Health Disparities, National Cancer Institute, Bethesda, MD USA; 42 Department of Internal Medicine, Division of Hematology and Oncology, University of California, Davis, Sacramento, CA USA; 43 Department of Biochemistry and Molecular Medicine, University of California, Davis, Sacramento, CA USA; 44 UC Davis Comprehensive Cancer Center, University of California, Davis, Sacramento, CA USA; 45 UC Davis Genome Center, University of California, Davis, Sacramento, CA USA; 46 Department of Medicine, Baylor College of Medicine, Houston, TX USA; 47 Department of Molecular and Cellular Biology, Baylor College of Medicine, Houston, TX USA; 48 Department of Pathology, Baylor College of Medicine, Houston, TX USA; 49 Manchester Breast Centre, Division of Cancer Sciences, University of Manchester, Manchester, UK; 50 University Hospital of Basel, University of Basel, Basel, Switzerland; 51 Institute of Cancer Sciences, University of Glasgow, Glasgow, UK; 52 Cancer Research UK Cambridge Institute, Cambridge Cancer Centre, Cambridge, UK; 53 Catalan Institute of Oncology, L’Hospitalet de Llobregat, Barcelona, Spain; 54 European Bioinformatics Institute, European Molecular Biology Laboratory, Wellcome Genome Campus, Hinxton, UK; 55 Institute of Computer Science, Masaryk University, Brno, Czech Republic; 56 Weill Cornell Medical College, Cornell University, New York, NY USA; 57 NorLux Neuro-Oncology Laboratory, Department of Oncology, Luxembourg Institute of Health, Luxembourg, Luxembourg; 58 University Medical Centre Groningen, Groningen, the Netherlands; 59 Czech Center for Phenogenomics, Institute of Molecular Genetics, Prague, Czech Republic; 60 TRACE PDX Platform, Katholieke Universiteit Leuven, Leuven, Belgium; 61 European Institute of Oncology, Milan, Italy; 62 Oslo University Hospital, Oslo, Norway; 63 Seeding Science SPRL, Limelette, Belgium

**Keywords:** Cancer, Computational biology and bioinformatics

## Abstract

Patient-derived xenografts (PDXs) are resected human tumors engrafted into mice for preclinical studies and therapeutic testing. It has been proposed that the mouse host affects tumor evolution during PDX engraftment and propagation, affecting the accuracy of PDX modeling of human cancer. Here, we exhaustively analyze copy number alterations (CNAs) in 1,451 PDX and matched patient tumor (PT) samples from 509 PDX models. CNA inferences based on DNA sequencing and microarray data displayed substantially higher resolution and dynamic range than gene expression-based inferences, and they also showed strong CNA conservation from PTs through late-passage PDXs. CNA recurrence analysis of 130 colorectal and breast PT/PDX-early/PDX-late trios confirmed high-resolution CNA retention. We observed no significant enrichment of cancer-related genes in PDX-specific CNAs across models. Moreover, CNA differences between patient and PDX tumors were comparable to variations in multiregion samples within patients. Our study demonstrates the lack of systematic copy number evolution driven by the PDX mouse host.

## Abstract (web summary)

Analysis of copy number alterations in 1,451 patient-derived xenografts (PDXs) and matched patient tumor samples shows strong conservation from patient tumors through late-passage PDXs and a lack of systematic copy number evolution driven by the mouse host.

## Main

Human tumors engrafted into transplant-compliant recipient mice (patient-derived xenografts (PDXs)) have advantages over previous model systems of human cancer (for example, genetically engineered mouse models^1,2^ and cancer cell lines^3^) for preclinical drug efficacy studies because they allow researchers to directly study human cells and tissues in vivo^4–7^. Comparisons of genome characteristics and histopathology of primary tumors and xenografts of various cancer types^8–14^ have demonstrated that the biological properties of patient-derived tumors are largely preserved in xenografts. A growing body of literature supports their use in cancer drug discovery and development^15–17^.

A caveat to PDX models is that intratumoral evolution can occur during engraftment and passaging^18–22^. Such evolution could potentially modify treatment response of PDXs with respect to the patient tumors (PTs)^19,23,24^, particularly if the evolution were to systematically alter cancer-related genes. Recently, Ben-David et al.^23^ reported extensive PDX copy number divergence from the PT of origin and across passages, based mainly on large-scale assessment of copy number alteration (CNA) profiles inferred from gene expression microarray data. They raised concerns about genetic evolution in PDXs as a consequence of mouse-specific selective pressures, which could impact the capacity of PDXs to faithfully model patient treatment response. Such results contrast with reports of observations of genomic fidelity of PDX models with respect to the originating PTs and from early to late passages by direct DNA measurements in several dozen PDX models^8,11,25^.

Here, we resolve these contradicting observations by systematically evaluating CNA changes and the genes they affect during engraftment and passaging in a large, internationally collected set of PDX models, comparing both RNA- and DNA-based approaches. The data collected, as part of the US National Cancer Institute (NCI) PDX Development and Trial Centers Research Network (PDXNet) Consortium and EurOPDX Consortium, comprises PT and PDX samples from >500 models. Our study demonstrates that previous reports of systematic copy number divergence between PTs and PDXs are incorrect, and that there is high retention of copy number during PDX engraftment and passaging. This work also finely enumerates the copy number profiles in hundreds of publicly available models, which will enable researchers to assess the suitability of each for individualized treatment studies.

## Results

### Catalog of CNAs in PDXs

We have assembled CNA profiles of 1,451 unique samples (324 PT samples and 1,127 PDX samples), corresponding to 509 PDX models contributed by participating centers of the PDXNET, the EurOPDX Consortium and other published datasets^11,26^ (see Methods, Supplementary Methods, Supplementary Table 1 and Supplementary Fig. 1). We estimated the copy number from five data types (single nucleotide polymorphism (SNP) array, whole-exome sequencing (WES), low-pass whole-genome sequencing (WGS), RNA sequencing (RNA-seq) and gene expression array data), yielding 1,548 tumor datasets including samples assayed on multiple platforms (see Methods, Supplementary Methods and Supplementary Data 1). Paired normal DNA, and in some cases paired normal RNA, were also obtained to calibrate WES and RNA-seq tumor samples.

**Fig. 1:**
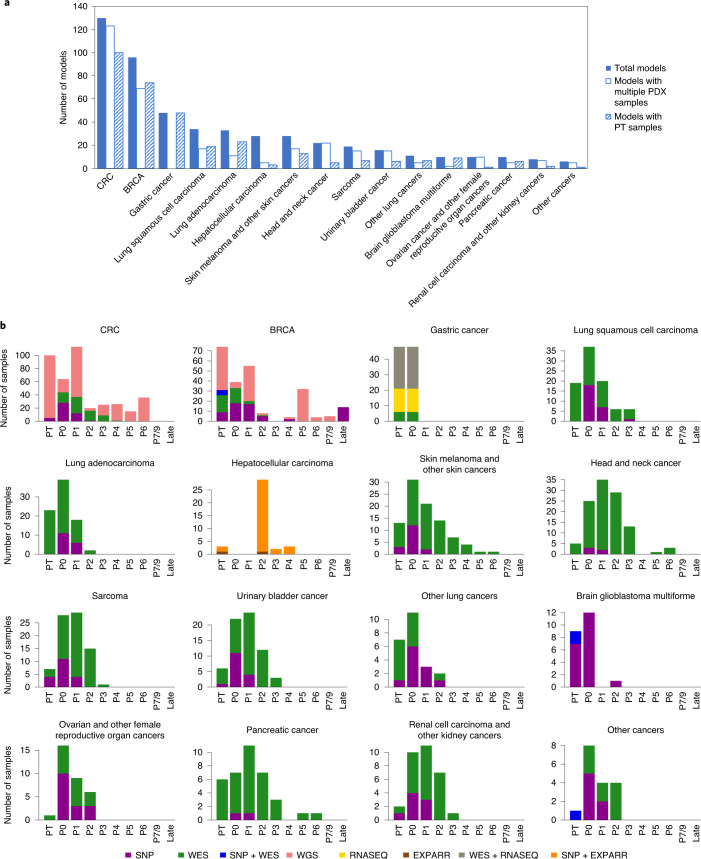
PDX datasets used for copy number profiling across 16 tumor types. **a**, Numbers of PDX models for each tumor type, with models also having multiple PDX samples or having matched PT samples specified. **b**, Distributions of datasets by passage number and assay platform for PTs and PDX samples, separated by tumor type. Late passages include P18, P19 and P21 samples.

The combined PDX data represent 16 broad tumor types derived from American, European and Asian patients with cancer (see Methods), with 64% (*n* = 324) of the models having their corresponding PTs assayed and another 64% (*n* = 328) having multiple PDX samples of either varying passages (P0–P21) or varying lineages from propagation into distinct mice (Fig. 1a and Supplementary Table 2). The distributions of PT and PDX samples across different tumor types, passages and assay platforms (Fig. 1b and Supplementary Figs. 2–12) show the wide spectrum of this combined dataset, which, to the best of our knowledge, is the most comprehensive copy number profiling of PDXs compiled to date (Supplementary Note 1). Additionally, our data include seven patients with multiple tumors collected either from different relapse time points or different metastatic sites, resulting in multiple PDX models derived from a single patient.

### Comparison of CNA profiles from SNP array, WES and gene expression data

To compare the CNA profiles from different platforms in a controlled fashion, we assembled a dataset with matched measurements across multiple platforms (Supplementary Table 3 and Supplementary Figs. 13–17). Copy number calling has been reported to be noisy for several data types^27,28^, and we observed that quantitative comparisons between CNA profiles are sensitive to: (1) the thresholds and baselines used to define gains and losses; (2) the dynamic range of copy number values from each platform; and (3) the differential impacts of normal cell contamination for different measurements. To control for such systematic biases, we assessed the similarity between two CNA profiles using the Pearson correlation of their log_2_[copy number ratio] values across the genome in 100-kilobase (kb) windows. Regions with discrepant copy number were identified as those with outlier values from the linear regression model (see Methods).

#### CNAs from WES are consistent with CNAs from SNP array data

**Fig. 2:**
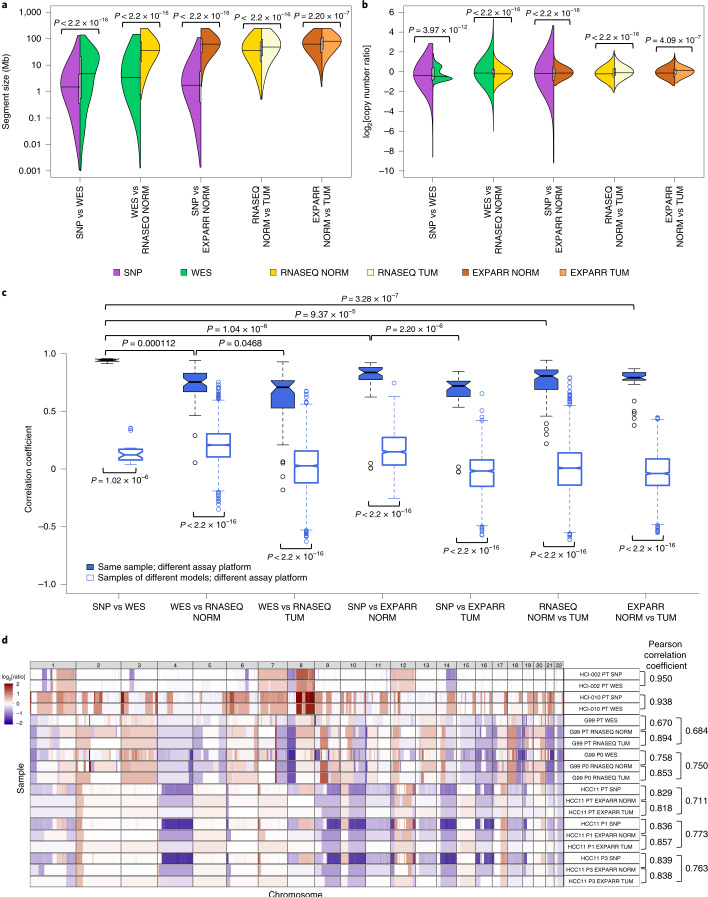
Comparisons of resolution and accuracy for CNAs estimated using DNA- and expression-based methods. **a**, Pairwise comparisons of the distributions of CNA segment sizes as estimated using different measurement platforms in the validation dataset. CNAs are regions with (|log_2_[copy number ratio]| ≥ 0.1). *P* values indicate the significance of the difference between distributions by two-sided Wilcoxon rank-sum test. vs, versus. **b**, Pairwise comparisons of the distributions of CNA segment log_2_[copy number ratio] values. *P* values were computed by two-sided Kolmogorov–Smirnov test. **c**, Distributions of Pearson correlation coefficients of median-centered log_2_[copy number ratio] values in 100-kb windows from CNA segments between pairs of samples estimated using different platforms. Samples with non-aberrant profiles in SNP array and WES data were omitted (5–95% inter-percentile range of log_2_[copy number ratio] < 0.3). *P* values were computed by two-sided Wilcoxon rank-sum test. In the box plots, the center line represents the median, the box limits are the upper and lower quantiles, the whiskers extend to 1.5× the interquartile range and the dots represent outliers. **d**, Examples of CNA profiles in comparisons of different platforms. Pearson correlation coefficients of CNA segments between pairs of samples are shown on the right. See Supplementary Table 3 for the number of samples per group. Examples of CNA profiles in comparisons of different platforms are shown; each sample ID is denoted by the model ID, passage number and platform used (see Supplementary Data 1).

As earlier studies reported that CNA estimates from WES data have more uncertainties than those from SNP arrays^29,30^, we implemented a WES-based CNA pipeline and validated it against SNP array-based estimates^31,32^ for matched samples. Copy number gain/loss segments (see Methods) from SNP arrays were of a higher resolution (Fig. 2a; median and mean segment sizes = 1.49 and 4.05 megabases (Mb) for SNP and 4.70 and 14.6 Mb for WES, respectively; *P* < 2.2 × 10^−^^16^) and wider dynamic range (Fig. 2b; range of log_2_[copy number ratio] = –8.62–2.84 for SNP and –3.04–1.85 for WES; *P* < 2.2 × 10^−^^16^). The difference in range is apparent in the linear regressions between platforms (Supplementary Fig. 18). These observations take into account the broad factors affecting CNA estimates across platforms, such as the positional distribution of sequencing loci, the sequencing depth of WES and the superior removal of normal cell contamination by SNP array CNA analysis workflows using SNP allele frequencies^33^.

**Extended Data Fig. 1:**
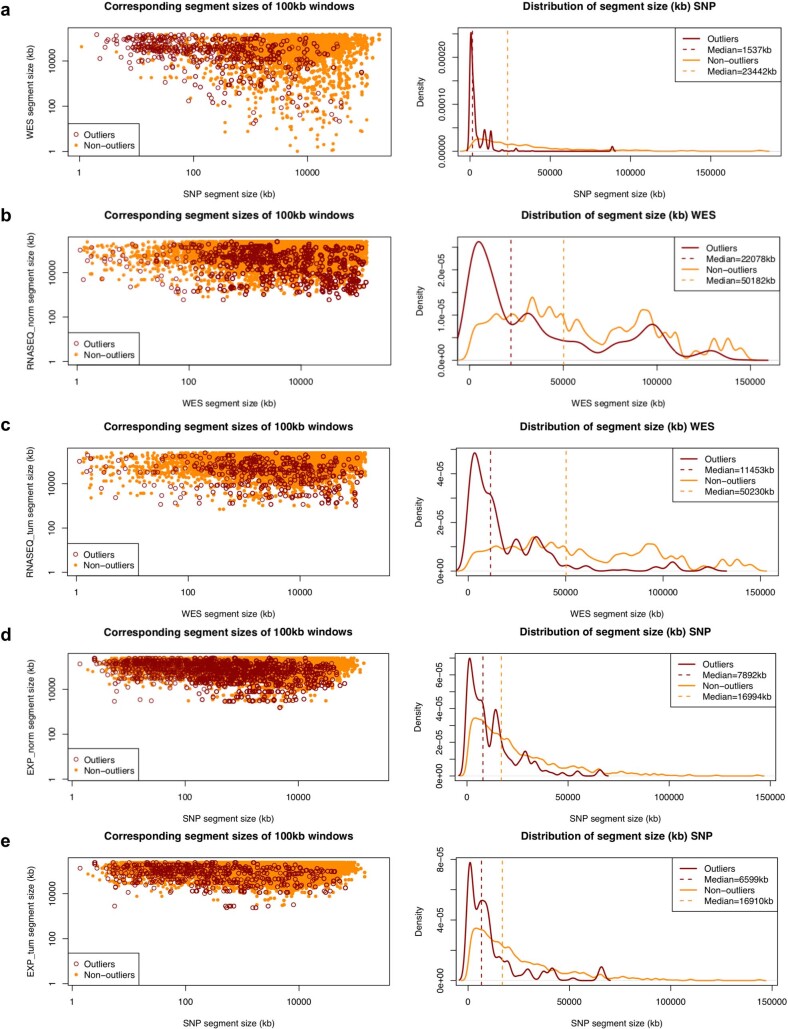
Comparison of segment sizes between different platforms. The left panel compares the combined corresponding segment sizes of outlier and non-outliers from the linear regression of the log_2_(CN ratio) of 100-kb windows binned from copy number segments between matched samples estimated from two different platforms or methods combined. Outliers of the linear regression are identified by studentized residuals > 3 and < -3. **a**, SNP vs. WES. **b**, WES vs. RNASEQ (NORM). **c**, WES vs. RNASEQ (TUM). **d**, SNP vs. EXPARR (NORM). **e**, SNP vs. EXPARR (TUM) (see Supplementary Table 3). The right panel compares the distribution of the segment sizes of outliers and non-outliers for the platform or method of higher resolution.

We observed strong agreement between SNP arrays and WES, with significantly higher Pearson correlation coefficients on matched samples than samples of different models (range = 0.913–0.957 for matched samples and 0.0366–0.354 for unmatched samples; *P* = 1.02 × 10^−^^6^), with the exception of two samples that lacked CNA aberrations and were removed (Fig. 2c and Supplementary Figs. 13, 18 and 19). The discordant copy number regions largely correspond to small focal events (average size = 1.53 Mb) detectable by SNP arrays but missed by WES (Supplementary Fig. 18 and Extended Data Fig. 1a; see Methods). Hence, CNA profiling by WES is reliable in most regions in this small dataset, with 99% of the genome locations across the samples consistent with the values from SNP arrays (Supplementary Note 2). These PT-based observations are also applicable to PDXs given that mouse DNA is absent in SNP array signal and removed from WES reads^34–36^.

#### Low accuracy for gene expression-derived CNA profiles

To compare the suitability of gene expression for quantifying evolutionary changes in CNA, we adapted the e-karyotyping method^23,37,38^ for RNA-seq and gene expression array data (Supplementary Figs. 15 and 17; see Methods). Copy number segments calibrated by non-tumor expression were of higher resolution (Fig. 2a; median and mean segment sizes = 36.0 and 51.9 Mb for RNASEQ NORM versus 48.2 and 65.3 Mb for RNASEQ TUM (*P* < 2.2 × 10^−^^16^) and 62.0 and 72.4 Mb for EXPARR NORM versus 80.1 and 85.2 Mb for EXPARR TUM (*P* = 2.20 × 10^−^^7^), where RNASEQ and EXPARR relate to RNA-seq and gene expression array, respectively, and NORM and TUM relate to normalization by median expression of normal and tumor samples, respectively) and a wider dynamic range (Fig. 2b; range of log_2_[copy number ratio] = –2.07–2.17 for RNASEQ NORM versus –1.79–1.81 for RNASEQ TUM (*P* < 2.2 × 10^−^^16^) and –1.40–1.89 for EXPARR NORM versus –1.13–1.59 for EXPARR TUM (*P* = 4.09 × 10^−^^7^)) compared with segments calculated by calibration with tumor samples. These alternative expression calibrations yielded biased gain and loss frequencies (Supplementary Note 3 and Supplementary Fig. 20) and strong variability (Pearson correlation range = 0.218–0.943 for RNASEQ NORM versus TUM and 0.377–0.869 for EXPARR NORM versus TUM) in the CNA calls (Fig. 2c and Supplementary Fig. 21). This range of correlations was far greater than was observed in comparisons between the DNA-based methods (*P* = 9.37 × 10^−^^5^ and *P* = 3.28 × 10^−^^7^ relative to SNP versus WES). This indicates the problematic nature of RNA-based CNA calling with calibration by tumor samples, which has been used when normal samples are not available.

**Extended Data Fig. 2:**
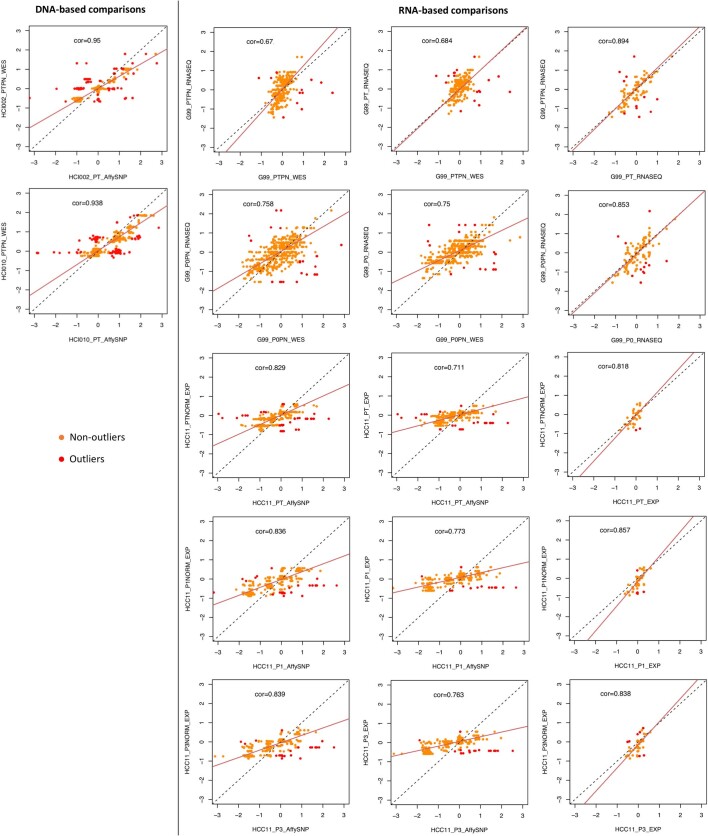
Comparison of copy number between different platforms. Pearson correlation and linear regression of the log_2_(CN ratio) of 100-kb windows binned from copy number segments of CNA profiles between matched patient tumor samples estimated from different platforms or analysis methods for examples shown in Fig. 2d. Outliers of the linear regression are identified by studentized residuals > 3 and < -3. RNA-seq and expression array samples denoted with ‘PN’ or ‘NORM’ are normalized by the median expression of normal samples.

Furthermore, expression-based calling had segmental resolution an order of magnitude worse than the DNA-based methods (Fig. 2a and Supplementary Figs. 14–17; median and mean segment sizes = 3.45 and 14.0 Mb for WES versus 36.0 and 51.9 Mb for RNASEQ NORM (*P* < 2.2 × 10^−^^16^) and 1.73 and 5.18 Mb for SNP versus 62.0 and 72.4 Mb for EXPARR NORM (*P* < 2.2 × 10^−^^16^)). The range of detectable copy number values was also superior for DNA-based methods (Fig. 2b; range of log_2_[copy number ratio] = –6.00–5.33 for WES versus –2.07–2.17 for RNASEQ NORM (*P* < 2.2 × 10^−^^16^) and –9.19–4.65 for SNP versus –1.40–1.89 for EXPARR NORM (*P* < 2.2 × 10^−^^16^)). In addition, there was a lack of correlation between the expression-based and DNA-based methods (range = 0.0541–0.942 for WES versus RNASEQ NORM and 0.00517–0.921 for SNP versus EXPARR NORM) (Fig. 2c and Supplementary Figs. 22 and 23). CNA estimates after tumor-based expression normalization resulted in further discordance with DNA-based copy number results (range = −0.182–0.929 (*P* = 0.0468) for WES versus RNASEQ TUM and −0.0274–0.847 (*P* = 2.20 × 10^−^^6^) for SNP versus EXPARR TUM). Many focal copy number events detected by DNA-based methods, as well as some larger segments, were missed by the expression-based methods (Extended Data Fig. 1b–e). Representative examples illustrating the superior resolution and accuracy from DNA-based estimates are given in Fig. 2d (correlations are shown in Extended Data Fig. 2).

### Concordance of PDXs with PTs and during passaging

Next, we adopted a pan-cancer approach to elucidate potential tumor type-independent copy number evolution in PDXs driven by the mouse host. We tracked the similarity of CNA profiles during tumor engraftment and passaging by calculating the Pearson correlation of gene-level copy number for samples measured on the same platform (see Methods, Extended Data Fig. 3 and Supplementary Figs. 24–60 and 62). All pairs of samples derived from the same PDX model were compared, yielding 501 PT–PDX pairs and 1,257 PDX–PDX pairs (Supplementary Note 4).

**Fig. 3:**
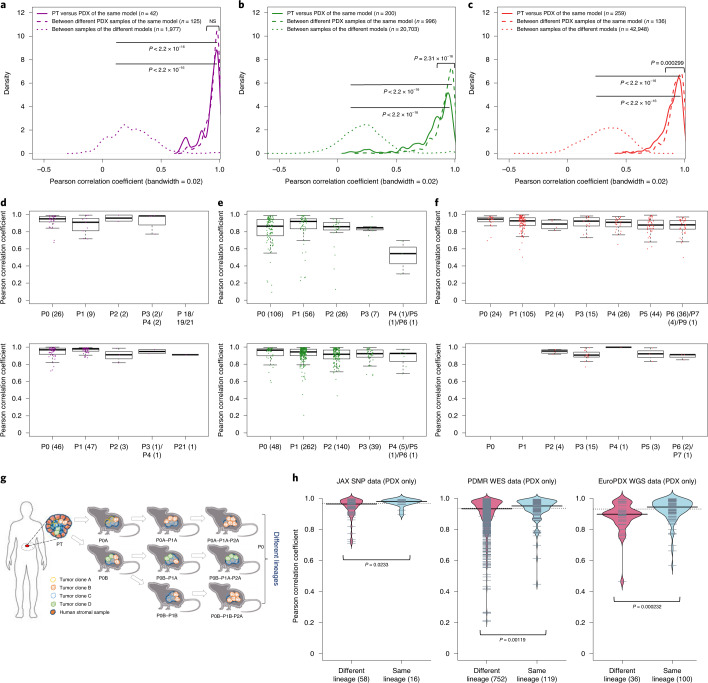
Comparisons of CNAs from PTs with early and late PDX passages. **a**–**c**, Distributions of Pearson correlation coefficients of gene-based copy number, estimated by SNP array (**a**), WES (**b**) and WGS (**c**) between: PT–PDX samples of the same model; PDX–PDX samples of the same model; and samples of different models from a common tumor type and contributing center. *P* values were computed by one-sided Wilcoxon rank-sum test (*P* > 0.05). Numbers of data points are indicated. NS, not significant. **d**–**f**, Distributions of Pearson correlation coefficients of gene-based copy number, estimated by SNP array (**d**), WES (**e**) and WGS (**f**) among PT and PDX passages of the same model. Comparisons relative to PT (top) and P0 (bottom) are shown (higher passages are shown in Extended Data Fig. 5). In the box plots, the center line represents the median, the box limits are the upper and lower quantiles, the whiskers extend to 1.5× the interquartile range and the dots represent all data points. **g**, Schematic of lineage splitting during passaging and expansion of tumors into multiple mice. This is a simplified illustration for passaging procedures in which different fragments of a tumor are implanted into different mice. **h**, Pearson correlation distributions for PDX sample pairs of different lineages and sample pairs within the same lineage, for (from left to right): JAX SNP array, PDMR WES and EuroPDX WGS datasets. *P* values were computed by one-sided Wilcoxon rank-sum test. For all box plots and violin plots, the numbers of pairwise correlations are indicated in the *x* axis labels.

For all DNA-based platforms, we observed strong concordance between matched PT–PDX and PDX–PDX pairs, and this was significantly higher than between different models from the same tumor type and the same center (*P* < 2.2 × 10^−^^16^) (Fig. 3a–c and correlation heatmaps in Supplementary Figs. 24–60). We observed no significant difference in the correlation values between PT–PDX and PDX–PDX pairs for SNP array data (median correlation = 0.950 for PT–PDX and 0.964 for PDX–PDX; *P* > 0.05), although there were small but statistically significant shifts for WES (PT–PDX = 0.874; PDX–PDX = 0.936; *P* = 2.31 × 10^−^^16^) and WGS data (PT–PDX = 0.914; PDX–PDX = 0.931; *P* = 0.000299). PT samples have a smaller CNA range than their derived PDXs (median ratios for PT/PDX and PDX/PDX, respectively = 0.832 and 0.982 (*P* = 0.000120) for SNP, 0.626 and 0.996 (*P* < 2.2 × 10^−^^16^) for WES and 0.667 and 1.00 (*P* < 2.2 × 10^−^^16^) for WGS; Supplementary Fig. 62b and Extended Data Fig. 4), which can be attributed to stromal DNA in PT samples diluting the CNA signal. In PDXs, the human stromal DNA is reduced^11,13^. The minimal effect for SNP array data confirms this interpretation as human stromal DNA contributions can be removed from SNP arrays based on allele frequencies of germline heterozygous sites, while such contributions to WES and WGS have higher uncertainties. We also performed intra-model comparisons using RNA-based approaches, which showed that the expression-based comparison of CNA profiles between PTs and PDXs can lead to overestimation of copy number changes during engraftment and passage (Supplementary Fig. 63 and Supplementary Note 5).

#### Late PDX passages maintain CNA profiles similar to early passages

Systematic mouse environment-driven evolution, if present, should reduce copy number correlations at each subsequent passage. However, we observed no apparent effect during passaging on the SNP, WES or WGS platforms (Fig. 3d–f and Extended Data Fig. 5). For example, the SNP data showed no significant difference between passages (Fig. 3d and Extended Data Fig. 5a). For those models having very late passages, there was a small but statistically significant correlation decrease compared with models with earlier passages (*P* < 8.98 × 10^−^^5^; Extended Data Fig. 6b), indicating that some copy number changes can occur over long-term passaging (Supplementary Fig. 35). However, even at these late passages, the correlations with early passages remained high (median = 0.896). In any given comparison, only a small proportion of the genes were affected by copy number changes (median = 2.72%; range = 1.03–11.9%). Genes that are deleted and subsequently gained in the later passages (top left quadrant of regression plots; Extended Data Fig. 6a) suggest selection of pre-existing minor clones as the key mechanism in these regions. For WES and WGS data, more variability in the correlations can be observed (Fig. 3e,f and Extended Data Fig. 5b,c), probably due to a few samples having more stromal contamination or low aberration levels (Supplementary Fig. 62b and Extended Data Fig. 4). However, the lack of downward trend over passaging was also apparent in these sets (Supplementary Note 6).

#### PDX copy number profiles trace lineages

Next, we compared the similarity of engrafted PDXs of the same model with the same passage number. Surprisingly, we discovered that these pairs were not more similar than pairs of PDXs from different passage numbers (Fig. 3d,e, Extended Data Fig. 5 and Supplementary Note 7). Such similarity in correlations suggested that copy number divergence might be associated with effects other than passaging. To further this analysis, we defined, for The Jackson Laboratory (JAX) SNP array and Patient-Derived Models Repository (PDMR) WES datasets, samples within a lineage as those differing only by consecutive serial passages, while we defined lineages as split when a tumor was divided and propagated into multiple mice (Fig. 3g). For the EurOPDX colorectal cancer (CRC) and WGS breast cancer (BRCA) datasets, such lineage splitting was due only to cases with initial engraftment of different fragments of the PT (that is, PDX samples of different passages were considered as different lineages if they originated from different PT fragments). We observed lower correlation between PDX samples from different lineages compared with within a lineage (Fig. 3h; *P* = 0.0233 for SNP; *P* = 0.00119 for WES; *P* = 0.000232 for WGS), despite a majority of these pairwise comparisons exhibiting high correlation (>0.9) (Supplementary Notes 8 and 9). This suggests that lineage splitting is often responsible for deviations in CNAs between samples, and that copy number evolution during passaging mainly arises from evolved spatial heterogeneity^24^.

We further explored whether the stability of copy number during engraftment and passaging is affected by mutations in genes known to impact genome stability (see Methods). Overall, we observed that the presence of mutations in such genes does not lead to increased copy number changes during PDX engraftment and passaging (Supplementary Note 10 and Supplementary Fig. 66).

### Genes with CNAs acquired during engraftment and passaging show no preference for cancer or treatment-related functions

**Fig. 4:**
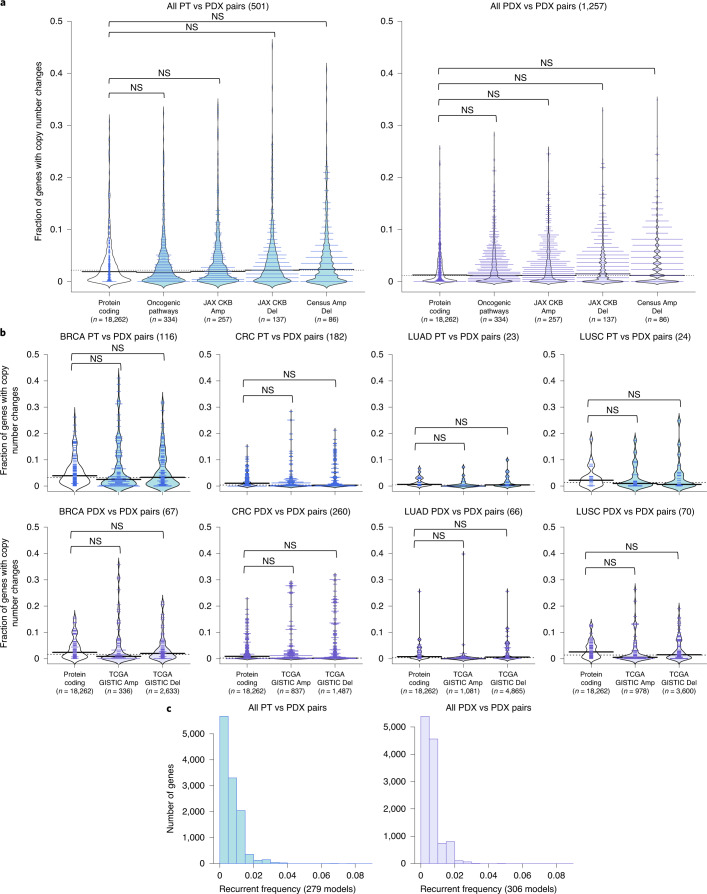
Cancer gene set analysis for copy number–altered genes during engraftment and passaging. **a**, Distribution of the proportion of altered genes between pairwise PT–PDX (left) and PDX–PDX comparisons (right) of the same model in various gene sets. Along the *x* axes from left to right are: protein-coding genes annotated by Ensembl; genes in oncogenic signaling pathways identified by TCGA; genes with copy number gain or overexpression (Amp) and genes with copy number loss or underexpression (Del) associated with therapeutic sensitivity or resistance or changes in drug response identified by JAX CKB; and genes from the Cancer Gene Census frequently altered by amplifications or deletions. CNA genes were identified by |residual| > 0.5 from a linear regression model. **b**, Distribution of the proportion of altered genes between pairwise PT–PDX (top) PDX–PDX comparisons (bottom) of the same model in various gene sets within BRCA, CRC, lung adenocarcinoma (LUAD) and lung squamous cell carcinoma (LUSC) models. Along the *x* axes from left to right are: protein-coding genes annotated by Ensembl, followed by significantly amplified and deleted genes from TCGA GISTIC analysis for the corresponding tumor type. For all violin plots, *P* values were computed by one-sided Wilcoxon rank-sum test (*P* > 0.1). The numbers of pairwise comparisons are indicated above each plot, whereas the numbers of genes per gene set are indicated in the *x* axis labels. **c**, Recurrence frequencies of protein-coding genes with CNAs, |residual| > 1, across all models in PT–PDX (left) and PDX–PDX comparisons (right). Number of models are indicated in the *x* axis labels.

**Extended Data Fig. 3:**
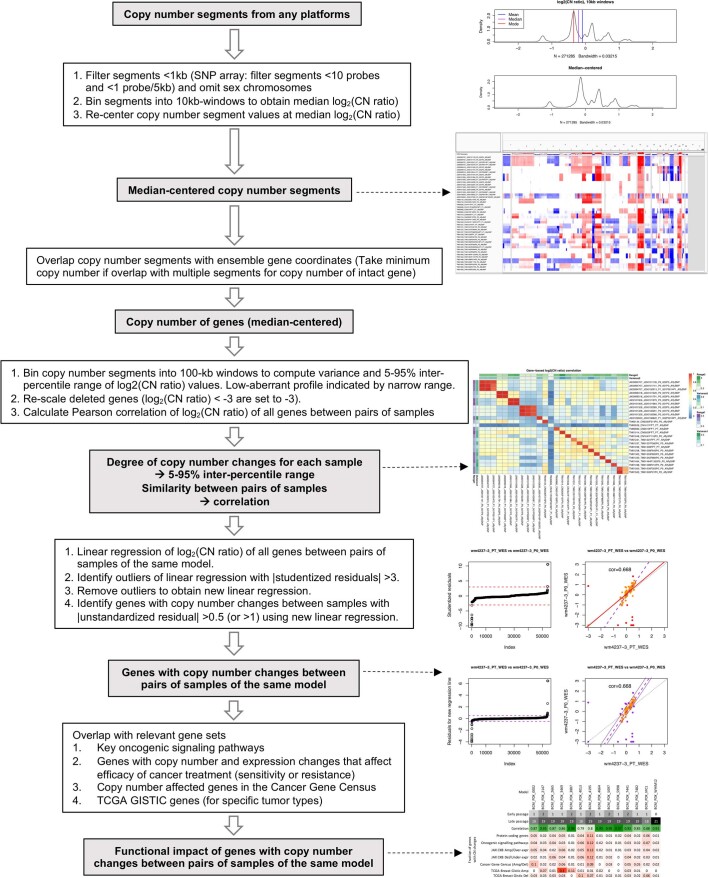
Analysis workflow to compare CNA between two samples of the same PDX model. A correlation and robust regression approach to quantify similarity of CNA profiles and identify genes with copy number changes between two samples.

**Extended Data Fig. 4:**
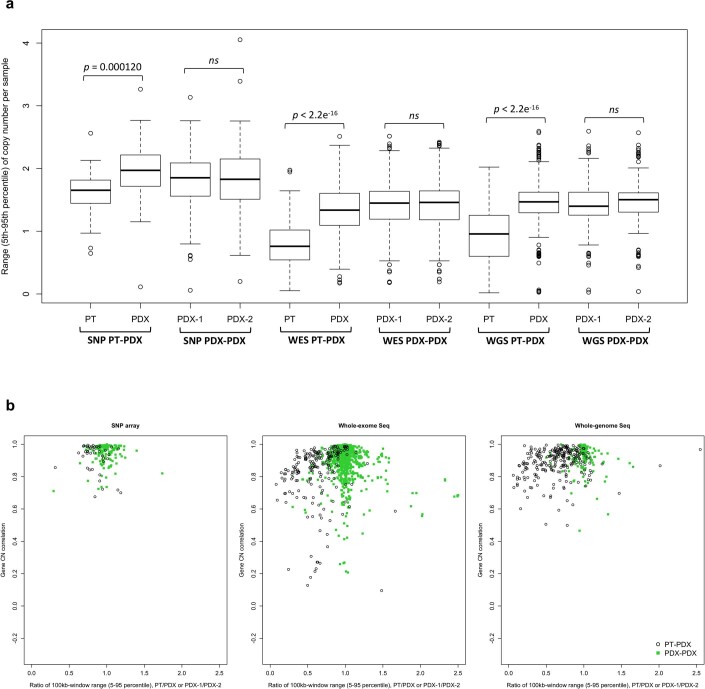
Correlations between PT-PDX and PDX-PDX pairs. **a**, The 5-95% inter-percentile range of CNA profiles between PT-PDX or PDX-PDX sample pairs from the same model on different platforms as shown in Fig. 3a–c. The 5-95% inter-percentile range of log_2_(CN ratio) values were calculated across all 100-kb windows per sample. *P*-values were computed by one-sided Wilcoxon rank sum test (ns: non-significant, *P* > 0.05). In the boxplots, the center line is the median, box limits are the upper and lower quantiles, whiskers extend 1.5× the interquartile range, and dots represent the outliers. **b**, Pearson correlation of the samples versus the ratio of 5-95% inter-percentile range between two samples (PT/PDX or PDX-1/PDX-2). Samples pairs with ratio of range much greater or less than 1 (that is one sample is much less aberrant than the other) tend to have lower correlations. PDX-1, lower passage PDX; PDX-2, later passage PDX or same passage PDX of different lineage.

**Extended Data Fig. 5:**
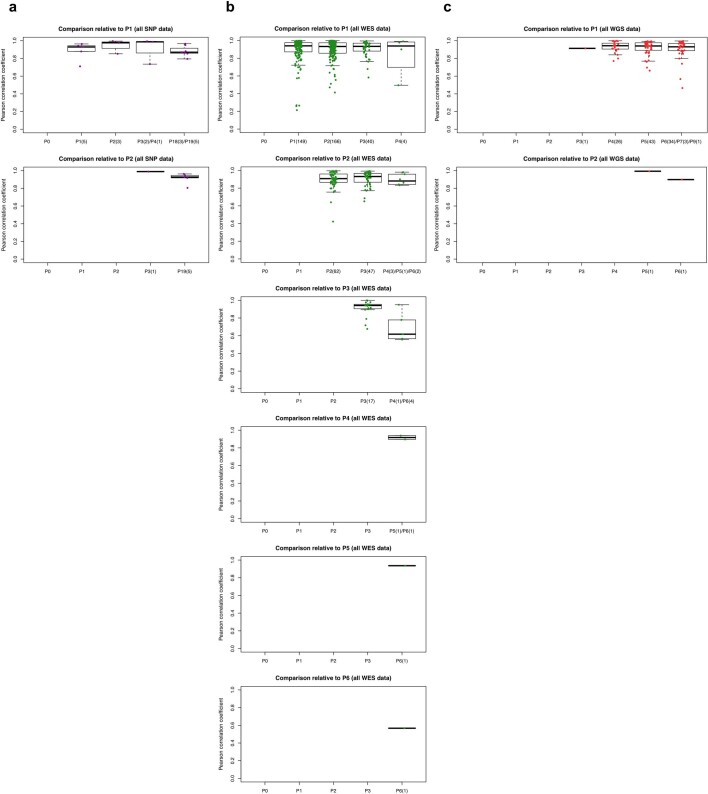
Distribution of Pearson correlation coefficients of gene-based copy number. **a-c**, Estimated by SNP array (**a**), WES (**b**), and WGS (**c**) between different combinations of patient tumor and PDX passages of the same model. Comparisons relative to passages P1 or later passages (refer to Fig. 3d–f for comparisons with PT and P0). In the boxplots, the center line is the median, box limits are the upper and lower quantiles, whiskers extend 1.5× the interquartile range, and dots represent all data points.

Next, we investigated which genes tend to undergo copy number changes. Genes with changes during engraftment or during passaging were identified based on a residual threshold with respect to the improved linear regression^39^ (see Methods and Extended Data Fig. 3). To test for functional biases, we compared CNA-altered genes with gene sets with known cancer- and treatment-related functions^40–43^ (see Methods). We calculated the proportion of altered genes for sample pairs from each model across all platforms and tumor types. In agreement with the high maintenance of CNA profiles described above, we found the proportion of altered protein-coding genes to be low (median and IQR, respectively = 1.90 and 4.11% for PT–PDX pairs and 1.25 and 3.60% for PDX–PDX pairs; Fig. 4a). Only 8.78% of PT–PDX pairs and 4.53% of PDX–PDX pairs showed alteration of >10% of their protein-coding genes. We observed no significant increase (*P* > 0.1) in alterations among any of the cancer gene sets compared with the background of all protein-coding genes, for either the PT–PDX or PDX–PDX comparisons. This provides evidence that there is no systematic selection for CNAs in oncogenic or treatment-related pathways during engraftment or passaging. Next, we considered tumor-type-specific effects, focusing on tumor types with larger numbers of models to ensure statistical power. We observed no significant increase in alterations in tumor-type-specific driver gene sets significantly altered in TCGA^44–47^ compared with the background (*P* > 0.1) for either PT–PDX or PDX–PDX comparisons (Fig. 4b and Supplementary Note 11).

#### Low recurrence of altered genes across models

We observed a very low recurrent frequency (Fig. 4c; see Methods), with only 12 and two genes recurring at >5% frequency for PT–PDX and PDX–PDX comparisons, respectively (Supplementary Table 4). No gene had a recurrence frequency higher than 8.96% (Supplementary Note 12). None of these recurrent genes overlapped cancer- or treatment-related gene sets, nor did they intersect genes (*n* = 3) reported by Ben-David et al.^23^ to have mouse-induced copy number changes associated with drug response in the Cancer Cell Line Encyclopedia (CCLE)^48,49^ database (Supplementary Note 12).

### Absence of CNA shifts in 130 WGS PT, early-passage PDX and late-passage PDX trios

**Fig. 5:**
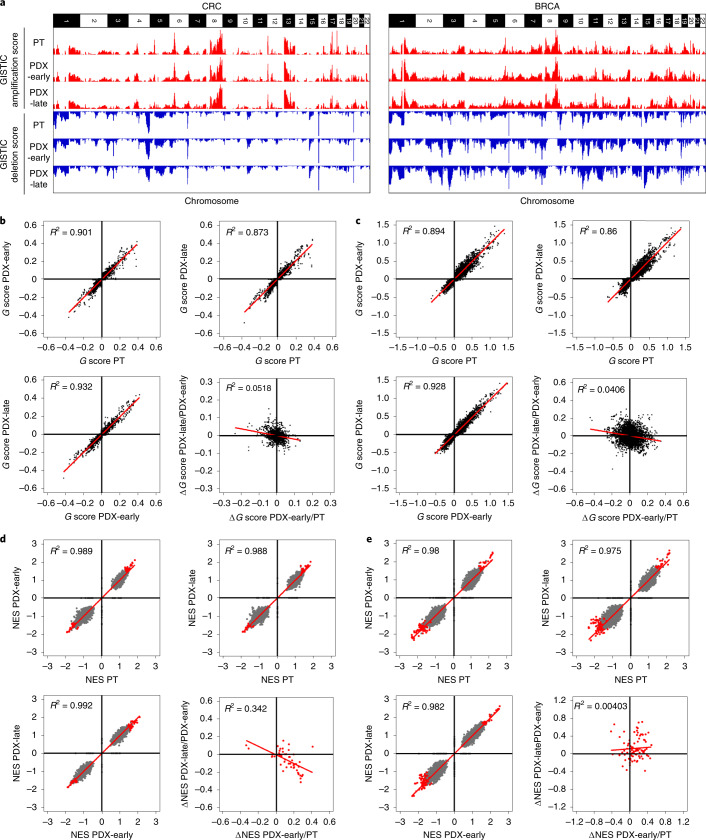
Absence of mouse-driven recurrent CNAs during engraftment and propagation of CRC and BRCA PDXs. **a**, Bar charts representing genome-wide *G* scores for amplifications and deletions in each of the three cohorts of CRC (left; 87 trios) and BRCA (right; 43 trios): PT, PDX-early (P0–P1 for CRC; P0–P2 for BRCA) and PDX-late (P2–P7 for CRC; P3–P9 for BRCA). **b**,**c**, Scatter plots comparing gene-level *G* scores between each of the three cohorts for CRC (**b**) and BRCA (**c**). The bottom-right panels of **b** and **c** show scatter plots comparing Δ*G* values from PT to PDX-early and from PDX-early to PDX-late. **d**,**e**, Scatter plots comparing GSEA NESs for gene sets between each of the three cohorts for CRC (**d**) and BRCA (**e**). The bottom-right panels of **d** and **e** show scatter plots comparing ΔNES from PT to PDX-early and from PDX-early to PDX-late. Gray data points represent all gene sets, whereas red data points represent gene sets significantly enriched in at least one of the three cohorts (that is, PT, PDX-early or PDX-late).

Next, we investigated whether recurrent CNA changes occur in PDXs in a tumor-type-specific fashion. To this aim, we analyzed further the WGS-based CNA profiles of large metastatic CRC and BRCA series, composed of matched trios of PT, PDX at early passage (PDX-early) and PDX at later passage (PDX-late). Genomic Identification of Significant Targets in Cancer (GISTIC)^50,51^ analysis was applied separately to identify recurrent CNAs in each PT, PDX-early and PDX-late cohort of CRC and BRCA (see Methods and Supplementary Table 6). As expected, CRCs and BRCAs generated different patterns of significant CNAs but, within each tumor type, GISTIC profiles of the PT, PDX-early and PDX-late cohorts were virtually indistinguishable (Fig. 5a, Extended Data Fig. 7 and Supplementary Note 13), demonstrating no gross genomic alteration systematically acquired or lost in PDXs.

We then carried out gene-level analysis, where each gene was attributed the GISTIC score (*G* score) of the respective segment (Supplementary Table 7). In both the CRC and BRCA cohorts, gene-level *G* scores of the PTs were highly correlated with the respective PDX-early and PDX-late cohorts (Fig. 5b,c). Moreover, PT versus PDX correlations were comparable to PDX-early versus PDX-late correlations. To search for progressive shifts, we compared the change in *G* score (Δ*G*): (1) from tumor to PDX-early; and (2) from PDX-early to PDX-late. Correlations in these two Δ*G* values were absent or even slightly negative (bottom-right panels of Fig. 5b,c and Supplementary Note 13). Overall, these results confirmed the absence of systematic CNA shifts in PDXs, even under high-resolution gene-level analysis. To evaluate the possibility of systematic copy number evolution at the pathway level in these trios, we performed gene set enrichment analysis (GSEA)^52,53^ using *G* scores to rank genes in each cohort (see Methods and Supplementary Note 14). For both CRC and BRCA, the normalized enrichment score (NES) profiles for the ~8,000 gene sets of PTs were highly correlated with the respective PDX-early and PDX-late cohorts (Fig. 5d,e). Moreover, PT versus PDX correlations were comparable to PDX-early versus PDX-late correlations. To search for progressive shifts, we calculated for each significant gene set ΔNES values between PT and PDX-early, as well as between PDX-early and PDX-late. Similar to what was observed for Δ*G*, correlations were absent or at most slightly negative (bottom-right panels of Fig. 5d,e), confirming the absence of systematic CNA-based functional shifts in PDXs.

### CNA evolution across PDXs is no greater than variation in patient multiregion samples

**Fig. 6:**
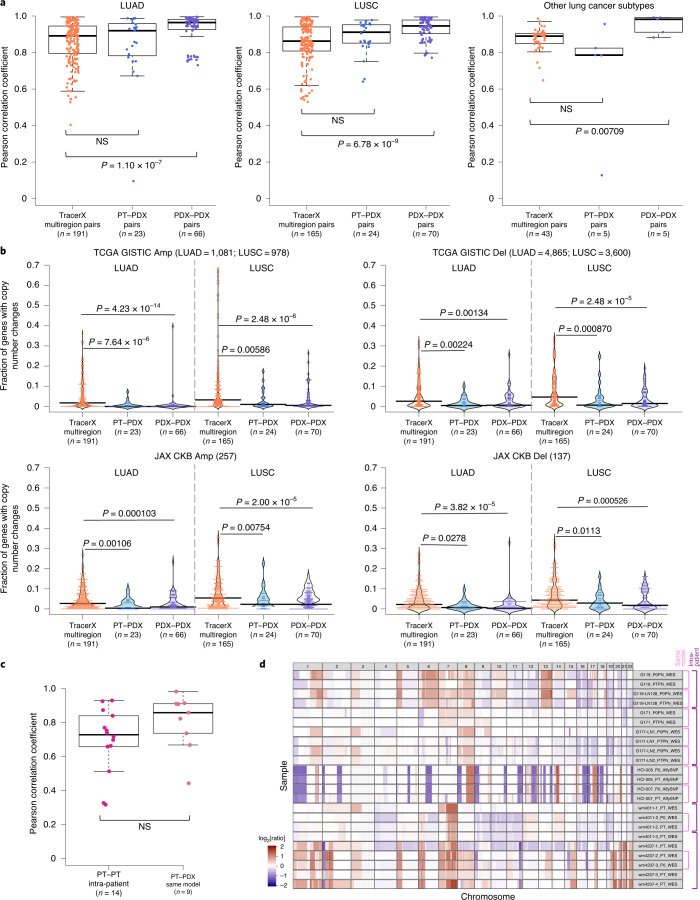
Comparison of CNA variation during PDX engraftment and passaging with CNA variation among patient multiregion, tumor relapse and metastasis samples. **a**, Distributions of Pearson correlation coefficients of gene-based copy number for lung adenocarcinoma (LUAD), lung squamous cell carcinoma (LUSC) and other lung cancer subtypes, comparing different datasets. From left to right on the *x* axis, these include: multiregion tumor samples of the same patient from TRACERx (*n* = 92 PTs; *n* = 295 multiregion samples); PT–PDX samples of the same model; and PDX–PDX samples of the same model. *P* values were computed by two-sided Wilcoxon rank-sum test (*P* > 0.05). **b**, Distributions of the proportion of altered genes between multiregion tumor pairs from TRACERx, as well as PT–PDX and PDX–PDX pairs, for various gene sets for LUAD and LUSC. The gene sets and CNA thresholds are the same as in Fig. 4. TCGA GISTIC Amp/Del and JAX CKB Amp Del gene sets are shown (other gene sets are shown in Extended Data Fig. 8). *P* values were computed by one-sided Wilcoxon rank-sum test. The numbers of genes per gene set are indicated above each plot. **c**, Distributions of Pearson correlation coefficients of gene-based copy number between intra-patient PT pairs (*n* = 14; primary, relapse or metastasis) from the same patient (*n* = 5) and corresponding PT–PDX pairs (derived from the same model; a different PT sample from the same patient generates a different model) for the same set of patients. *P* values were computed by two-sided Wilcoxon rank-sum test (*P* > 0.05). For all box and violin plots, the numbers of pairwise comparisons are indicated in the *x* axis labels. In all box plots the center line represents the median, the box limits are the upper and lower quantiles, the whiskers extend to 1.5× the interquartile range and the dots represent all data points. **d**, CNA profiles of PT and PDX samples from patients with PDX models derived from multiple PT collections (primary, relapse and metastasis). Each sample ID is denoted by the model ID, passage number and platform used (see Supplementary Data 1).

As a reference for the treatment relevance of PDX-specific evolution, we compared this with the levels of copy number variation in multiregion samples of PTs. For this, we used copy number data from multiregion sampling of non-small-cell lung cancer from the TRACERx Consortium^54^, performing analogous CNA correlation and gene analyses between multiregion pairs (Supplementary Fig. 69). We observed no significant differences in correlation (*P* > 0.05) between patient multiregion and lung cancer PT–PDX pairs, while PDX–PDX pairs in fact showed significantly better correlation than the multiregion pairs (*P* < 0.05; Fig. 6a), consistent across all lung cancer subtypes. Cancer gene set analyses confirmed these results, with multiregion samples showing greater differences than either PT–PDX or PDX–PDX comparisons, across all cancer gene sets considered (*P* < 0.05; Fig. 6b and Extended Data Fig. 8). These results show that PDX-associated CNA evolution is no greater than what patients experience naturally within their tumors. Our PDX collection also contains a few cases in which the PT was assayed at multiple time points (relapse/metastasis) or multiple metastatic sites, allowing for controlled comparison of intra-patient variation versus PDX evolution (Supplementary Figs. 3, 4 and 7). Despite a lower median in correlations among intra-patient samples, the difference compared with CNA evolution during engraftment (PT–PDX) was not statistically significant (*P* > 0.05; Fig. 6c). CNA profiles for these samples are shown visually in Fig. 6d.

## Discussion

Here, we have investigated the evolutionary stability of PDXs—an important model system for which there have been previous reports of mouse-induced copy number evolution. To better address this, we assembled a collection of CNA profiles of PDX models, comprising PDX models with multiple passages and their originating PTs. Our analysis showed the reliability of copy number estimation by DNA-based measurements over RNA-based inferences, which are substantially inferior in terms of resolution and accuracy (Supplementary Note 15). The importance of DNA measurements is supported by the inconsistent conclusions by two independent studies (Ben-David et al.^23,55^ and Mer et al.^56^) on the same PDX expression array dataset by Gao et al.^15^. Ben-David et al. concluded that drastic copy number changes, driven by mouse-specific selection, often occur within a few passages. In contrast, Mer et al. reported high similarity between passages of the same PDX model based on direct correlations of gene expression, consistent with our findings in large, independent DNA-based datasets.

The copy number shifts inferred by Ben-David et al. were inherently impacted by major technical issues. First, the microarray signal for PT samples is diluted by introgressed human stromal cells, while in PDXs mouse stromal transcripts only hybridize to a fraction of the human probes^57^. Consequently, PT samples with substantial stromal content would display a reduced signal compared with the corresponding PDX, which can lead to an erroneous inference of systematic increase in aberrations during PDX engraftment when gain/loss regions are directly compared. Second, the mouse host microenvironment can affect the transcriptional profile of the PDX tumor^58^ and the quantity of mouse stroma can vary across passages. This can result in variability in the expression signal, which can be wrongly inferred as copy number changes, both from the tumor itself and through cross-hybridization of mouse RNA to the human microarray. Although improved concordance in expression between PT and PDX can be achieved with RNA-seq with the removal of mouse reads^59,60^, we observed that expression-based copy number inferences still have low resolution and robustness. Hence, many cancer-driving genes, which are found mainly in focal events with a size of 3 Mb or lower^61–64^, cannot be evaluated for PDX-specific alterations. These issues are further worsened by the lack of tissue-matched normal gene expression profiles for calibration^37^, which have been only intermittently available but can substantially impact copy number inferences. Because of these considerations, the question of how much PDXs evolve as a consequence of mouse-specific selective pressures cannot be adequately addressed by expression data.

The studies we have presented here take into account the above issues by the use of DNA data, as well as by assessing copy number changes by pairwise correlation/residual analysis to control for systematic biases, and they overall confirm the high retention of CNA profiles from PDX engraftment to passaging. We did observe larger deviations between PT–PDX than in PDX–PDX comparisons, although this was probably due to dilution of the PT signal by human stromal cells. Interestingly, we found that a major contributor to the differences between PDX samples is lineage-specific drift associated with the splitting of tumors into fragments during PDX propagation. This spatial evolution within tumors appears to affect sample comparisons more than time or the number of passages. This suggests that PDX expansion and passaging is the bottleneck of copy number evolution in PDXs, reflecting stochasticity in sampling within spatially heterogeneous tumors (Supplementary Note 16).

A challenge for evaluating any model system is that there is no clear threshold for genomic change that determines whether the model will still reflect patient response. Genetic variation among multiregion samples within a patient can shed light on this point^54,65–68^ since the goal of a successful treatment would be to eradicate all of the multiple regions of the tumor. We found that the copy number differences between PT and PDX are no greater than the variations among multiregion tumor samples or intra-patient samples. Thus, concerns about the genetic stability of the PDX system are likely to be less important than the spatial heterogeneity of solid tumors themselves. This result is consistent with our results on lineage effects during passaging, which indicate that intratumoral spatial evolution is the major reason for genetic drift.

**Extended Data Fig. 6:**
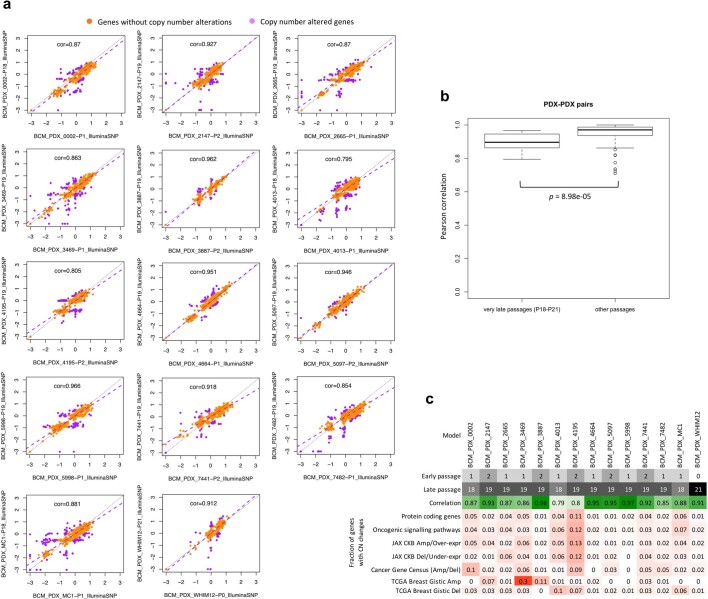
Comparison of CNA between early and very-late passages. In the BCM SNP array breast cancer dataset. **a**, Correlation and robust regression of gene-based copy number between early (P0-P2) and very-late passages (P18-P21) of the same model. Genes with copy number changes between the passages are identified by |residual| > 0.5. Some genes show signs of complete deletion (log_2_(CN ratio) < -2) but then reappear in later passages. This can only be explained by the early and late passages being dominated by different pre-existing subclones. **b**, Distribution of Pearson correlation coefficients of gene-based copy number between early and very-late passages of the same model (14 models/pairwise correlations) compared to correlation coefficients between lower passages denoted as ‘other passages’ (< P4). Correlation for ‘other passages’ are based on models from all other non-BCM SNP array datasets (111 pairwise correlations). *P*-values were computed by one-sided Wilcoxon rank sum test. In all boxplots, the center line is the median, box limits are the upper and lower quantiles, whiskers extend 1.5× the interquartile range, and dots represent outliers. **c**, Summary of passage numbers, copy number correlation, and fraction of genes of different gene sets with copy number changes (|residual| > 0.5) between passages of each breast cancer model.

We observed no evidence for systematic mouse environment-induced selection for cancer- or treatment-related genes via copy number changes, although individual cases vary (see example in Extended Data Fig. 6c). Moreover, only a small fraction of sample pairs (2.44%; 43 out of 1,758) showed large CNA discordance (see Methods), suggesting that clonal selection out of a complex population is rare. These results indicate that the variations observed in PDXs are mainly due to spontaneous intratumoral evolution, rather than murine pressures (Supplementary Note 17).

In summary, our in-depth tracking of CNAs throughout PDX engraftment and passaging confirms that tumors engrafted and passaged in PDX models maintain a high degree of molecular fidelity to the original PTs, thus verifying their suitability for preclinical drug testing. At the same time, our study does not rule out that PDXs will evolve in individual trajectories over time; thus, for therapeutic dosing studies, the best practice is to confirm the existence of expected molecular targets and obtain sequence characterizations in the cohorts used for testing as close to the time of the treatment study as is practical.

## Methods

### Experimental details for sample collection, PDX engraftment and passaging, and array or sequencing

For details of sample collection, abbreviations of PDX model sources, PDX engraftment and passaging, and array/sequencing, see the Supplementary Methods.

### Consolidating tumor types from different datasets

As the terminology of tumor types/subtypes by the different contributing centers was not consistent, we used the Disease Ontology database^69^ (http://disease-ontology.org/), along with cancer types listed on the NCI website (https://www.cancer.gov/types) and in TCGA publications^70,71^, to unify and group the tumor types/subtypes under broader terms, as shown in Fig. 1 and Supplementary Table 2.

### CNA estimation methods

#### SNP array

The estimation of CNA profiles from SNP array was detailed previously^34^. In short, for Affymetrix Human SNP 6.0 arrays, PennCNV-Affy and Affymetrix Power Tools^72^ were used to extract the B-allele frequency and log[R ratio] from the CEL files. Due to the absence of paired normal samples, the allele-specific signal intensity for each PDX tumor was normalized relative to 300 randomly selected sex-matched Affymetrix Human SNP 6.0 array CEL files obtained from the International HapMap Project^73^. For Illumina Infinium Omni2.5Exome-8 SNP arrays (version 1.3 and version 1.4 kits), the Illumina GenomeStudio software was used to extract the B-allele frequency and log[R ratio] from the signal intensity of each probe. The single sample mode of the Illumina GenomeStudio was used, which normalizes the signal intensities of the probes with an Illumina in-house dataset. The single tumor version of ASCAT^33^ (version 2.4.3 for JAX SNP data and version 2.5.1 for SIBS SNP data) was used for GC correction, predictions of the heterozygous germline SNPs based on the SNP array platform, and estimation of ploidy, tumor content and allele-specific copy number segments. The resultant copy number segments were annotated with the log_2_[ratio of the total copy number relative to the predicted ploidy from ASCAT].

#### WES data

Aligned BAMs (see Supplementary Methods) were subset to the target region by GATK 4.0.5.1, and SAMTools^74^ version 0.1.18 was used to generate the pileup for each sample. Pileup data were used for CNA estimation, as calculated with Sequenza^29^ version 2.1.2. Both tumor and normal data, which utilized the same capture array, were used as input. pileup2seqz and GC-windows (-w 50) modules from sequenza-utils.py utility were used to create the native seqz format file for Sequenza and to compute the average GC content in sliding windows from the hg38 genome, respectively. We ran the three Sequenza modules with these modified parameters (sequenza.extract: assembly = ‘hg38’, sequenza.fit: chromosome.list = 1:23 and sequenza.results: chromosome.list = 1:23) to estimate the segments of copy number gains/losses. Finally, segments lacking read counts, in which ≥50% of the segment had zero read coverage, were removed. A reference implementation of this workflow (Supplementary Fig. 71) was developed and deployed in the Cancer Genomics Cloud by Seven Bridges (https://cgc.sbgenomics.com/public/apps#pdxnet/pdx-wf-commit2/wes-cnv-tumor-normal-workflow/ and https://cgc.sbgenomics.com/public/apps#pdxnet/pdx-wf-commit2/pdx-wes-cnv-xenome-tumor-normal-workflow/).

#### Low-pass WGS data

For EuroPDX CRC liver metastasis data, raw copy number profiles for each sample were estimated using the QDNAseq^75^ R package (version 1.20) by dividing the human reference genome into non-overlapping 50-kb windows and counting the number of reads (see Supplementary Methods) in each bin. Bins in problematic regions were removed^76^. Read counts were corrected for GC content and mappability using a LOESS regression, median normalized and log_2_ transformed. Values below –1,000 in each chromosome were floored to the first value greater than –1,000 in the same chromosome. Raw log_2_[ratio] values were then segmented using the ASCAT^33^ algorithm implemented in the ASCAT R package (version 2.0.7). For EuroPDX BRCA tumors, raw copy number profiles were estimated for each sample by dividing the human reference genome into non-overlapping 20-kb windows and counting the number of reads (see Supplementary Methods) in each bin. Only reads with a mapping quality of at least 37 were considered. Bins within problematic regions (that is, multimapper regions) were excluded. Downstream analysis to estimate copy number was conducted as described above.

#### RNA-seq and gene expression microarray data

For expression-based copy number inference, we referred to the previous protocols for e-karyotyping and CGH-Explorer^37,38,77,78^. For each cancer type, expression values (see Supplementary Methods) of tumor samples and corresponding normal samples were merged in a single table, and gene identifiers were annotated with chromosomal nucleotide positions. Genes located on sex chromosomes were excluded. Genes with values below one transcript per million (TPM) (RNA-seq) or probeset log_2_ values below 6 (microarray) in more than 20% of the analyzed dataset were removed. Remaining gene expression values below the thresholds were respectively raised to 1 TPM or a log_2_ value of 6. In the case of multiple transcripts (RNA-seq) or probesets (microarray) per gene, the one with the highest median value across the entire dataset was selected. According to the e-karyotyping protocol, the sum of squares of the expression values relative to their median expression across all samples was calculated for each gene, and 10% of the most highly variable genes were removed. For each gene, the median log_2_[expression] value in normal samples was subtracted from the log_2_[expression] value in each tumor sample and subsequently input into CGH-Explorer. For tumor-only datasets, the median log_2_[expression] value in the same set of tumor samples was instead subtracted. The preprocessed expression profiles of each sample were individually analyzed using CGH-Explorer (http://heim.ifi.uio.no/bioinf/Projects/CGHExplorer/). Piecewise constant fit analysis was carried out to call copy number according to parameters previously reported^23^: least allowed deviation = 0.25; least allowed aberration size = 30; winsorize at quantile = 0.001; penalty = 12; and threshold = 0.01.

### Statistical methods

All statistical analyses for data comparison were performed using either a one- or two-tailed Wilcoxon rank-sum test, a two-tailed Kolmogorov–Smirnov test or a one-tailed Wilcoxon signed-rank test.

### Filtering and gene annotation of copy number segments

Copy number segments with a log_2_[copy number ratio] estimated from the various platforms were processed in the following steps (Extended Data Fig. 3). Segments <1 kb were filtered based on the definition of CNA^79^. In addition, SNP array segments had to be covered by more than ten probes, with an average probe density of one probe per 5 kb. The copy number segments were then binned into 10-kb windows to derive the median log_2_[copy number ratio], which was subsequently used to re-center the copy number segments. Median-centered copy number segments were visualized using IGV^80^ version 2.4.13 and GenVisR^81^ version 1.16.1. The median-centered copy number of genes was calculated by intersecting the genome coordinates of copy number segments with the genome coordinates of genes (Ensembl Genes 93 for human genome assembly GRCh38 and Ensembl Genes 96 for human genome assembly GRCh37). In the case where a gene overlapped multiple segments, the most conservative (lowest) estimate of copy number was used to represent the copy number of the entire intact gene.

### Comparison of copy number gains and losses

For the comparison of resolution, the range of copy number values and the frequency of gains and losses between different platforms and analysis methods, we defined copy number gain or loss segments as log_2_[copy number ratio] > 0.1 (for gain) and log_2_[copy number ratio] < −0.1 (for loss).

### Correlation of CNA profiles

The overall workflow to compare CNA profiles is shown in Extended Data Fig. 3. PDX samples without passage information were omitted in the following downstream analysis. The copy number segments were binned into 100-kb windows or smaller using BEDTools^82^ version 2.26.0, and the variance of the log_2_[copy number ratio] and 5–95% inter-percentile range of the log_2_[copy number ratio] values across all of the bins were calculated as a measure of the degree of aberration for each CNA profile. A non-aberrant profile results in a low variance or range. While variance can be biased for CNA profiles with small segments of extreme gains or losses, we preferred use of the 5–95% inter-percentile range of log_2_[copy number ratio] to identify samples with a low degree of aberration, such that a narrow range indicates that ≥90% of the genome has very low-level gains and losses. The similarity of two CNA profiles is quantified by the Pearson correlation coefficient of the log_2_[copy number ratio] of 100-kb windows binned from segments or genes between two samples. Gene-based and segment-based (100-kb-window) correlations were highly similar (data not shown). Using correlation avoided the issue of making copy number gain and loss calls based on thresholds. Sample-based variations in the baseline due to median normalization and the range in copy number values could introduce further inconsistencies in gain and loss calls between samples. Such variations are further impacted by sample-specific variation in human stromal contamination or the sensitivity of copy number detection by different platforms. As median centering of each CNA profile approximates normalization by the sample ploidy, we confirmed that, in general, ploidy (estimated from ASCAT analysis of SNP array samples) had no association with the copy number correlation values (Pearson’s product moment correlation = 0.0248; *P* > 0.05). However, one caveat of our approach is that it cannot distinguish genome-wide multiplication of ploidy between samples, as the correlation statistic is invariant to such genome-wide transformations. As such, we cannot assess whether ploidy changes occur between samples of a given model.

#### Comparison of CNA profiles between different platforms

The copy number segments of each pair of data were intersected and binned into 100-kb windows or smaller using BEDTools. The Pearson correlation coefficient and linear regression model were calculated for the log_2_[copy number ratio] of the windows. Windows with discrepant copy numbers were identified by outliers of the linear regression model defined by |studentized residual| > 3. These outlier windows were mapped to their corresponding segments to identify the size of CNA events that were discordant between the different copy number estimation methods. The proportion of the genome-discordant CNA was calculated from the summation of the outlier windows.

#### Identification of genes with CNA between different samples of the same model

To compare the CNA profiles between different samples (PT or PDX) of the same model, the Pearson correlation coefficient and linear regression model were calculated for the log_2_[copy number ratio] of the genes for each pair of data. Before that, deleted genes with a log_2_[copy number ratio] of <−3 were rescaled to −3 to avoid large shifts in the correlation coefficient and linear regression model due to extremely negative values on the log scale. Extreme outliers of the linear regression model defined by |studentized residual| > 3 were removed to derive an improved linear regression model^39^ not biased by a few extreme values. Genes with copy number changes between the samples were identified by the difference in log_2_[copy number ratio] relative to the improved linear regression model of |standard residual| < 0.5. We also removed some samples with low correlation due to sample mislabeling as they displayed high correlation with samples from other models. We also omitted samples with low correlation values (<0.6), which resulted from non-aberrant CNA profiles in genomically stable tumors (5–95% inter-percentile range of log_2_[copy number ratio] < 0.3; Supplementary Fig. 62).

#### Identification of aberrant sample pairs with highly discordant CNA profiles

Aberrant CNA profiles were identified based on the 5–95% inter-percentile range of log_2_[copy number ratio] > 0.5, for both samples. Sample pairs with a Pearson correlation of < 0.6 were selected as having highly discordant CNA profiles between them.

#### Association of mutations with copy number correlations

Mutational calls for each WES sample used in this study were obtained using a tumor normal variant calling workflow developed for PTs and PDXs^35^. Subsequently, genes with either germline or somatic variants that passed through the quality filters (FILTER = PASS or germline) and IMPACT = MODERATE or HIGH by SnpEff (version 4.3) annotation were labeled as mutated. Otherwise, they were labeled as wild type. For SNP array and WGS data, we collected the mutational status (wild type or mutated) of *TP53*, *BRCA1* and *BRCA2* per model where available, which may or may not have been obtained from the exact same tumor samples used in this study. For the JAX SNP array dataset, variant calls (tumor only) were made from various targeted sequencing approaches (TruSeq Amplicon Cancel Panel, JAX Cancer Treatment Profile panel and WES). The workflow and filtering criteria to call mutations is described elsewhere^34^. For the HCI SNP array data, mutations were obtained from WES (unpublished data) and were filtered for frameshift, inframe, missense, nonsense and splice-site mutations. For the BCM SNP array data, mutational status was obtained from clinical samples by immunohistochemistry or Sequenom^83^ (unpublished data). For the WGS data, mutations were obtained from WES or targeted panel sequencing^84^ (unpublished data), and high-quality and probable functional mutations were retained. For each sample pair with copy number correlations, the mutational status of *TP53* or *BRCA* was obtained for each individual sample for the WES data, while the mutational status was available on a per-model basis for the SNP and WGS data. *BRCA* was labeled as mutated when either *BRCA1* or *BRCA2* was mutated. For mutations in DNA repair genes^85^ from the WES data, each pair of samples was classified as mutated if any DNA repair gene was reported to be mutated in either sample.

### Annotation with gene sets with known cancer- or treatment-related functions

A low copy number change threshold (|log_2_[copy number ratio] change| > 0.5) was selected to include genes with subclonal alterations. Copy number–altered genes (|residual| > 0.5) were annotated by various gene sets with cancer- or treatment-related functions gathered from various databases and publications (Extended Data Fig. 3):

1. Genes in ten oncogenic signaling pathways curated by TCGA that were found to be frequently altered in different cancer types^40^;
2. Genes with a gain in copy number or expression or a loss in copy number or expression that conferred therapeutic sensitivity, resistance or an increase/decrease in drug response from the JAX Clinical Knowledgebase (CKB)^41,42^ (based on literature curation (https://ckbhome.jax.org/; as of 18 June 2019).
3. Genes with evidence of promoting oncogenic transformation by amplification or deletion from the Cancer Gene Census^43^ (COSMIC version 89); and
4. Significantly amplified or deleted genes in TCGA cohorts of BRCA^44^, CRC^45^, lung adenocarcinoma^46^ and lung squamous cell carcinoma^47^ by GISTIC analysis, which identified significantly altered genomic driver regions that can be used to differentiate between tumor types and subtypes.

### Identification of genes with recurrent copy number changes

A stringent CNA threshold (|log_2_[copy number ratio] change| > 1.0 with respect to the linear regression model) was selected to distinguish genes with a possible functional impact. Genes with |residual| > 1.0 with respect to the improved regression linear model (without discriminating gain or loss) were selected for each pairwise comparison between different samples of the same model. Pairwise cases in which genes were deleted in both samples (log_2_[copy number ratio] ≤ −3) were omitted. The recurrent frequency for each gene across all models was calculated on a model basis such that genes with a copy number between multiple pairs of the same model were counted once. This avoided bias towards models with many samples of similar copy number changes between the different pairs.

### Drug response analysis using CCLE data

We developed a pipeline to evaluate gene copy number effects on drug sensitivity^86,87^ by using CCLE^48,88^ cell line genomic and drug response data (Cancer Therapeutics Response Portal version 2). We downloaded the CCLE drug response data from the Cancer Therapeutics Response Portal (www.broadinstitute.org/ctrp) and CCLE gene-level CNA and gene expression data from the DepMap data portal (public_19Q1_gene_cn.csv and CCLE_depMap_19Q1_TPM.csv; https://depmap.org/portal/download/). For CCLE drug response data, we used the area-under-the-concentration-response curve (AUC) sensitivity scores for each cancer cell line and each drug. In total, we collected gene-level log_2_[copy number ratio] data derived from the Affymetrix SNP 6.0 platform from 668 pan-cancer CCLE cell lines, with a total of 545 cancer drugs tested. With the CCLE gene-level CNA and AUC drug sensitivity scores, we performed gene–drug response association analyses for genes with recurrent copy number changes. Pearson correlation *P* values between each gene’s log_2_[copy number ratio] and each drug’s AUC score across all cell lines were calculated, and *q* values were calculated by multiple-testing Bonferroni correction. Significant gene CNA–drug associations were kept (*q* value < 0.1) to further evaluate gene expression and drug response associations. If a gene’s expression was also significantly correlated with AUC drug sensitivity scores, particularly in the same direction (either positively or negatively correlated) as the gene CNA–drug association, that gene would be considered as significantly correlated with drug response based on both its CNA and gene expression.

### GISTIC analysis of WGS data

We carried out GISTIC analysis to identify recurrent CNAs by evaluating the frequency and amplitude of observed events. To obtain perfectly matching and comparable PT–PDX cohorts for GISTIC analysis, CRC trios in which at least one sample displayed non-aberrant CNA profiles were excluded from the analysis, resulting in a total of 87 triplets. The GISTIC^51^ algorithm (GISTIC 2.0 version 6.15.28) was applied on the segmented profiles using the GISTIC GenePattern module (https://cloud.genepattern.org/), with default parameters and the genome reference files Human_Hg19.mat for the EuroPDX CRC data and hg38.UCSC.add_miR.160920.refgene.mat for the EuroPDX BRCA data. For each dataset, GISTIC provides separate results (including segments, *G* scores and false discovery rate *q* values) separately for recurrent amplifications and recurrent deletions. Deletion *G* scores were assigned negative values for visualization. We observed that the *G* score range was systematically lower in PT cohorts, which was probably the result of the dilution of CNA by normal stromal DNA. In contrast, human stromal DNA in PDX samples was lower or negligible. To account for this difference in gene-level *G* scores, PDXs at early and late passages were scaled with respect to PT gene-level *G* score values using global linear regression, separately for amplification and deletion outputs.

### GSEA of WGS data

**Extended Data Fig. 7:**
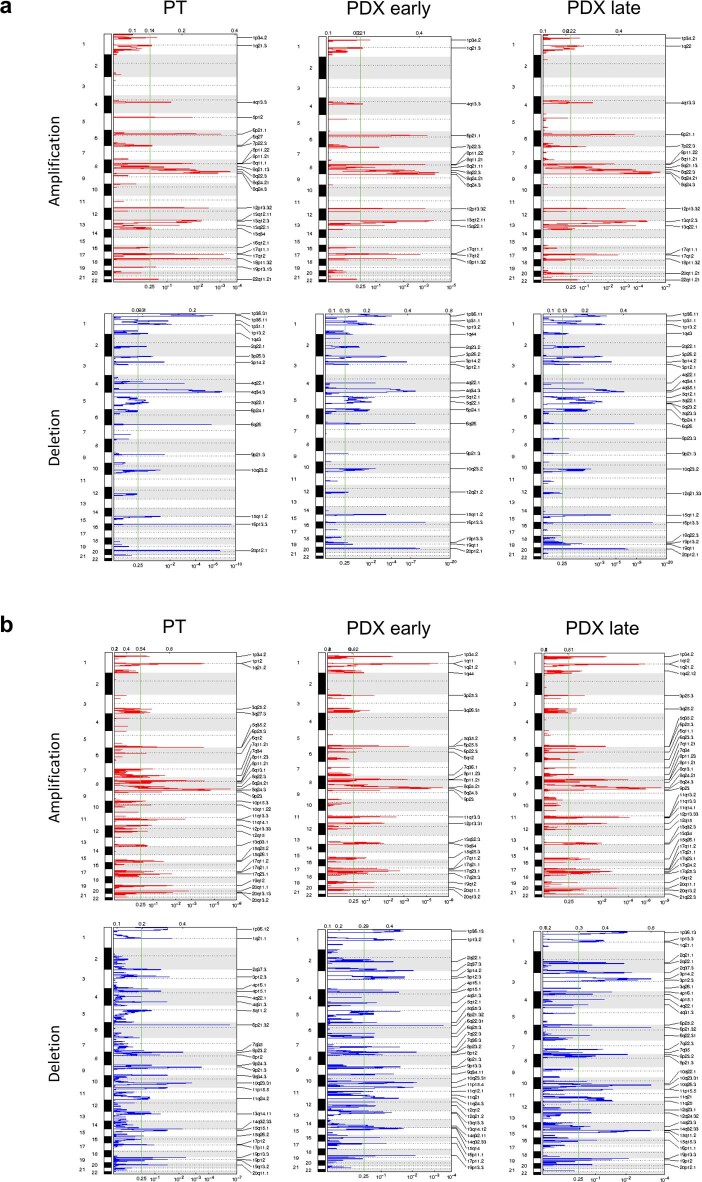
GISTIC analysis of recurrent CNAs. **a**,**b**, GISTIC plots showing amplified and deleted regions in the EurOPDX WGS of trios of PTs and derived PDXs, at early and late passages, of colorectal cancer (**a**, 87 trios) and breast cancer (**b**, 43 trios). For each GISTIC plot, the top axis reports the G-score and the bottom axis the *q*-value.

**Extended Data Fig. 8:**
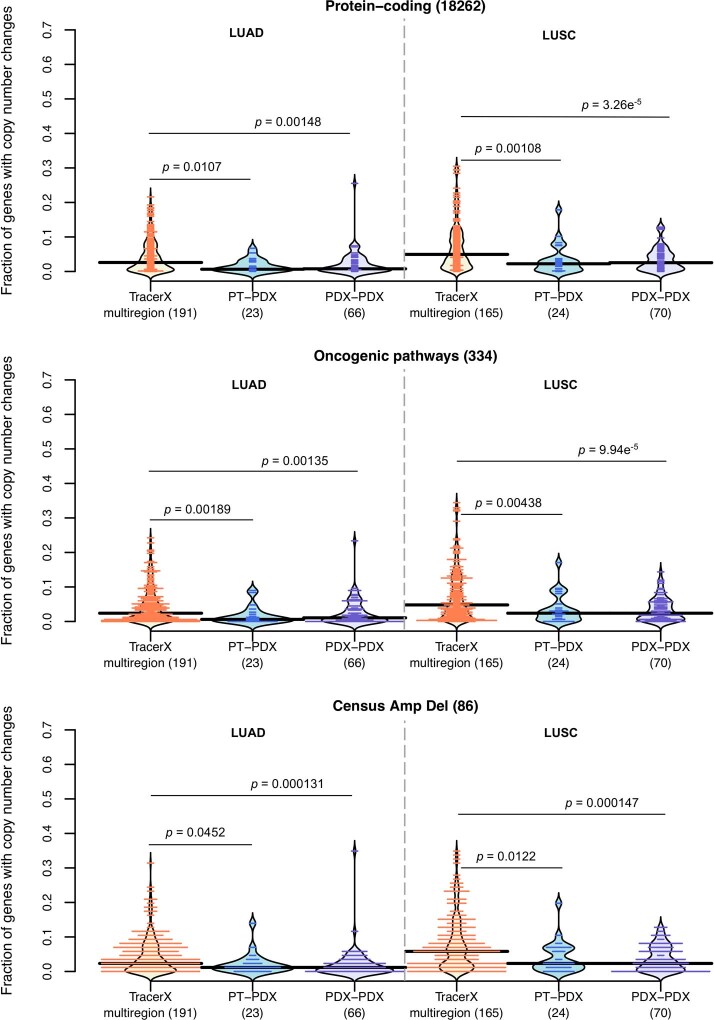
Distribution of proportion of altered genes for lung cancer samples. Comparison between multi-region tumor pairs from TRACERx, and PT-PDX and PDX-PDX pairs for various gene sets for LUAD and LUSC. Gene sets and CNA thresholds are the same as Fig. 4, other gene sets are shown in Fig. 6b. *P*-values were computed by one-sided Wilcoxon rank sum test. Numbers of genes per gene set are indicated in the plot title, and number of pairwise comparisons are indicated in the horizontal axis labels.

To assess the biological functions associated with the recurrent alterations detected by the GISTIC analysis, we performed GSEAPreranked analysis^52,53^ (GSEA version 3.0) on gene-level *G* score profiles for both amplifications and deletions. In particular, we applied the algorithm with 1,000 permutations on various gene set collections from the Molecular Signatures Database^89,90^ (MSigDB version 6.2): (1) hallmark; (2) curated (chemical and genetic perturbations and canonical pathways); (3) Gene Ontology (biological processes, molecular functions and cellular components); and (4) oncogenic signatures. These collections were composed of 50, 4,762, 5,917 and 189 gene sets, respectively. We also included gene sets with known cancer- or treatment-related functions, as described above. We noted that multiple genes with contiguous chromosomal locations—typically in recurrent amplicons—generated spurious enrichment for gene sets consisting of multiple genes of adjacent positions, while very few or none of them had a significant *G* score. To avoid this confounding issue, we only considered the leading-edge genes (that is, those genes with an increasing NES up to its maximum value that contribute to the GSEA significance for a given gene set). The leading-edge subset can be interpreted as the core that accounts for the gene set’s enrichment signal (http://software.broadinstitute.org/gsea). We included a requirement that the leading-edge genes passing the *G* score significance thresholds based on a GISTIC *q* value of 0.25 (Supplementary Table 8 and Extended Data Fig. 7) make up at least 20% of the gene set. This 20% threshold was chosen as the minimum threshold at which gene sets assembled from TCGA-generated lists of genes with recurrent CNAs in CRC or BRCA were identified as significant in GSEA (see Supplementary Table 9). Finally, gene sets with a NES of >1.5 and a false discovery rate *q* value of <0.05 that passed the leading-edge criteria were considered significantly enriched in genes affected by recurrent CNAs.

### Ethics

All of the xenograft studies were completed in accordance with animal research ethics regulations. For details, see the Supplementary Methods.

### Reporting Summary

Further information on research design is available in the Nature Research Reporting Summary linked to this article.

## Online content

Any methods, additional references, Nature Research reporting summaries, source data, extended data, supplementary information, acknowledgements, peer review information; details of author contributions and competing interests; and statements of data and code availability are available at 10.1038/s41588-020-00750-6.

## Supplementary information

Supplementary Information Supplementary Notes 1–17, Methods, Figs. 1–71, Tables 1–4 and 10 and References

Reporting Summary

Supplementary Tables Supplementary Tables 5–9

Supplementary Data 1 Supplementary Data 1

## Data Availability

Copy number calls from all datasets are available in Supplementary Data 1, and these were used for all of the figures. Raw sequence data for these calls are a combination of previously described sources (notably, the publicly available NCI Patient-Derived Models Repository; pdmr.cancer.gov) and newly sequenced data. New sequence data from PDXNet are being shared as part of the NCI Cancer Moonshot initiative through the Cancer Data Service. For further details, contact the corresponding authors. The SNP array data generated by The Jackson Laboratory can be requested via the Mouse Models of Human Cancer Database (tumor.informatics.jax.org). The WGS data generated by EurOPDX can be made available by directly contacting the EurOPDX Consortium (dataportal.europdx.eu or e-mail to E. Medico). Other publicly available data used in the analyses include those deposited to the Gene Expression Omnibus (GSE90653, GSE3526 and GSE33006) and ArrayExpress (E-MTAB-1503-3), as well as CCLE cell line genomic and drug response data (Cancer Therapeutics Response Portal version 2), and MSigDB version 6.2 and TRACERx non-small cell lung cancer data (10.1056/NEJMoa1616288).

## Code availability

We have used well-established computational sequence analysis and statistical analysis techniques, so no code is provided. Full descriptions of all of the analysis techniques are provided in the Methods. The implementation of the copy number estimation workflow from WES data is deployed in the cancer genomics cloud at SevenBridges (https://cgc.sbgenomics.com/public/apps#pdxnet/pdx-wf-commit2/wes-cnv-tumor-normal-workflow/ and https://cgc.sbgenomics.com/public/apps#pdxnet/pdx-wf-commit2/pdx-wes-cnv-xenome-tumor-normal-workflow/).
