# Author Correction: Conservation of copy number profiles during engraftment and passaging of patient-derived cancer xenografts

**DOI:** 10.1038/s41588-021-00811-4

**Published:** 2021-02-19

**Authors:** Xing Yi Woo, Jessica Giordano, Anuj Srivastava, Zi-Ming Zhao, Michael W. Lloyd, Roebi de Bruijn, Yun-Suhk Suh, Rajesh Patidar, Li Chen, Sandra Scherer, Matthew H. Bailey, Chieh-Hsiang Yang, Emilio Cortes-Sanchez, Yuanxin Xi, Jing Wang, Jayamanna Wickramasinghe, Andrew V. Kossenkov, Vito W. Rebecca, Hua Sun, R. Jay Mashl, Sherri R. Davies, Ryan Jeon, Christian Frech, Jelena Randjelovic, Jacqueline Rosains, Francesco Galimi, Andrea Bertotti, Adam Lafferty, Alice C. O’Farrell, Elodie Modave, Diether Lambrechts, Petra ter Brugge, Violeta Serra, Elisabetta Marangoni, Rania El Botty, Hyunsoo Kim, Jong-Il Kim, Han-Kwang Yang, Charles Lee, Dennis A. Dean, Brandi Davis-Dusenbery, Yvonne A. Evrard, James H. Doroshow, Alana L. Welm, Bryan E. Welm, Michael T. Lewis, Bingliang Fang, Jack A. Roth, Funda Meric-Bernstam, Meenhard Herlyn, Michael A. Davies, Li Ding, Shunqiang Li, Ramaswamy Govindan, Claudio Isella, Jeffrey A. Moscow, Livio Trusolino, Annette T. Byrne, Jos Jonkers, Carol J. Bult, Enzo Medico, Jeffrey H. Chuang, PDXNET Consortium, Matthew H. Bailey, Vito W. Rebecca, Michael A. Davies, Peter N. Robinson, Brian J. Sanderson, Steven B. Neuhauser, Lacey E. Dobrolecki, Xiaofeng Zheng, Mourad Majidi, Ran Zhang, Xiaoshan Zhang, Argun Akcakanat, Kurt W. Evans, Timothy A. Yap, Dali Li, Erkan Yucan, Christopher D. Lanier, Turcin Saridogan, Bryce P. Kirby, Min Jin Ha, Huiqin Chen, Scott Kopetz, David G. Menter, Jianhua Zhang, Shannon N. Westin, Michael P. Kim, Bingbing Dai, Don L. Gibbons, Coya Tapia, Vanessa B. Jensen, Gao Boning, John D. Minna, Hyunsil Park, Brenda C. Timmons, Luc Girard, Dylan Fingerman, Qin Liu, Rajasekharan Somasundaram, Min Xiao, Vashisht G. Yennu-Nanda, Michael T. Tetzlaff, Xiaowei Xu, Katherine L. Nathanson, Song Cao, Feng Chen, John F. DiPersio, Kian H. Lim, Cynthia X. Ma, Fernanda M. Rodriguez, Brian A. Van Tine, Andrea Wang-Gillam, Michael C. Wendl, Yige Wu, Matthew A. Wyczalkowski, Lijun Yao, Reyka Jayasinghe, Rebecca L. Aft, Ryan C. Fields, Jingqin Luo, Katherine C. Fuh, Vicki Chin, John DiGiovanna, Jeffrey Grover, Soner Koc, Sara Seepo, Tiffany Wallace, Chong-Xian Pan, Moon S. Chen, Luis G. Carvajal-Carmona, Amanda R. Kirane, May Cho, David R. Gandara, Jonathan W. Riess, Tiffany Le, Ralph W. deVere White, Clifford G. Tepper, Hongyong Zhang, Nicole B. Coggins, Paul Lott, Ana Estrada, Ted Toal, Alexa Morales Arana, Guadalupe Polanco-Echeverry, Sienna Rocha, Ai-Hong Ma, Nicholas Mitsiades, Salma Kaochar, Bert W. O’Malley, Matthew J. Ellis, Susan G. Hilsenbeck, Michael Ittmann, EurOPDX Consortium, Roebi de Bruijn, Petra ter Brugge, Simona Corso, Alessandro Fiori, Silvia Giordano, Marieke van de Ven, Daniel S. Peeper, Ian Miller, Cristina Bernadó, Beatriz Morancho, Lorena Ramírez, Joaquín Arribas, Héctor G. Palmer, Alejandro Piris-Gimenez, Laura Soucek, Ahmed Dahmani, Elodie Montaudon, Fariba Nemati, Virginie Dangles-Marie, Didier Decaudin, Sergio Roman-Roman, Denis G. Alférez, Katherine Spence, Robert B. Clarke, Mohamed Bentires-Alj, David K. Chang, Andrew V. Biankin, Alejandra Bruna, Martin O’Reilly, Carlos Caldas, Oriol Casanovas, Eva Gonzalez-Suarez, Purificacíon Muñoz, Alberto Villanueva, Nathalie Conte, Jeremy Mason, Ross Thorne, Terrence F. Meehan, Helen Parkinson, Zdenka Dudova, Ales Křenek, Dalibor Stuchlík, Olivier Elemento, Giorgio Inghirami, Anna Golebiewska, Simone P. Niclou, G. Bea A. Wisman, Steven de Jong, Petra Kralova, Radislav Sedlacek, Elisa Claeys, Eleonora Leucci, Massimiliano Borsani, Luisa Lanfrancone, Pier Giuseppe Pelicci, Gunhild Mari Mælandsmo, Jens Henrik Norum, Emilie Vinolo

**Affiliations:** 1 The Jackson Laboratory for Genomic Medicine, Farmington, CT USA; 2 Department of Oncology, University of Turin, Turin, Italy; 3 Candiolo Cancer Institute, FPO-IRCCS, Turin, Italy; 4 The Jackson Laboratory for Mammalian Genetics, Bar Harbor, ME USA; 5 Netherlands Cancer Institute, Amsterdam, the Netherlands; 6 College of Medicine, Seoul National University, Seoul, Republic of Korea; 7 Frederick National Laboratory for Cancer Research, Frederick, MD USA; 8 Department of Oncological Sciences, Huntsman Cancer Institute, University of Utah, Salt Lake City, UT USA; 9 Department of Human Genetics, University of Utah, Salt Lake City, UT USA; 10 Department of Bioinformatics and Computational Biology, The University of Texas MD Anderson Cancer Center, Houston, TX USA; 11 The Wistar Institute, Philadelphia, PA USA; 12 Department of Medicine, Washington University School of Medicine in St. Louis, St. Louis, MO USA; 13 Seven Bridges Genomics, Charlestown, MA USA; 14 Department of Physiology and Medical Physics, Centre for Systems Medicine, Royal College of Surgeons in Ireland, Dublin, Ireland; 15 Center for Cancer Biology, VIB, Leuven, Belgium; 16 Laboratory of Translational Genetics, Department of Human Genetics, KU Leuven, Leuven, Belgium; 17 Vall d´Hebron Institute of Oncology, Barcelona, Spain; 18 Department of Translational Research, Institut Curie, PSL Research University, Paris, France; 19 Precision Medicine Center, The First Affiliated Hospital of Xi’an Jiaotong University, Xi’an, People’s Republic of China; 20 Department of Life Sciences, Ewha Womans University, Seoul, Republic of Korea; 21 Division of Cancer Treatment and Diagnosis, National Cancer Institute, Bethesda, MD USA; 22 Department of Surgery, Huntsman Cancer Institute, University of Utah, Salt Lake City, UT USA; 23 Lester and Sue Smith Breast Center, Baylor College of Medicine, Houston, TX USA; 24 Department of Thoracic and Cardiovascular Surgery, The University of Texas MD Anderson Cancer Center, Houston, TX USA; 25 Department of Investigational Cancer Therapeutics, The University of Texas MD Anderson Cancer Center, Houston, TX USA; 26 Department of Melanoma Medical Oncology, The University of Texas MD Anderson Cancer Center, Houston, TX USA; 27 Investigational Drug Branch, National Cancer Institute, Bethesda, MD USA; 28 Department of Biostatistics, The University of Texas MD Anderson Cancer Center, Houston, TX USA; 29 Department of Gastrointestinal Medical Oncology, The University of Texas MD Anderson Cancer Center, Houston, TX USA; 30 Department of Genomic Medicine, The University of Texas MD Anderson Cancer Center, Houston, TX USA; 31 Department of Gynecologic Oncology and Reproductive Medicine, The University of Texas MD Anderson Cancer Center, Houston, TX USA; 32 Department of Surgical Oncology, The University of Texas MD Anderson Cancer Center, Houston, TX USA; 33 Department of Thoracic/Head and Neck Medical Oncology, The University of Texas MD Anderson Cancer Center, Houston, TX USA; 34 Department of Translational Molecular Pathology, The University of Texas MD Anderson Cancer Center, Houston, TX USA; 35 Department of Veterinary Medicine and Surgery, The University of Texas MD Anderson Cancer Center, Houston, TX USA; 36 Hamon Center For Therapeutic Oncology, UT Southwestern Medical Center, Dallas, TX USA; 37 Department of Pathology and Laboratory Medicine, Hospital of the University of Pennsylvania, Philadelphia, PA USA; 38 Abramson Cancer Center, University of Pennsylvania, Philadelphia, PA USA; 39 Department of Surgery, Washington University School of Medicine in St. Louis, St. Louis, MO USA; 40 Division of Gynecologic Oncology, Washington University School of Medicine in St. Louis, St. Louis, MO USA; 41 Center to Reduce Cancer Health Disparities, National Cancer Institute, Bethesda, MD USA; 42 Department of Internal Medicine, Division of Hematology and Oncology, University of California, Davis, Sacramento, CA USA; 43 Department of Biochemistry and Molecular Medicine, University of California, Davis, Sacramento, CA USA; 44 UC Davis Comprehensive Cancer Center, University of California, Davis, Sacramento, CA USA; 45 UC Davis Genome Center, University of California, Davis, Sacramento, CA USA; 46 Department of Medicine, Baylor College of Medicine, Houston, TX USA; 47 Department of Molecular and Cellular Biology, Baylor College of Medicine, Houston, TX USA; 48 Department of Pathology, Baylor College of Medicine, Houston, TX USA; 49 Manchester Breast Centre, Division of Cancer Sciences, University of Manchester, Manchester, UK; 50 University Hospital of Basel, University of Basel, Basel, Switzerland; 51 Institute of Cancer Sciences, University of Glasgow, Glasgow, UK; 52 Cancer Research UK Cambridge Institute, Cambridge Cancer Centre, Cambridge, UK; 53 Catalan Institute of Oncology, L’Hospitalet de Llobregat, Barcelona, Spain; 54 European Bioinformatics Institute, European Molecular Biology Laboratory, Wellcome Genome Campus, Hinxton, UK; 55 Institute of Computer Science, Masaryk University, Brno, Czech Republic; 56 Weill Cornell Medical College, Cornell University, New York, NY USA; 57 NorLux Neuro-Oncology Laboratory, Department of Oncology, Luxembourg Institute of Health, Luxembourg, Luxembourg; 58 University Medical Centre Groningen, Groningen, the Netherlands; 59 Czech Center for Phenogenomics, Institute of Molecular Genetics, Prague, Czech Republic; 60 TRACE PDX Platform, Katholieke Universiteit Leuven, Leuven, Belgium; 61 European Institute of Oncology, Milan, Italy; 62 Oslo University Hospital, Oslo, Norway; 63 Seeding Science SPRL, Limelette, Belgium

**Keywords:** Cancer, Computational biology and bioinformatics

Correction to: *Nature Genetics* 10.1038/s41588-020-00750-6, published online 7 January 2021.

This paper was originally published without open access. As of the date of this correction, the paper is available online as an open-access paper under a Creative Commons Attribution 4.0 International License.

