# Supplementary material for: Conservation of copy number profiles during engraftment and passaging of patient-derived cancer xenografts
Source: Nat Genet. 2021 Jan 7;53(1):86–99. doi: 10.1038/s41588-020-00750-6 (PMC7808565; doi:10.1038/s41588-020-00750-6)
Supplement: Supplementary file 1 — Supplementary Notes 1–17, Methods, Figs. 1–71, Tables 1–4 and 10 and References [file 41588_2020_750_MOESM1_ESM.pdf]

nature portfolio

https://doi.org/10.1038/s41588-020-00750-6

Supplementary information

Conservation of copy number profiles during engraftment and passaging of patient-derived cancer xenografts

In the format provided by the authors and unedited

nature research

https://doi.org/10.1038/s41588-020-00750-6

Supplementary information

Conservation of copy number profiles during engraftment and passaging of patient-derived cancer xenografts

In the format provided by the authors and unedited

SUPPLEMENTARY INFORMATION

Conservation of copy number alteration profiles during engraftment and passaging of patient-derived cancer xenografts

CONTENTS

Supplementary notes

Supplementary methods

Supplementary figures

Supplementary tables

Supplementary references

SUPPLEMENTARY NOTES

Supplementary Note 1: Copy number profiles of PDXs

The copy number profiles of the PDXs and PTs represented a variety of copy number profiles (Supplementary Fig. 24 – 60). We also profiled a small set of breast cancer patient tumors that failed engraftment and observed no significant difference in the extent of altered genome between tumors that were successfully engrafted into the mice and failed engraftment, indicating that selection of certain levels of genomic instability due to biases in successful PDX engraftment is unlikely (Supplementary Fig. 61).

Supplementary Note 2: Comparison of SNP array and WES copy number data

A larger discordance is more likely for CNA profiles dominated by large number of small focal events, for example in the tandem duplicator and chromothripsis phenotypes[1,2]. Evolutionary behaviors of focal mutations may be of particular interest in cancer types such as pancreas adenocarcinoma, for which chromothripsis has been reported to have a high[3]. However, our WES data for pancreas adenocarcinoma and SNP data for patient tumors did not show chromothripsis. This may be a limitation of the copy number data, as genomic rearrangements from WGS data is superior for detecting chromothripsis[4]. Future WGS studies may be useful for clarifying such questions.

Supplementary Note 3: Comparison of expression-based copy number calls calibrated against normal and tumor samples

A notable problem with the expression-based calls is that the alternative expression calibrations can have a major impact on called gains and losses. This is especially apparent for regions frequently called as gains or losses in specific tumor types (Supplementary Fig. 20), e.g. as identified by GISTIC analysis in other studies[5-7]. Chromosomes 8q and 13 were almost exclusively identified as gains and chromosomes 21 and 22 were almost exclusively as losses in the gastric cancer RNA-Seq dataset when normal samples were used for calibration. Similarly, we called exclusive gains in chromosomes 7q and 20 and losses in chromosomes 4q31-35, 8p,16q and 21 using normal samples for calibration for the hepatocellular carcinoma expression array dataset. However, changing the calibration to use tumor samples resulted in these regions being erroneously called with approximately equal frequencies of gains and losses.

Supplementary Note 4: Copy number correlation of PT-PDX and PDX-PDX pairs

The concordance analysis of PT-PDX pairs quantifies the extent of CNA retention in PDXs relative to their originating PTs, including during initial tumor engraftment. On the other hand, concordance analysis of PDX-PDX pairs evaluates copy number evolution during PDX expansion and passaging.

Supplementary Note 5: Copy number correlation using RNA-based approaches

We also performed intra-model comparisons using RNA-based approaches, and observed that the tumor-median normalization for expression data results in lower concordance between the sample pairs compared to normal-median normalization (p = 6.79e^{-6} for RNASEQ, p = 0.00170 for EXPARR) (Supplementary Fig. 63a and b). Furthermore, the Pearson correlations between pairs of samples using expression data did not reproduce the Pearson correlations from SNP array platforms for those same sample pairs (p = 0.0136 for EXPARR NORM, p = 0.000976 for EXPARR TUM) (Supplementary Fig. 63c). This confirms that the comparison of CNA profiles between PT and PDXs based on expression data can lead to the overestimation of copy number changes during engraftment and passage, which is caused by the low accuracy of expression-based copy number estimation coupled with potentially problematic normalization.

Supplementary Note 6: Copy number correlations for specific tumor types

Additionally, we repeated the same analysis using individual tumor types with ≥10 models having multiple passages including PT. We observed no tumor type-specific behaviors different from the pan-cancer analysis (Supplementary Fig. 64).

Supplementary Note 7: Copy number correlations of PDX samples with same passage number.

We observed very similar median (same/different passages: 0.962/0.966 for SNP, 0.940/0.932 for WES) and interquartile range (IQR) (same/different passages: 0.0700/0.0619 for SNP and 0.103/0.0979 for WES) of correlation coefficients between same-passage (i.e. all P0s, all P1s, all P2s, etc) and different-passage PDX-PDX pairs (two-sided Wilcoxon rank sum test: p > 0.1 for SNP and WES; Fig. 3d – f).

Supplementary Note 8: Examples of models exhibiting large copy number drift between lineages

A few examples of models exhibiting large drift between lineages include TM01500 (Supplementary Fig. 26); 416634, 558786 and 665939 (Supplementary Fig. 47); 135848 and 762968 (Supplementary Fig. 48); 245127 and 959717 (Supplementary Fig. 49); 287954 and 594176 (Supplementary Fig. 56); 174316 and 695221 (Supplementary Fig. 54).

Supplementary Note 9: Effect of phylogenetic distance on copy number correlation

We asked if the phylogenetic distance between samples could explain the observed shifts in the correlations. These distance relationships are clearest for the CRC and BRCA WGS sets because these models have only one lineage split occurring at the engraftment stage. We compared correlation as a function of phylogenetic distance within a lineage, which in this phylogeny is simply equal to the difference in passage number between the two samples. Increase in passage difference did not consistently reduce the correlation between samples (Supplementary Fig. 65).

Supplementary Note 10: Mutations in genome stability-related genes do not predict PDX copy number stability.

We further explored if the stability of copy number during engraftment and passaging is affected by mutations in genes known to impact genome stability. Hence, we compared the within-model copy number correlations in models with wildtype vs. mutated *TP53* or *BRCA1/2* (see METHODS). We did not observe any increase in copy number variability (i.e. decrease in correlation) associated with the mutational status of *TP53* or *BRCA1/2* that was consistent across platforms, though a very small but significant decrease in correlation was observed for WES models with mutated *TP53* or *BRCA1/2* and for SNP models with mutated *BRCA1/2* (Supplementary Fig. 66a – f). Similarly, we did not observe any significant decrease in correlation associated with having mutations in DNA repair genes[8,9] (Supplementary Fig. 66g). Overall, this indicates that presence of mutations in such genes does not lead to increased copy number changes during PDX engraftment and passaging Interestingly, for the small set of pairs in which the two samples have discordant mutation status, copy number concordance is also lower between the samples. This suggests that tumor purity and/or heterogeneity have an impact on estimates of copy number evolution, consistent with our other findings.

Supplementary Note 11: *TP53* and *BRCA1/2* mutations in breast cancer models

We also observed that breast cancer models display more copy number changes compared to other tumor types (Fig. 4b). We observed no significant decrease in the copy number correlation among the breast cancer models carrying *TP53* or *BRCA1/2* mutations (Supplementary Fig. 67). In particular, among the PDX models with the largest copy number changes (correlation < 0.6), all (n=4) lack both *TP53* or *BRCA1/2* mutations. This indicates that there is no association between copy number instability in breast cancer PDXs and mutations in *TP53* or *BRCA1/2* genes.

Supplementary Note 12: Recurrently altered genes in PDXs

We observed that all these recurrent genes overlapped models in which one sample displayed an unusually large gain or loss ($|\log2 \text{(CN ratio)}| > 1.5$). This suggests that these regions may be subject to more noise in the CNA estimation procedure at these loci (Supplementary Fig. 68).

We further queried from CCLE data whether any of these recurrent genes showed evidence for copy number-related drug response (see METHODS, Supplementary Table 5). For the 6 genes with sufficient data available, we found no association between copy number and drug response mediated by gene expression (*q-value* < 1).

Supplementary Note 13: GISTIC analysis for WGS dataset

We observed that the GISTIC profiles of both CRC and BRCA (Extended Data Fig. 7) were similar to the respective TCGA BRCA and COADREAD GISTIC 2.0 plots[10,11].

In the gene-level G-score analysis, not a single gene had both ΔG concordant and passing the respective GISTIC threshold for significance (see Supplementary Table 8). It should be noted that very small segments of recurrent copy number gain or loss could be missed by this analysis due to the bin size imposed by the WGS coverage.

Supplementary Note 14: GSEA analysis for WGS dataset

Consistent with the known recurrence of cancer CNAs at driver genes, multiple gene sets displayed significant enrichment in individual cohorts. To avoid spurious apparent enrichment for sets of genes with adjacent chromosomal location, we implemented an additional filter based on G-score significance (see METHODS and Supplementary Table 8). After applying the Normalized Enrichment Score (NES), FDR q-value and G-score filters, 49 gene sets were found to be significant in at least one of the three CRC cohorts, and 89 gene sets in at least one of the three BRCA cohorts (Supplementary Table 9). Importantly, control gene sets composed of GISTIC hits identified in TCGA CRC and BRCA datasets were all significant, confirming that the WGS cohorts used here correctly recapitulate the major CNA features of these two cancer types.

Supplementary Note 15: Preference of DNA-based CN measurements

The conclusion DNA-based copy number measurements being superior to RNA-based inferences applies to all copy number analysis of tumors, and is not specific to xenografts. Amongst the DNA-based approaches for copy number analysis, SNP arrays still have a competitive edge because they have the highest resolution, despite the non-uniform distribution of probes along the genome[12,13]. Robust algorithms are available for SNP arrays to deconvolute tumor purity and B-allele frequency, allowing correct estimation of allele-specific copy number profiles despite the presence of normal DNA contamination[14]. Our analysis has shown that there are significant variations in purity that affect copy number inferences among the patient tumor samples that are more difficult to resolve with other platforms.

Supplementary Note 16: Impact of subclonality on copy number correlation

The CNA profiles observed in this study are weighted contributions summed over all clonal fractions, including human stroma. Individual samples can also contain diverse subclones (see Extended Data Fig. 6a), though resolution of subclones and their copy number profiles across passages is an underdetermined problem that remains algorithmically challenging[15].

Supplementary Note 17: Models with large copy number discordance

The extreme cases of copy number discordance within models (see Supplementary Fig. 70 for examples with same lineage) may be informative for future studies of the evolutionary process, especially through consideration of repeated spatial sampling. It may be informative to compare such examples to those reported by Eirew et al.[16], who described a variety of clonal selection dynamics during engraftment and passaging for breast cancer PDXs, as well as by Ding et al.[17], who demonstrated the possibility of cellular selection during xenograft formation similar to that during metastasis. While such cases are uncommon in our study, further subclonal analysis may be useful for clarifying potential selection pressures.

SUPPLEMENTARY METHODS

Experimental details for sample collection, PDX engraftment and passaging, and array or sequencing

The tumor types and patient tumor (PT) and patient derived xenograft (PDX) samples contributed by various centers are summarized in Supplementary Fig. 1-12 and Supplementary Table 1. The sample collection, PDX engraftment and passaging, and array and sequencing methodologies by the various centers are described below. The xenograft initial take rates at each contributing center are summarized in Supplementary Table 10.

The Jackson Laboratory (JAX). Patient tumor engraftment and PDX passaging of various tumor types were performed as previously described[18-20]. Detailed information of the PDX models can be found in the PDX model search form in Mouse Tumor Biology Database (MTB, http://tumor.informatics.jax.org/mtbwi/pdxSearch.do). SNP array samples were genotyped with the Affymetrix Genome-Wide Human SNP Array 6.0 as described in Woo et al[20]. Whole-exome sequencing were processed as follows: DNA was isolated from tumor and blood samples using the Wizard Genomic DNA Purification Kit (Promega) according to the manufacturer's protocols. DNA quality was assessed using an E-Gel General Purpose Agarose Gel, 0.8% (Invitrogen) and Nanodrop 2000 spectrophotometer (Thermo Scientific). DNA concentration was determined using a Qubit dsDNA BR Assay Kit (Thermo Scientific). Libraries were prepared by the Genome Technologies core facility at The Jackson Laboratory using SureSelectXT Reagents and SureSelectXT Human All Exon V4 Target Enrichment System (Agilent Technologies), according to the manufacturer's instructions. Briefly, the protocol entails shearing the DNA using the Covaris E220 Focused-ultrasonicator (Covaris), ligating Illumina specific adapters, and PCR amplification. Amplified DNA libraries are then hybridized to the Human All Exon probes, amplified using indexed primers, and checked for quality and concentration using the DNA High-Sensitivity LabChip assay (Agilent Technologies) and quantitative PCR (KAPA Biosystems), according to the manufacturers' instructions. Libraries were sequenced on a HiSeq 2500 100bp paired-end flow cell using TruSeq Rapid SBS reagents (Illumina). Average coverage for normal samples was 154.38x (115.13 min – 212.31 max), and was 232.10x for tumor samples (161.48 min – 280.65 max).

Seoul National University-Jackson Laboratory (SNU-JAX). Gastric cancer tissues, paired normal gastric tissues, and blood samples were obtained from individuals who underwent gastrectomies at the Hospital of Seoul National University from 2014 to 2016. All samples were obtained with informed consent at the Hospital of Seoul National University, and the institutional

review board approved the study per the Declaration of Helsinki. These samples were stored into RPMI media with 1% penicillin/streptomycin immediately after resected from patients and shipped using specimen ice box to the laboratory within half an hour. Gastric cancer samples were divided into several small pieces (2mm × 2mm) and used to generate PDX models and for genomic analysis. Mice were cared for according to institutional guidelines of the Institutional Animal Care and Use Committee of the Seoul National University (no. 14-0016-C0A0). For PDX models, surgically resected tissues were minced into pieces approximately ~2 mm in size and injected into the subcutaneous area in the flanks of 6-week-old NOD/SCID/IL-2γ-receptor null female mice (NSG™ mice, Jackson Laboratory, Bar Harbor, ME). The volume of tumors and body weight of mice were checked once or twice a week. The volume was calculated as (tumor length x tumor width^2) / 2. When a tumor reached >700~1000 mm^3, the mouse was sacrificed, and tumor tissues were stored. Tumor tissues were divided and stored for several purposes: (1) Tumor tissues were cryopreserved in liquid nitrogen and stored at −80 °C for generating next passage PDXs. (2) Tumor tissues were frozen in liquid nitrogen for genomic analysis. Whole-exome sequencing was conducted as follows: Genomic DNA (gDNA) was extracted from blood and tissues using DNeasy blood and tissue kit (QIAGEN) and checked for purity, concentration, and integrity by OD260/280 ratio using NanoDrop Instruments (NanoDrop Technologies, Wilmington, DE, USA) and agarose gel electrophoresis. DNA was sheared by fragmentation by Bioruptor (Diagenode, Inc., Denville, NJ, USA) and purified using Agencourt AMPure XP beads (Beckman Coulter, Fullerton, CA, USA). DNA samples were then tested for size distribution and concentration using an Agilent Bioanalyzer 2100. Standard protocols were utilized for adaptor ligation, indexing, high-fidelity PCR amplification. Subsequently, exome enrichment was performed by hybrid capture with the All Exon v5 capture library. Capture libraries were amplified, pooled, and submitted to the commercial sequencing company (Macrogen) for 100bp paired-end, multiplex sequencing on a HiSeq 2000 sequencing system. Average coverage for normal samples was 62.67x (38.97 min – 108.77 max), and was 102.35x for tumor samples (36.02 min – 150.49 max). RNA-Sequencing data was generated as follows: RNA was extracted from tissues using the RNeasy Mini Kit (Qiagen, Valencia, CA, USA). RNA-Sequencing libraries were prepared from 1 µg total RNA using the TruSeq RNA Sample Preparation v2 Kit (Illumina, San Diego, CA) according to the manufacturer’s protocol. Libraries were submitted to the commercial sequencing company (Macrogen) for 100bp paired-end, multiplex sequencing on a HiSeq 2000 sequencer.

Huntsman Cancer Institute (HCI). Patient tumor engraftment and PDX passaging of breast cancer samples were performed as previously described[21,22]. SNP array samples were genotyped by the Affymetrix SNP 6.0 array for profiling. These samples were processed, according to

DeRose et. al.[22]. Additionally, some samples, were also processed using the Illumina Infinium Omni 2.5 Exome-8 v1.3 Beadchip array. Hybridized arrays were scanned using an Illumina iScan instrument following the Illumina Infinium LCG Assay Manual Protocol and processed using GenomeStudio. When samples had both Affymetrix and Illumina chips, we deferred to Illumina intensity values for copy number calling. Whole-exome sequencing was conducted as follows: Agilent SureSelectXT Human All Exon V6+COSMIC or Agilent Human All Exon 50Mb library preparation protocols were used with inputs of 100-3000ng sheared genomic DNA (Covaris). Library construction was performed using the Agilent Technologies SureSelectXT Reagent Kit. The concentration of the amplified library was measured using a Qubit dsDNA HS Assay Kit (ThermoFisher Scientific). Amplified libraries (750 ng) were enriched for exonic regions using either the Agilent Technologies SureSelectXT Human All Exon v6+COSMIC or Agilent Human All Exon 50Mb kits and PCR amplified. Enriched libraries were qualified on an Agilent Technologies 2200 TapeStation using a High Sensitivity D1000 ScreenTape assay and the molarity of adapter-modified molecules was defined by quantitative PCR using the Kapa Biosystems Kapa Library Quant Kit. The molarity of individual libraries was normalized to 5 nM, and equal volumes were pooled in preparation for Illumina sequence analysis. Sequencing libraries (25 pM) were chemically denatured and applied to an Illumina HiSeq v4 paired-end flow cell using an Illumina cBot. Hybridized molecules were clonally amplified and annealed to sequencing primers with reagents from an Illumina HiSeq PE Cluster Kit v4-cBot (PE-401-4001). Following the transfer of the flowcell to an Illumina HiSeq 2500 instrument (HCS v2.2.38 and RTA v1.18.61), a 125-cycle paired-end sequence run was performed using HiSeq SBS Kit v4 sequencing reagents (FC-401-4003). Average coverage for normal samples was 90.22x (15.28 min – 131.69 max), and was 96.66x for tumor samples (10.65 min – 166.06 max).

Baylor College of Medicine (BCM). Patient tumor engraftment and PDX passaging of breast cancer samples were performed as previously described[23,24]. SNP array samples were genotyped at Huntsman Cancer Institute using the Illumina Infinium Omni 2.5Exome-8 v1.4 Beadchip array by the procedures provided in the HCI section above.

The University of Texas MD Anderson Cancer Center (MDACC). Fresh non-small-cell lung carcinoma tumor samples were collected from surgically resected specimens with the informed consent of the patients. Generation and passaging of PDXs, and histological analysis and DNA fingerprint assay for PDXs and their primary tumor tissues were performed as previously described[25]. The protocols for the use of clinical specimens and data in this study were approved by the Institutional Review Board at The University of Texas MD Anderson Cancer Center. All animal studies were carried out in accordance with the Guidelines for the Care and Use of

Laboratory Animals (National Institutes of Health Publication 85-23) and the institutional guidelines of MDACC. Whole-exome sequencing was conducted at the Sequencing and Microarray Core Facility at MD Anderson Cancer Center as follows: Genomic DNA was quantified and quality was assessed using Picogreen (Invitrogen) and Genomic DNA Tape for the 2200 Tapestation (Agilent), respectively. DNA from each sample (100-500 ng of genomic DNA) was sheared by sonication and then used for library preparation by using KAPA library preparation kit (KAPA) following manufacturer's instruction. Equimolar amounts of DNA were pooled (2-6 samples per pool) and whole exome regions were captured by using biotin labeled probes from Roche Nimblegen (Exome V3) followed manufacture's protocol. The captured libraries were sequenced on a HiSeq 2000 with 100bp paired-end (Illumina Inc., San Diego, CA, USA) on a paired-end flowcell. Average coverage for normal samples was 85.61x (40.80 min – 228.41 max), and was 125.79x for tumor samples (25.12 min – 251.53 max).

The WISTAR Institute (WISTAR). Tumor biopsy samples were collected according to IRB-approved protocol with the informed written consent of the patients. Collected fresh tumor pieces were snap frozen and stored at -80 °C. Subcutaneous implantation into NSG SCID mice were used to create PDX models. BRAF inhibitor treatment (PLX) was administered as PLX4720 200ppm chemical additive diet chow (Research Diets, New Brunswick, NJ). Whole exome sequencing was conducted as follows: Genome DNA extraction was done using Qiagen DNeasy Blood & Tissue Kit, and libraries for whole exome sequencing were performed using Nextera DNA exome kit. Capture libraries were amplified, pooled, and then sequenced on an Illumina HiSeq 2500 76bp paired-end run. Average coverage for normal samples was 97.50x (71.46 min – 124.64 max), and was 208.27x for tumor samples (146.88 min – 281.20 max).

National Cancer Institute Patient-Derived Models Repository (PDMR). For engraftments, tumor material plus a drop of Matrigel (BD BioSciences, Bedford, MA) were implanted subcutaneously in NSG^{TM} mouse model NOD.Cg-Prkdc^{scid} Il2rg^{tm1Wjl}/SzJ. Mice were housed in sterile, filter-capped polycarbonate cages, maintained in a barrier facility on a 12-hour light/dark cycle, and were provided sterilized food and water, ad libitum. Animals were monitored weekly for tumor growth. The initial passage of material was grown to approximately 1000-2000mm^3 calculated using the following formula: weight (mg) = (tumor length x [tumor width]^2) / 2. Tumor material was then harvested, a portion cryopreserved, and the remainder implanted into NSG host mice. Every PDX tumor harvested and cryopreserved also has 2-3 fragments snap frozen for next generation sequence analysis and short tandem repeat validation and a piece is fixed in neutral buffered formalin and then embedded in paraffin for histological assessment. Related patient data, clinical history, representative histology and short-tandem repeat profiles for the PDX

models can be found at https://pdmr.cancer.gov. Full PDMR standard operating procedures for tumor engraftment and PDX passaging are available at https://pdmr.cancer.gov/sops. Whole-exome sequencing data were generated with the Agilent SureSelect capture kit, and sequenced with 125bp pair-end Illumina HiSeq 2500 runs following standard operating procedures available here: https://pdmr.cancer.gov/sops. Average coverage for normal samples was 148.47x (50.95 min – 242.24 max), and was 174.77x for tumor samples (81.41 min – 403.22 max).

Washington University in St. Louis (WUSTL). All human tissues acquired for these experiments were processed in compliance with NIH regulations and institutional guidelines, approved by the Institutional Review Board at Washington University. Tumors from all patients were obtained via core needle biopsy, skin punch biopsy, or surgical resection after informed consent. All animal procedures were reviewed and approved by the Institutional Animal Care and Use Committee at Washington University in St. Louis. Pancreatic cancer models were derived from tissue fragments implanted subcutaneously into dorsal flank regions of non-humanized, female NOD/SCID/γ mice (Jackson Laboratory, Bar Harbor, ME) using Matrigel. The sample tissues for these PDX models were obtained from archived, cryopreserved PDX harvests. Final tumor passages in mice were kept cold and harvested into RPMI-1640 with antibiotic and antimycotic additives. Pieces of each tumor were processed into the following: flash frozen tissue fragments, OCT blocks and matched Haemotoxylin and Eosin (H&E) slides, formalin fixed paraffin blocks and matched H&E slides, RNAlater tissue storage, and cryopreserved fragments (FBS + 10% DMSO). A minimum of 250 mg of flash frozen material was submitted to the Siteman Cancer Center's Proteomics Core. The tissues were cryo-pulverized and subsequently divided for DNA and RNA preparation, and long-term storage. Patient tumors were obtained directly from operating rooms and placed into sterile collection media (RPMI-1640 with antibiotic and antimycotic additives). Pieces of each tumor were processed into the following: flash frozen tissue fragments, OCT blocks and matched H&E slides, formalin fixed paraffin blocks and matched H&E slides, and cryopreserved fragments (FBS + 10% DMSO). Parental genomic DNA was prepared from OCT blocks if available, and if not available, paraffin blocks were utilized. In addition, genomic DNA for sequencing control was prepped from peripheral blood mononuclear cells that were both procured and processed at time of surgery. Breast cancer models were derived from tissue fragments implanted subcutaneously into dorsal flank regions of non-humanized, NOD/SCID/γ mice (Jackson Laboratories, Bar Harbor, ME) as previously described[17,24]. Whole-exome sequencing was conducted as follows: Libraries were constructed using unamplified genomic DNA (minimum 100 ng) from blood (normal), tumor, and xenograft samples. Exons were captured via IDT Exome library kit followed by high-throughput sequencing on an Illumina

NovaSeq S4 platform (Illumina Inc., San Diego, CA) using 150bp paired-end reads. Details of whole exome library construction have been given elsewhere (Fisher, Barry et al. 2011). Average coverage for normal pancreatic cancer samples was 85.73x (55.65 min – 108.91 max), and was 124.01x (49.68 min – 242.35 max) for tumor pancreatic cancer samples. Average coverage for normal breast cancer samples was 58.33x (45.37 min – 70.30 max), and was 89.90x (17.24 min – 149.53 max) for tumor breast cancer samples.

Shanghai Institute for Biological Sciences (SIBS). Gene expression and copy number data, generated by the Affymetrix Human Genome U133 Plus 2.0 Array and Affymetrix Human SNP 6.0 platforms respectively, of hepatocellular carcinoma (HCC) PDX models were retrieved from the Gene Expression Omnibus (GEO) accession ID GSE90653[26]. Expression microarray data generated by the Affymetrix Human Genome U133 Plus 2.0 Array for normal liver were downloaded from GEO and ArrayExpress: GSE3526[27], GSE33006[28] and E-MTAB-1503-3[29].

EurOPDX colorectal cancer (EuroPDX CRC). Liver-metastatic colorectal cancer samples were obtained from surgical resection of liver metastases at the Candiolo Cancer Institute, the Mauriziano Umberto I Hospital, and the San Giovanni Battista Hospital. Informed consent for research use was obtained from all patients at the enrolling institution before tissue banking, and study approval was obtained from the ethics committees of the three centers. Tissue from hepatic metastasectomy in affected individuals was fragmented and either frozen or prepared for implantation as described previously[30,31]. Non-obese diabetic/severe combined immunodeficient (NOD/SCID) female mice (4–6 weeks old) were used for tumor implantation. Snap-frozen aliquots were obtained from surgical specimens and corresponding tumor grafts at different passages. Whole genome sequencing was conducted as follows: DNA was extracted using Maxwell RSC Blood DNA kit (Promega AS1400) from colorectal cancer liver metastasis and corresponding tumor grafts at different passages. Genomic DNA was fragmented and used for Illumina TruSeq library construction (Illumina) according to the manufacturer's instructions. Libraries were then purified with Qiagen MinElute column purification kit and eluted in 17 µl of 70°C EB to obtain 15 µl of DNA library. The libraries were sequenced on HiSeq4000 (Illumina) with single-end reads of 51bp at low coverage (~0.1x genome coverage on average).

EurOPDX breast cancer (EuroPDX BRCA). Human breast tumors were obtained from surgical resections at the Netherland Cancer Institute (NKI), Institut Curie (IC) and Vall d'Hebron Institute of Oncology (VHIO). Engraftment was conducted with different procedures at each center. NKI: Small tumor fragments (2mm diameter) were implanted into the 4th mammary fat pad of 8-week-old Swiss female nude mice. Mice were checked for tumor appearance once a week, and supplemented with estrogen, if the tumor was ER positive. After palpable tumor detection, tumor

size was measured twice a week. When tumors reached a size of 700-1000 mm^3, animals were sacrificed and tumors were explanted and subdivided in fragments for serial transplantation as described above, or for frozen vital storage in liquid nitrogen. IC: Breast cancer fragments were obtained from patients at the time of surgery, with informed written patient consent. Fragments of 30 to 60 mm^3 were grafted into the interscapular fat pad of 8 to 12-week-old female Swiss nude mice. Mice were supplemented with estrogen. Xenografts appeared at the graft site 2 to 8 months after grafting. When tumors were close to 1500 mm^3, they were subsequently transplanted from mouse to mouse and stocked frozen in DMSO-fetal calf serum (FCS) solution or frozen dried in nitrogen. Fragment fixed tissues in phosphate buffered saline (PBS) 10% formol for histologic studies were also stored. The experimental protocol and animal housing were in accordance with institutional guidelines as proposed by the French Ethics Committee (Agreement B75-05-18, France). VHIO: Fresh tumor samples from patients with breast cancer were collected for implantation following an institutional IRB-approved protocol and the associated informed consent, or by the National Research Ethics Service, Cambridgeshire 2 REC (REC reference number: 08/H0308/178). Experiments were conducted following the European Union's animal care directive (2010/63/EU) and were approved by the Ethical Committee of Animal Experimentation of the Vall d'Hebron Research Institute. Surgical or biopsy specimens from primary tumors or metastatic lesions were immediately implanted in mice. Fragments of 30 to 60 mm^3 were implanted into the mammary fat pad (surgery samples) or the lower flank (metastatic samples) of 6-week-old female athymic HsdCpb:NMRI-Foxn1nu mice (Harlan Laboratories). Animals were continuously supplemented with estradiol. Upon growth of the engrafted tumors, the model was perpetuated by serial transplantation onto the lower flank. Tumor growth was measured with caliper bi-weekly. In all experiments, mouse weight was recorded twice weekly. When tumors reached 1500 mm3, mice were euthanized and tumors were explanted. Whole genome sequencing was conducted as follows: genomic DNA was extracted from breast cancers and corresponding PDXs using (i) QIAamp DNA Mini Kit s(50) (#51304, Qiagen) (IC) or (ii) according to Laird PW's protocol[32] (NKI and VHIO). The amount of double stranded DNA in the genomic DNA samples was quantified by using the Qubit® dsDNA HS Assay Kit (Invitrogen, cat no Q32851). Up to 2000 ng of double stranded genomic DNA were fragmented by Covaris shearing to obtain fragment sizes of 160-180bp. Samples were purified using 1.6X Agencourt AMPure XP PCR Purification beads according to manufacturer's instructions (Beckman Coulter, cat no A63881). The sheared DNA samples were quantified and qualified on a BioAnalyzer system using the DNA7500 assay kit (Agilent Technologies cat no. 5067-1506). With an input of maximum 1 µg sheared DNA, library preparation for Illumina sequencing was performed using the KAPA HTP

Library Preparation Kit (KAPA Biosystems, KK8234). During library enrichment, 4-6 PCR cycles were used to obtain enough yield for sequencing. After library preparation the libraries were cleaned up using 1X AMPure XP beads. All DNA libraries were analyzed on the GX Caliper (a PerkinElmer company) using the HT DNA High Sensitivity LabChip, for determining the molarity. Up to two pools of 24 uniquely indexed samples and one pool of 81 uniquely indexed samples were mixed together by equimolar pooling in a final concentration of 10nM, and subjected to sequencing on an Illlumina HiSeq2500 machine in a total of 12 lanes of a single read 65bp run at low coverage (~0.4x genome coverage on average), according to manufacturer's instructions.

Preprocessing of sequencing and expression array data

Whole-exome sequencing (WES) data. All the samples were subjected to quality control (filtering and trimming of poor-quality reads and bases) using in-house QC script with the cut-off that half of the read length should be ≥20 in base quality at phred scale. We further removed the known adaptors using cut-adapt[33] v1.15 at -m 36. Afterward, we aligned the reads to the human genome (GRCh38.p5) using bwakit[34] v0.7.15. Engrafted tumor samples were subjected to the additional step of mouse read removal using Xenome[35] v1.0.0, with default parameters. The alignment was converted to BAM format using Picard SortSam v2.8.1 (https://broadinstitute.github.io/picard/), and duplicates were removed by Picard MarkDuplicates utility. BaseRecalibrator from the Genome Analysis Tool Kit[36,37] (GATK) v4.0.5.1 was used to adjust the quality of raw reads. Training files for the base quality scale recalibration were Mills_and_1000G_gold_standard.indels.hg38.vcf.gz, Homo_sapiens_assembly38.known_indels.vcf.gz, and dbSNP v151. Mean target coverage was determined for each sample by Picard CollectHsMetrics.

Low-pass whole-genome sequencing (WGS) data. Whole-genome sequence reads from EuroPDX CRC liver metastasis and corresponding tumor grafts at different passages were mapped to the reference human genome (GRCh37) using Burrows-Wheeler Aligner[34] (BWA) v0.7.12. SAMTools[38] v0.1.18 was used to convert SAM files into BAM files and Picard v1.43 to remove PCR duplicates (http://broadinstitute.github.io/picard/). Whole-genome sequence reads from EuroPDX BRCA tumors and corresponding tumor grafts at different passages were mapped to the reference human genome (GRCh38) and mouse genome (GRCm38/mm10, Ensembl 76) using Burrows-Wheeler Aligner (BWA) v0.7.15. Subsequently, mouse reads were excluded with XenofilteR[39]. Other processing steps are similar as described above.

RNA-sequencing (RNA-Seq) and gene expression microarray (EXPARR) data. For SNU-JAX RNA-Seq data, simultaneous read alignment was performed to both mouse (mm10) and human

genome (GRCh38.p5) and only human specific reads were used for the expression quantification. Expression of mRNA was quantified as Transcripts Per Million (TPM) for downstream analysis using RNA-Seq by Expectation Maximization[40] (RSEM v1.3.1) with ensemble GTF reference GRCh38.92. Gene expression microarray data for SIBS HCC and normal liver samples from GEO and ArrayExpress databases were profiled as follows. After initial quality control and outlier removal, CEL files were normalized according to RMA algorithm and probesets were annotated according to Affymetrix annotation file for HG-U133 Plus 2, released on 2016-03-15 build 36.

SUPPLEMENTARY FIGURES

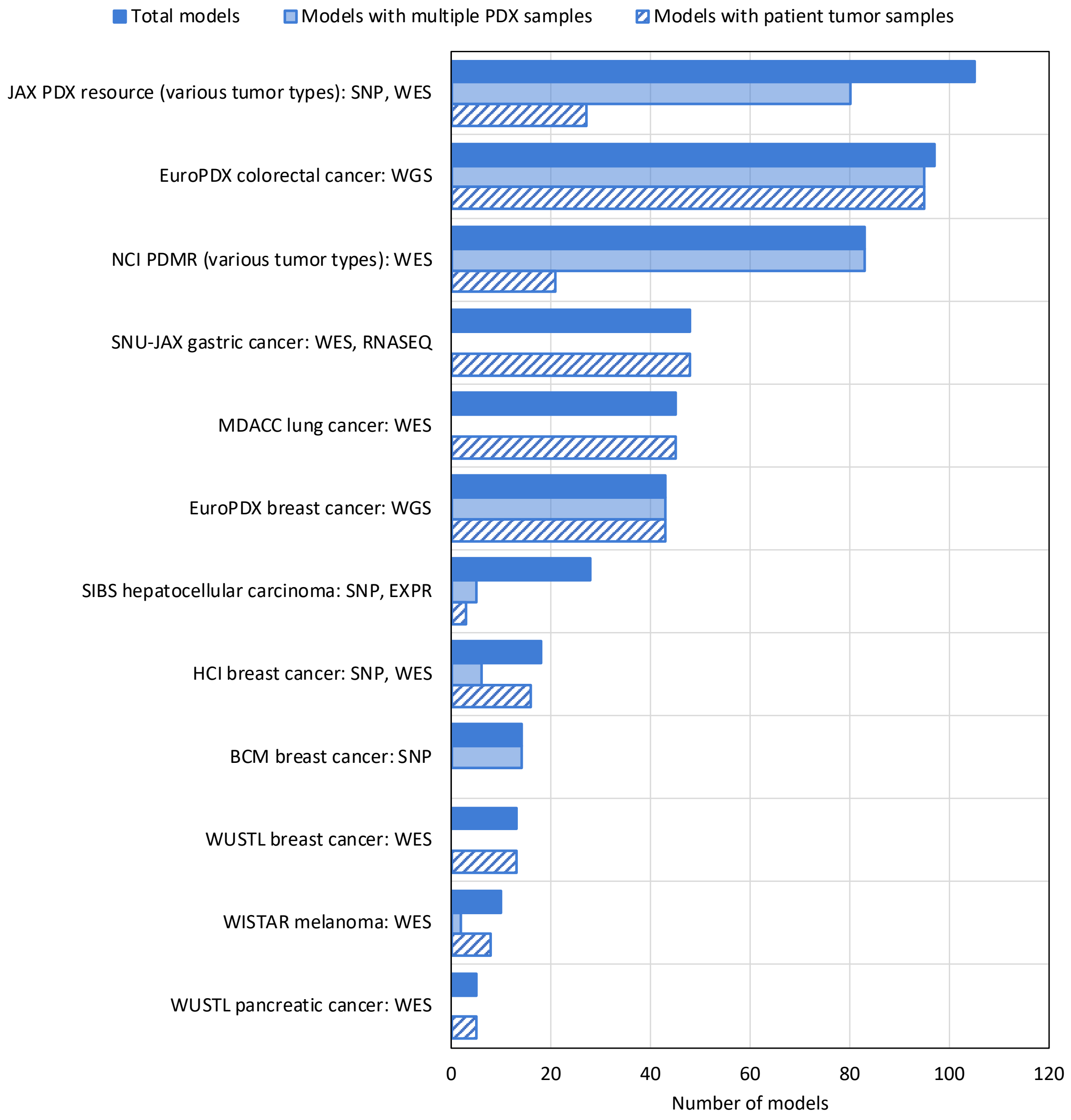

Supplementary Fig. 1: Summary of PDX models collected from various centers in the PDXNET and EuroPDX consortium, and publicly available data.

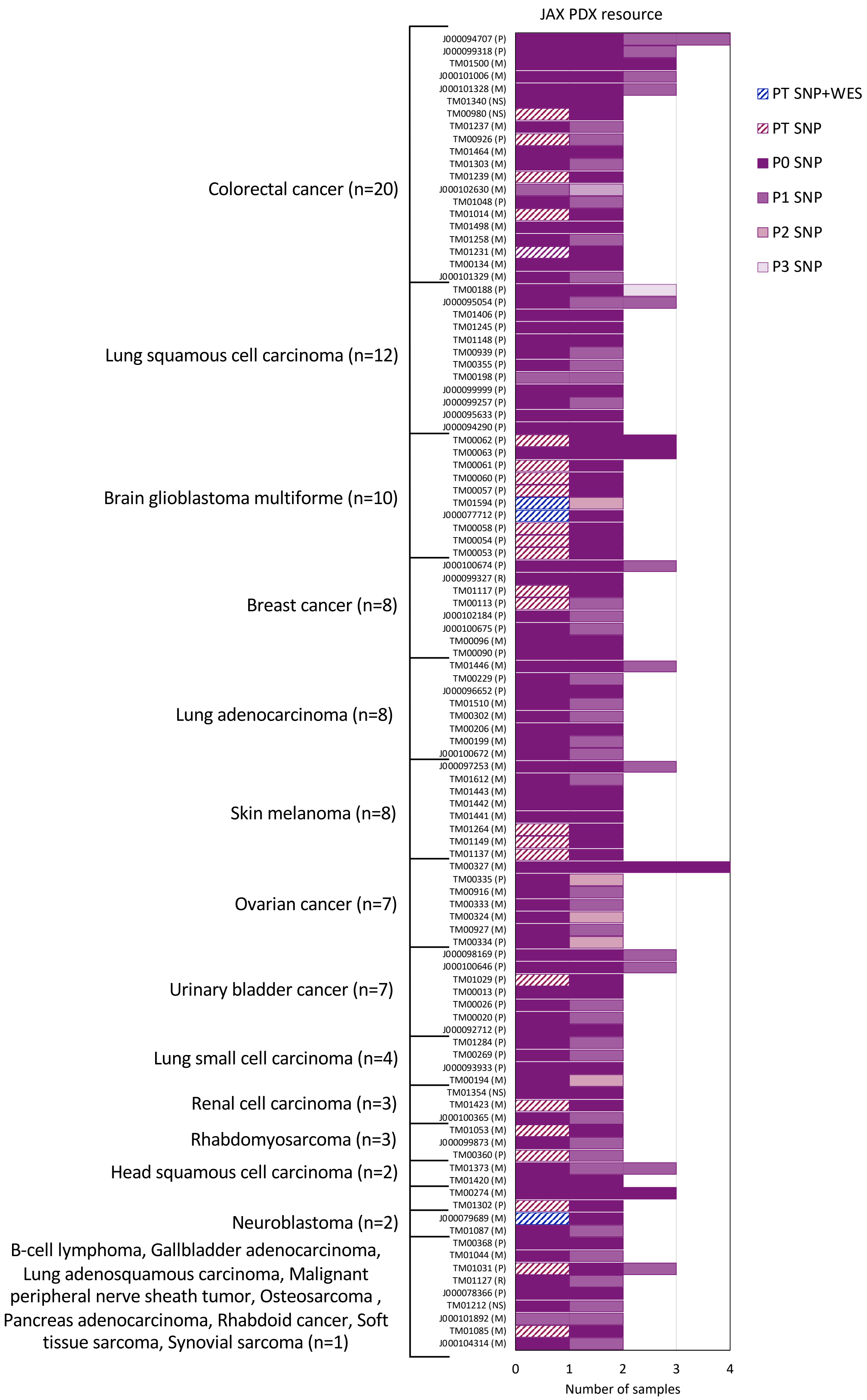

Supplementary Fig. 2: Number of patient tumor and PDX passages per model in the JAX PDX resource consisting of various tumor types assayed by Affymetrix SNP 6.0 array and whole exome sequencing (P: Primary malignancy, M: Metastatic, R: Recurrent/Relapse, NS: Not specified).

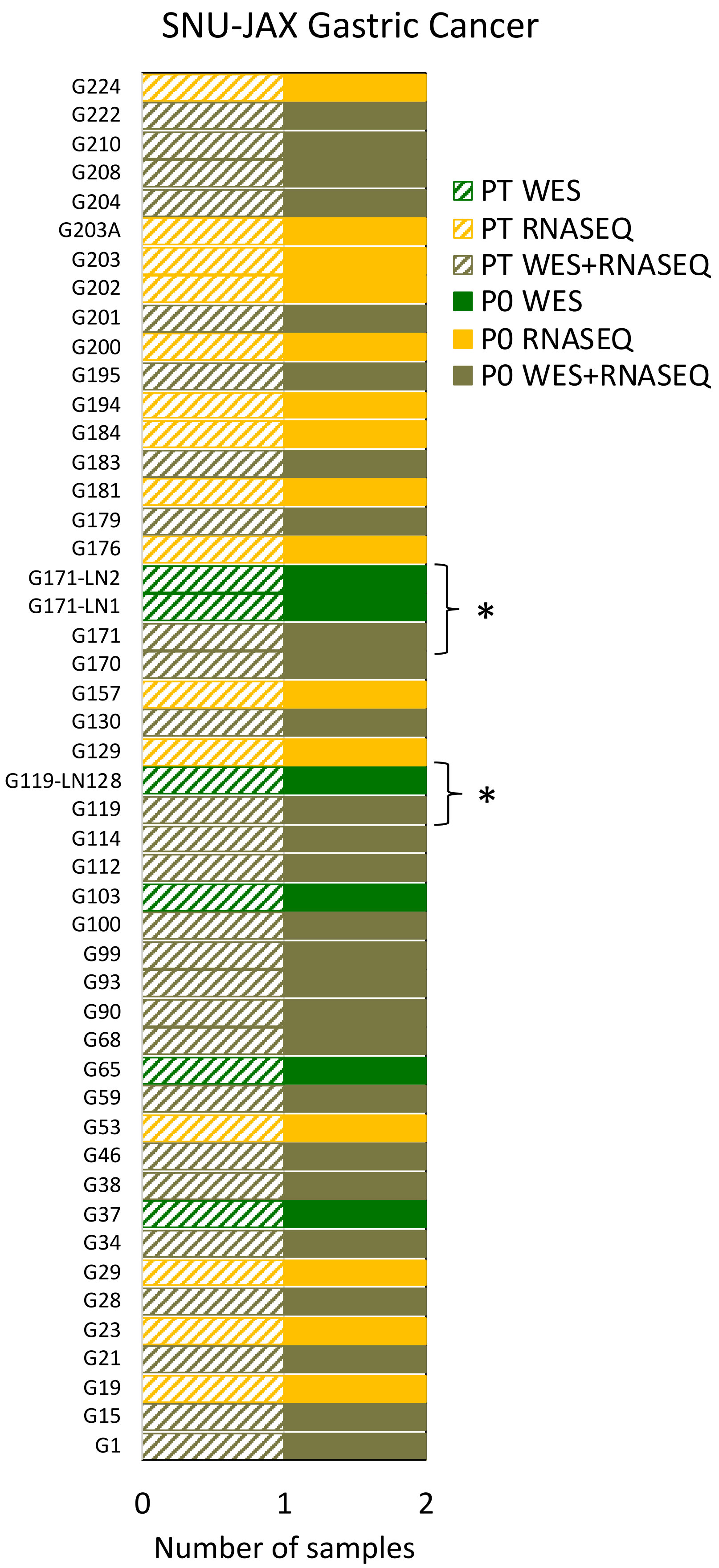

Supplementary Fig. 3: Number of patient tumor and PDX passages per model in SNU-JAX gastric cancer dataset assayed by whole-exome sequencing (WES) and RNA sequencing (RNASEQ). Models labeled with ”LN” are lymph node metastatic tumors for the same patient. (*: Multiple patient tumors available for the same patient, different relapse time points or different metastatic sites)

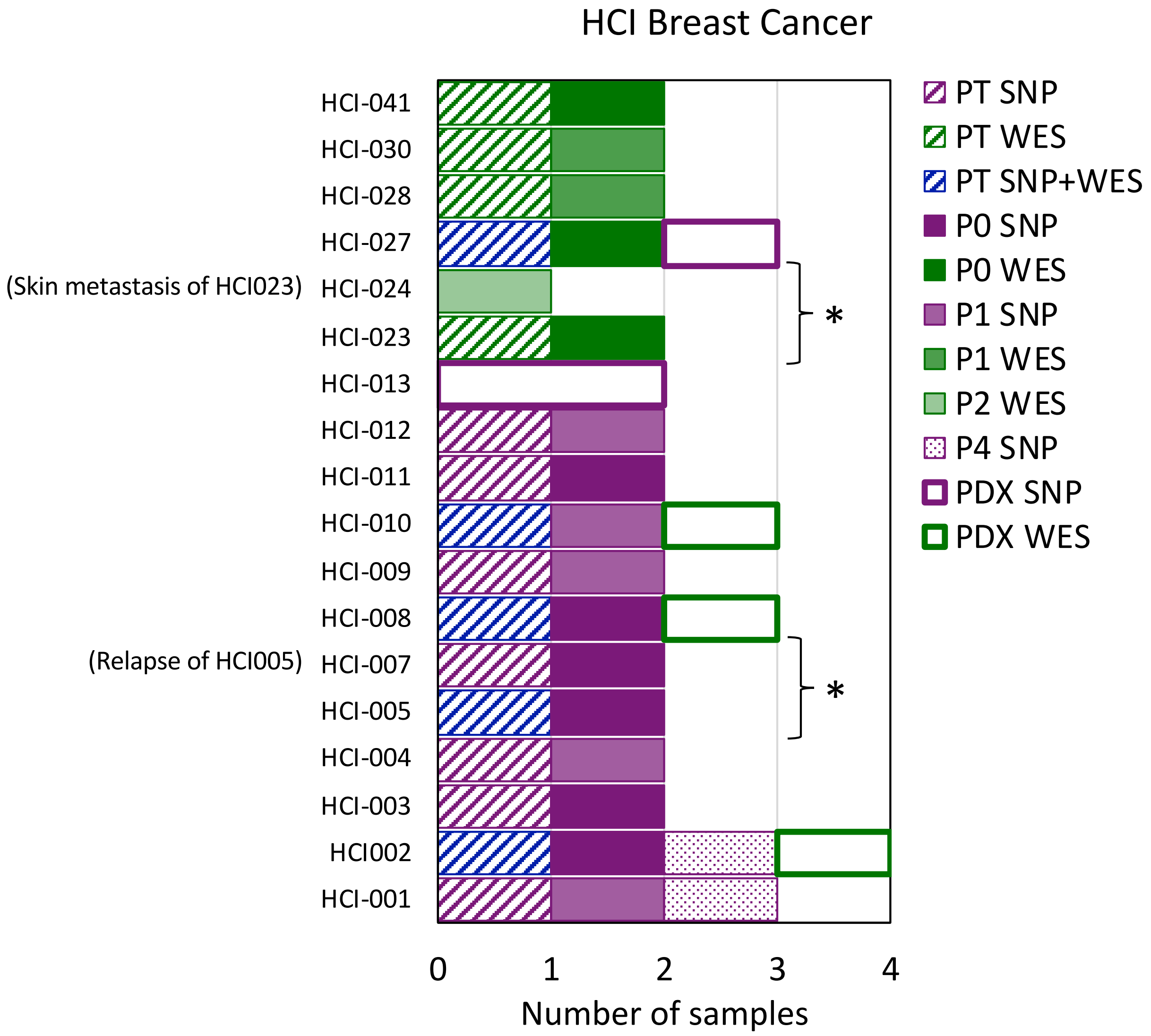

Supplementary Fig. 4: Number of patient tumor and PDX passages per model in the HCI breast cancer dataset assayed by whole-exome sequencing (WES), Affymetrix SNP 6.0 array and Illumina Infinium Omni2.5Exome8 (v1.3) SNP array. HCI-007 is relapse tumor from the patient of HCI-005, HCI-024 is a skin metastasis tumor from the patient of HCI-023. Samples labeled with “PDX” indicates passage number is unknown. (*: Multiple tumors available for the same patient, different relapse time points or different metastatic sites)

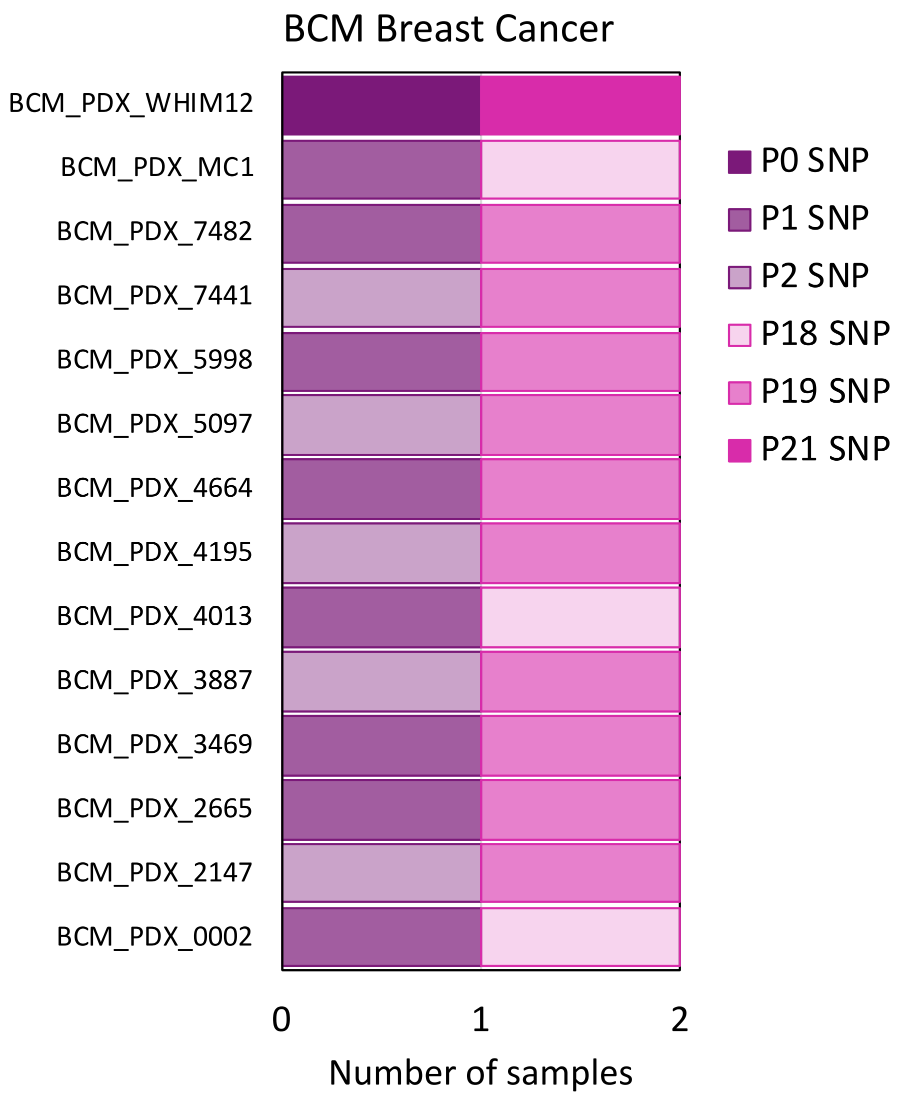

Supplementary Fig. 5: Number of PDX passages per model in the BCM breast cancer dataset assayed by Illumina Infinium Omni2.5Exome8 (v1.4) SNP array.

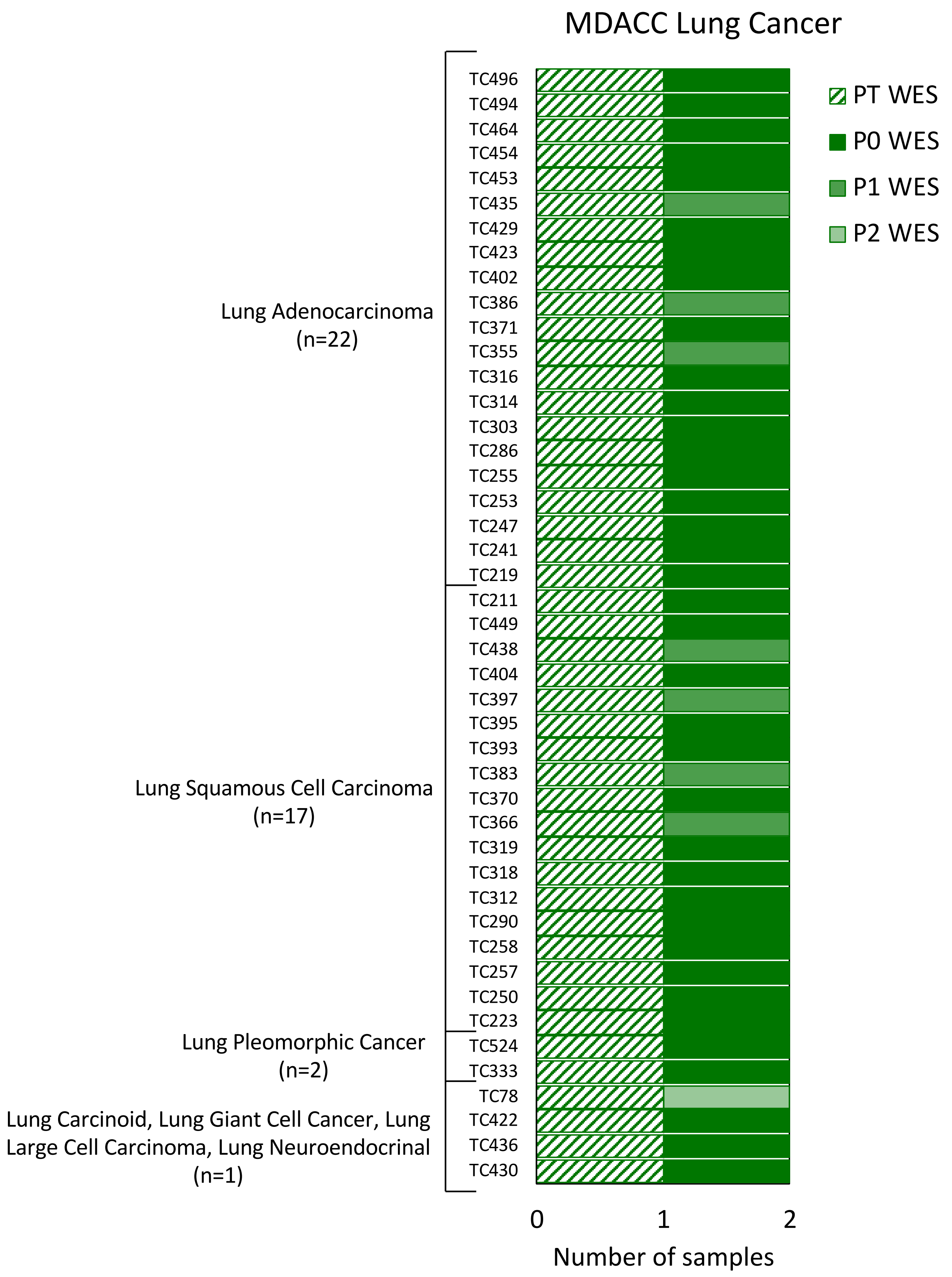

Supplementary Fig. 6: Number of patient tumor and PDX passages per model in the MDACC lung cancer dataset assayed by whole-exome sequencing (WES).

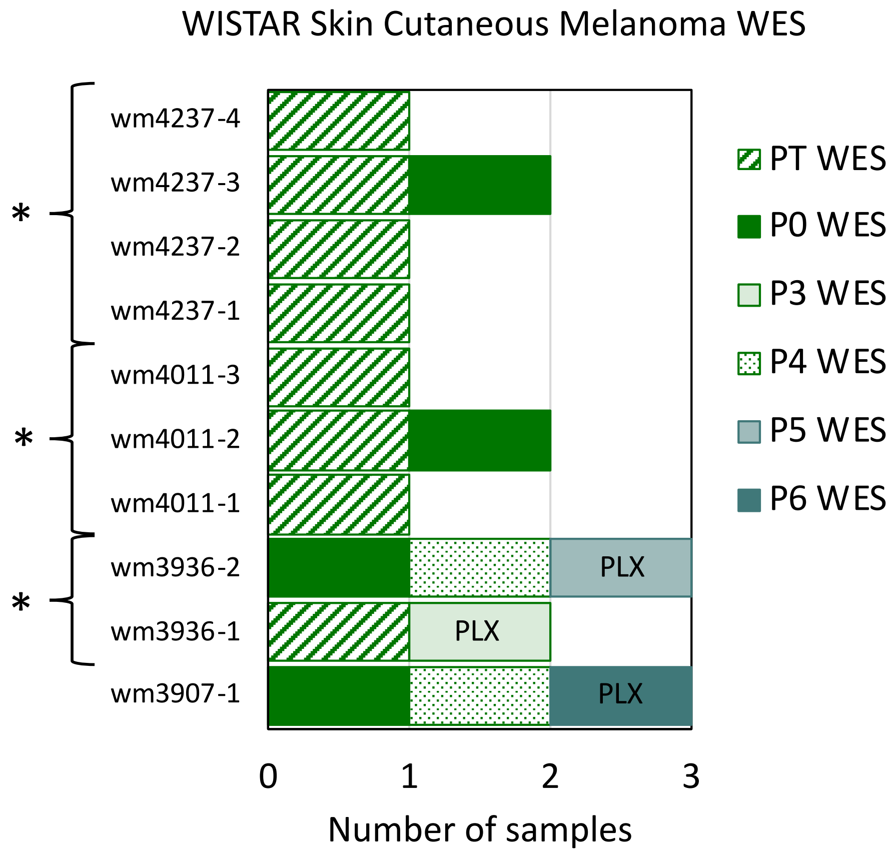

Supplementary Fig. 7: Number of patient tumor and PDX passages per model in Wistar skin cutaneous melanoma dataset assayed by whole-exome sequencing (WES). PDX samples labeled with ”PLX” are BRAF inhibitor (PLX) treated. (*: Multiple tumors available for the same patient, different relapse time points or different metastatic sites)

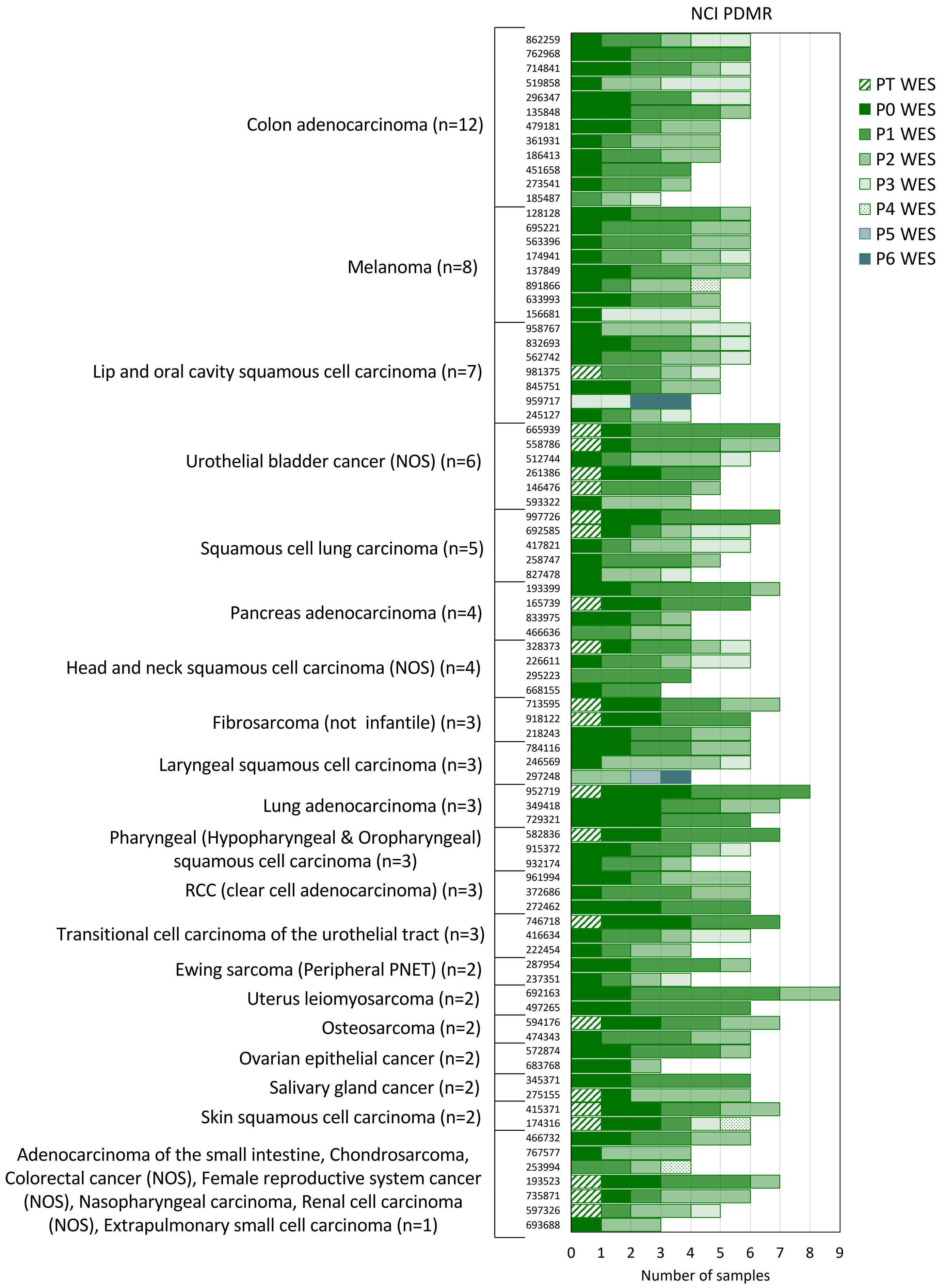

Supplementary Fig. 8: Number of patient tumor and PDX passages per model in the NCI PDMR resource consisting of various tumor types assayed by whole exome sequencing.

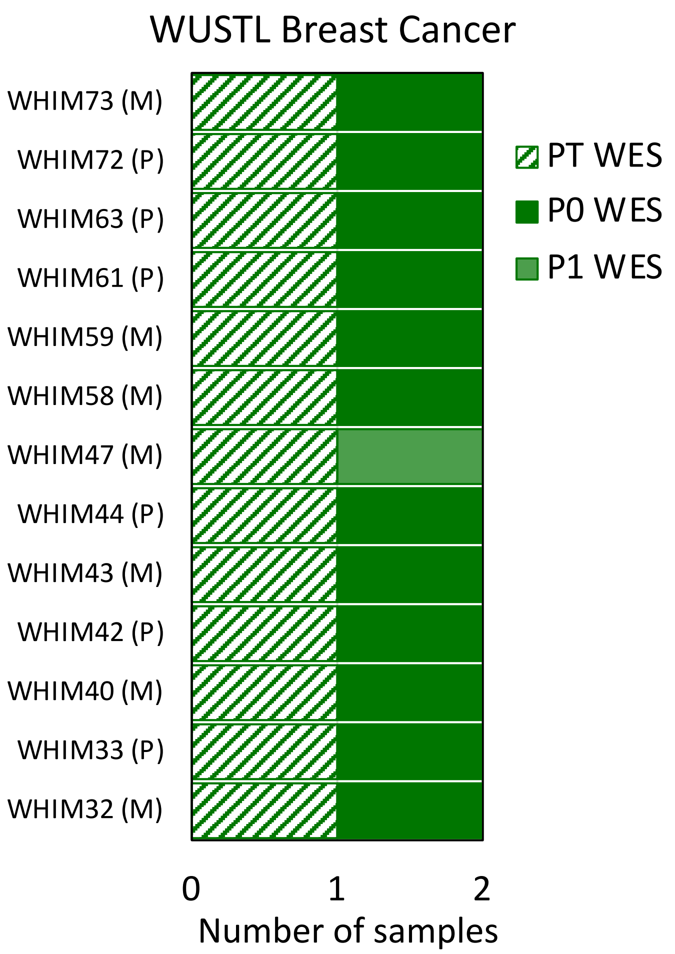

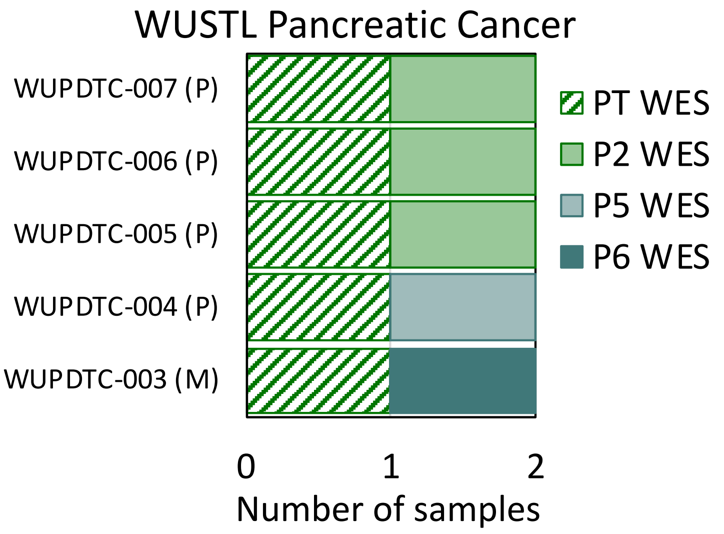

Supplementary Fig. 9: Number of patient tumor and PDX passages per model in the WUSTL breast cancer and pancreatic cancer datasets assayed by whole-exome sequencing (WES).

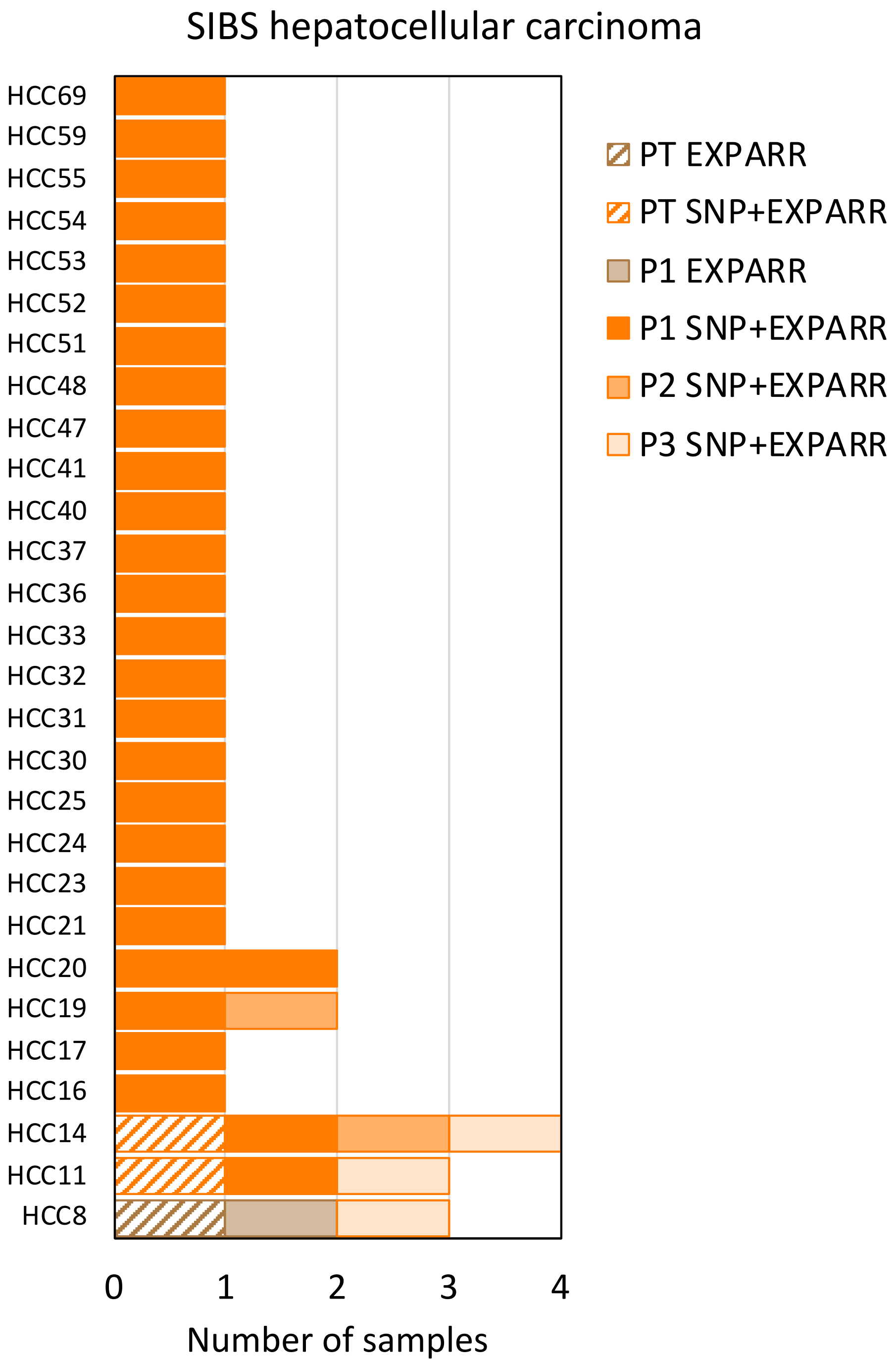

Supplementary Fig. 10: Number of patient tumor and PDX passages per model in the SIBS hepatocellular carcinoma dataset assayed by Affymetrix SNP 6.0 array and Affymetrix gene expression array (EXPARR).

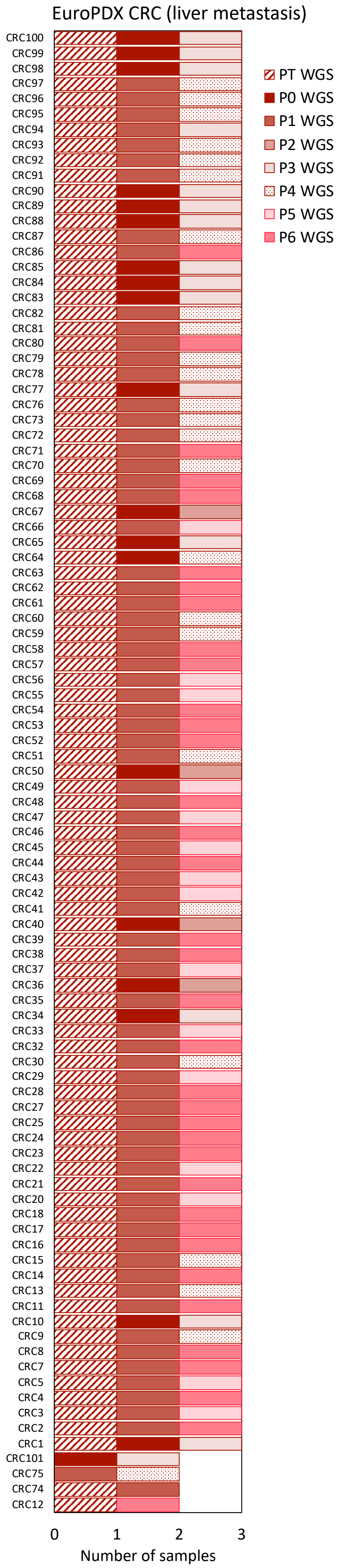

Supplementary Fig. 11: Number of patient tumor and PDX passages per model in the EuroPDX colorectal cancer (CRC) liver metastasis dataset assayed by whole-genome sequencing.

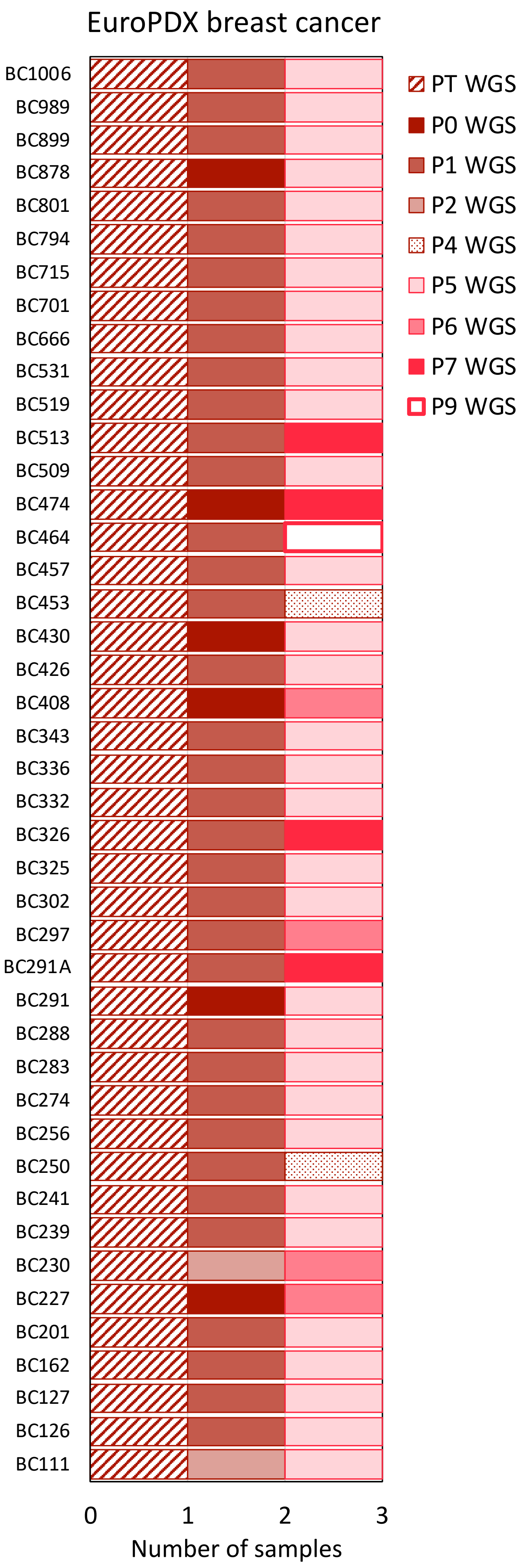

Supplementary Fig. 12: Number of patient tumor and PDX passages per model in the EuroPDX breast cancer (BRCA) dataset assayed by whole-genome sequencing.

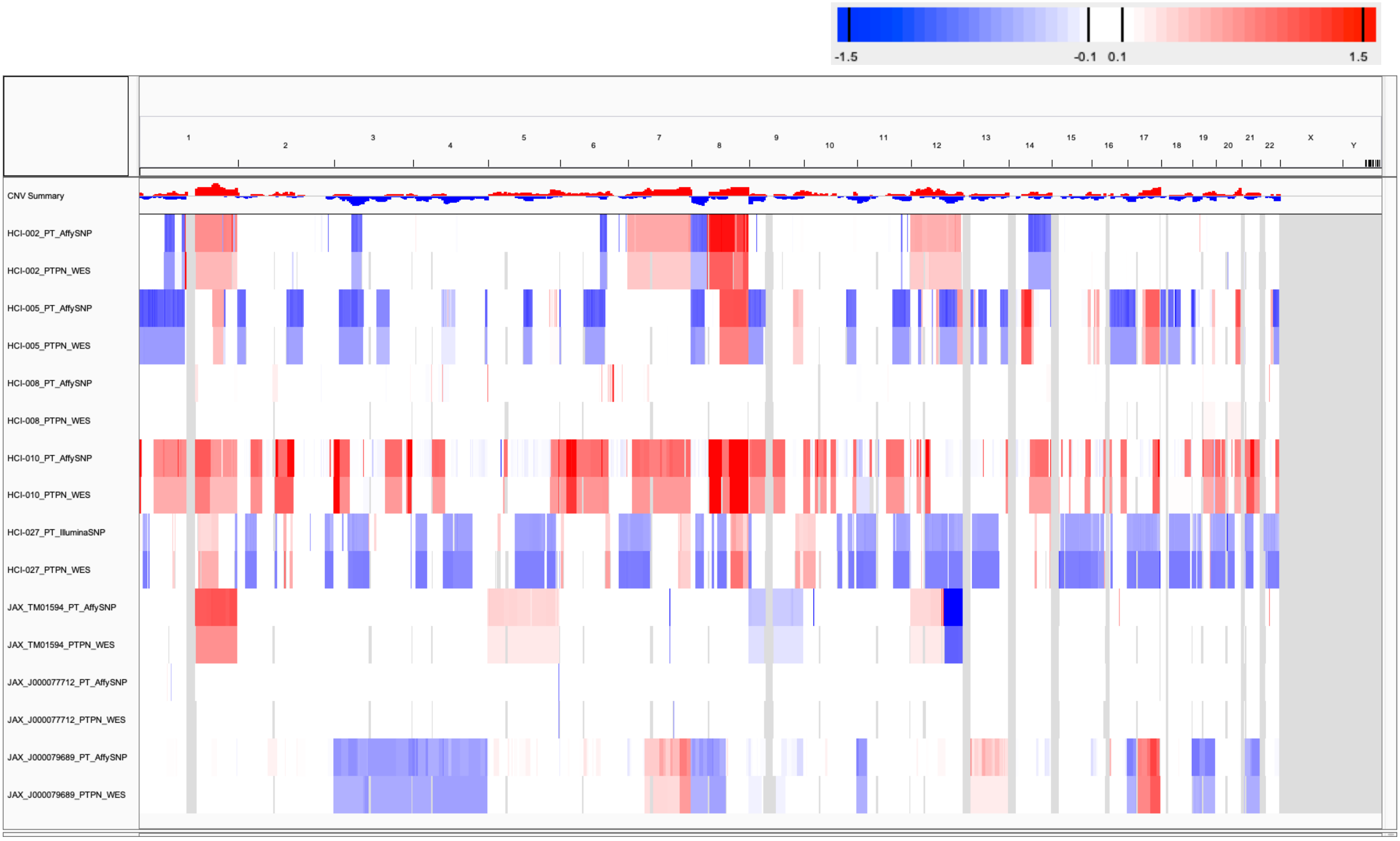

Supplementary Fig. 13: CNA profiles for matched patient tumor samples estimated from SNP array and WES for ”SNP vs WES” validation (see Supplementary Table 3).

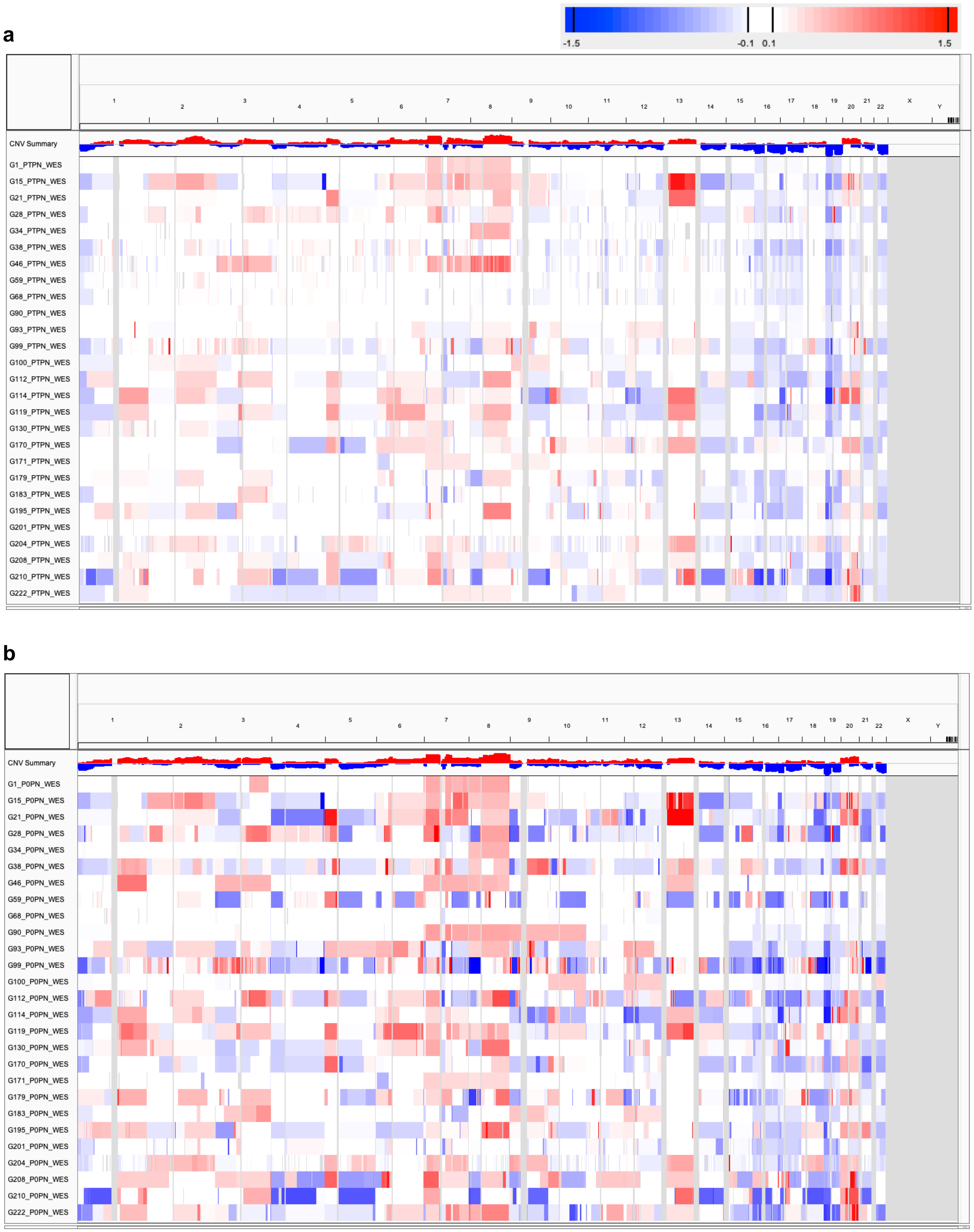

Supplementary Fig. 14: CNA profiles for (**a**) patient tumor and (**b**) PDX samples estimated from WES used for ”WES vs RNASEQ (NORM/TUM)” validation (see Supplementary Table 3).

a

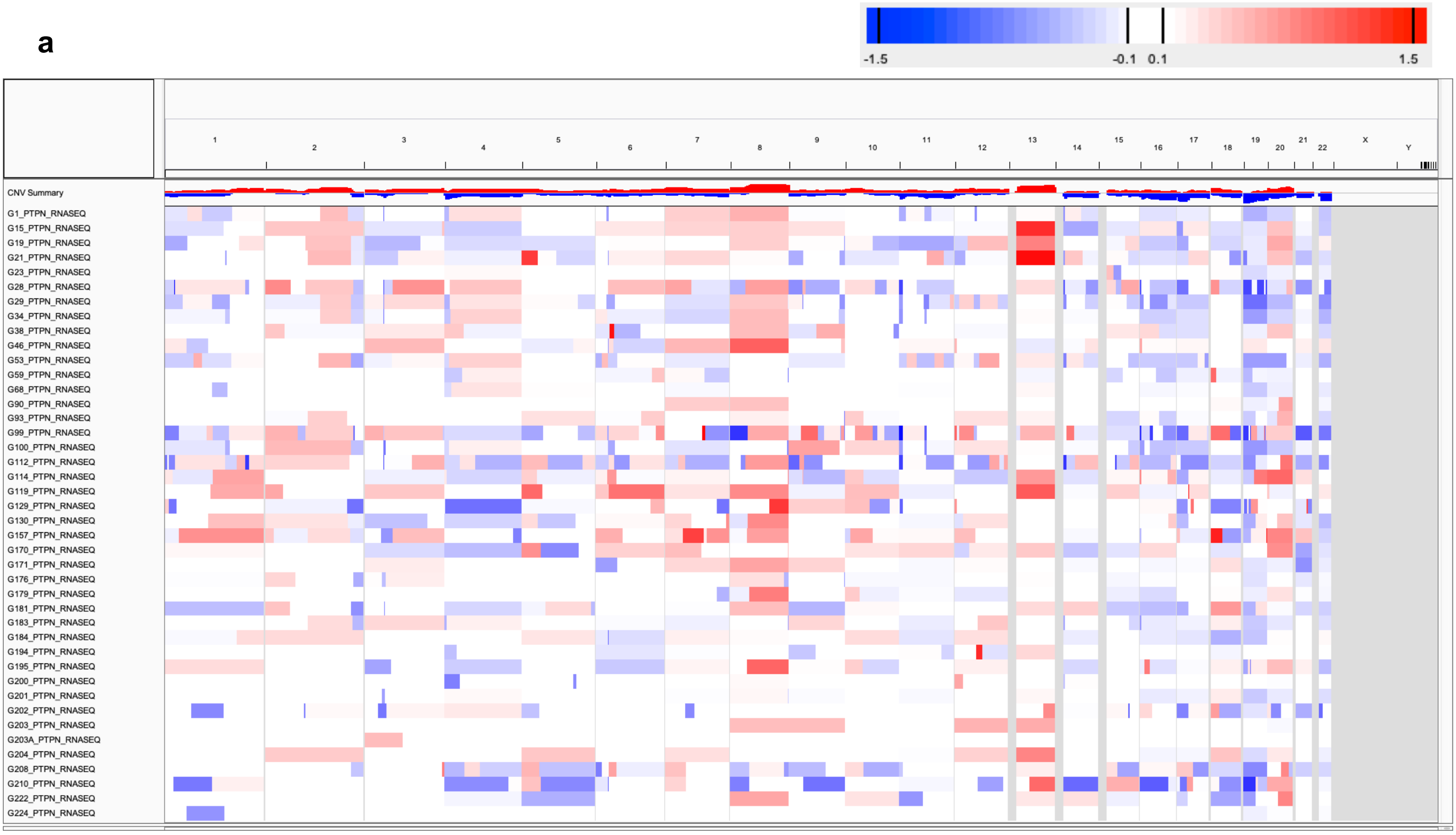

b

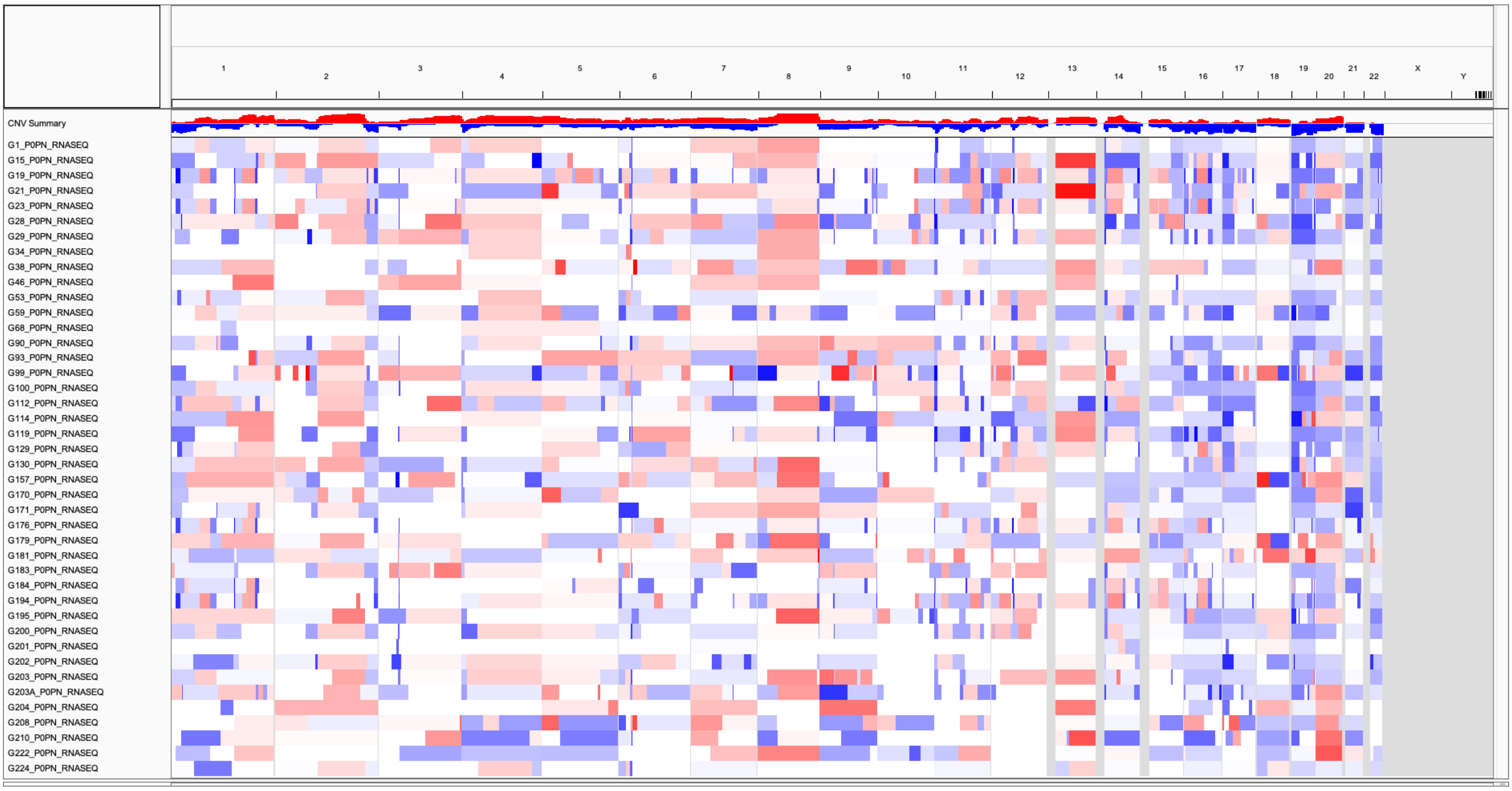

Supplementary Fig. 15: (Continue next page)

c

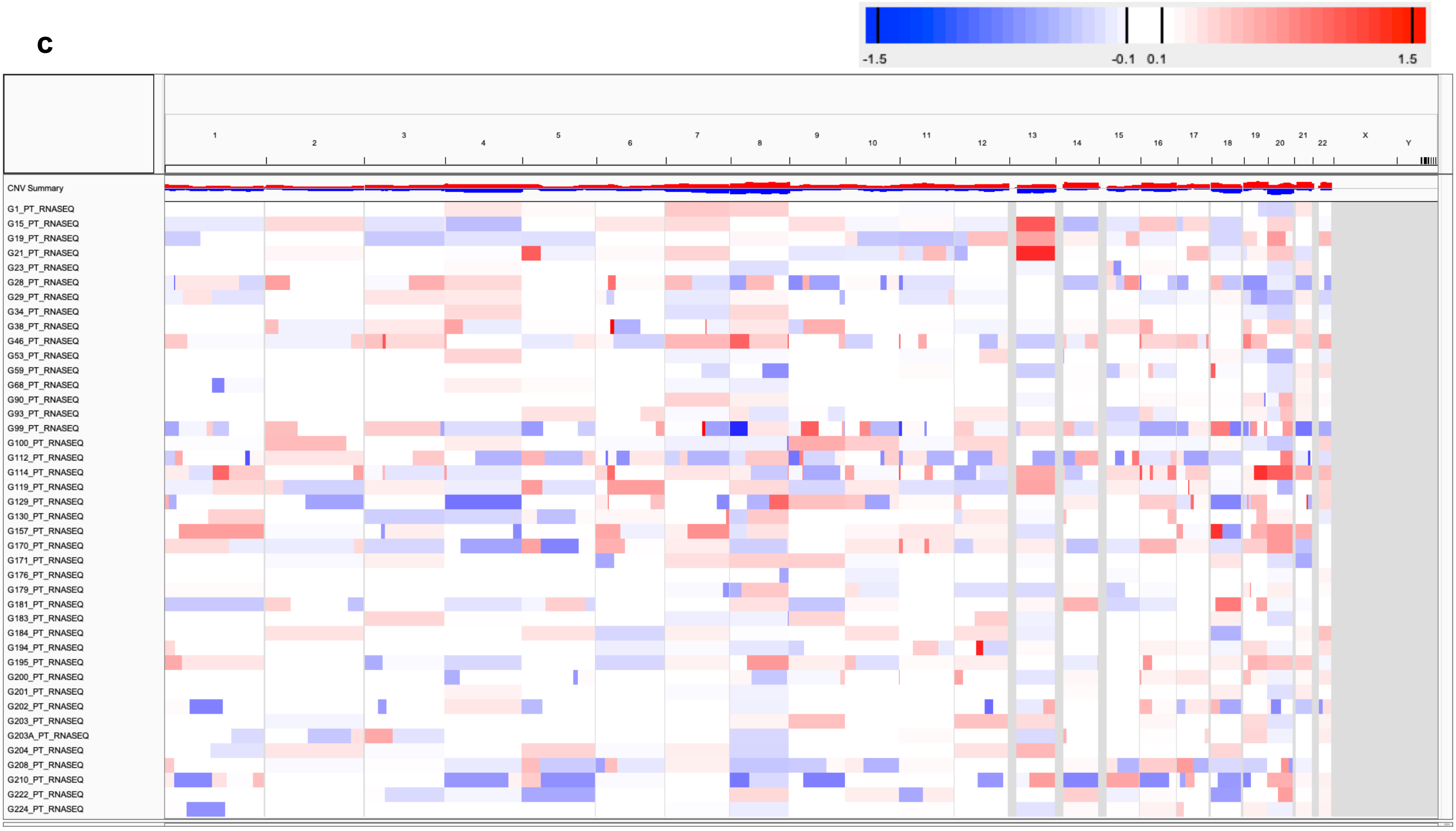

d

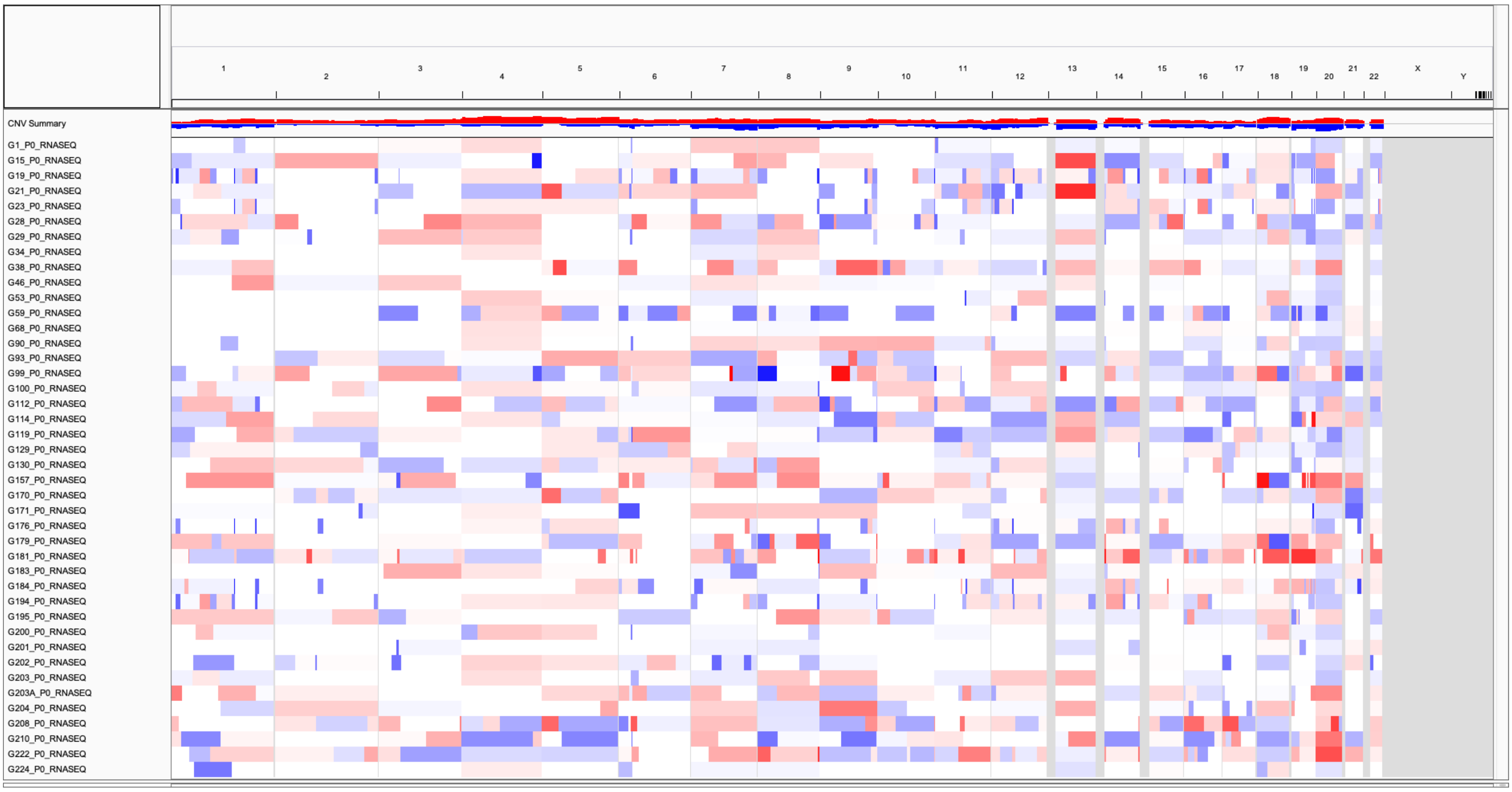

Supplementary Fig. 15: CNA profiles for (**a**) patient tumor and (**b**) PDX samples estimated from RNA-Seq, normalized by median expression of normal samples of the same tumor type, used for ”WES vs RNASEQ (NORM)” and “RNASEQ NORM vs TUM” validation. CNA profiles for (**c**) patient tumor and (**d**) PDX samples estimated from RNA-Seq, normalized by median expression of same set of patient tumors, used for ”WES vs RNASEQ (TUM)” and “RNASEQ NORM vs TUM” validation (see Supplementary Table 3).

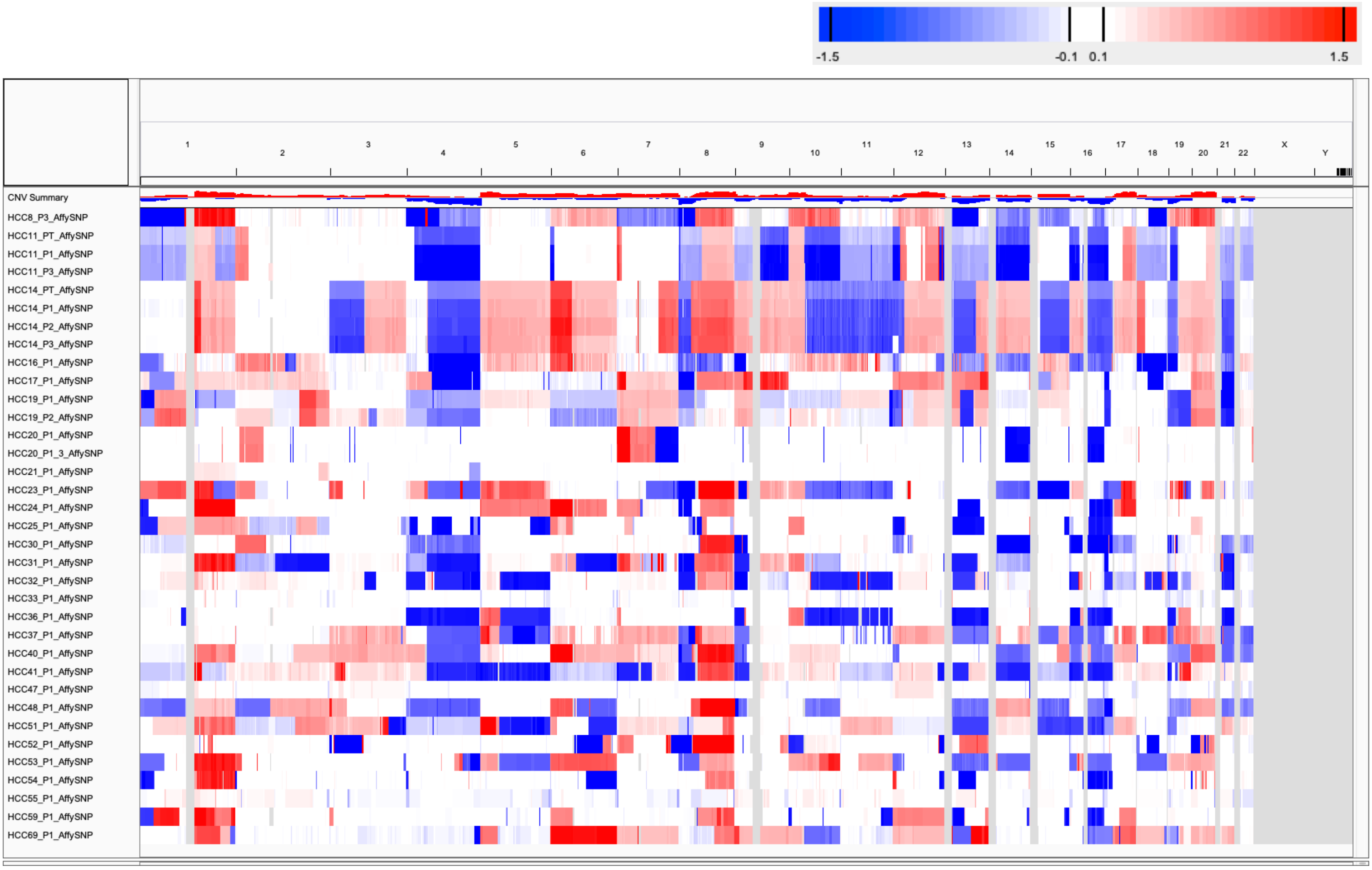

Supplementary Fig. 16: CNA profiles for patient tumor and PDX samples estimated from SNP array used for ”SNP vs EXPARR (NORM/TUM)” validation (see Supplementary Table 3).

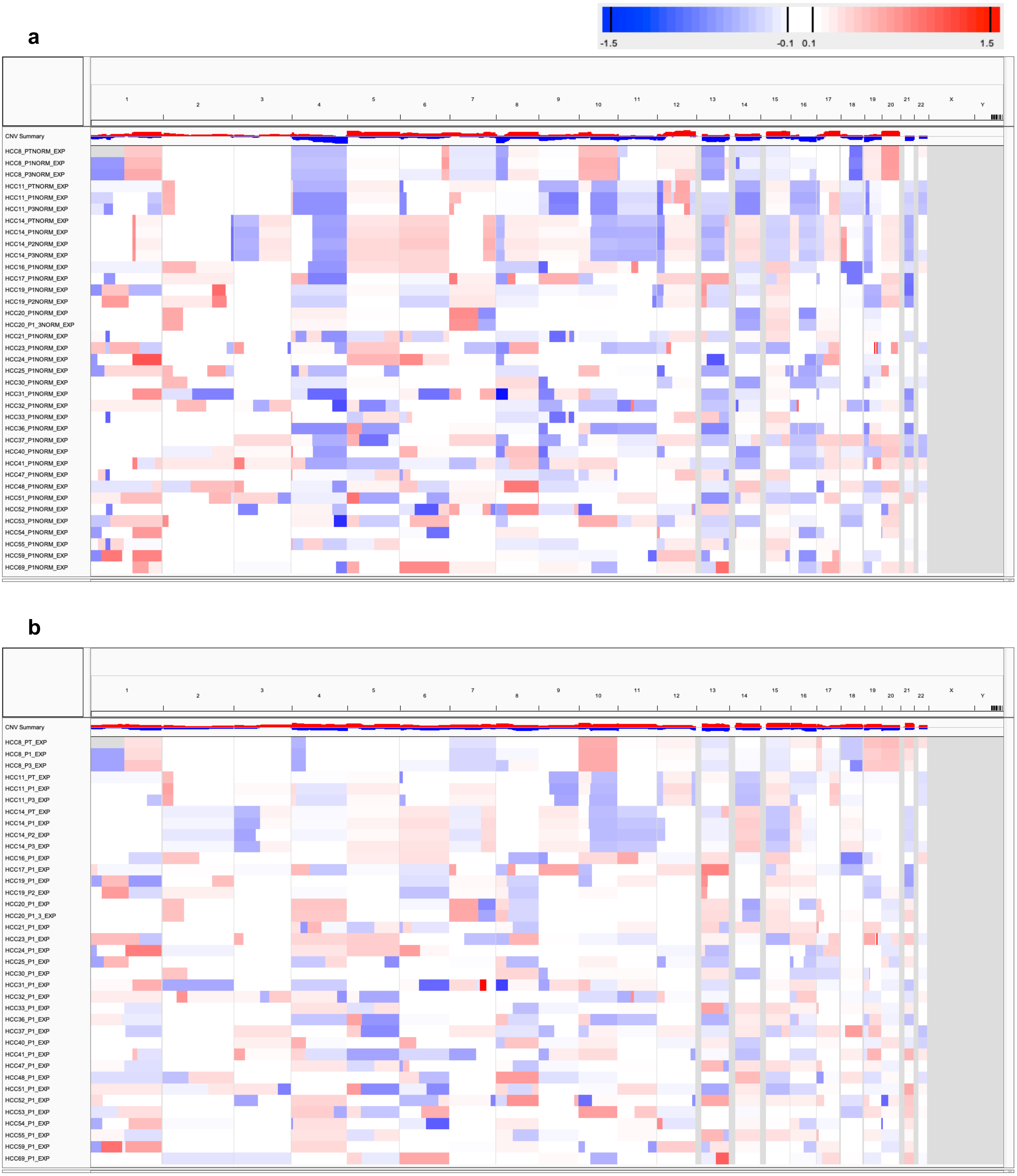

Supplementary Fig. 17: CNA profiles for patient tumor and PDX samples estimated from gene expression array, normalized by (**a**) median expression of normal samples of the same tumor type and (**b**) median expression of same set of patient tumors, used for ”SNP vs EXPARR (NORM/TUM)” and “EXPARR NORM vs TUM” validation (see Supplementary Table 3).

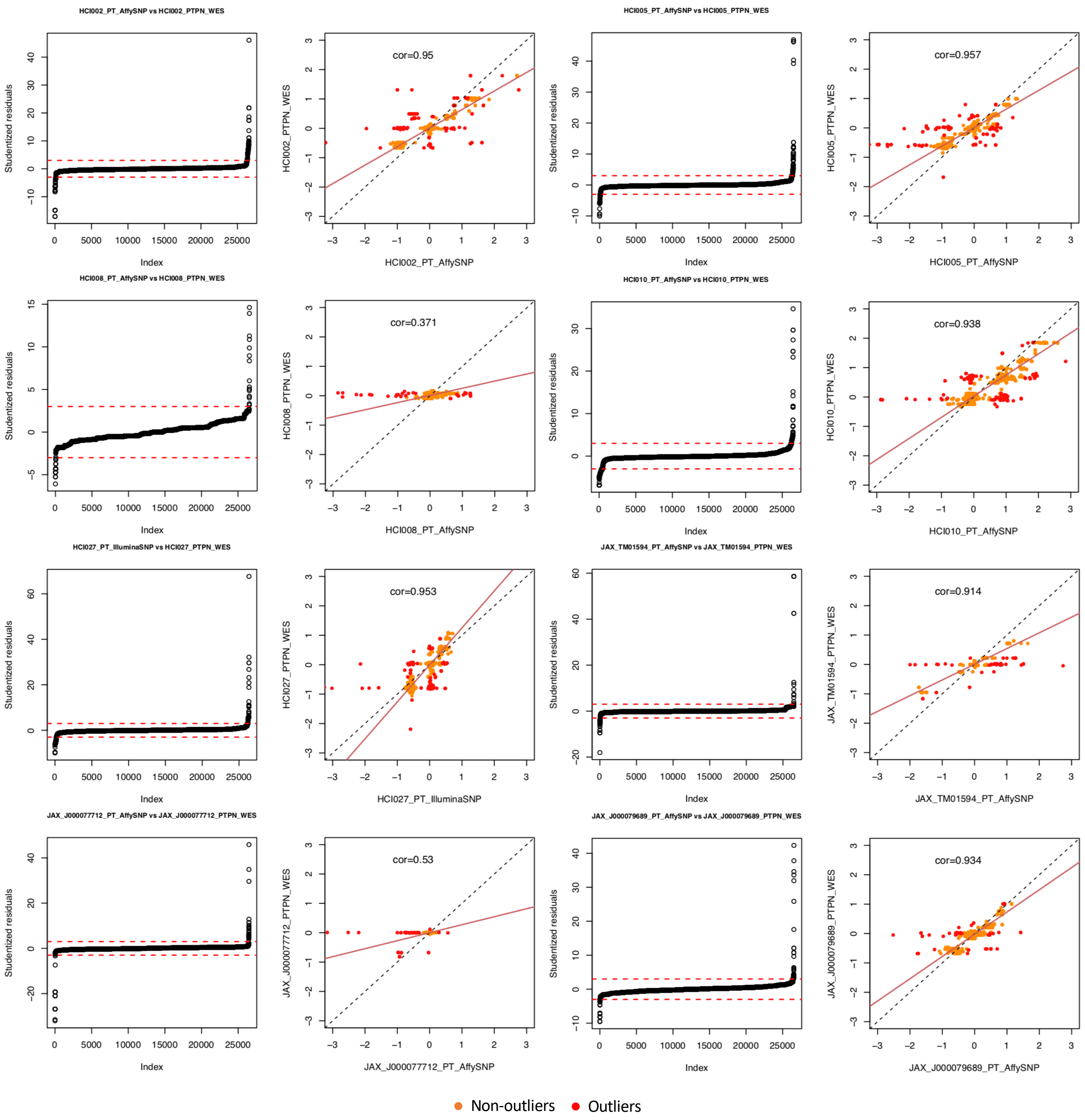

Supplementary Fig. 18: Pearson correlation and linear regression of the $\log_2$(CN ratio) of 100kb-windows binned from copy number segments of CNA profiles between matched patient tumor samples estimated from SNP array and WES. Outliers of the linear regression are identified by studentized residuals > 3 and < -3.

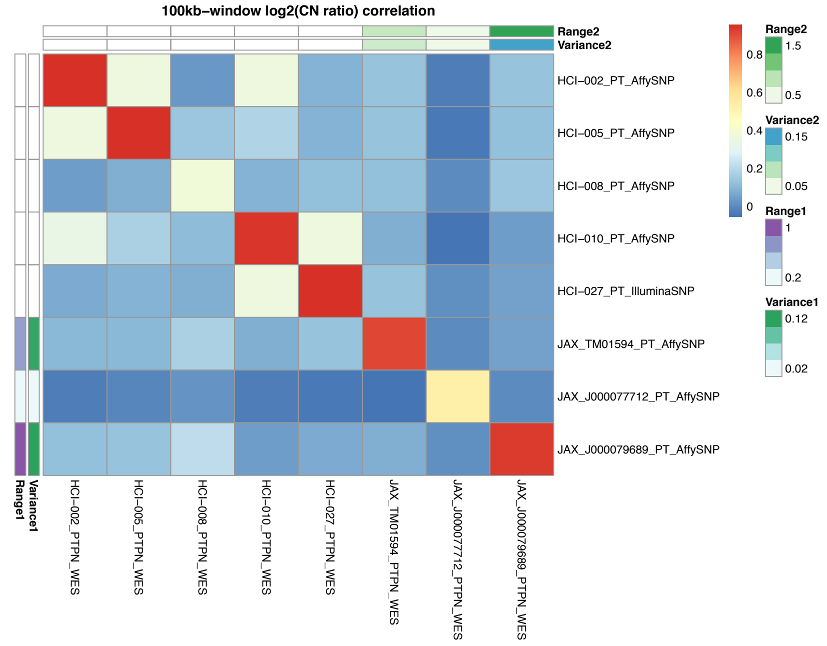

Supplementary Fig. 19: Heatmap representing the Pearson correlation coefficients of the $\log_2$(CN ratio) of 100kb-windows binned from copy number segments of CNA profiles between matched samples estimated from SNP array and WES. The variance and 5-95% inter-percentile range of log2(CN ratio) values were calculated across all 100kb-windows per sample.

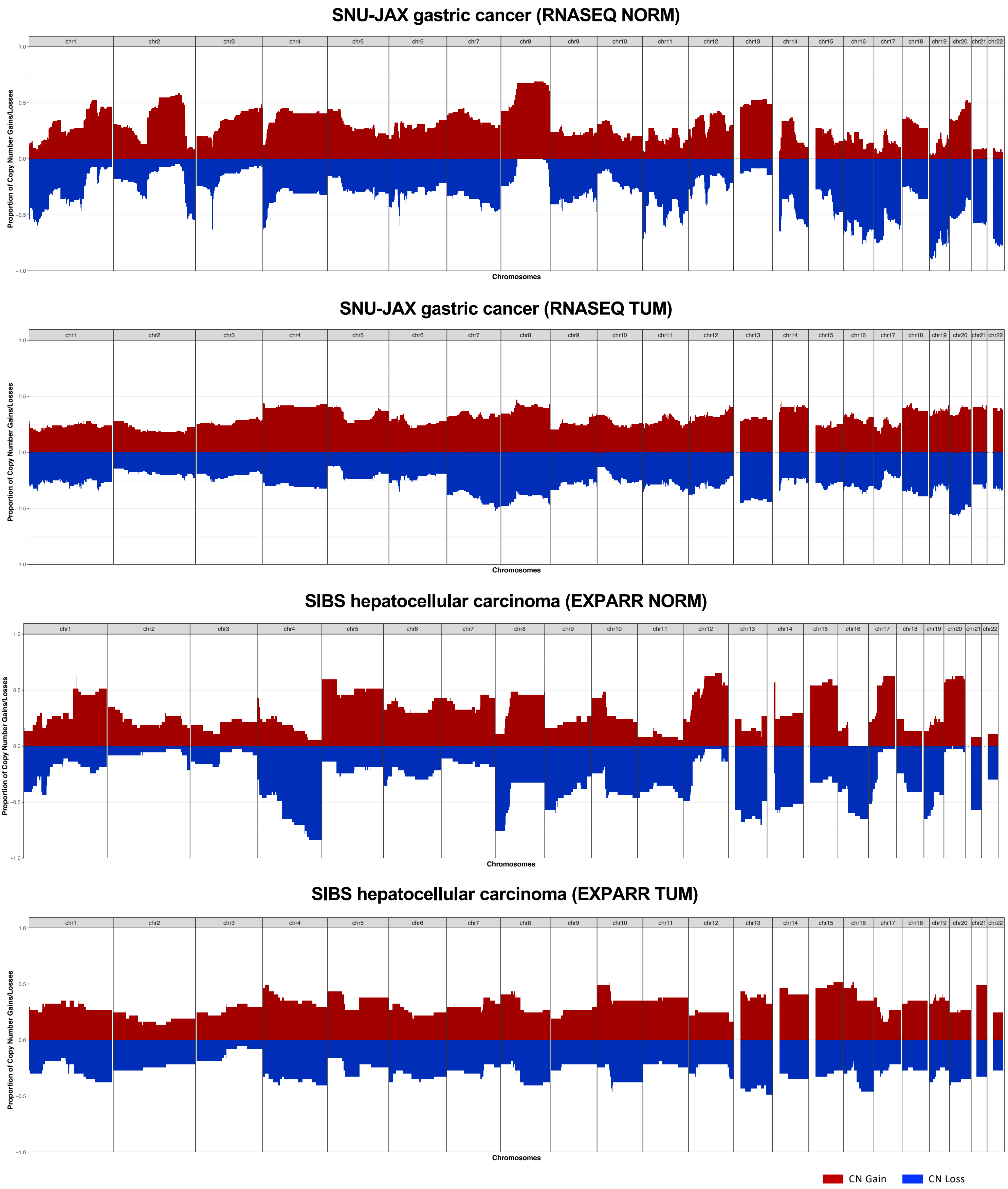

Supplementary Fig. 20: Frequencies of copy number gains (log2(CN ratio) > 0.1, red) and losses (log2(CN ratio) < –0.1, blue) estimated from RNA-Seq and gene expression array normalized by median expression of normal samples of the same tumor type (RNASEQ NORM, EXPARR NORM) or median expression of same set of patient tumors (RNASEQ TUM, EXPARR TUM) (see Supplementary Table 3).

a

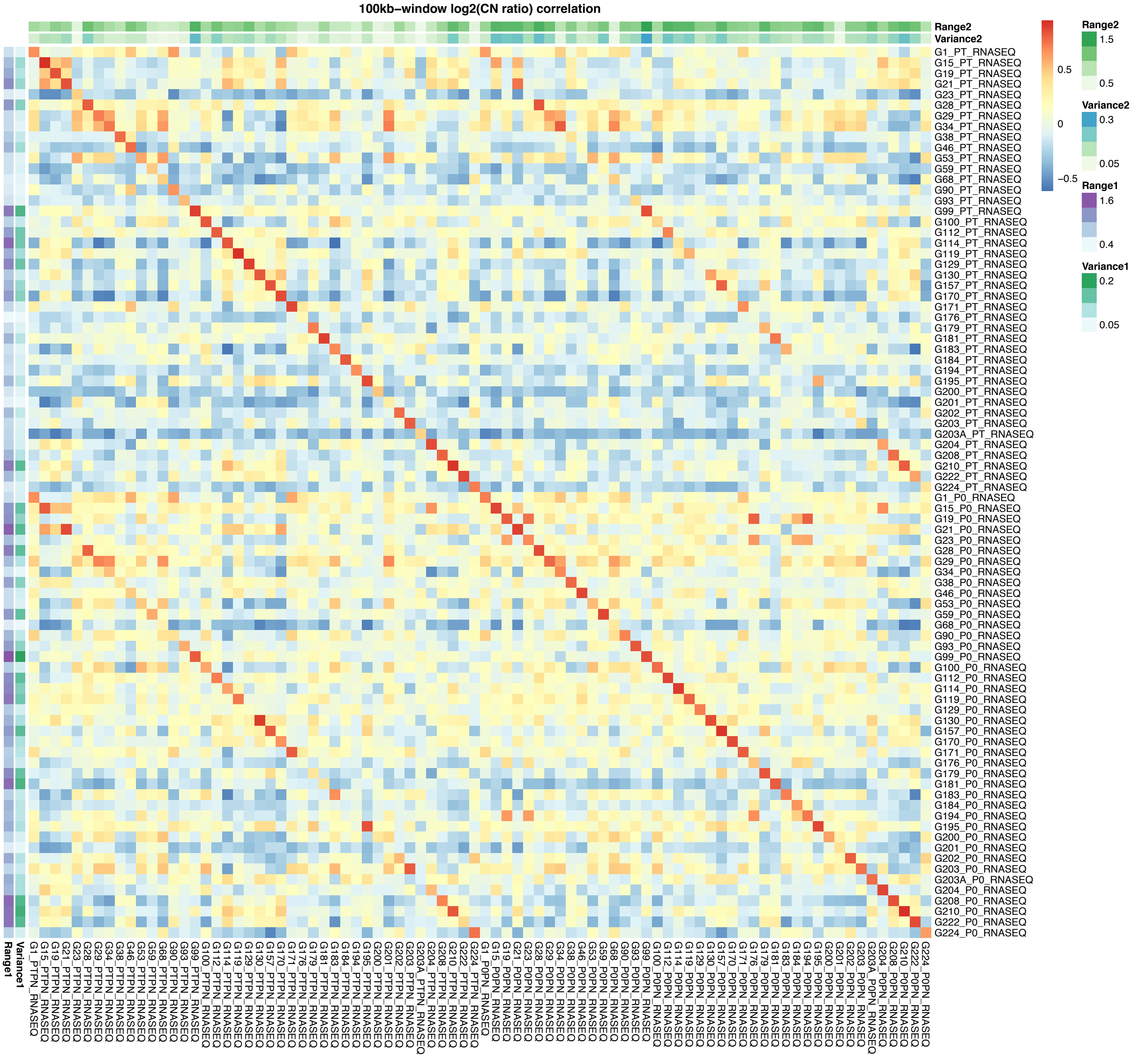

Supplementary Fig. 21: (Continue next page)

b

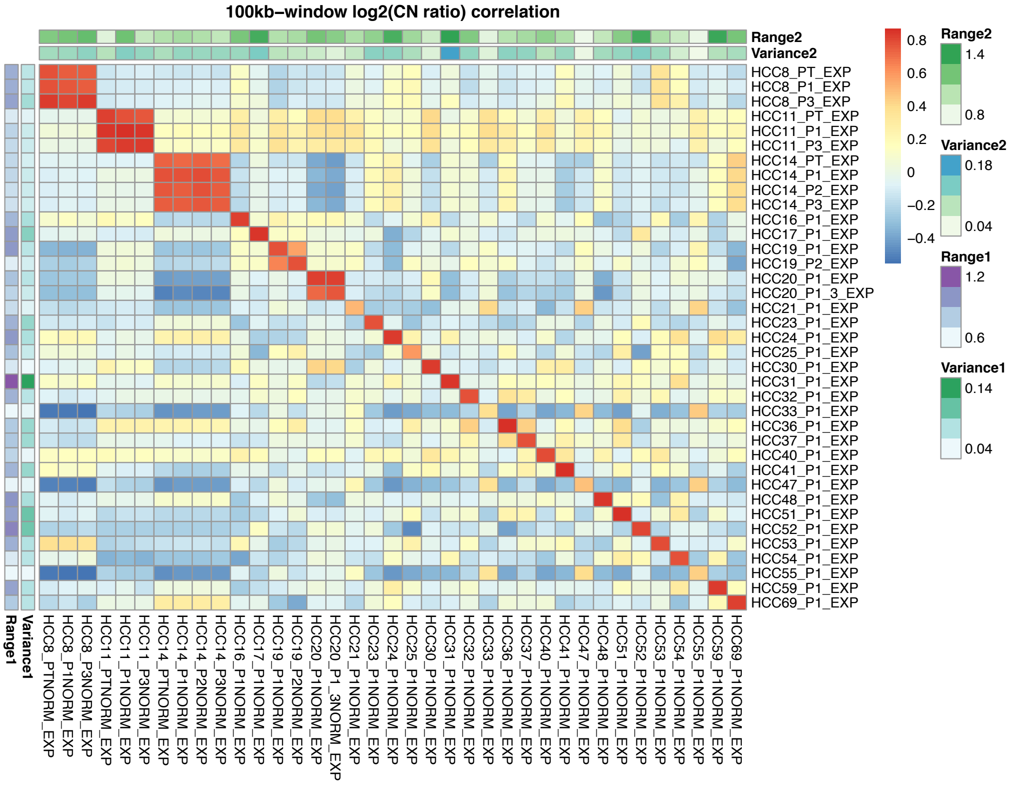

Supplementary Fig. 21: Heatmap representing the Pearson correlation coefficients of the $\log_2$(CN ratio) of 100kb-windows binned from copy number segments of CNA profiles estimated from (**a**) RNA-Seq (RNASEQ NORM vs TUM) and (**b**) gene expression array (EXPARR NORM vs TUM), between matched samples normalized by median expression of normal samples of the same tumor type and median expression of same set of patient tumors. The variance and 5-95% inter-percentile range of log2(CN ratio) values were calculated across all 100kb-windows per sample.

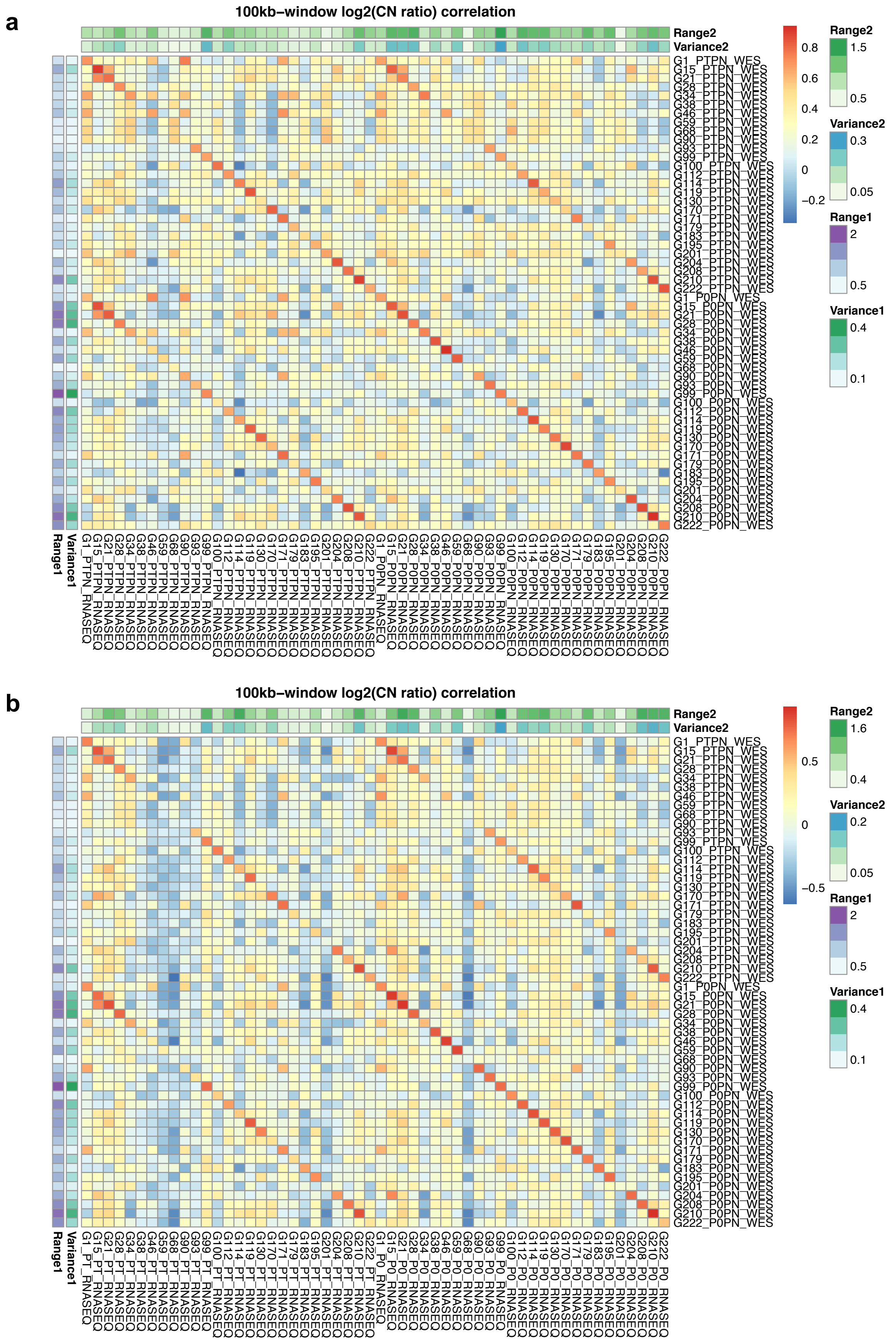

Supplementary Fig. 22: Heatmap representing the Pearson correlation coefficients of the log_2(CN ratio) of 100kb-windows binned from copy number segments of CNA profiles between matched samples estimated from WES and RNA-Seq, (**a**) normalized by median expression of normal samples of the same tumor type “WES vs RNASEQ (NORM)” or (**b**) median expression of same set of patient tumors “WES vs RNASEQ (TUM)”. The variance and 5-95% inter-percentile range of log2(CN ratio) values were calculated across all 100kb-windows per sample.

a

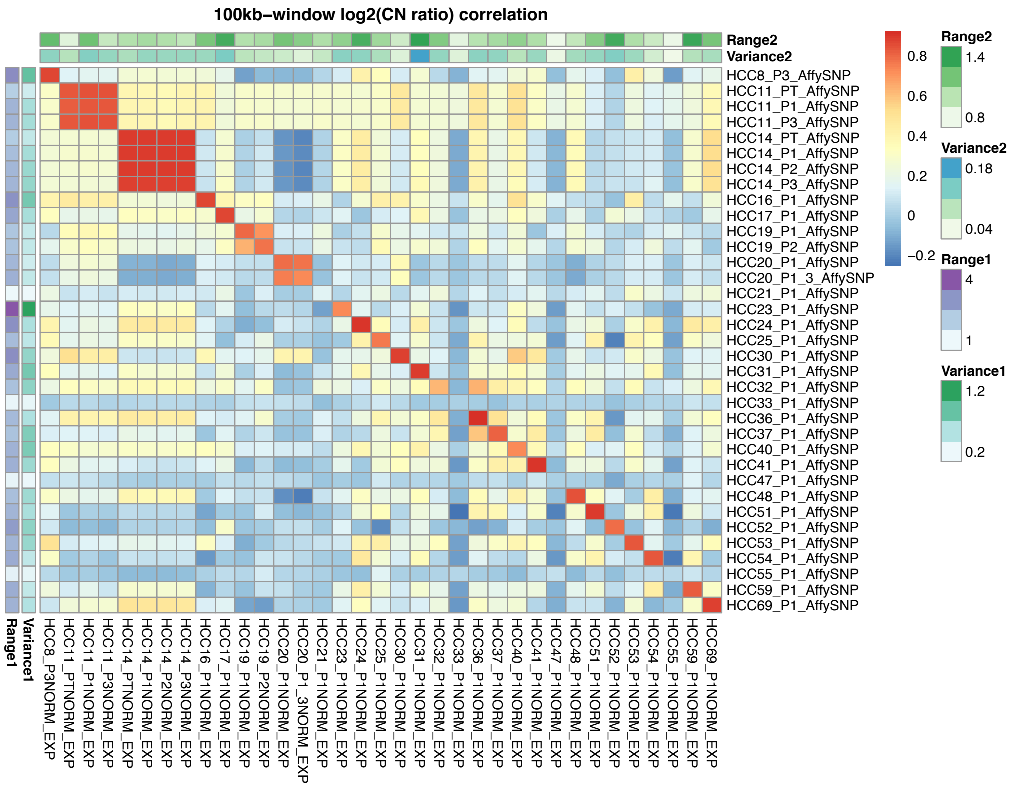

b

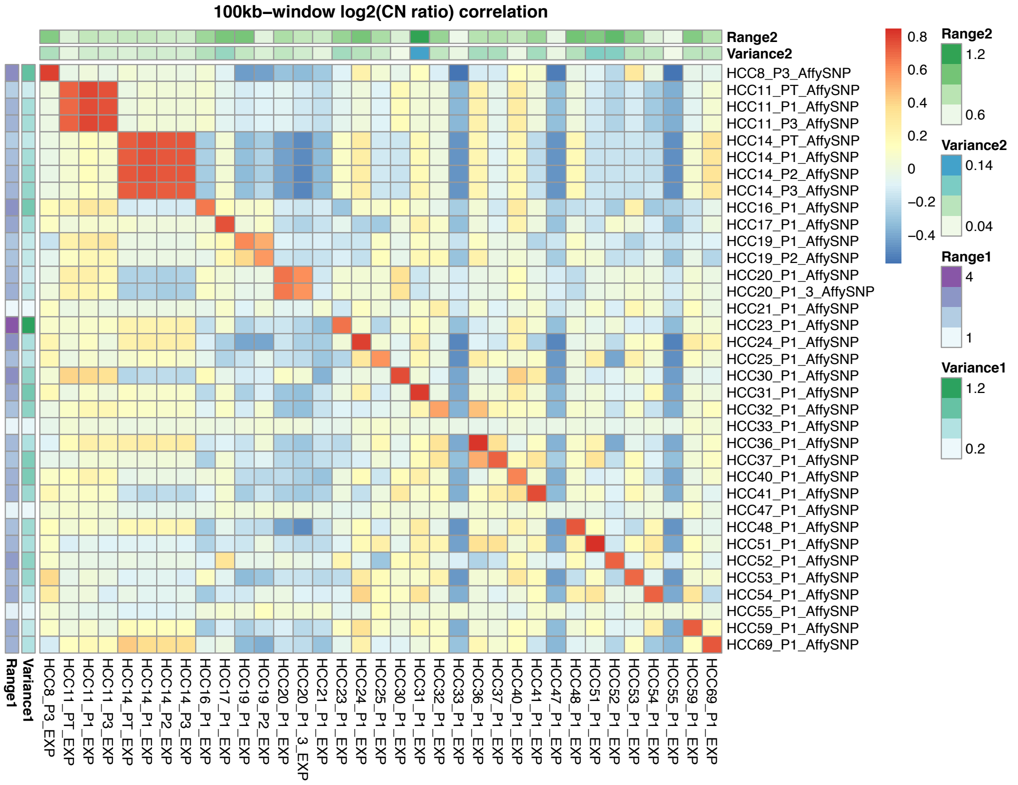

Supplementary Fig. 23: Heatmap representing the Pearson correlation coefficients of the $\log_2$(CN ratio) of 100kb-windows binned from copy number segments of CNA profiles between matched samples estimated from SNP array and gene expression microarray, (**a**) normalized by median expression of normal samples of the same tumor type ”SNP vs EXPARR (NORM)” or (**b**) median expression of same set of patient tumors ”SNP vs EXPARR (TUM)”. The variance and 5-95% inter-percentile range of log2(CN ratio) values were calculated across all 100kb-windows per sample.

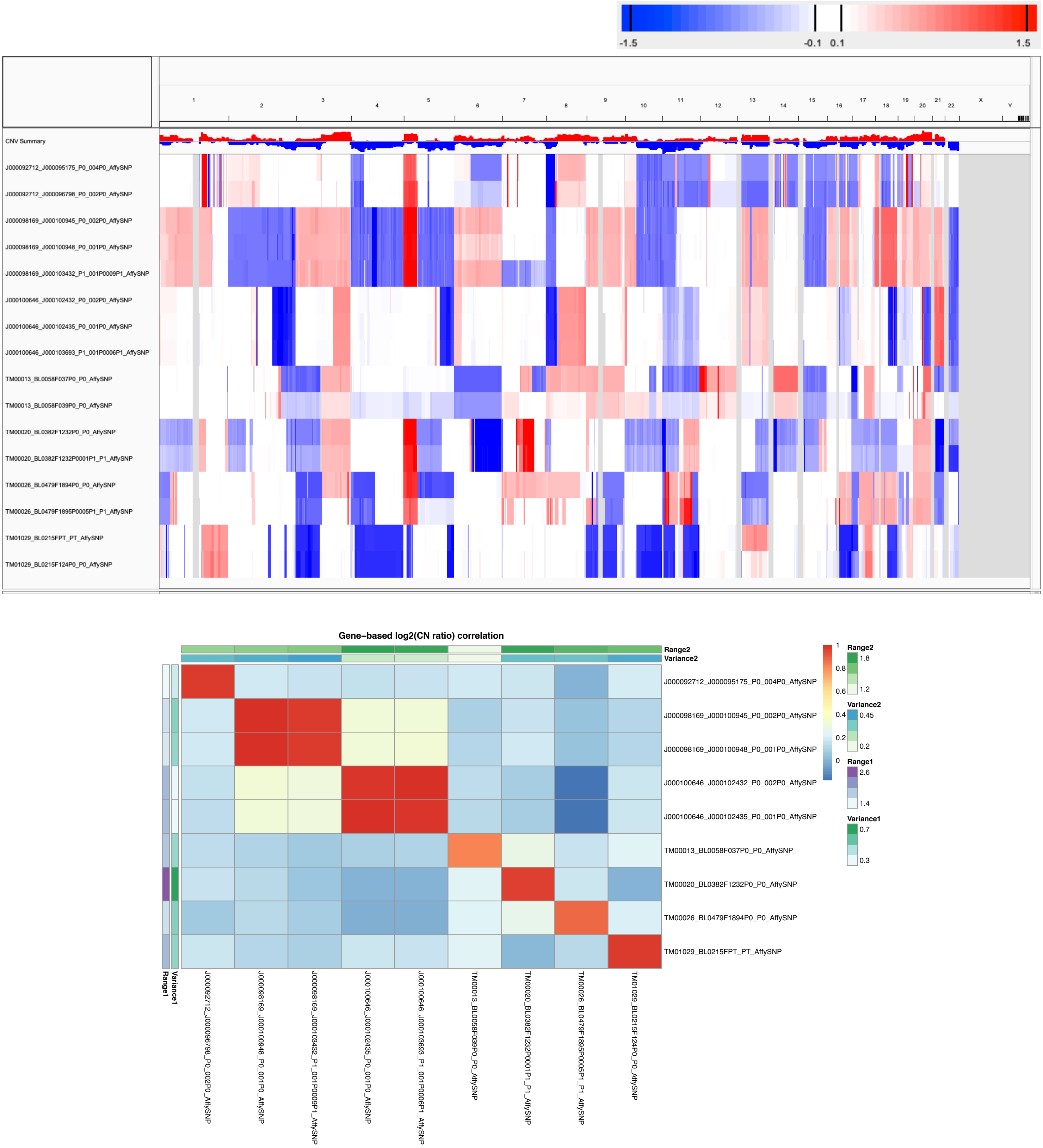

Supplementary Fig. 24: CNA profiles (IGV heatmap) and correlation heatmap of gene-based copy number (log2(CN ratio), median centered) of samples from JAX SNP array bladder cancer dataset.

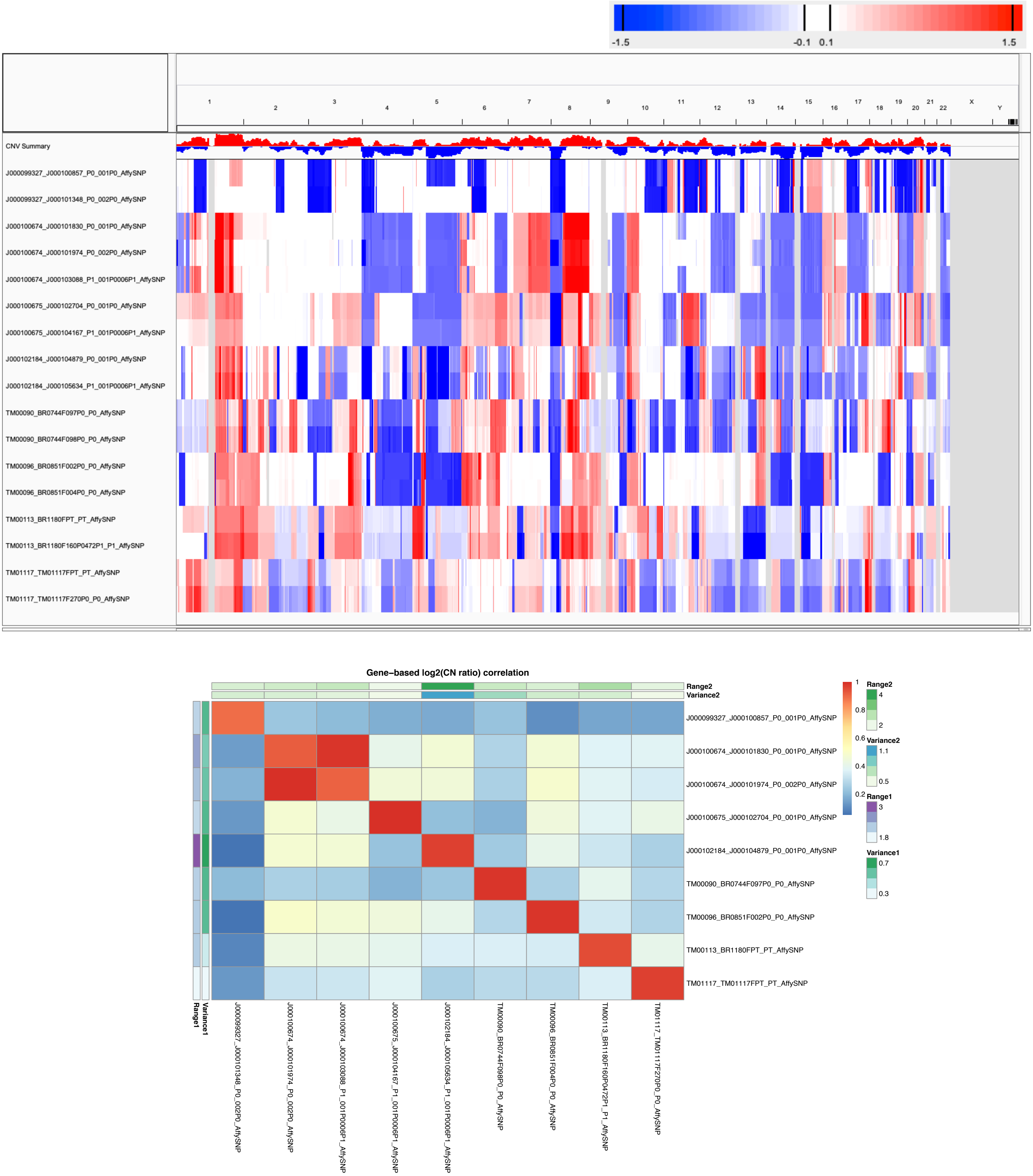

Supplementary Fig. 25: CNA profiles (IGV heatmap) and correlation heatmap of gene-based copy number (log2(CN ratio), median centered) of samples from JAX SNP array breast cancer dataset.

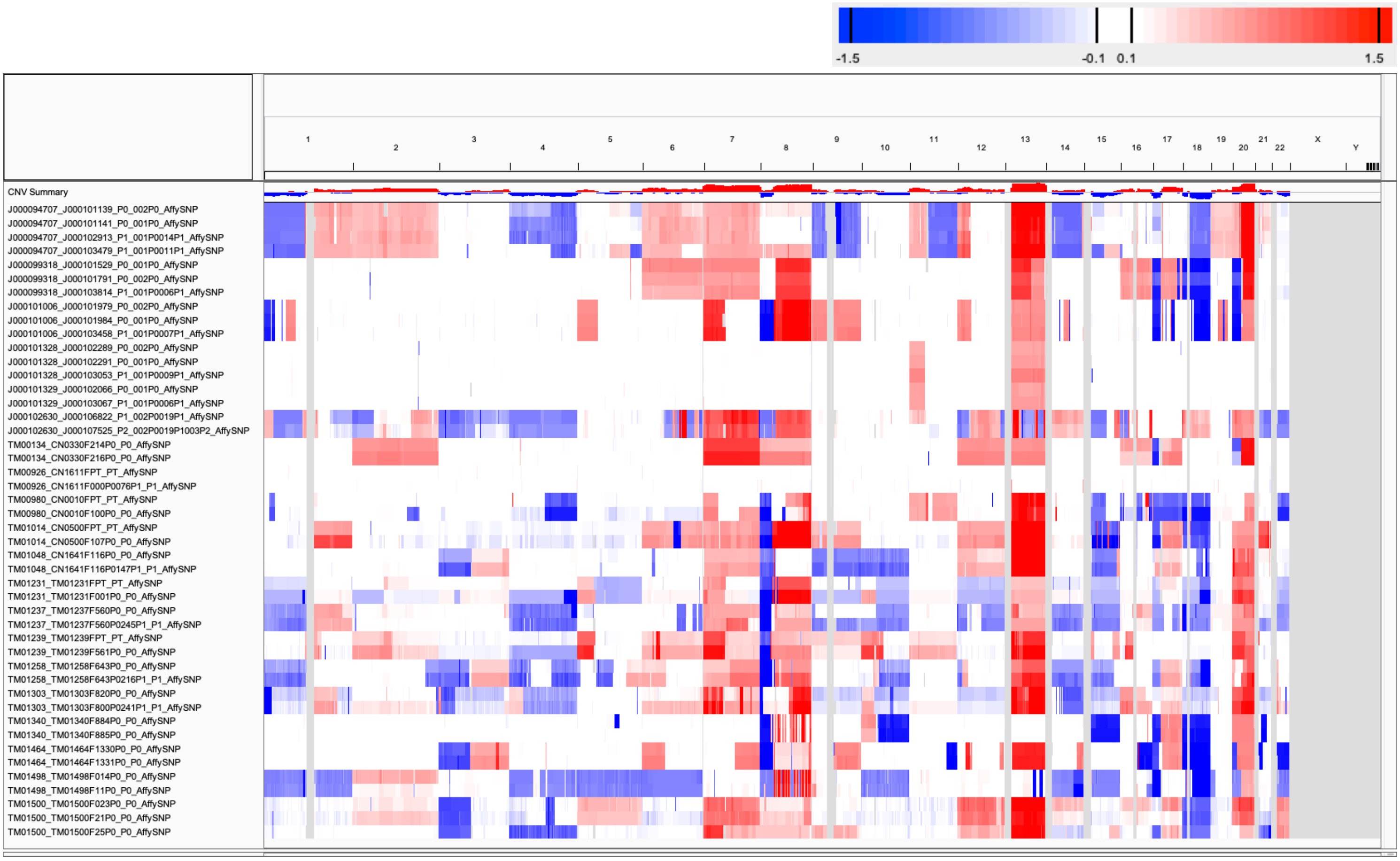

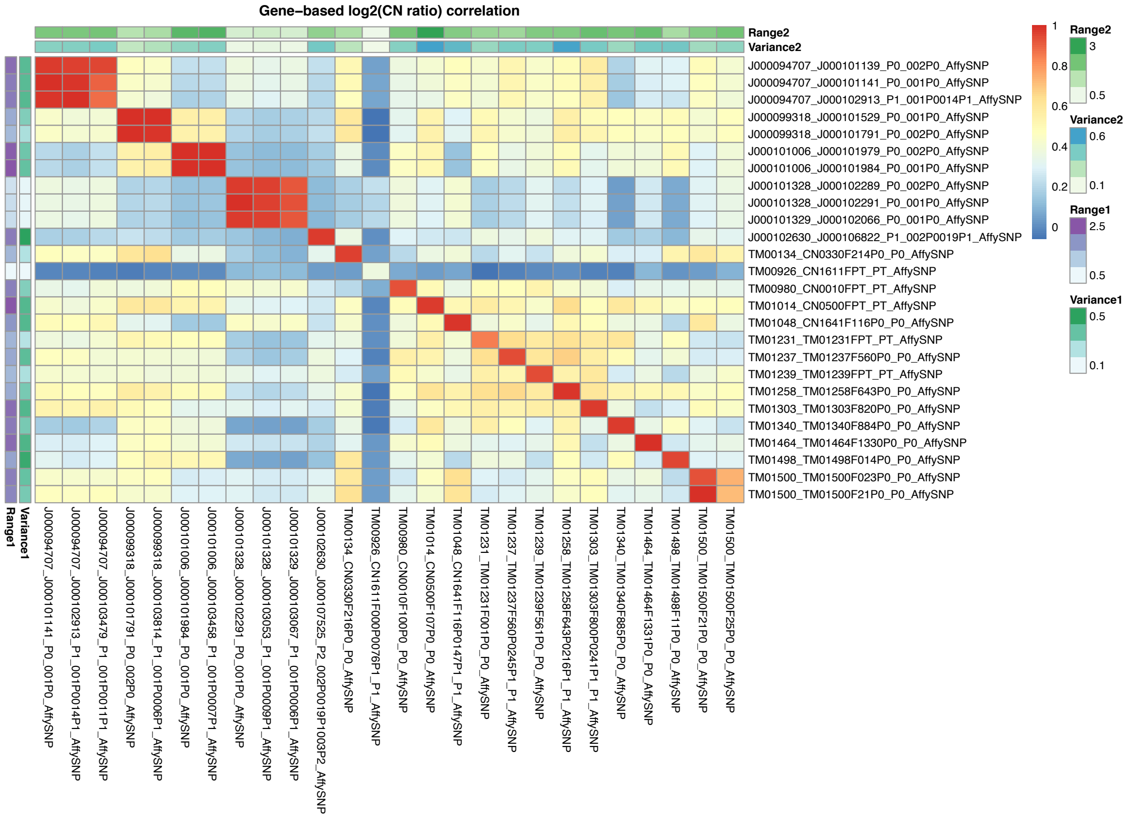

Supplementary Fig. 26: CNA profiles (IGV heatmap) and correlation heatmap of gene-based copy number (log2(CN ratio), median centered) of samples from JAX SNP array colorectal cancer dataset.

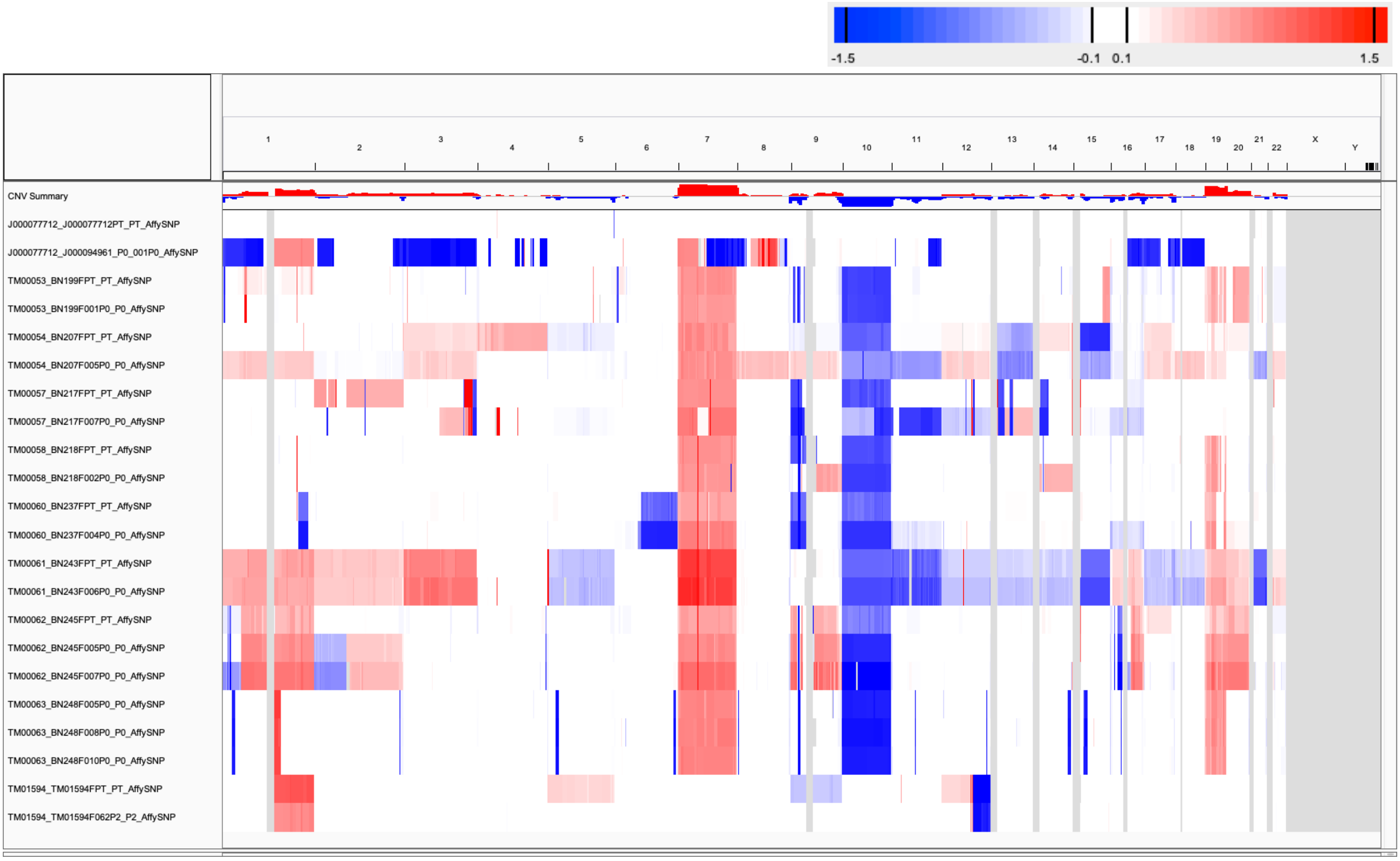

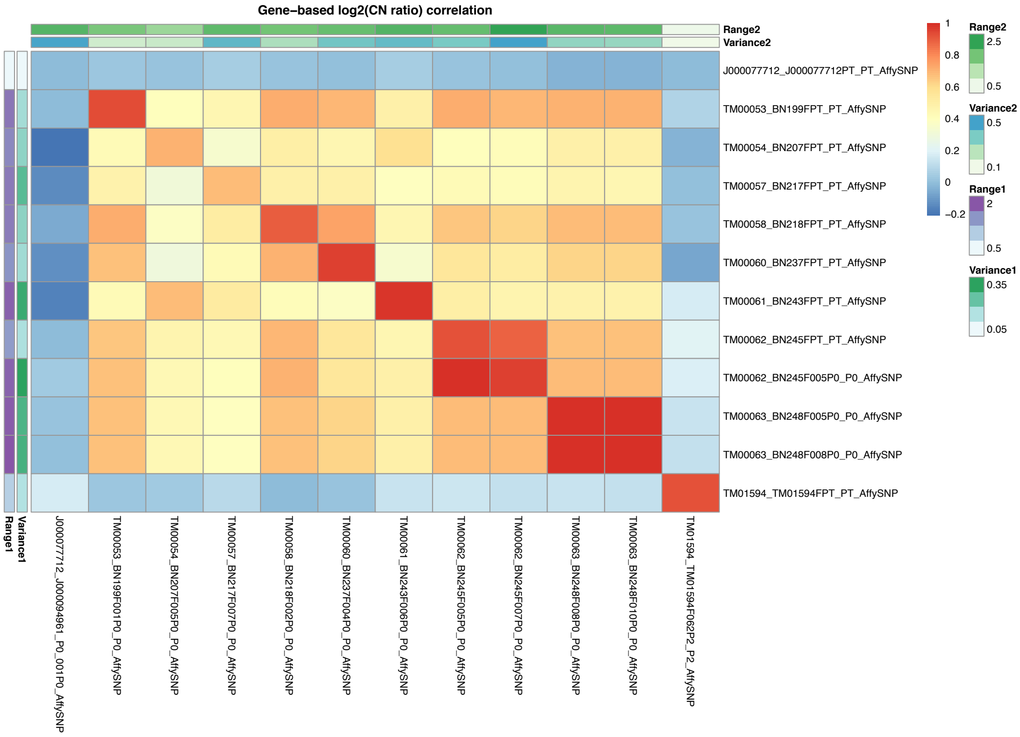

Supplementary Fig. 27: CNA profiles (IGV heatmap) and correlation heatmap of gene-based copy number (log2(CN ratio), median centered) of samples from JAX SNP array glioblastoma multiforme (GBM) dataset.

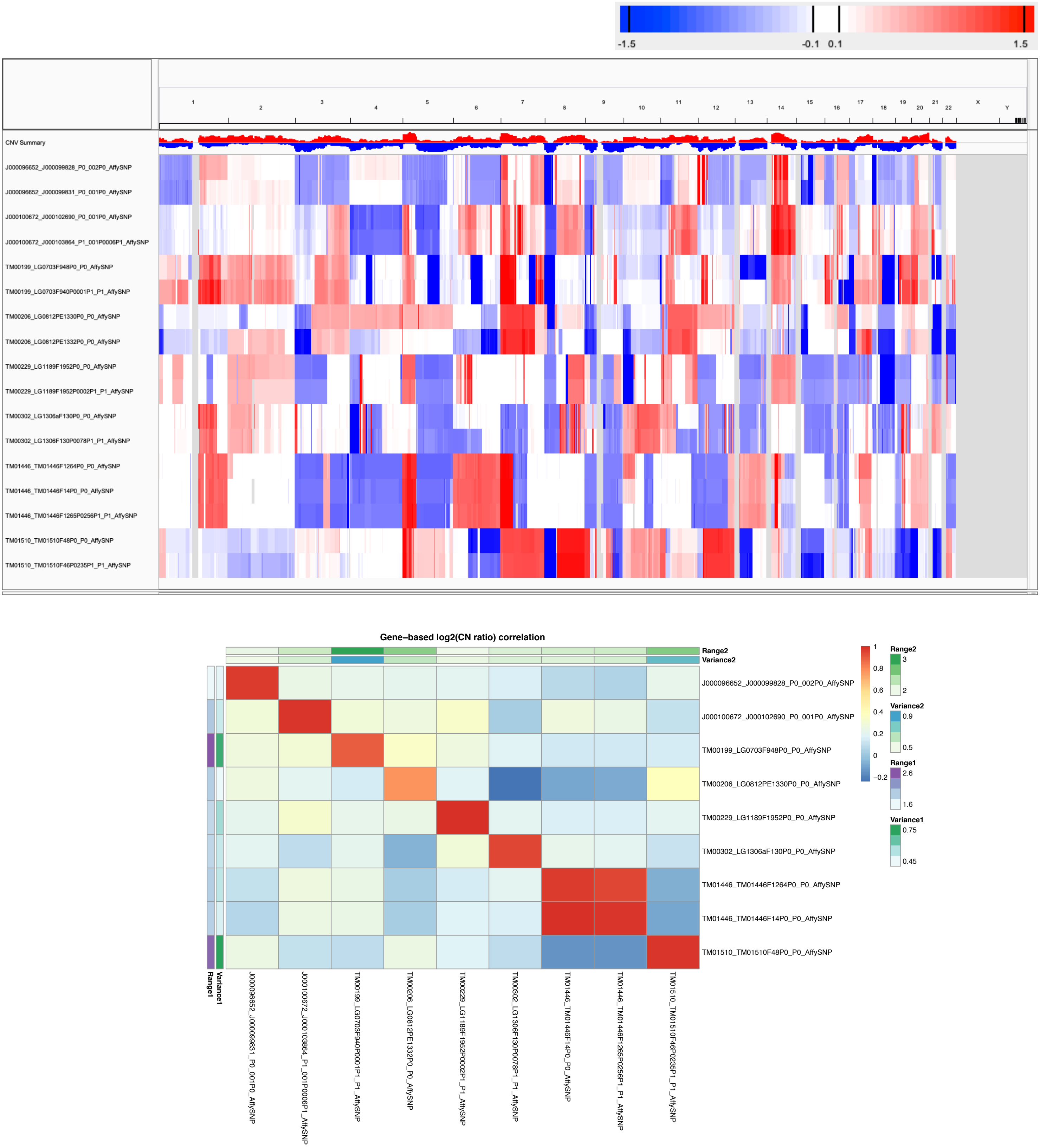

Supplementary Fig. 28: CNA profiles (IGV heatmap) and correlation heatmap of gene-based copy number (log2(CN ratio), median centered) of samples from JAX SNP array lung adenocarcinoma (LUAD) dataset.

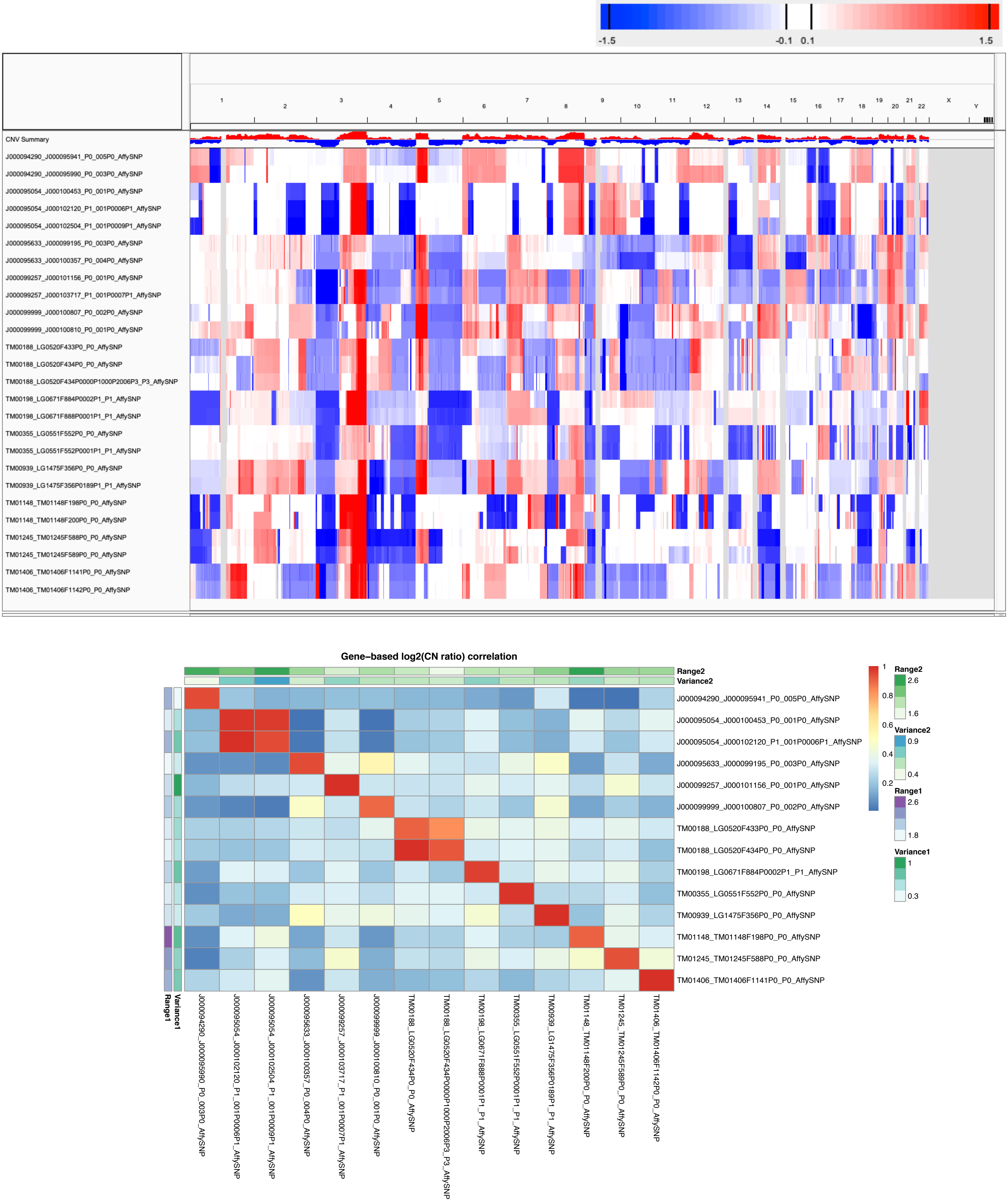

Supplementary Fig. 29: CNA profiles (IGV heatmap) and correlation heatmap of gene-based copy number (log2(CN ratio), median centered) of samples from JAX SNP array lung squamous cell carcinoma (LUSC) dataset.

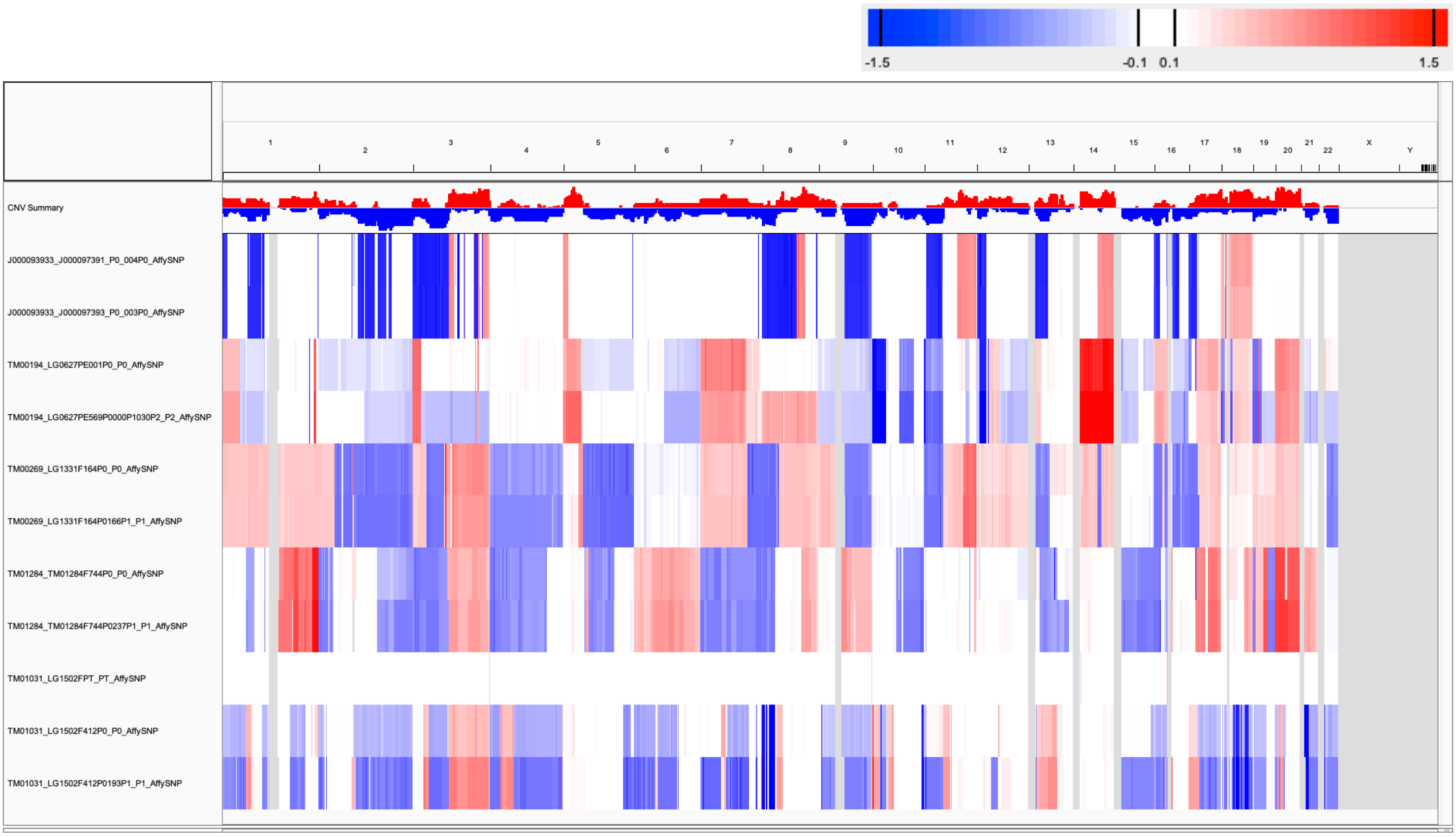

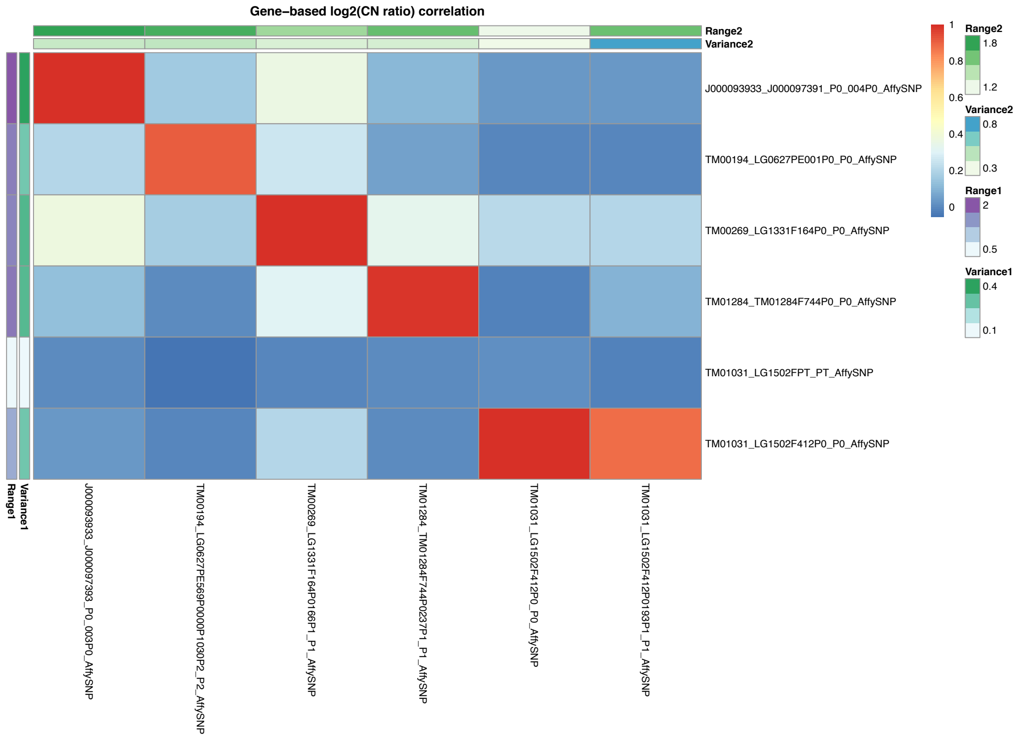

Supplementary Fig. 30: CNA profiles (IGV heatmap) and correlation heatmap of gene-based copy number (log2(CN ratio), median centered) of samples from JAX SNP array other lung cancer subtypes dataset.

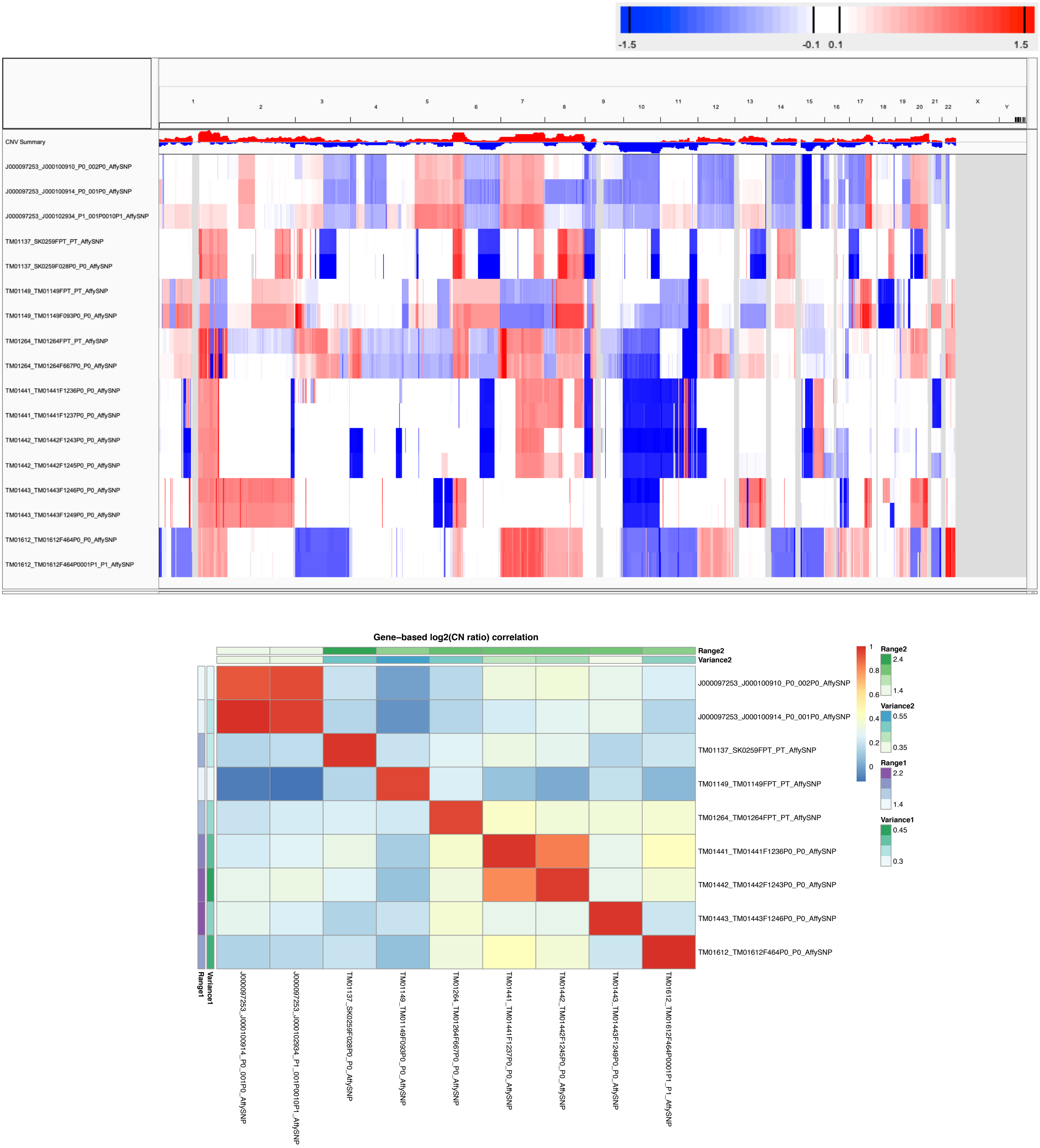

Supplementary Fig. 31: CNA profiles (IGV heatmap) and correlation heatmap of gene-based copy number (log2(CN ratio), median centered) of samples from JAX SNP array skin melanoma dataset.

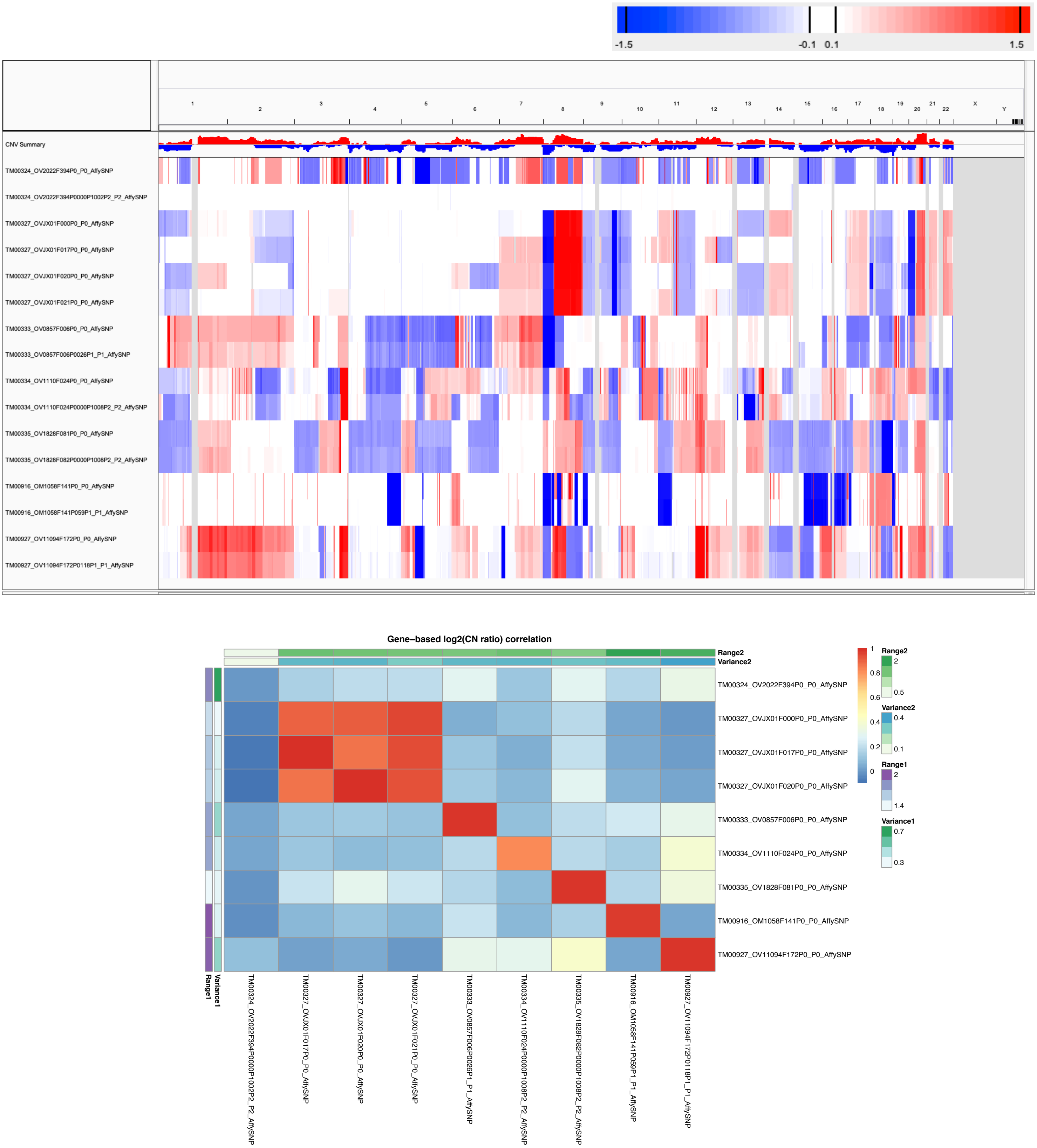

Supplementary Fig. 32: CNA profiles (IGV heatmap) and correlation heatmap of gene-based copy number (log2(CN ratio), median centered) of samples from JAX SNP array ovarian cancer dataset.

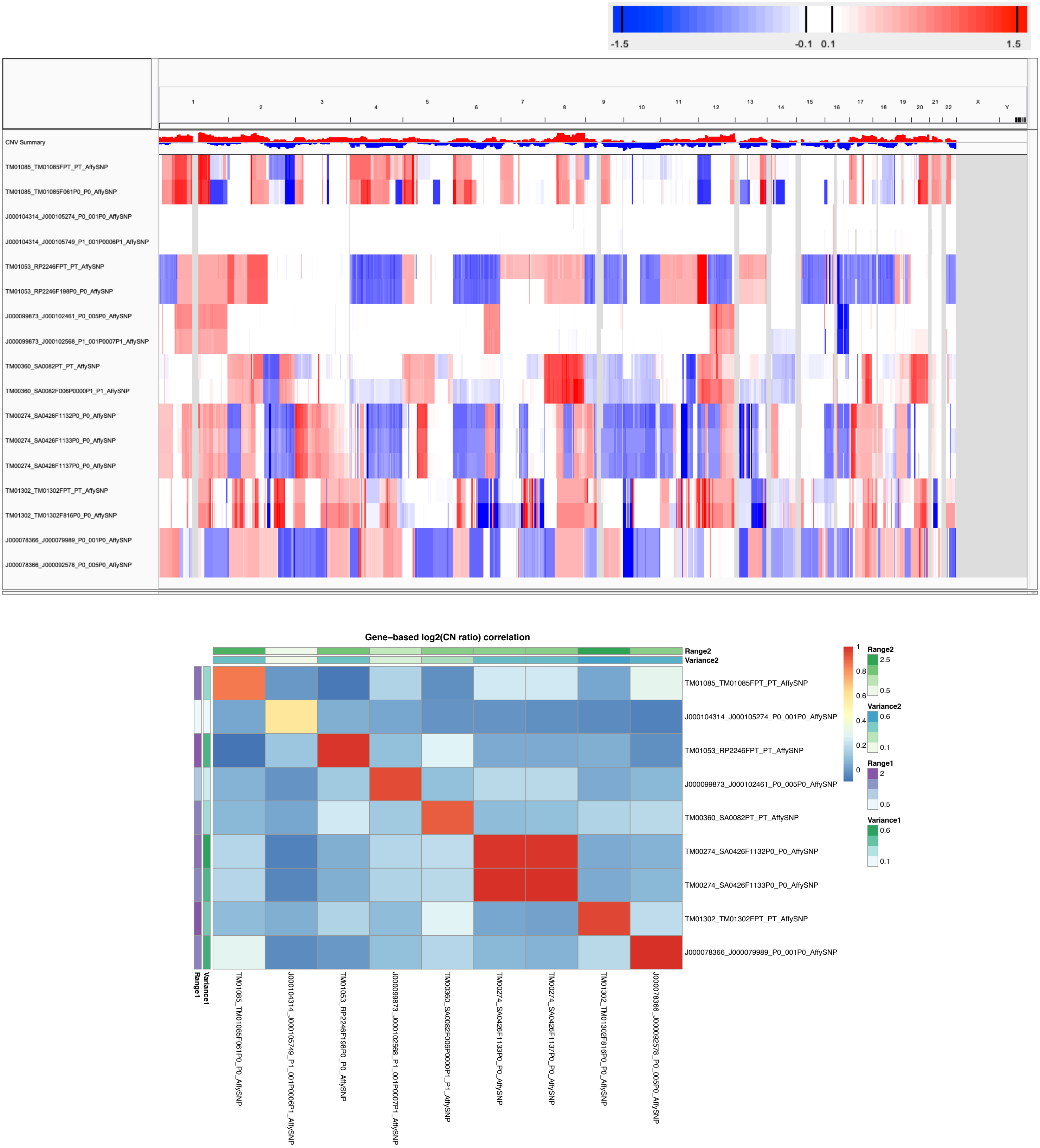

Supplementary Fig. 33: CNA profiles (IGV heatmap) and correlation heatmap of gene-based copy number (log2(CN ratio), median centered) of samples from JAX SNP array sarcoma dataset.

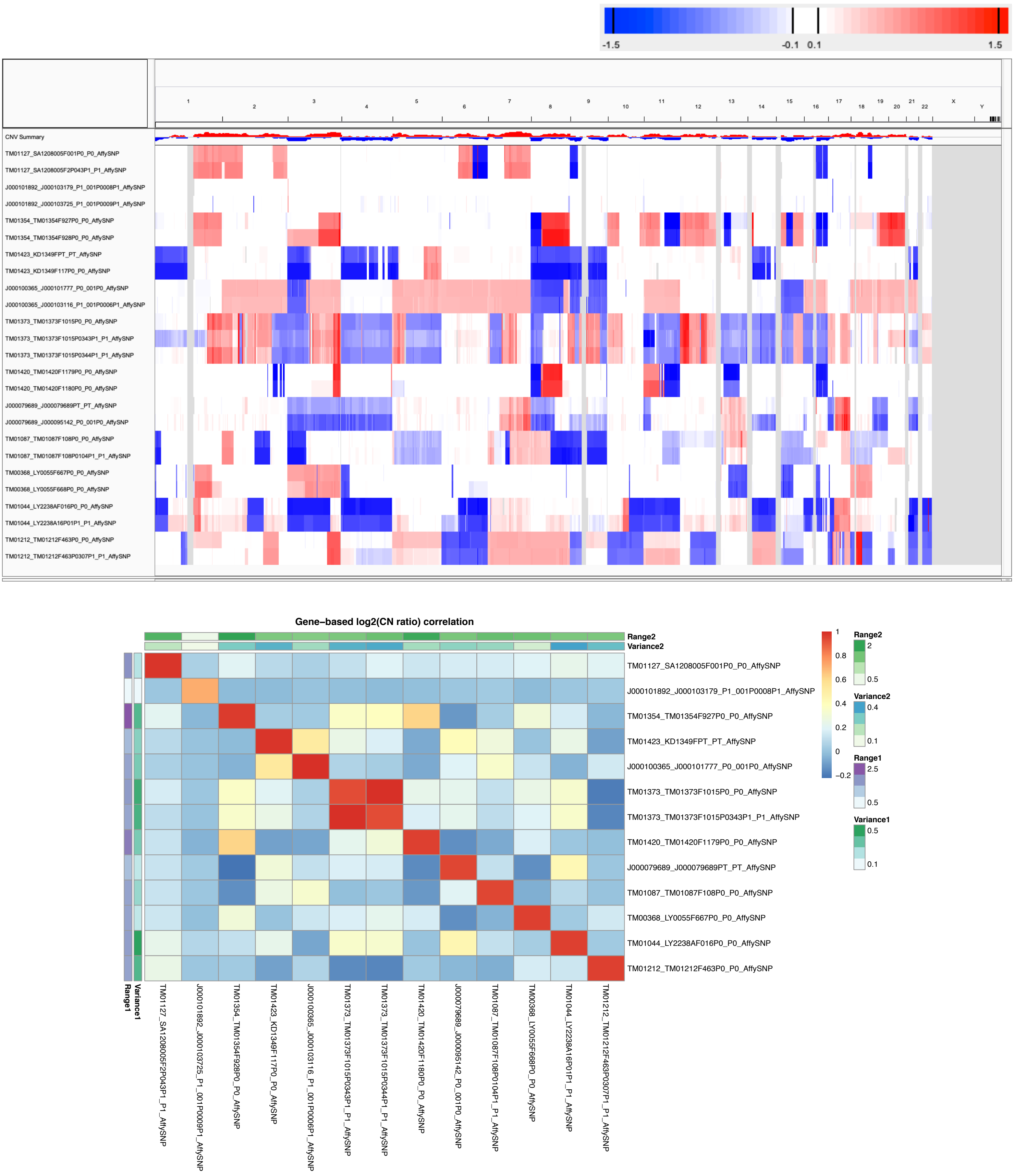

Supplementary Fig. 34: CNA profiles (IGV heatmap) and correlation heatmap of gene-based copy number (log2(CN ratio), median centered) of samples from JAX SNP array other cancers dataset.

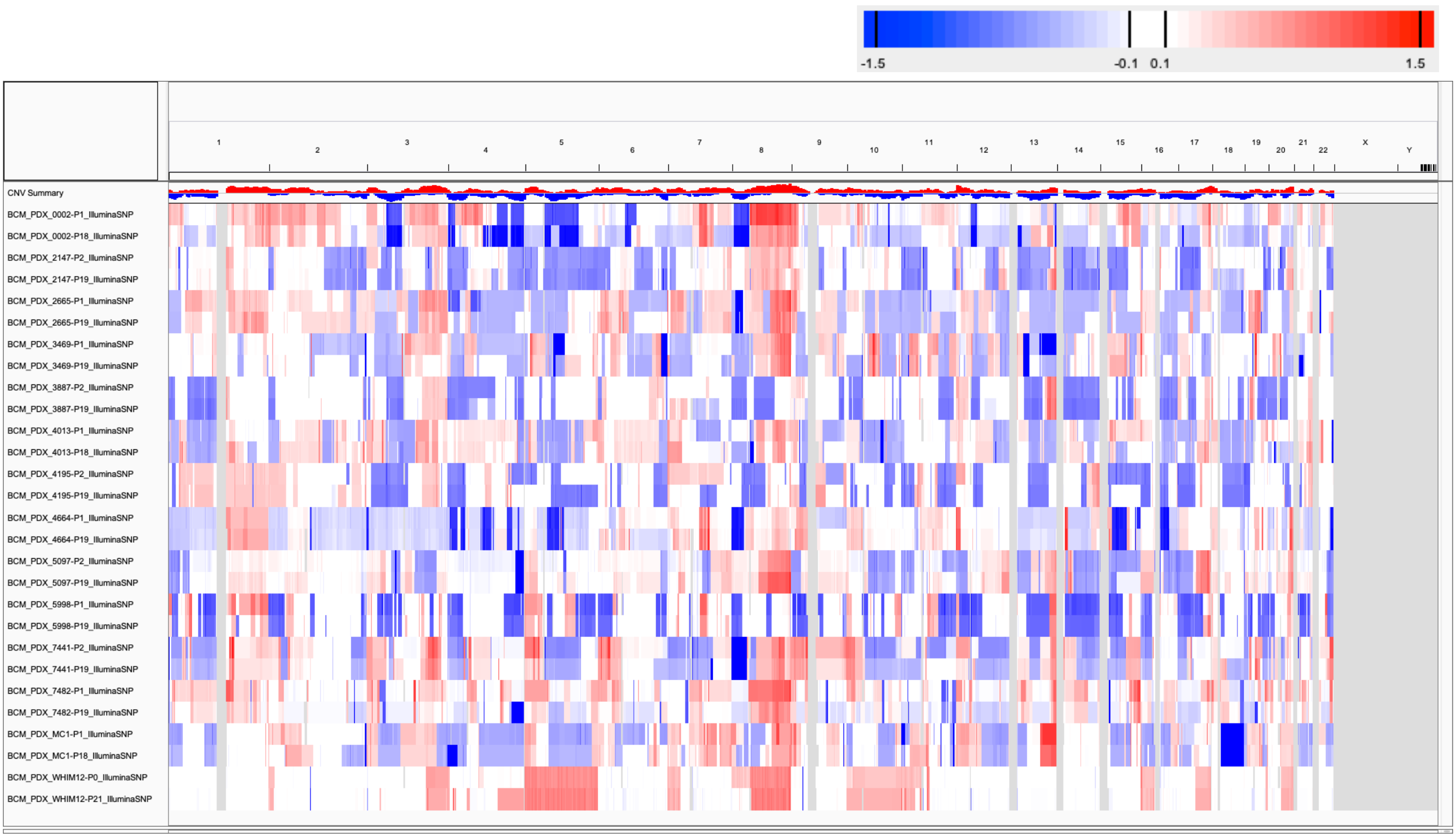

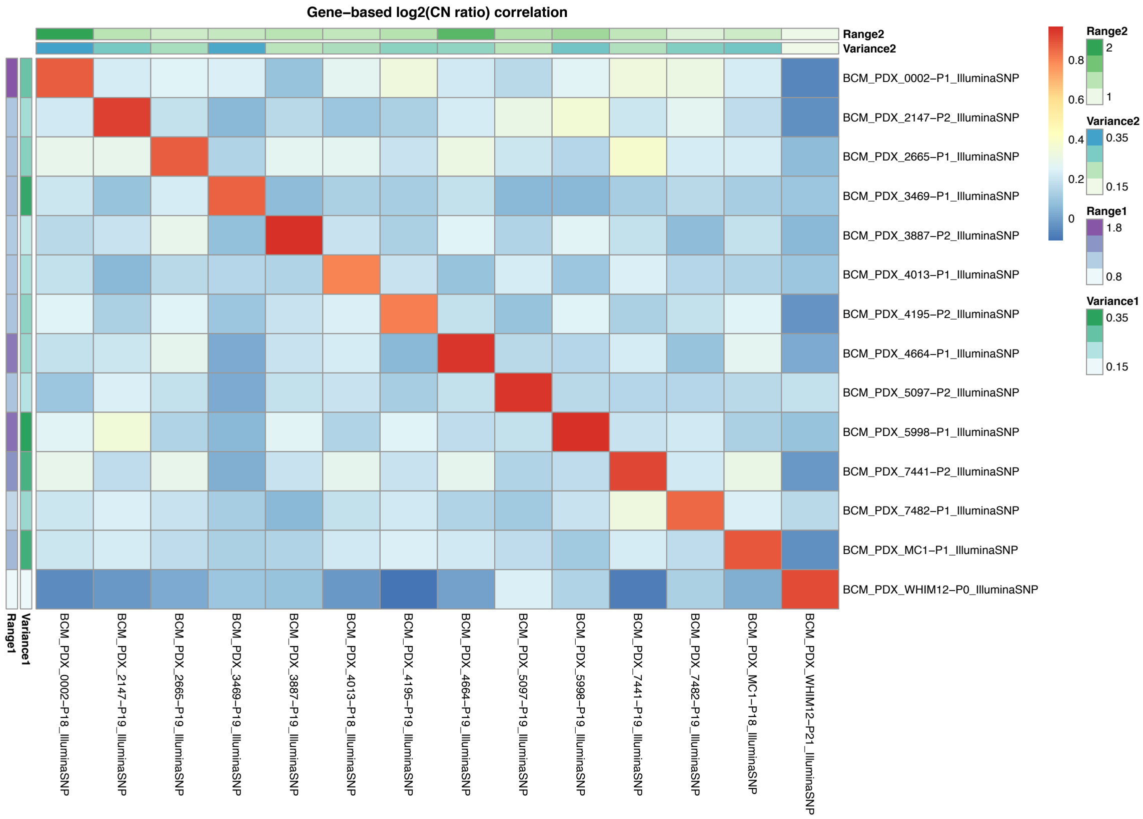

Supplementary Fig. 35: CNA profiles (IGV heatmap) and correlation heatmap of gene-based copy number (log2(CN ratio), median centered) of samples from BCM SNP array breast cancer dataset.

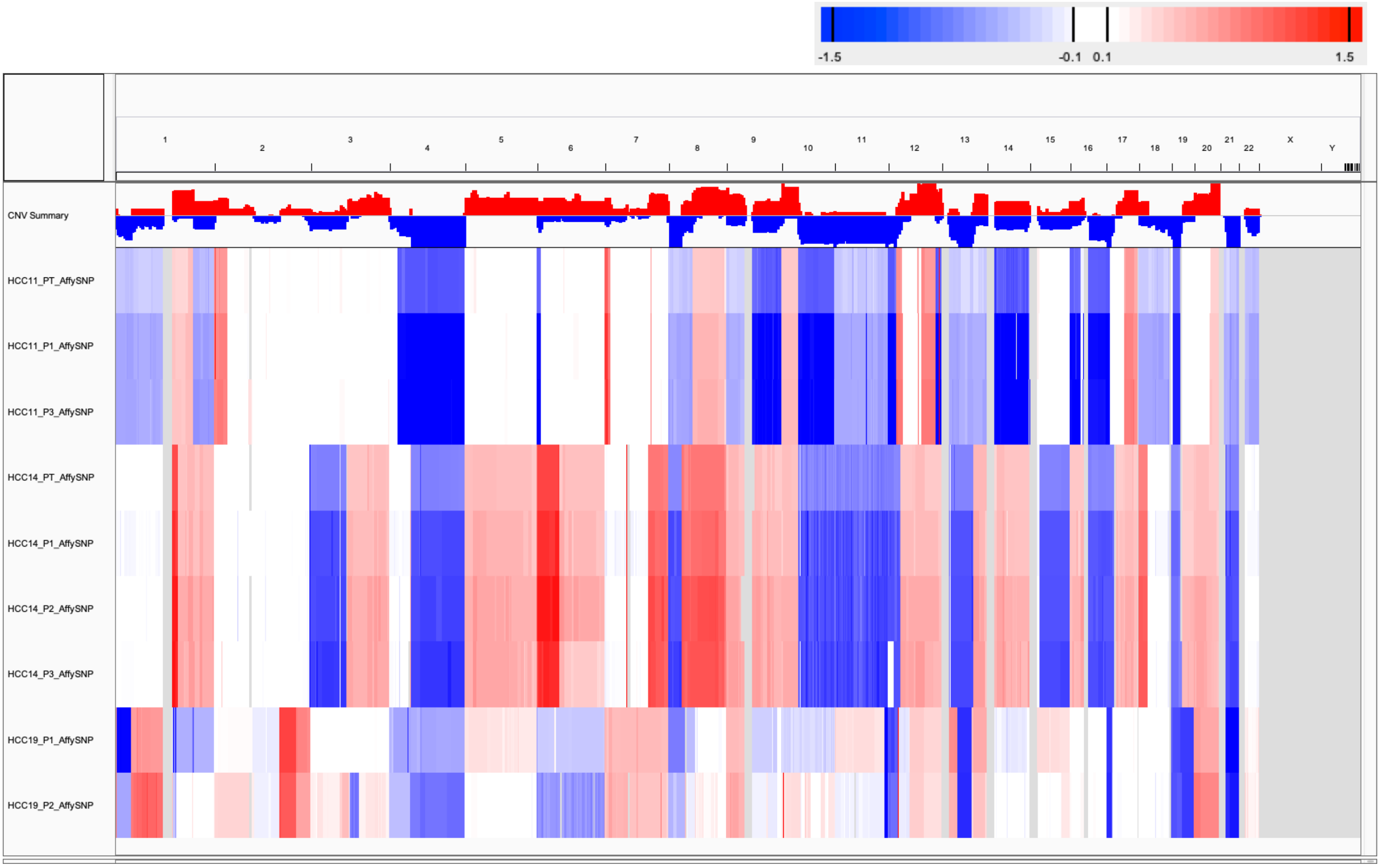

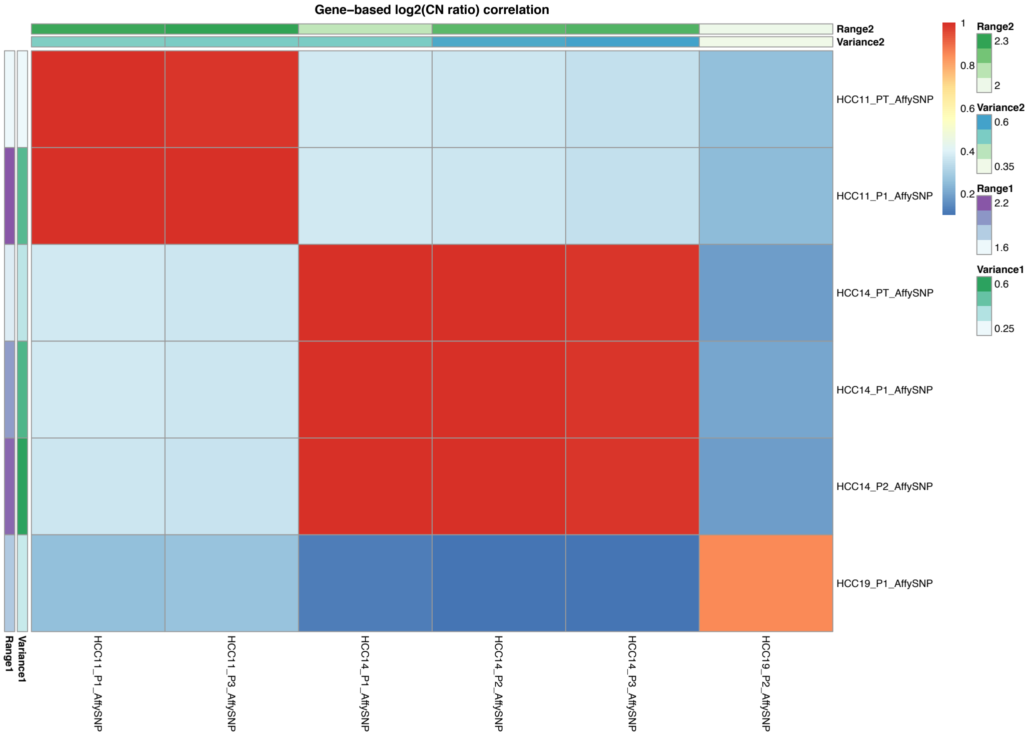

Supplementary Fig. 36: CNA profiles (IGV heatmap) and correlation heatmap of gene-based copy number (log2(CN ratio), median centered) of samples from SIBS SNP array hepatocellular carcinoma (HCC) dataset.

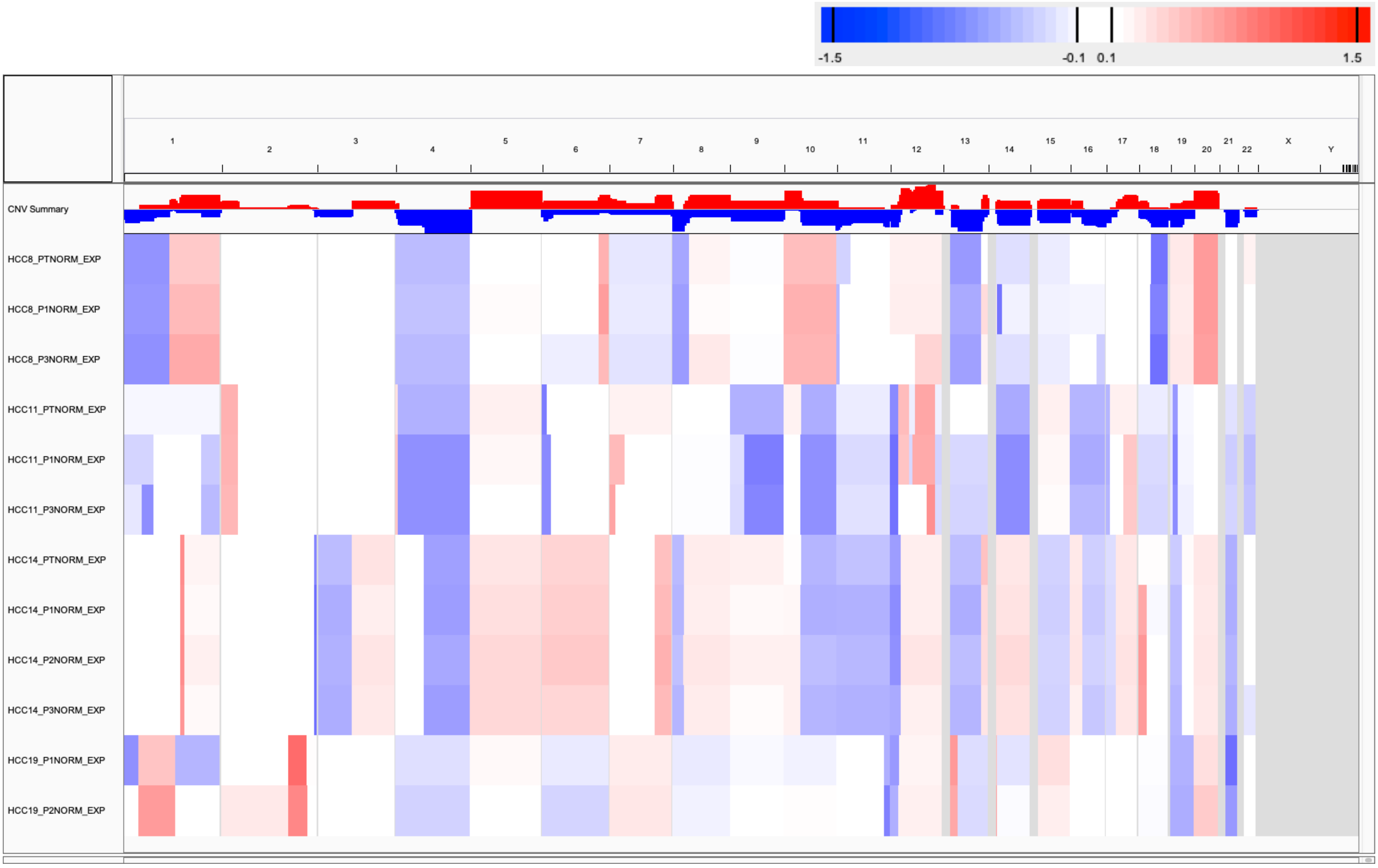

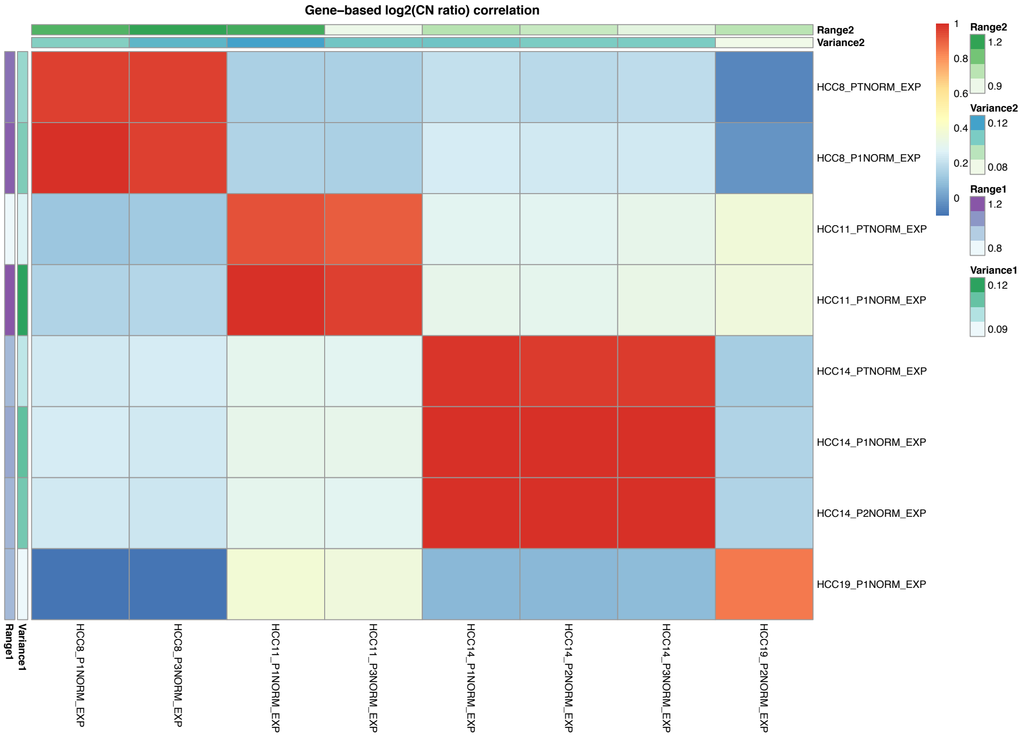

Supplementary Fig. 37: CNA profiles (IGV heatmap) and correlation heatmap of gene-based copy number (log2(CN ratio), median centered) of samples from SIBS gene expression array (normalized by median expression of normal liver tissue samples) hepatocellular carcinoma (HCC) dataset.

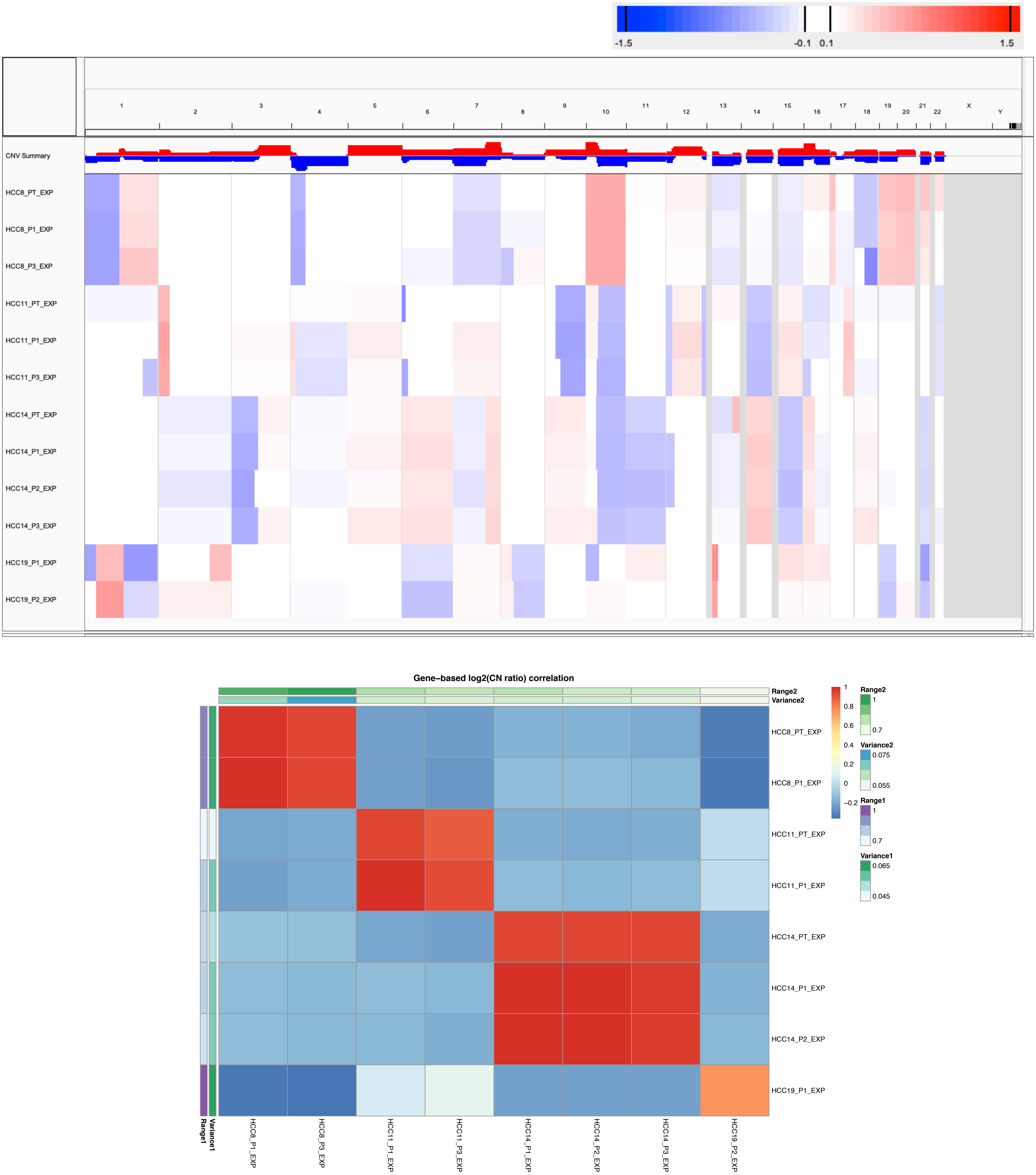

Supplementary Fig. 38: CNA profiles (IGV heatmap) and correlation heatmap of gene-based copy number (log2(CN ratio), median centered) of samples from SIBS gene expression array (normalized by median expression of tumor samples of the same dataset) hepatocellular carcinoma (HCC) dataset.

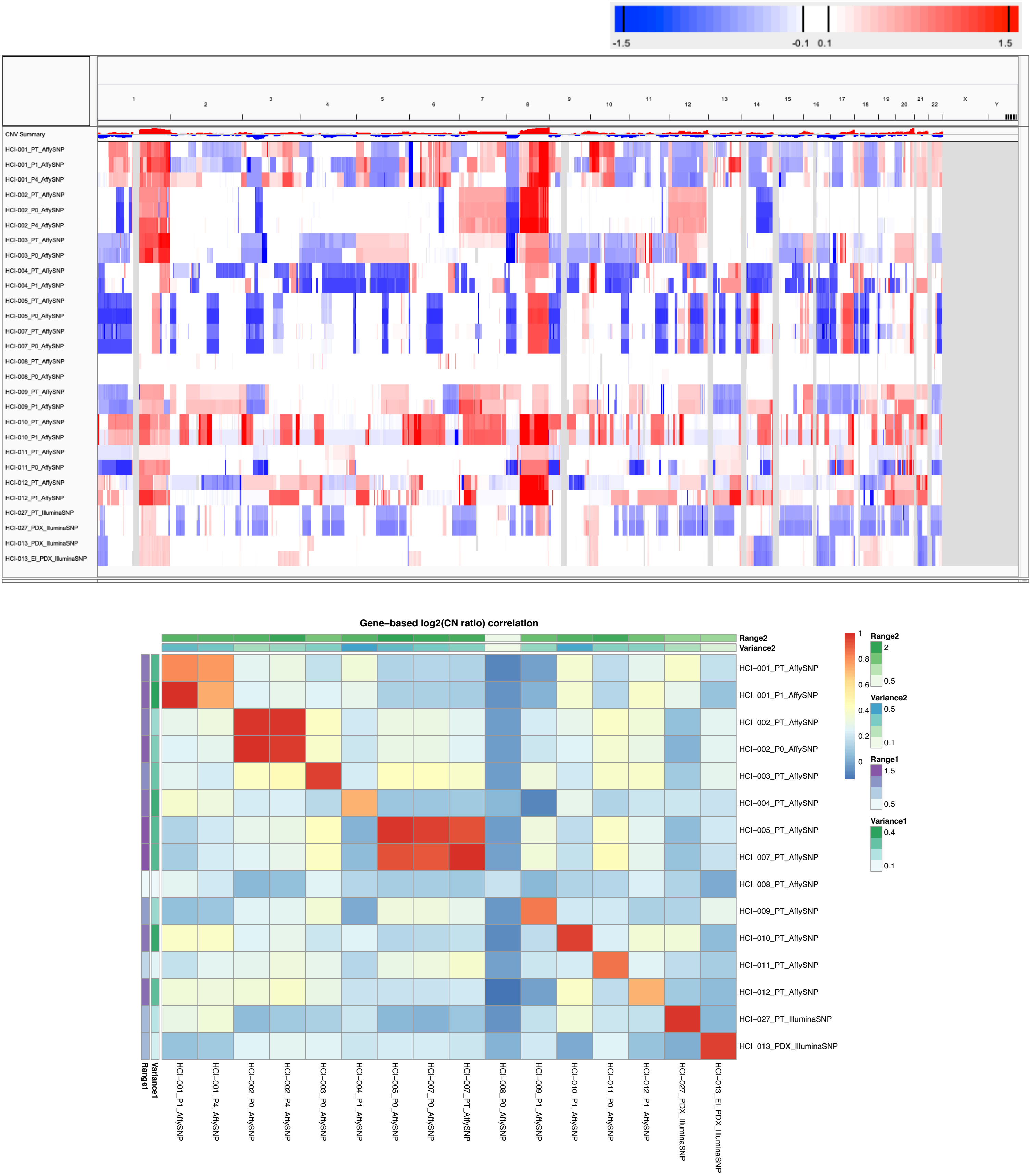

Supplementary Fig. 39: CNA profiles (IGV heatmap) and correlation heatmap of gene-based copy number (log2(CN ratio), median centered) of samples from HCI SNP array breast cancer dataset.

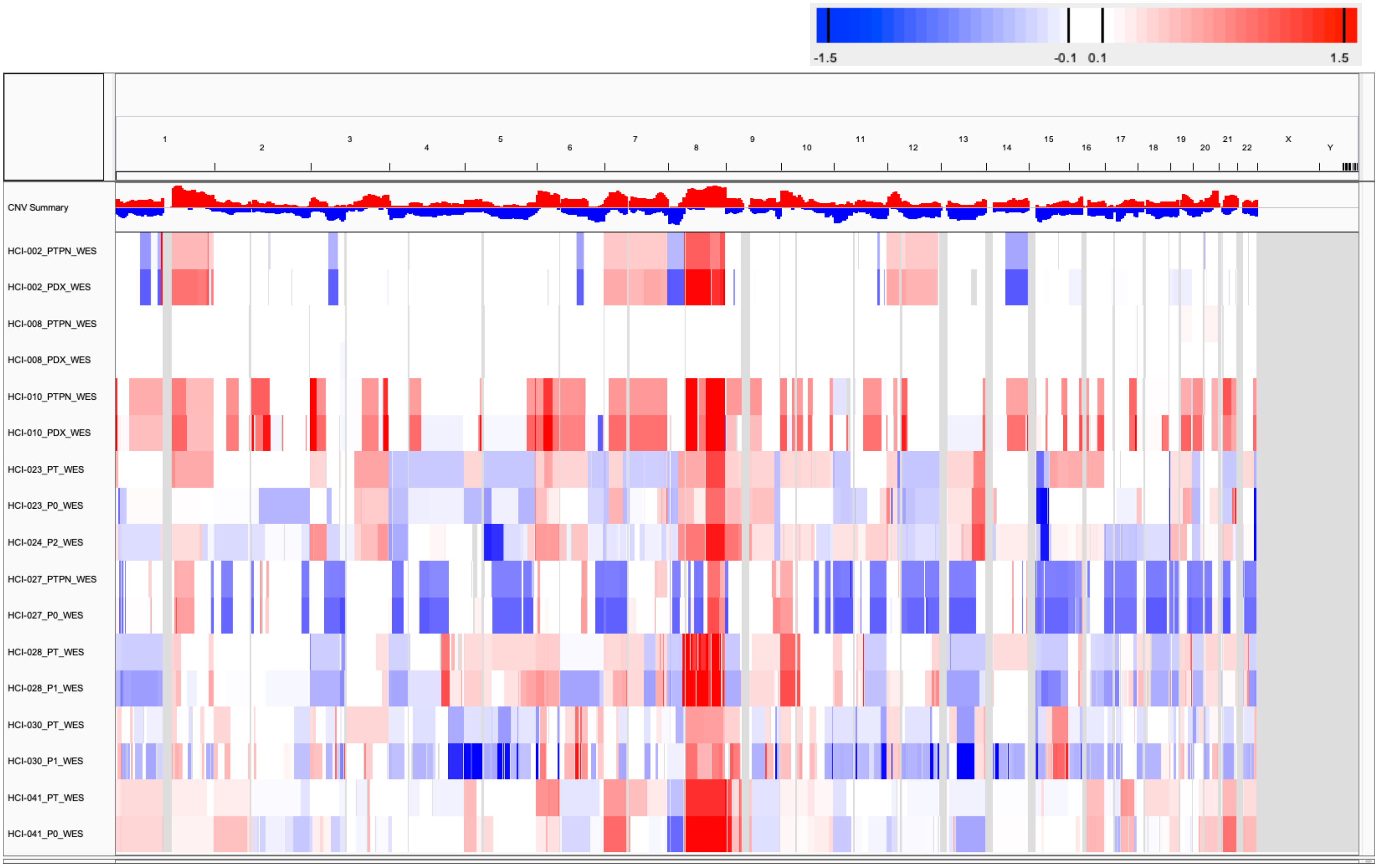

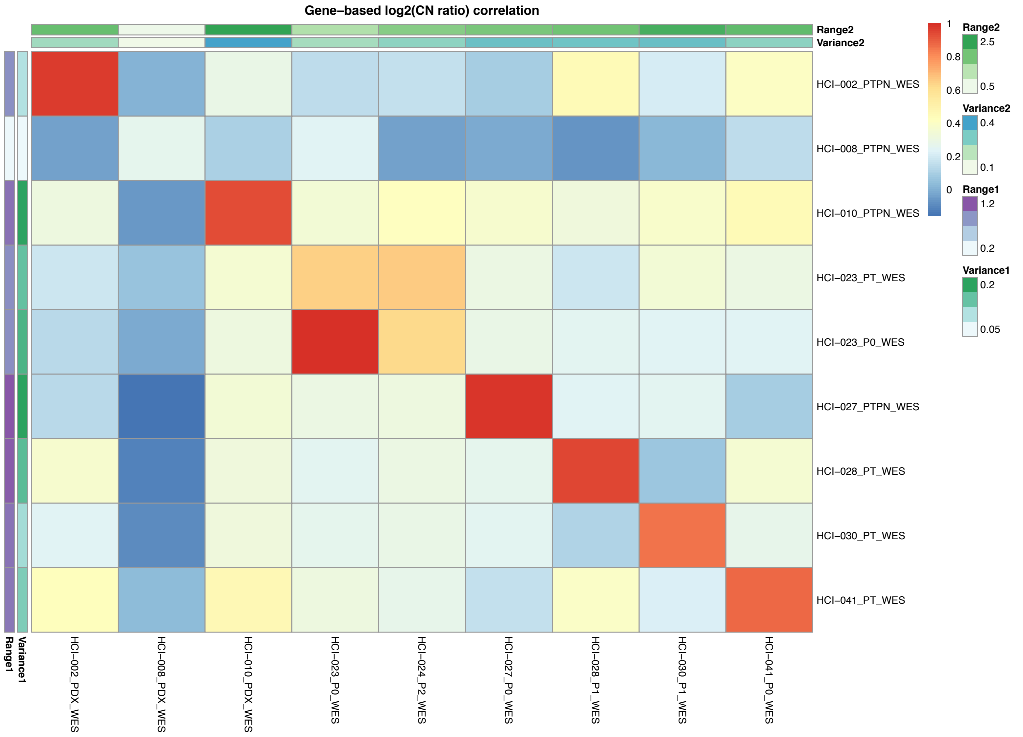

Supplementary Fig. 40: CNA profiles (IGV heatmap) and correlation heatmap of gene-based copy number (log2(CN ratio), median centered) of samples from HCI WES breast cancer dataset.

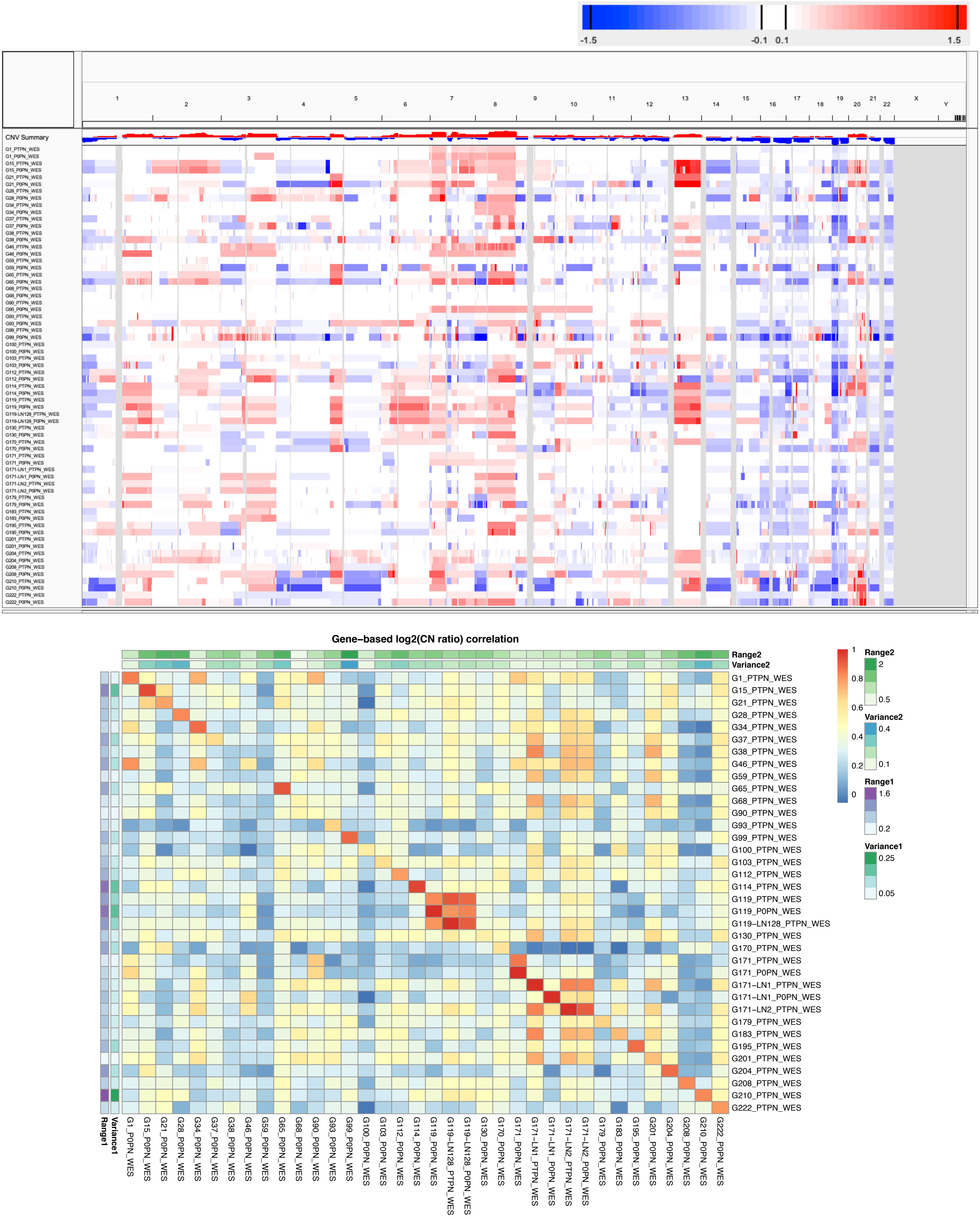

Supplementary Fig. 41: CNA profiles (IGV heatmap) and correlation heatmap of gene-based copy number (log2(CN ratio), median centered) of samples from SNU-JAX WES gastric cancer dataset.

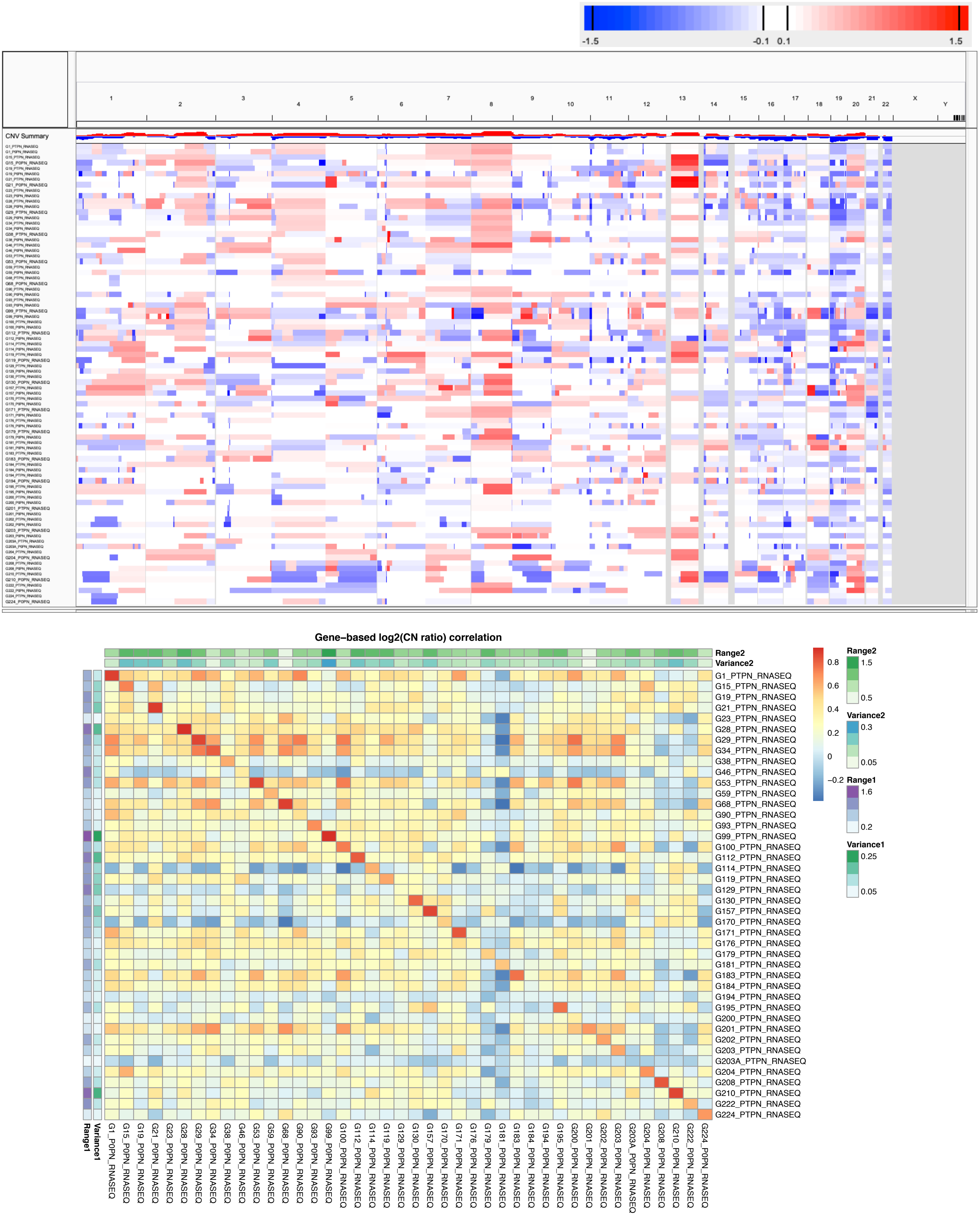

Supplementary Fig. 42: CNA profiles (IGV heatmap) and correlation heatmap of gene-based copy number (log2(CN ratio), median centered) of samples from SNU-JAX RNA-Seq (normalized by median expression of normal gastric tissue samples from the same patients) gastric cancer dataset.

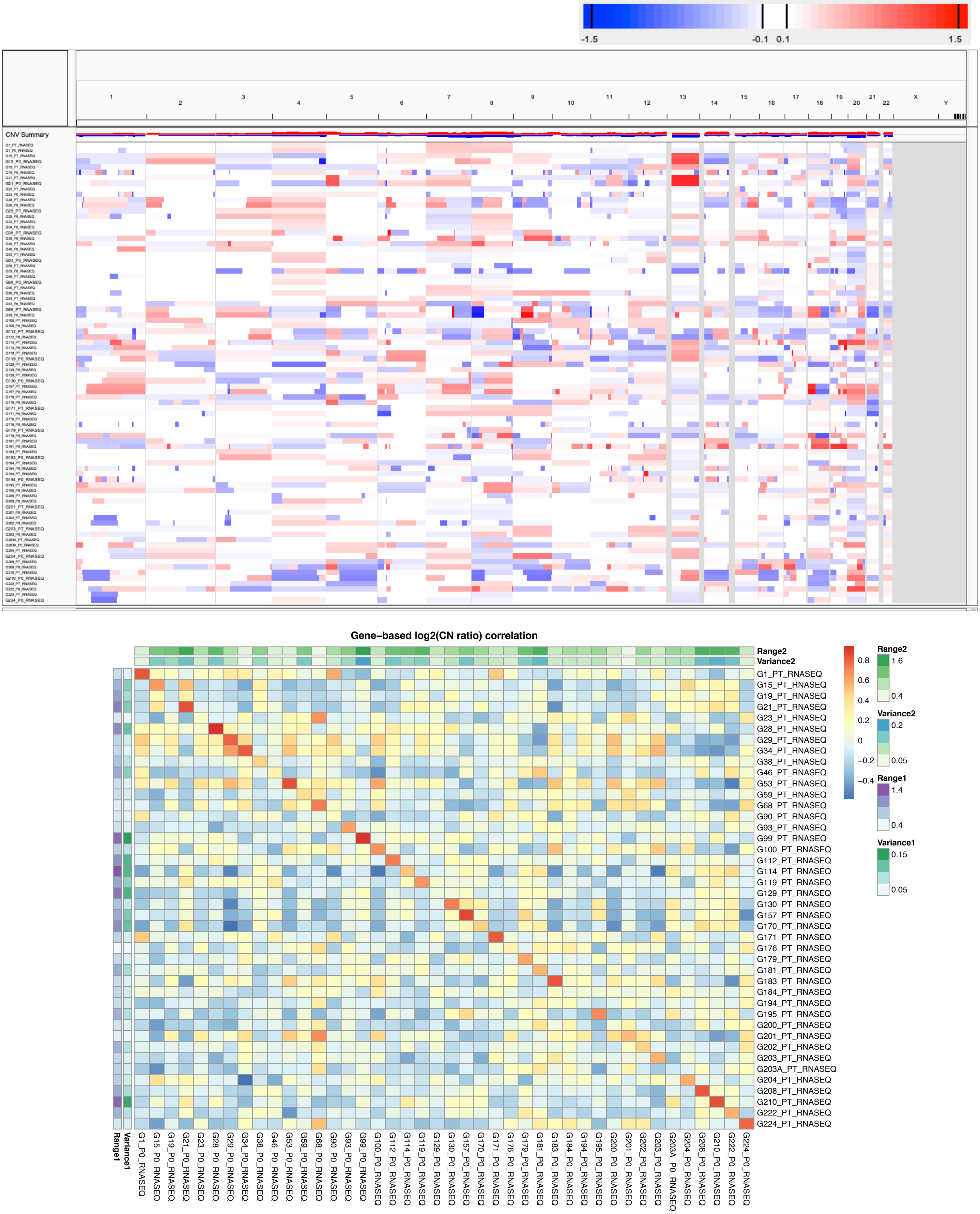

Supplementary Fig. 43: CNA profiles (IGV heatmap) and correlation heatmap of gene-based copy number (log2(CN ratio), median centered) of samples from SNU-JAX RNA-Seq (normalized by median expression of tumor samples of the same dataset) gastric cancer dataset.

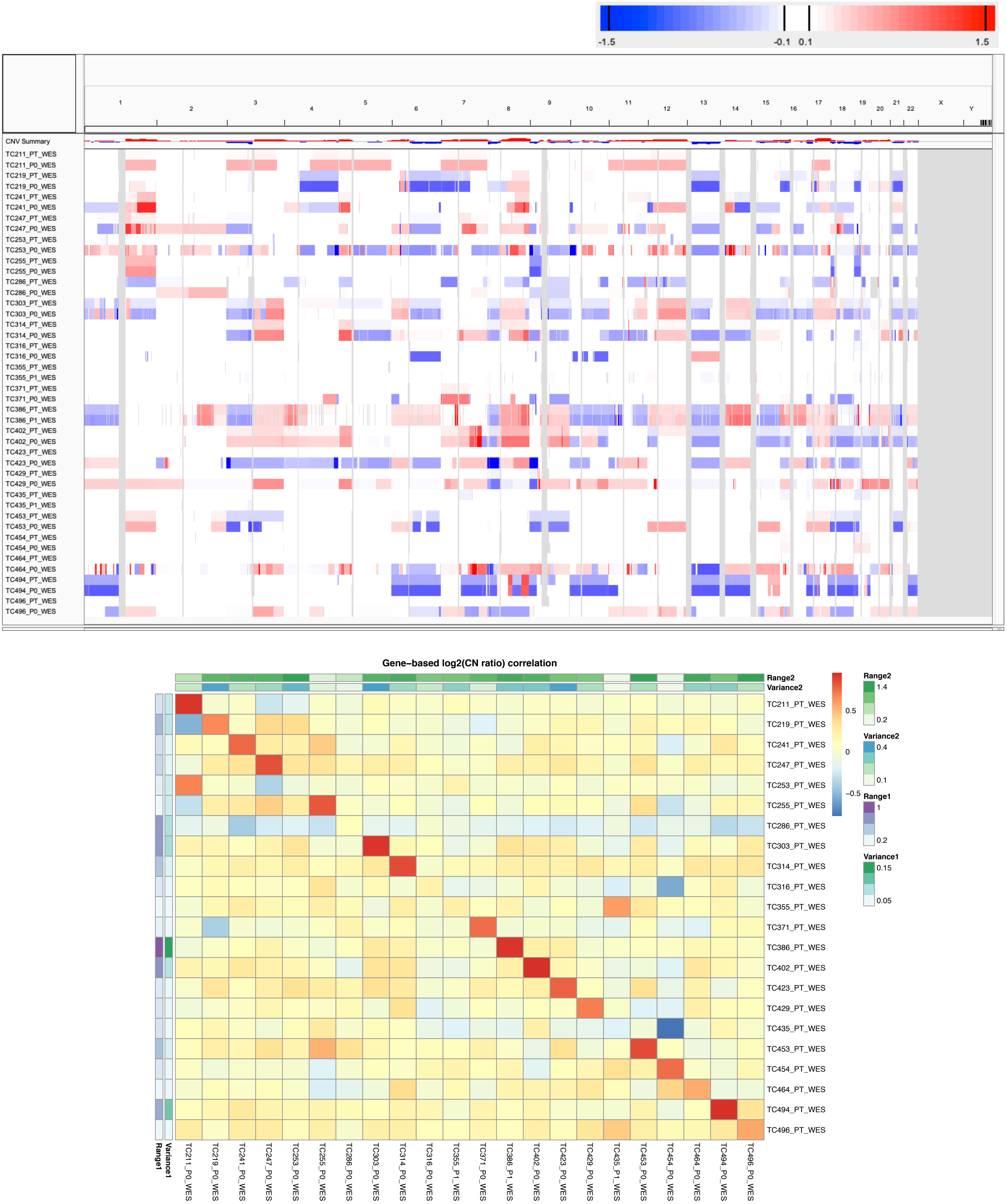

Supplementary Fig. 44: CNA profiles (IGV heatmap) and correlation heatmap of gene-based copy number (log2(CN ratio), median centered) of samples from MDACC WES lung adenocarcinoma (LUAD) dataset.

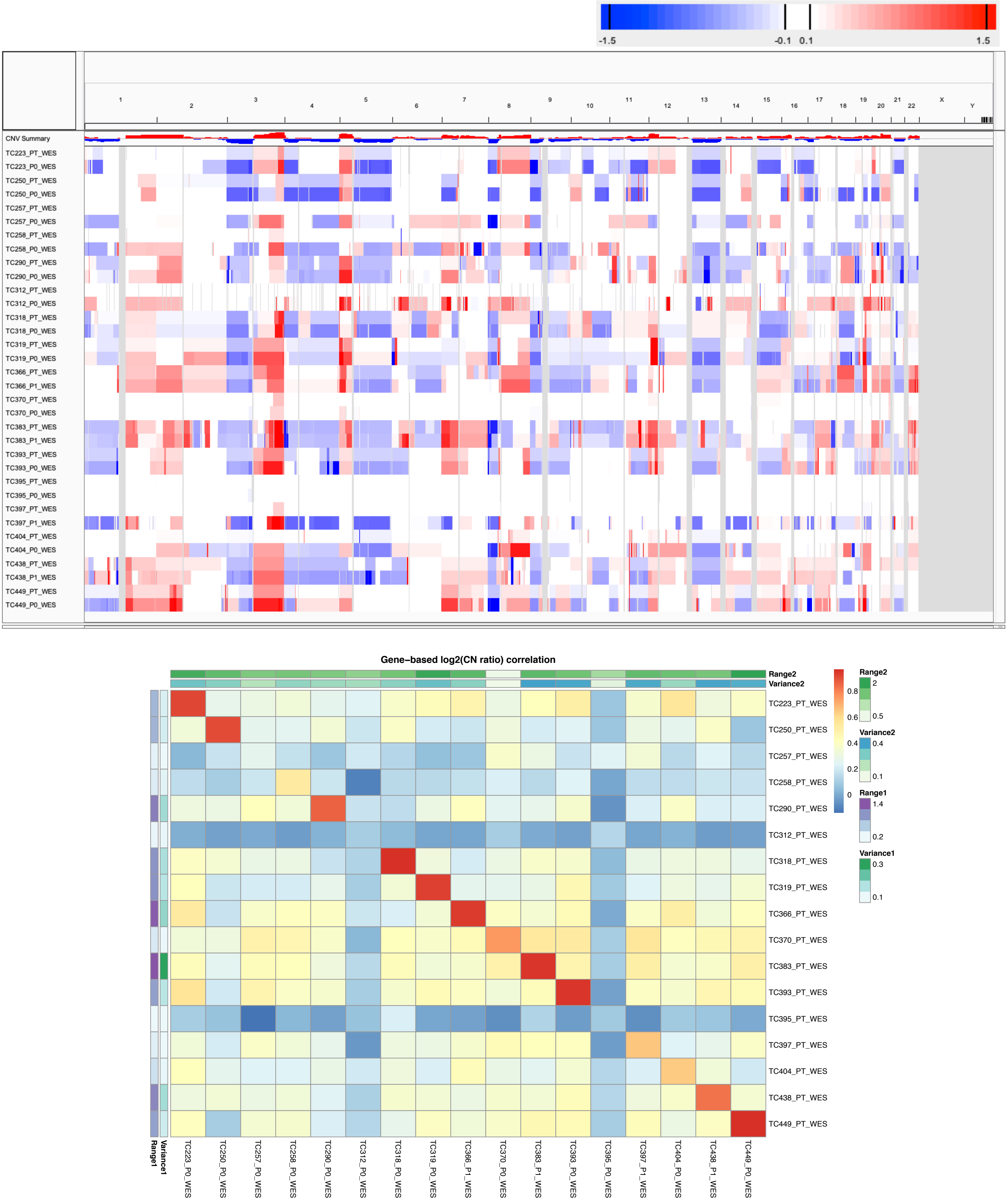

Supplementary Fig. 45: CNA profiles (IGV heatmap) and correlation heatmap of gene-based copy number (log2(CN ratio), median centered) of samples from MDACC WES lung squamous cell carcinoma (LUSC) dataset.

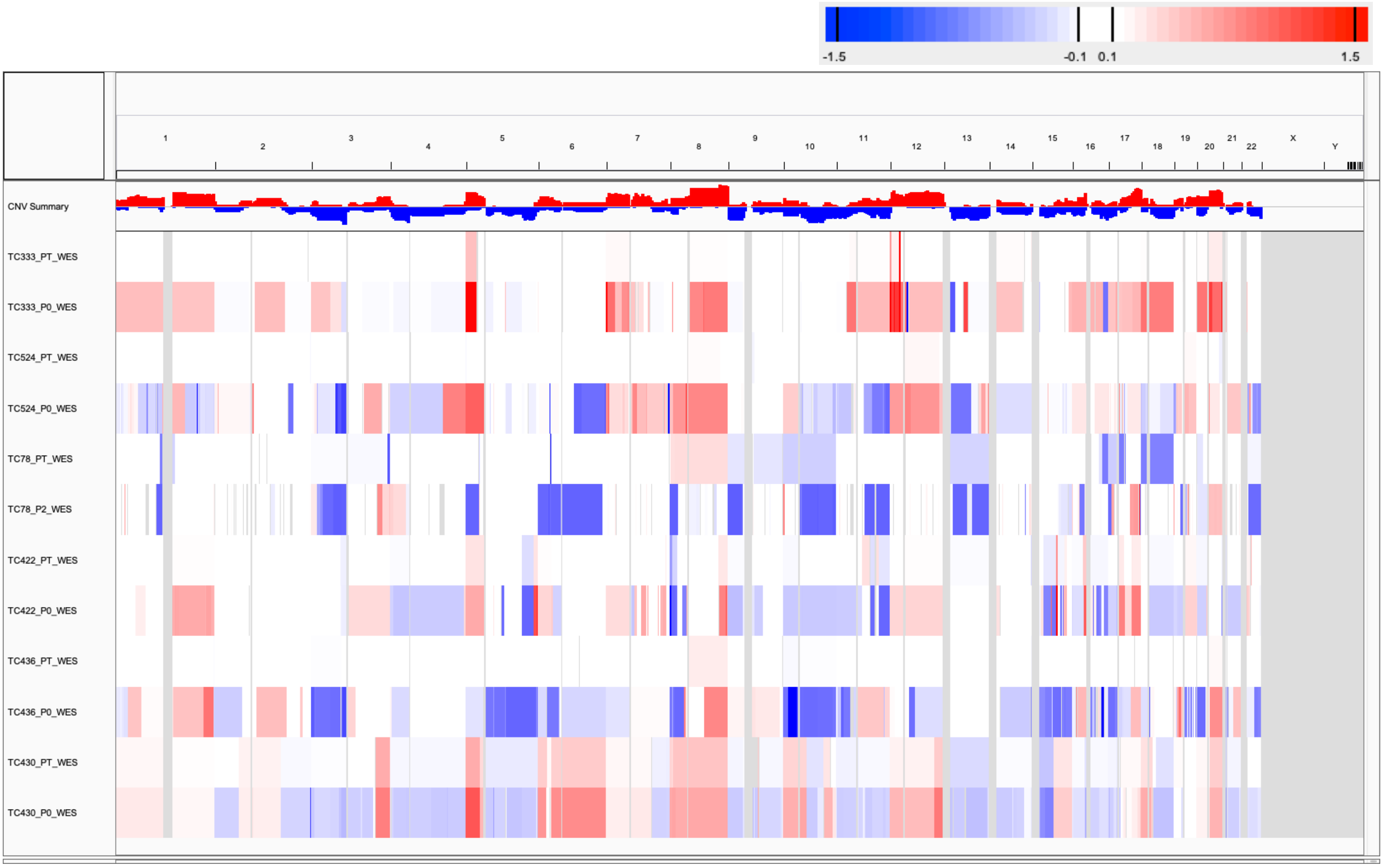

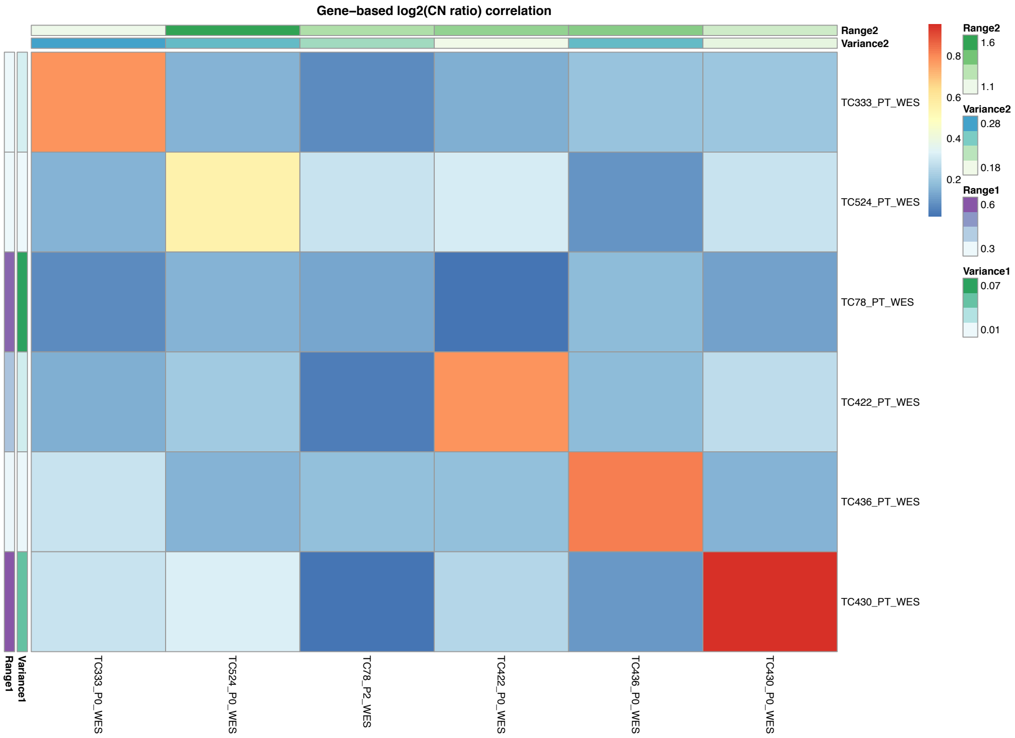

Supplementary Fig. 46: CNA profiles (IGV heatmap) and correlation heatmap of gene-based copy number (log2(CN ratio), median centered) of samples from MDACC WES other lung cancer subtypes dataset.

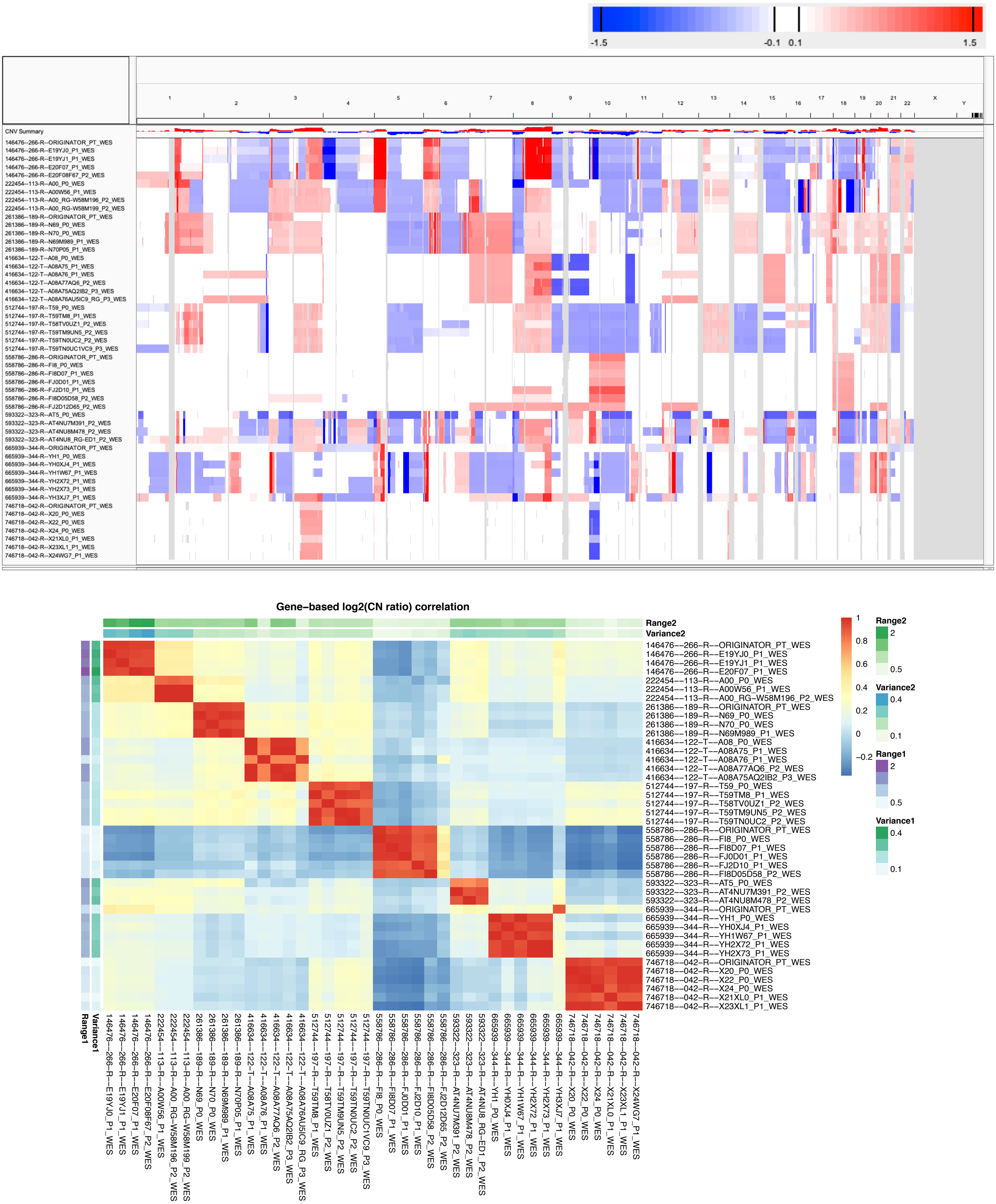

Supplementary Fig. 47: CNA profiles (IGV heatmap) and correlation heatmap of gene-based copy number (log2(CN ratio), median centered) of samples from PDMR WES bladder cancer dataset.

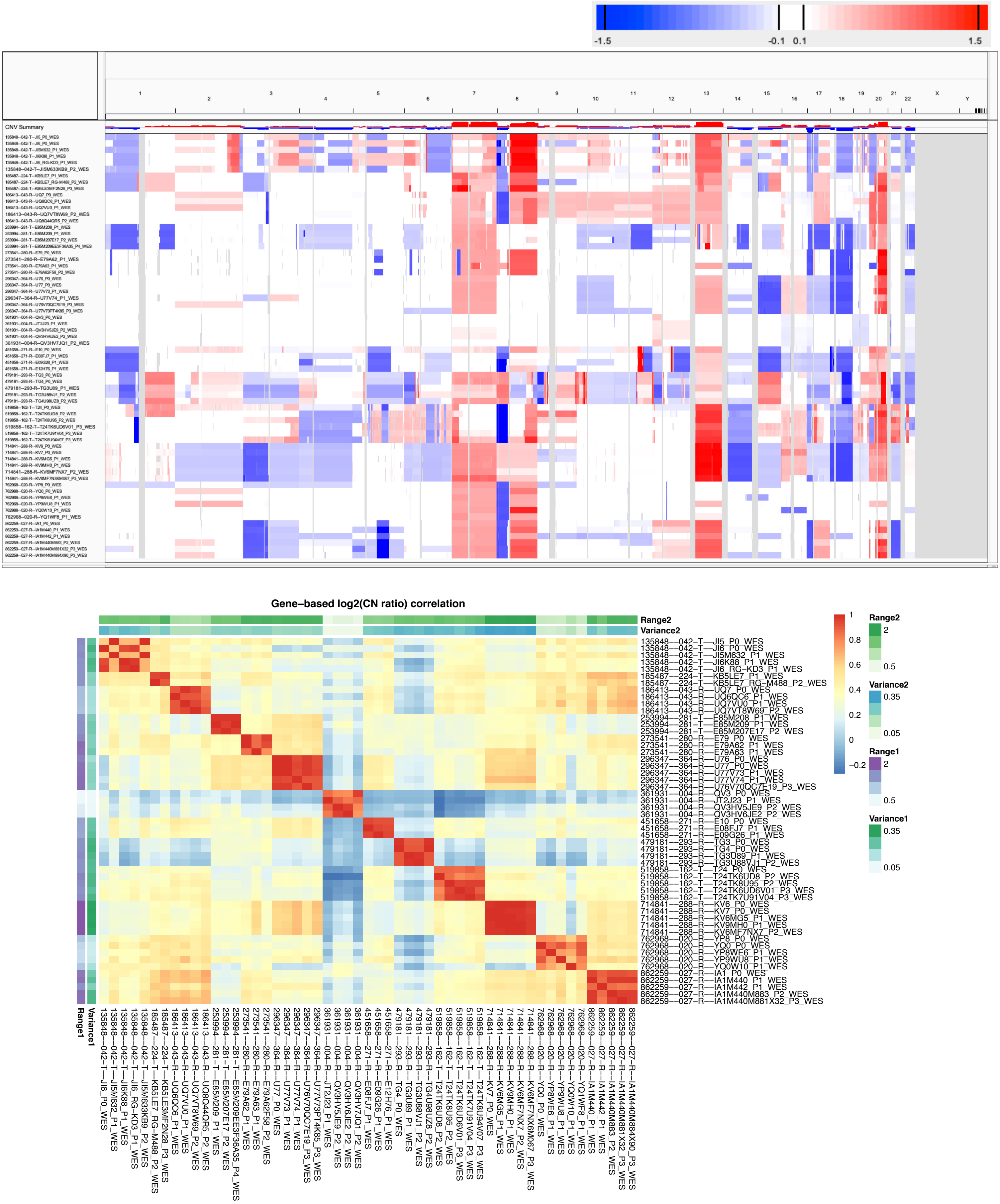

Supplementary Fig. 48: CNA profiles (IGV heatmap) and correlation heatmap of gene-based copy number (log2(CN ratio), median centered) of samples from PDMR WES colorectal cancer dataset.

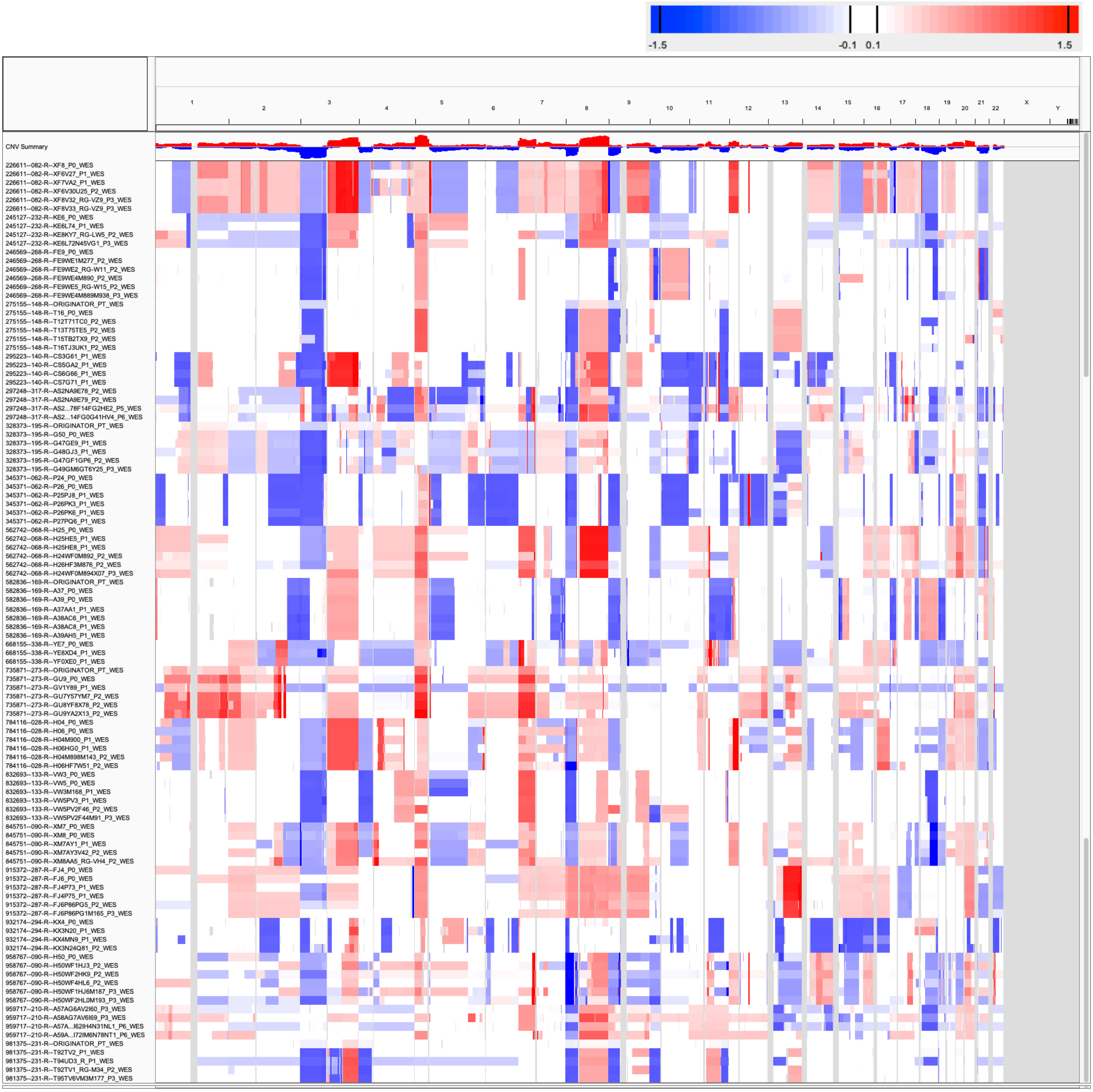

Supplementary Fig. 49: (Continue next page)

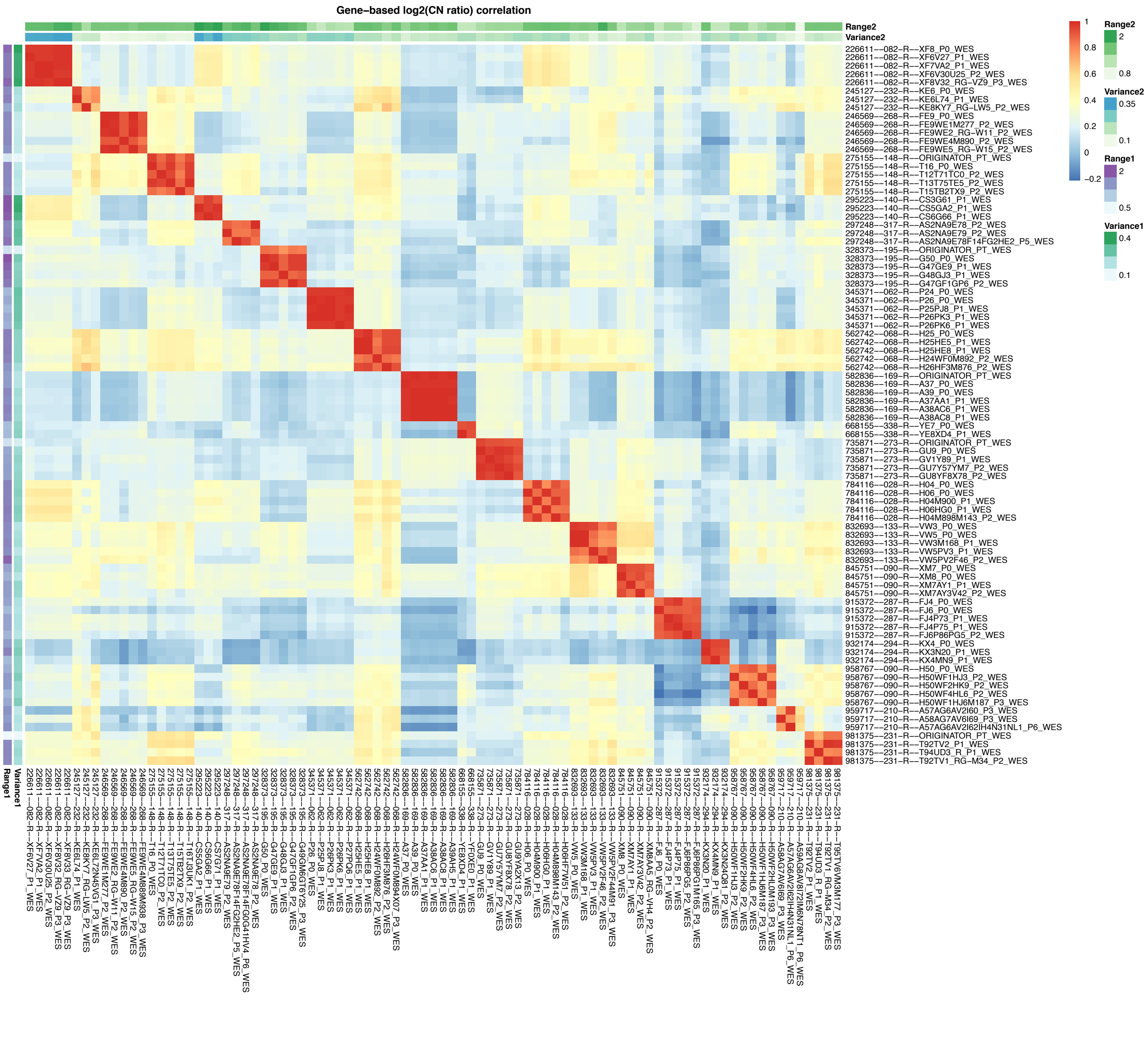

Supplementary Fig. 49: CNA profiles (IGV heatmap) and correlation heatmap of gene-based copy number (log2(CN ratio), median centered) of samples from PDMR WES head and neck cancer dataset.

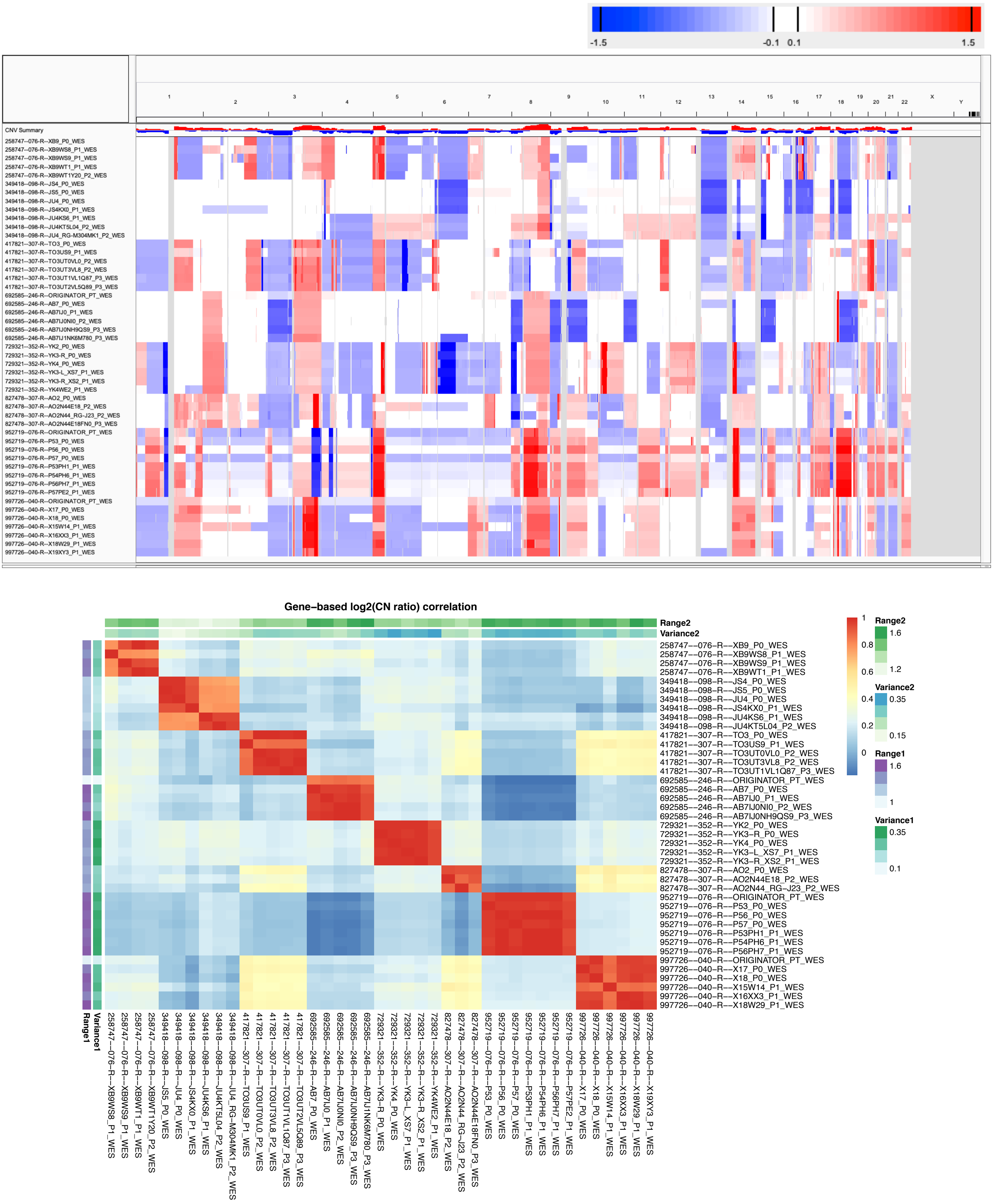

Supplementary Fig. 50: CNA profiles (IGV heatmap) and correlation heatmap of gene-based copy number (log2(CN ratio), median centered) of samples from PDMR WES lung cancer dataset.

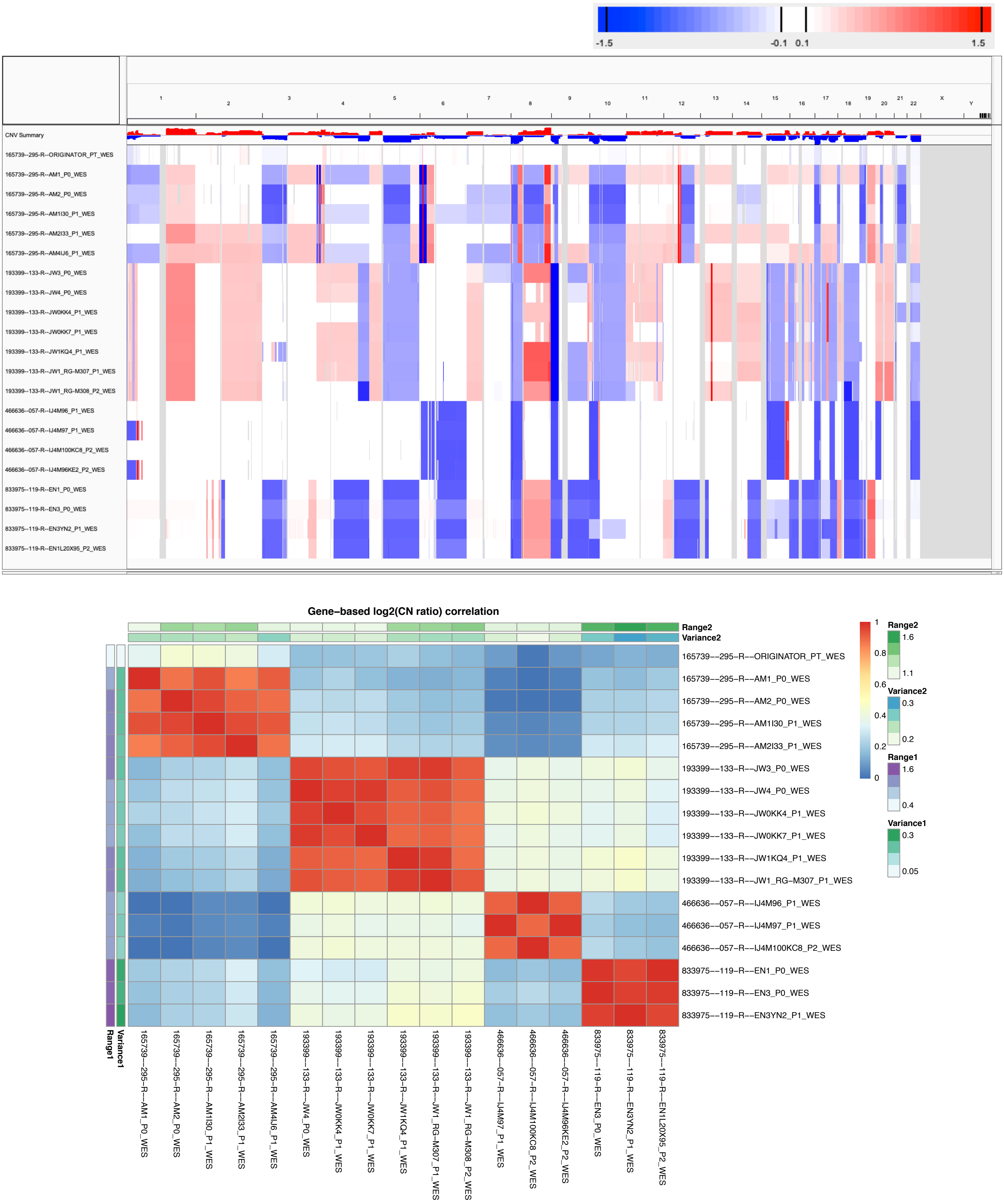

Supplementary Fig. 51: CNA profiles (IGV heatmap) and correlation heatmap of gene-based copy number (log2(CN ratio), median centered) of samples from PDMR WES pancreatic cancer dataset.

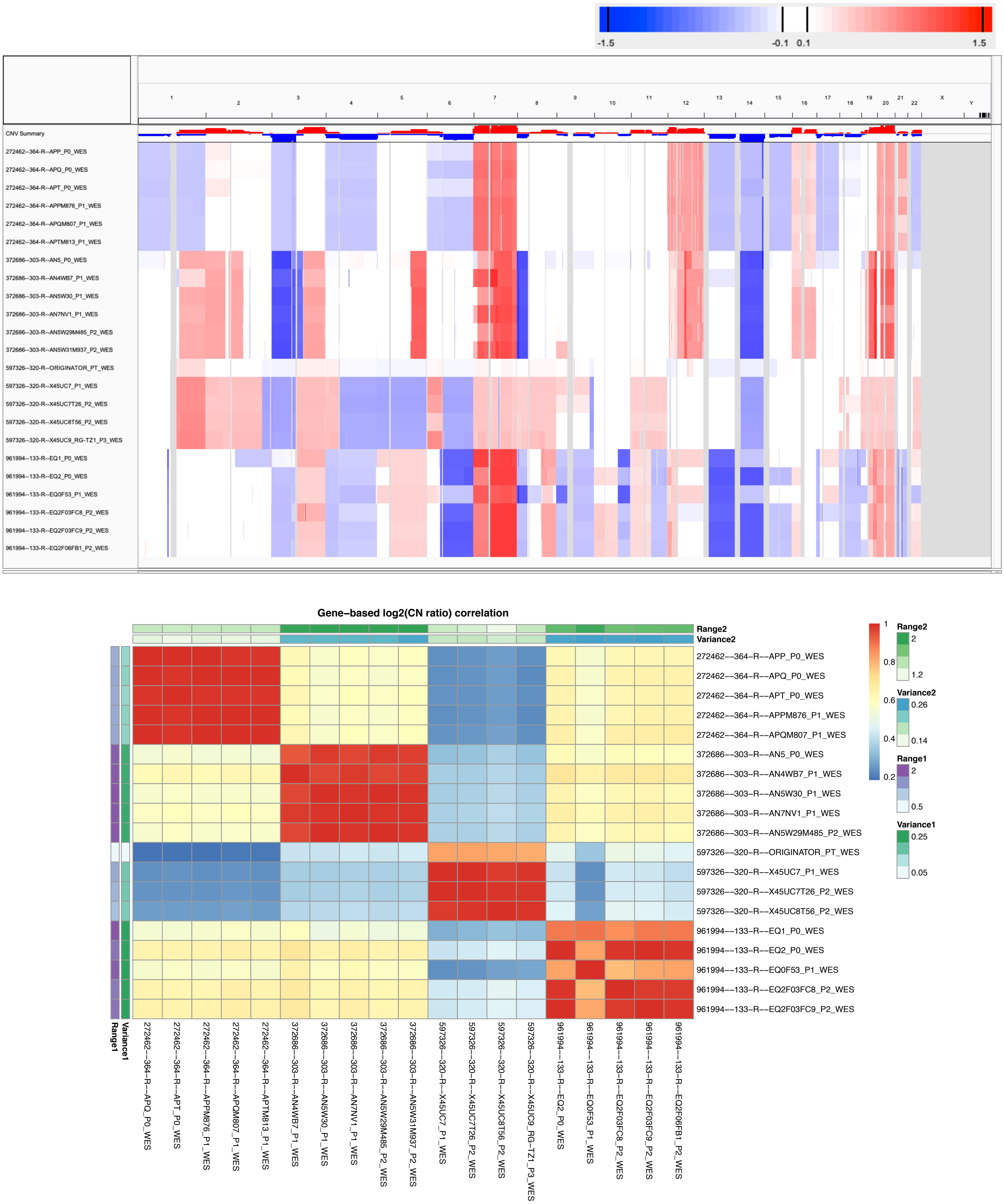

Supplementary Fig. 52: CNA profiles (IGV heatmap) and correlation heatmap of gene-based copy number (log2(CN ratio), median centered) of samples from PDMR WES renal cancer dataset.

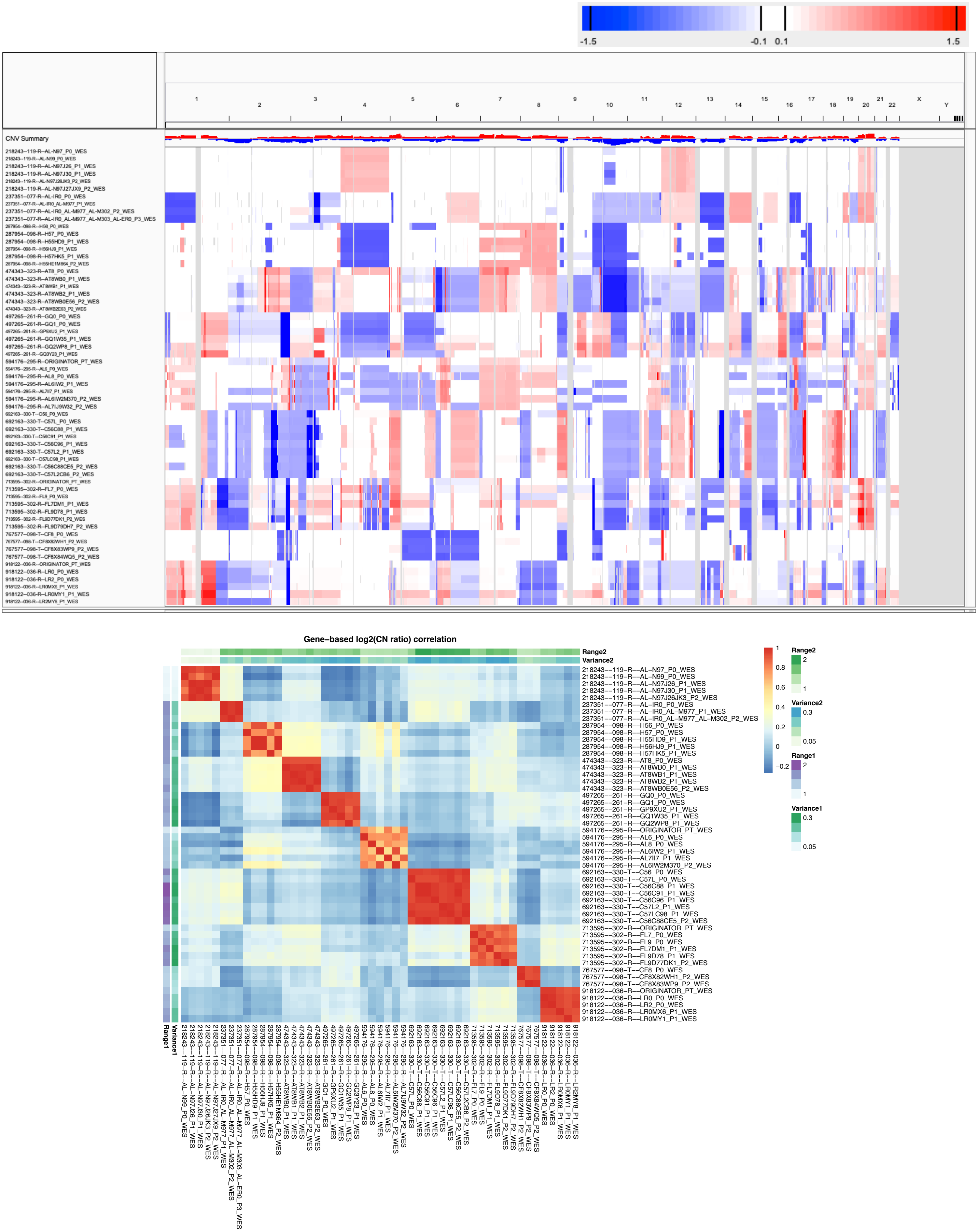

Supplementary Fig. 53: CNA profiles (IGV heatmap) and correlation heatmap of gene-based copy number (log2(CN ratio), median centered) of samples from PDMR WES sarcoma dataset.

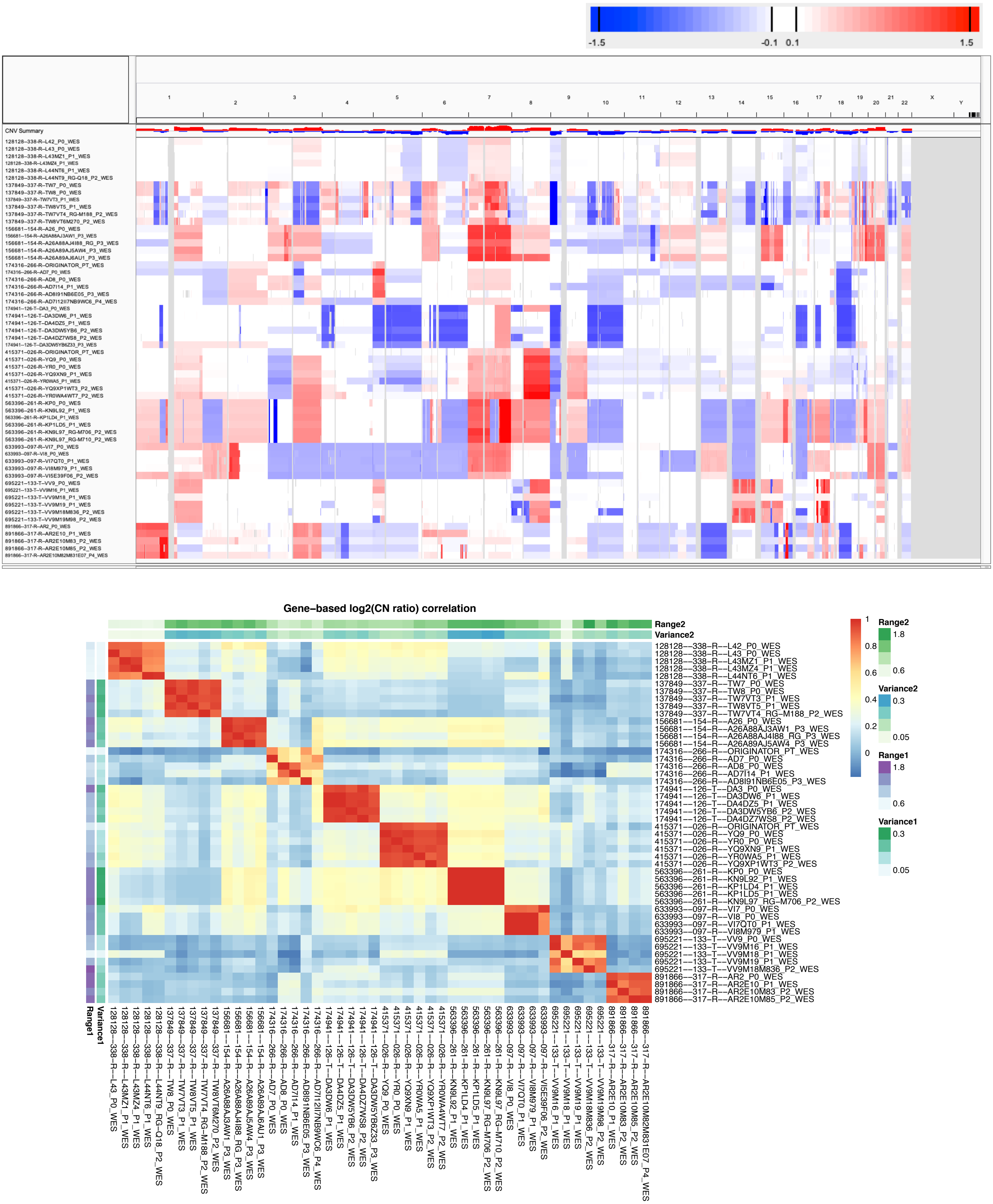

Supplementary Fig. 54: CNA profiles (IGV heatmap) and correlation heatmap of gene-based copy number (log2(CN ratio), median centered) of samples from PDMR WES skin cancer dataset.

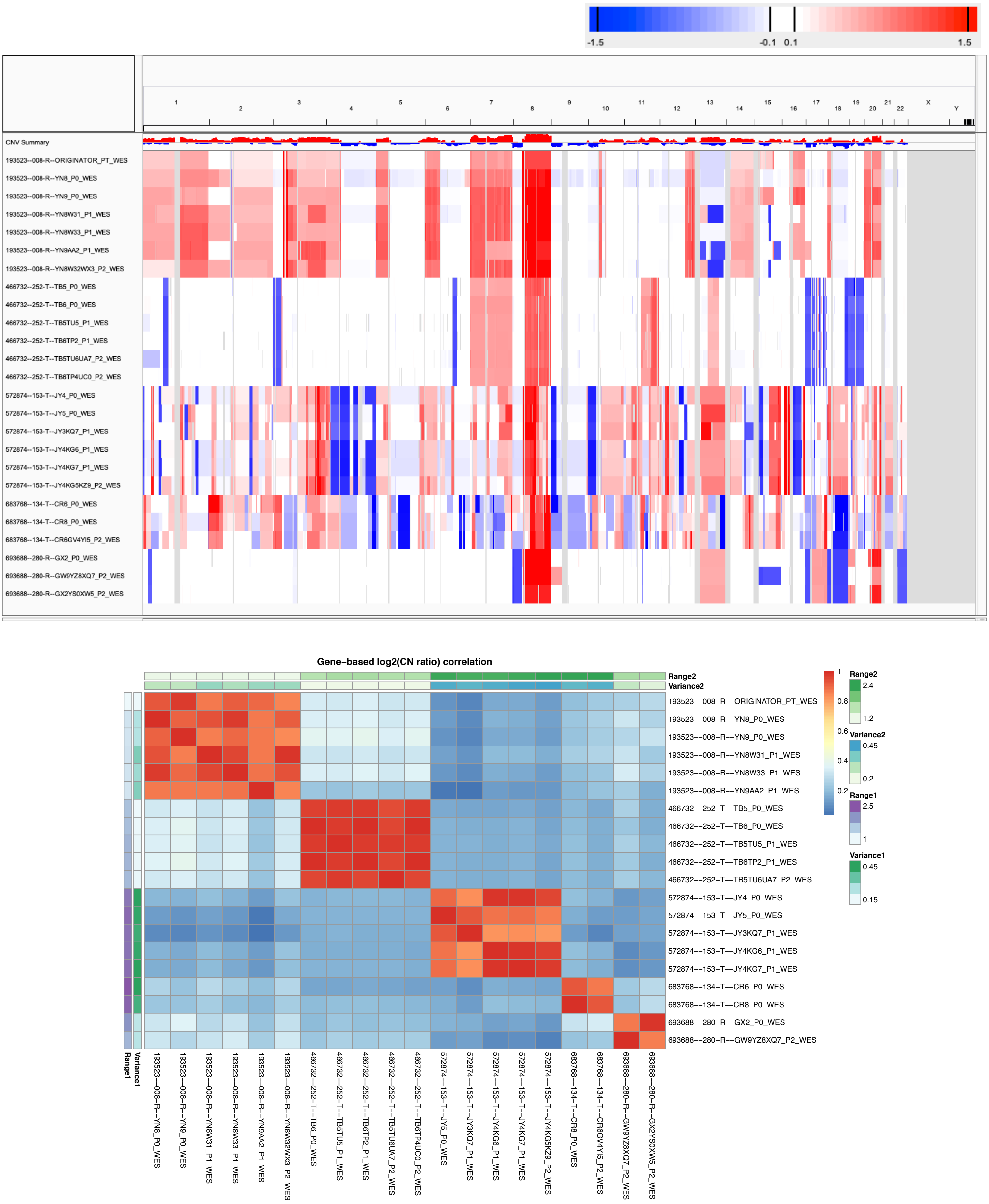

Supplementary Fig. 55: CNA profiles (IGV heatmap) and correlation heatmap of gene-based copy number (log2(CN ratio), median centered) of samples from PDMR WES other cancers dataset.

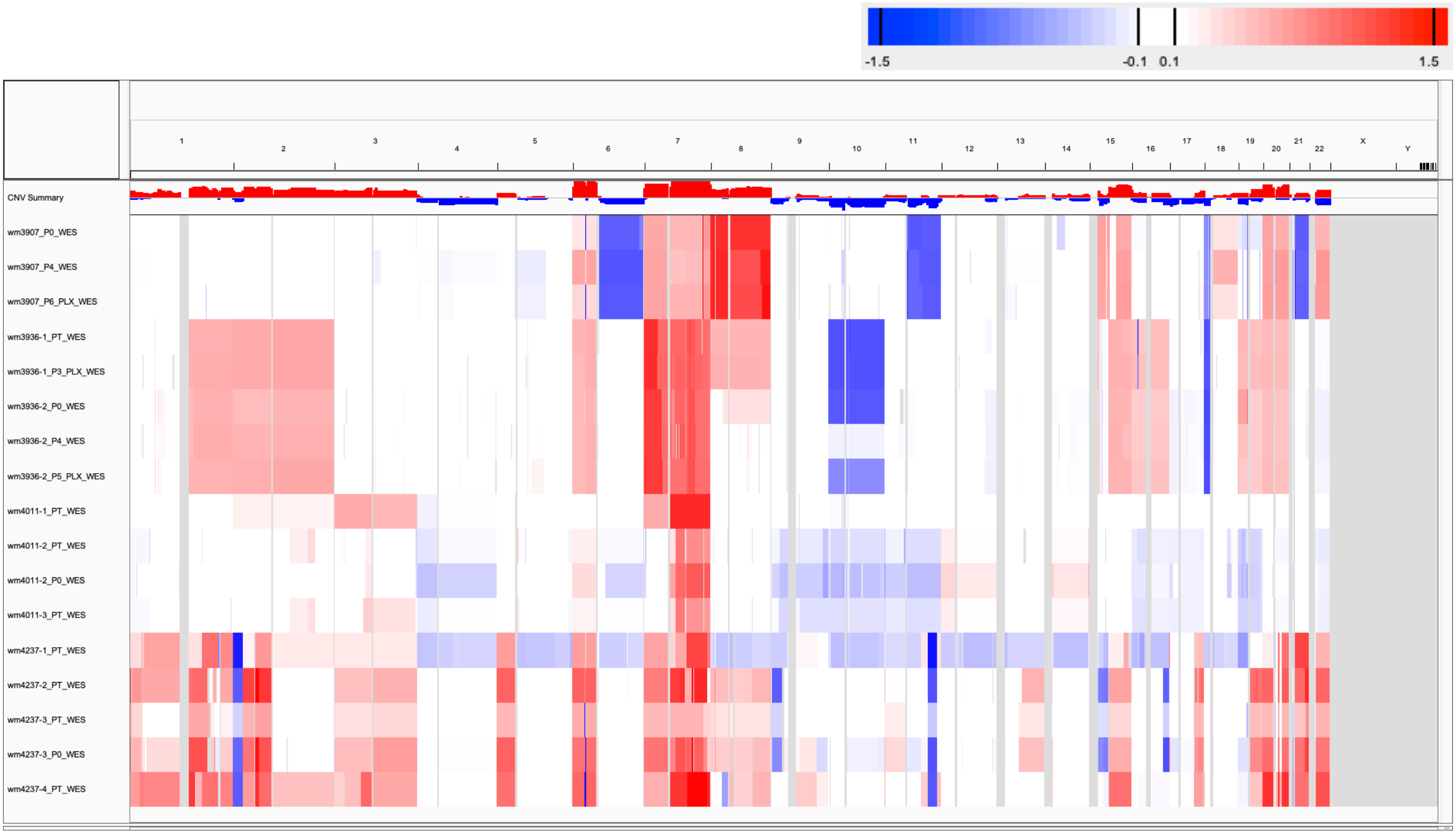

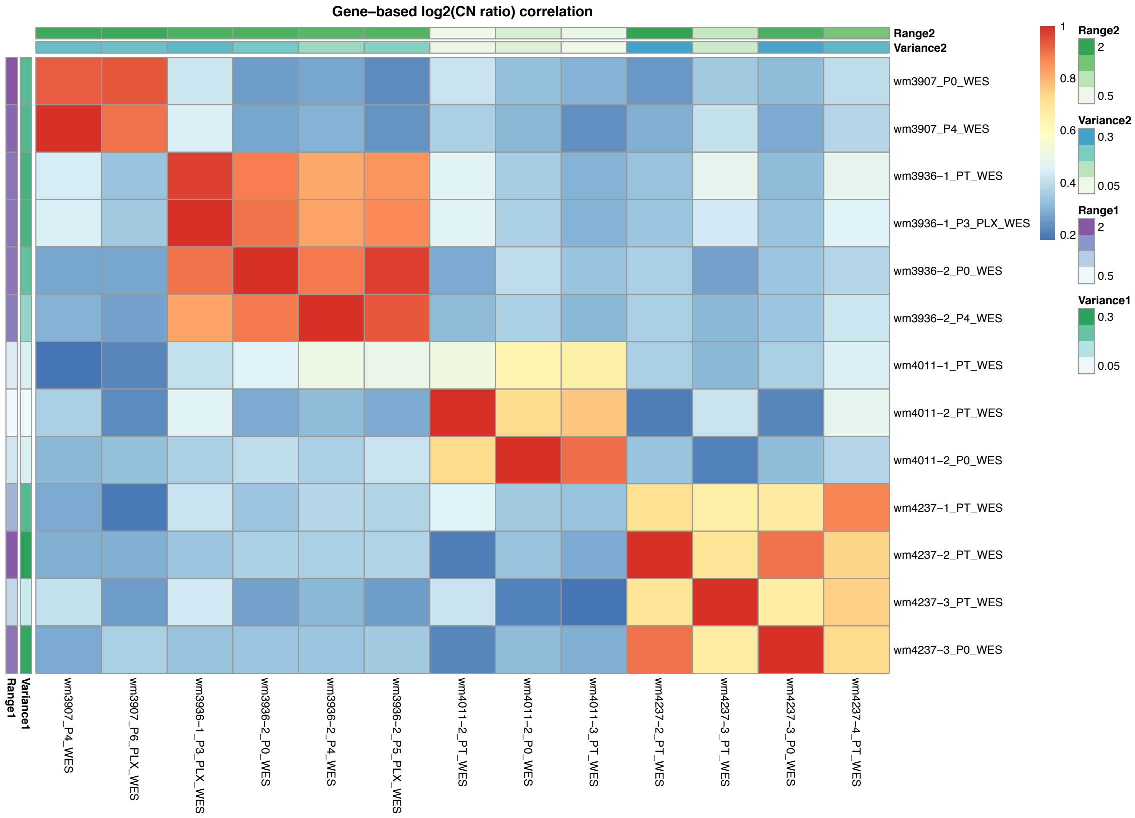

Supplementary Fig. 56: CNA profiles (IGV heatmap) and correlation heatmap of gene-based copy number (log2(CN ratio), median centered) of samples from WISTAR WES skin melanoma dataset.

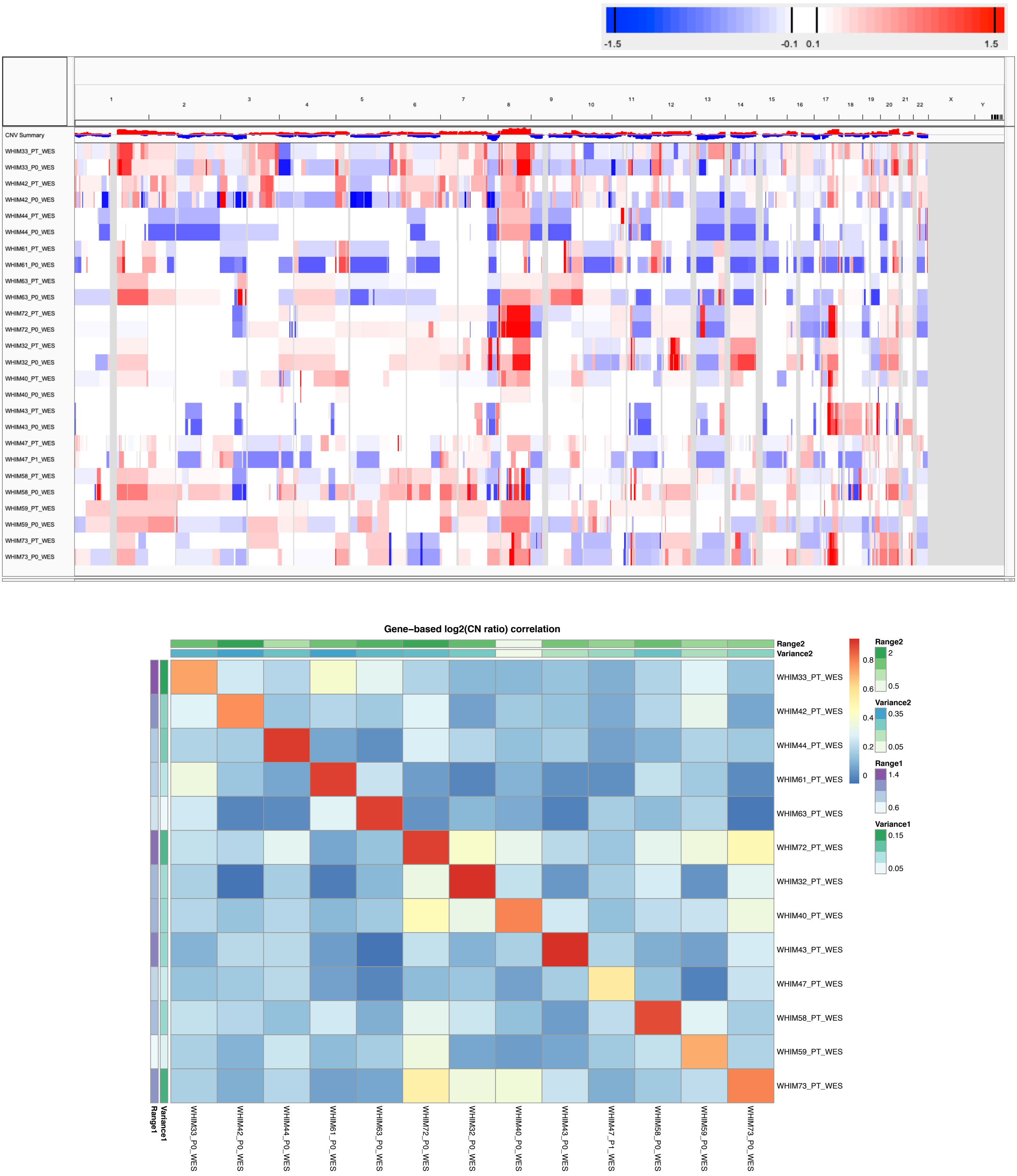

Supplementary Fig. 57: CNA profiles (IGV heatmap) and correlation heatmap of gene-based copy number (log2(CN ratio), median centered) of samples from WUSTL WES breast cancer dataset.

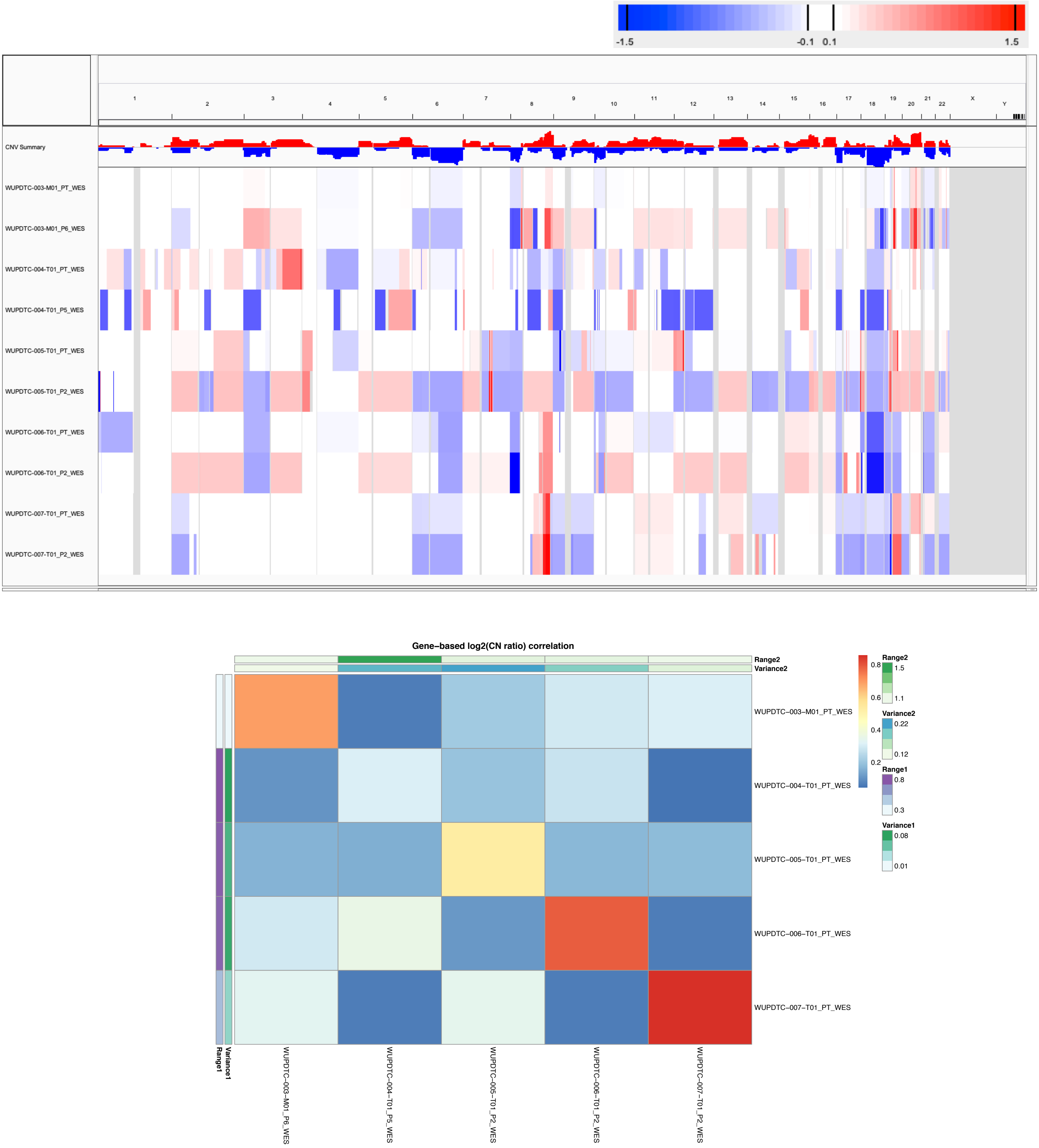

Supplementary Fig. 58: CNA profiles (IGV heatmap) and correlation heatmap of gene-based copy number (log2(CN ratio), median centered) of samples from WUSTL WES pancreatic cancer dataset.

Supplementary Fig. 59: (Continue next page)

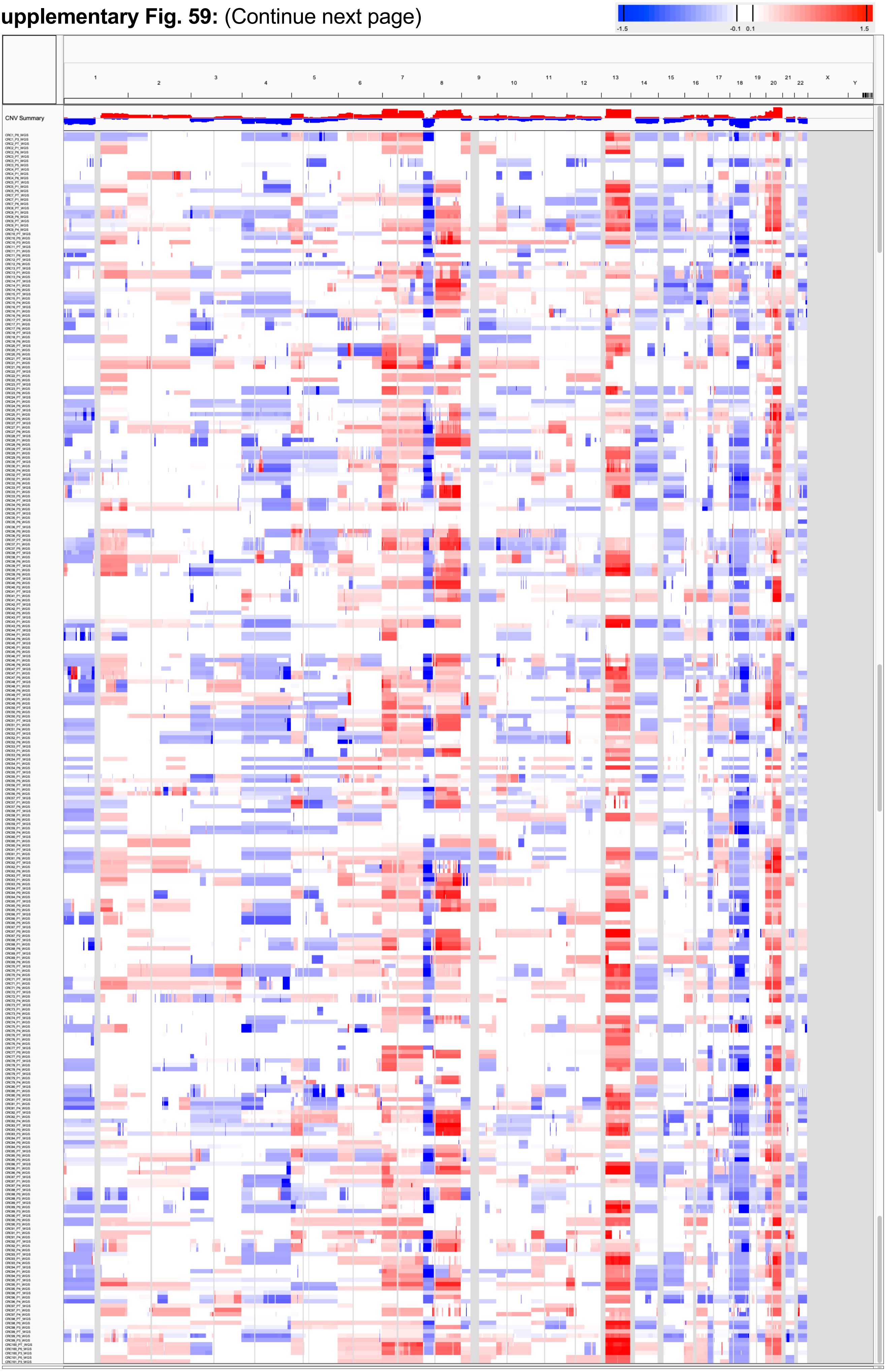

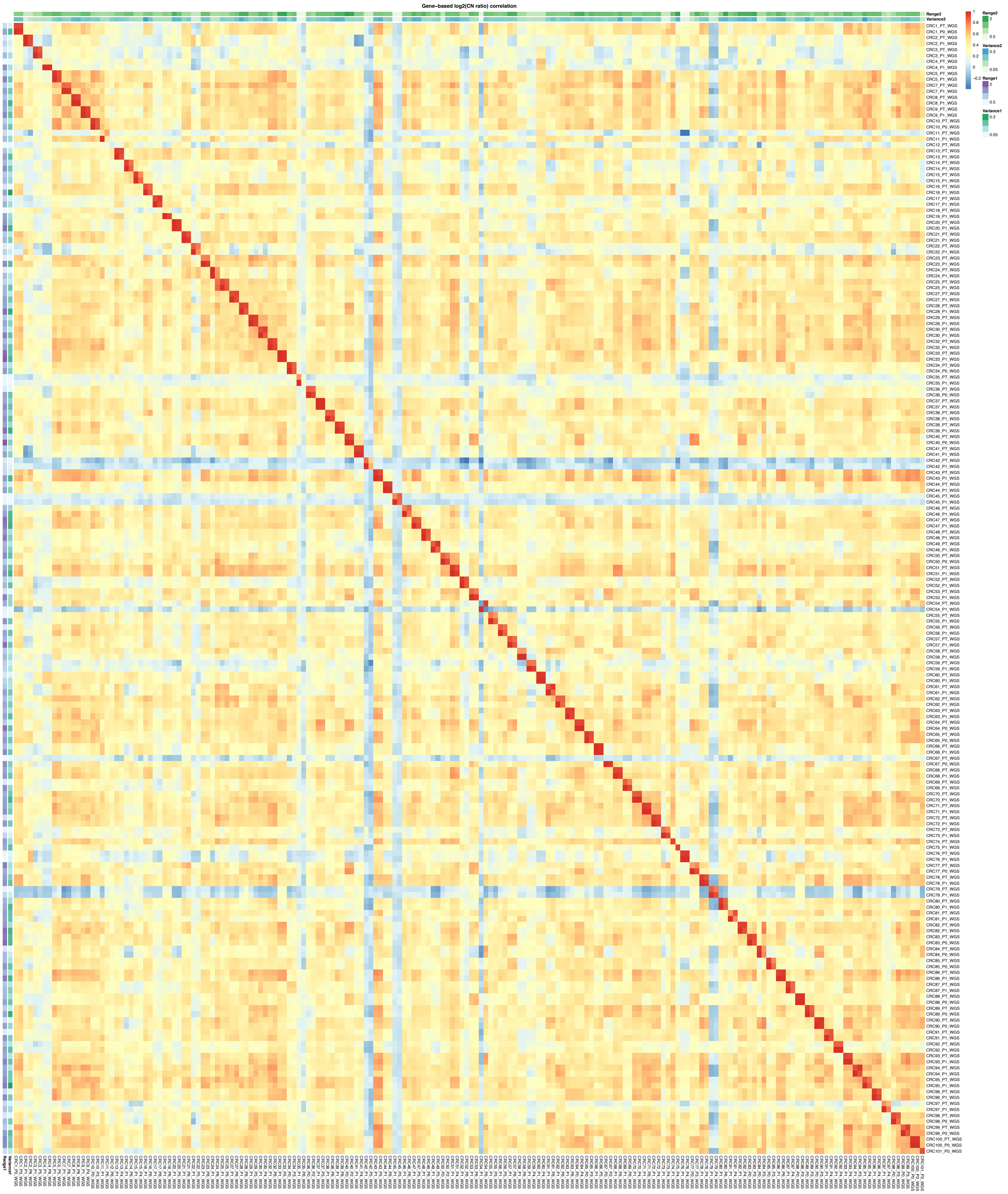

Supplementary Fig. 59: CNA profiles (IGV heatmap) and correlation heatmap of gene-based copy number (log2(CN ratio), median centered) of samples from EuroPDX WGS colorectal cancer (liver metastases) dataset.

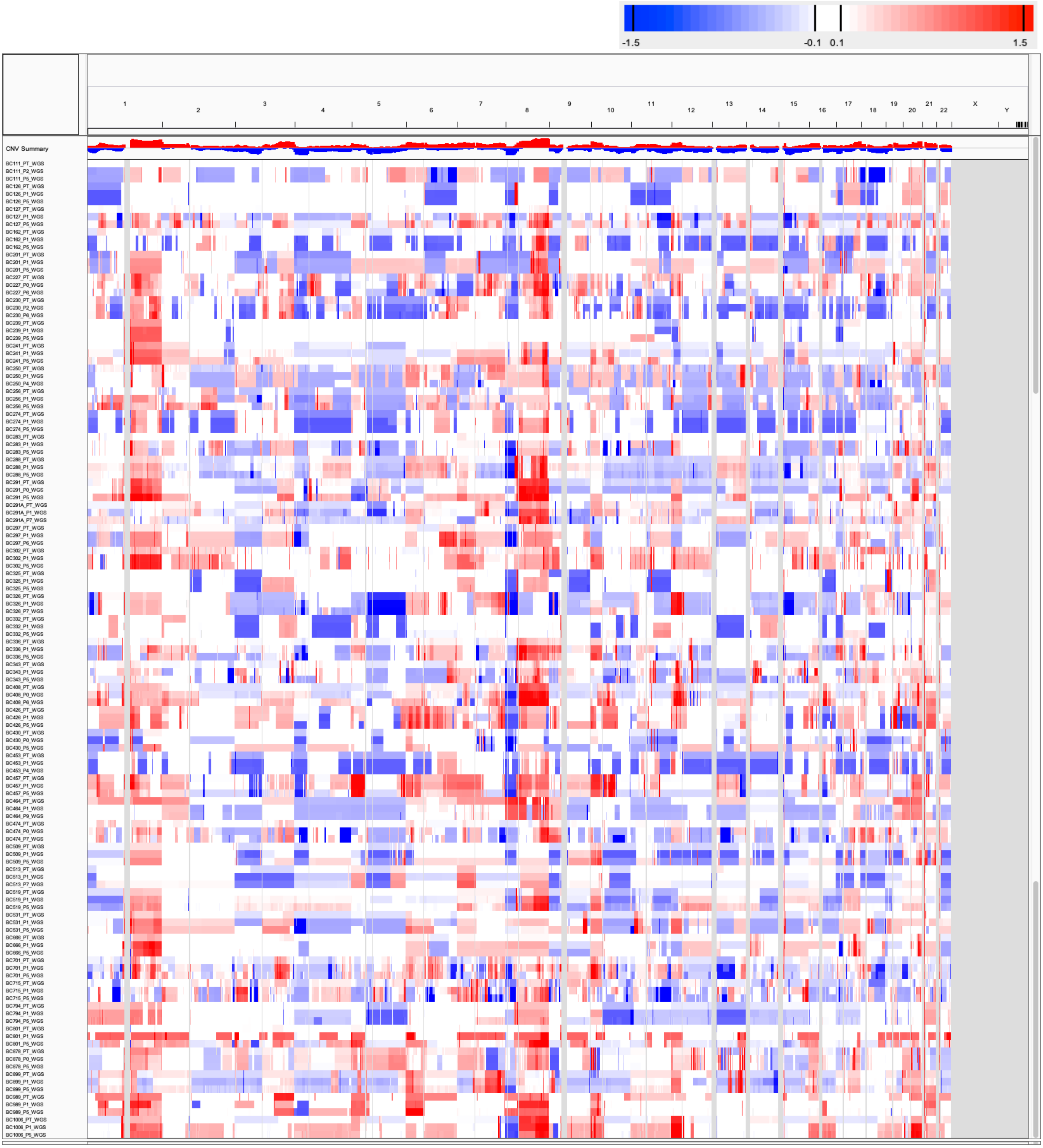

Supplementary Fig. 60: (Continue next page)

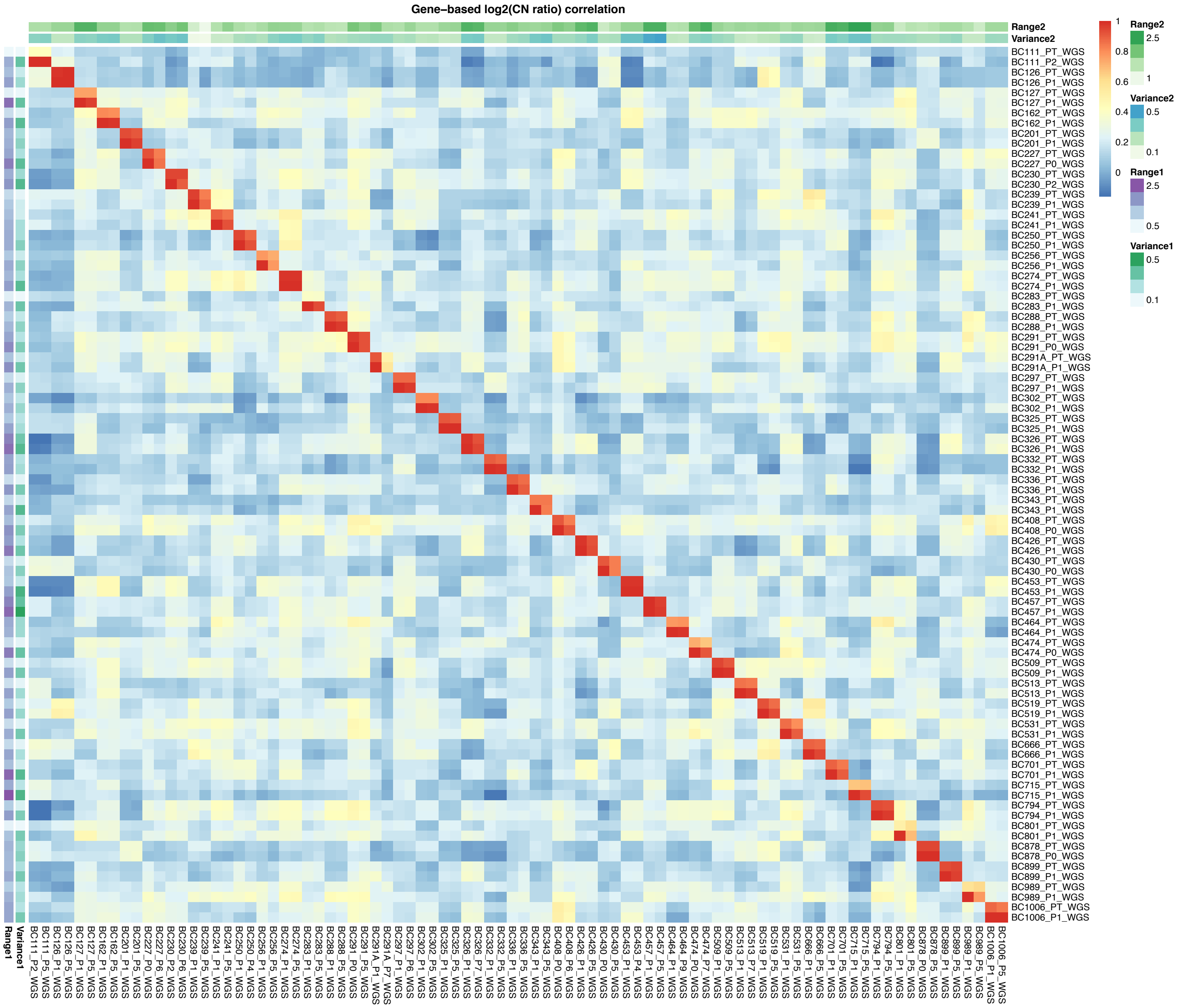

Supplementary Fig. 60: CNA profiles (IGV heatmap) and correlation heatmap of gene-based copy number (log2(CN ratio), median centered) of samples from EuroPDX WGS breast cancer dataset.

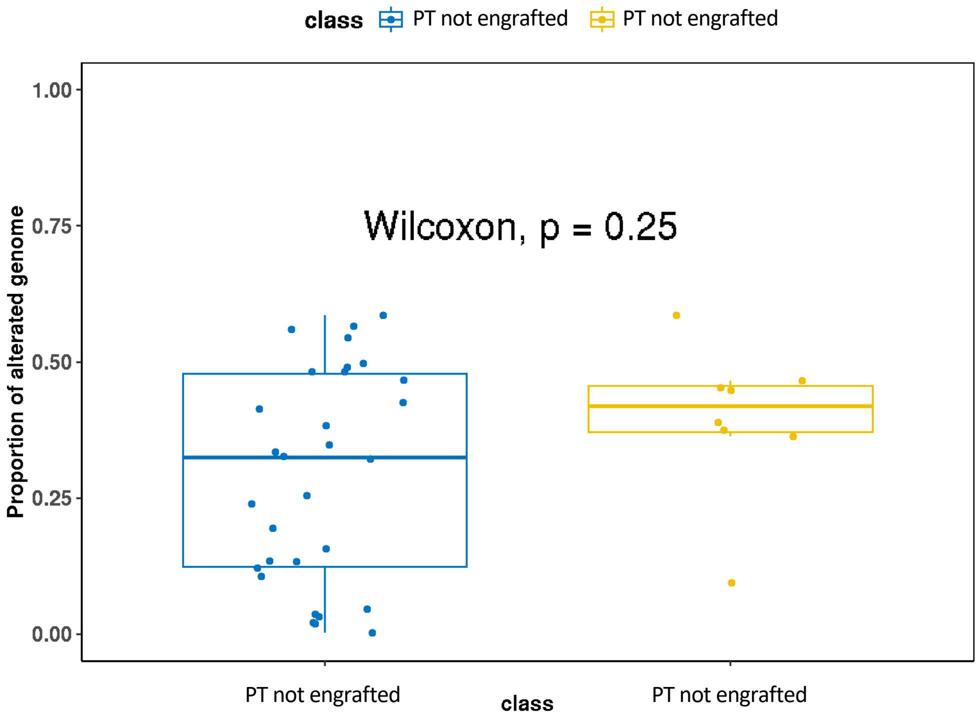

Supplementary Fig. 61: Boxplots comparing the fraction of genome altered by CNA events between patient tumors (n=38) that were (n=8) and were not engrafted successfully. The tumors were a subset of the the EuroPDX BRCA dataset and the copy number profiles were obtained using array CGH data. P-values were computed by two-sided Wilcoxon rank sum test. In the boxplot, the center line is the median, box limits are the upper and lower quantiles, whiskers extend 1.5 × the interquartile range, dots represent the all data points.

a

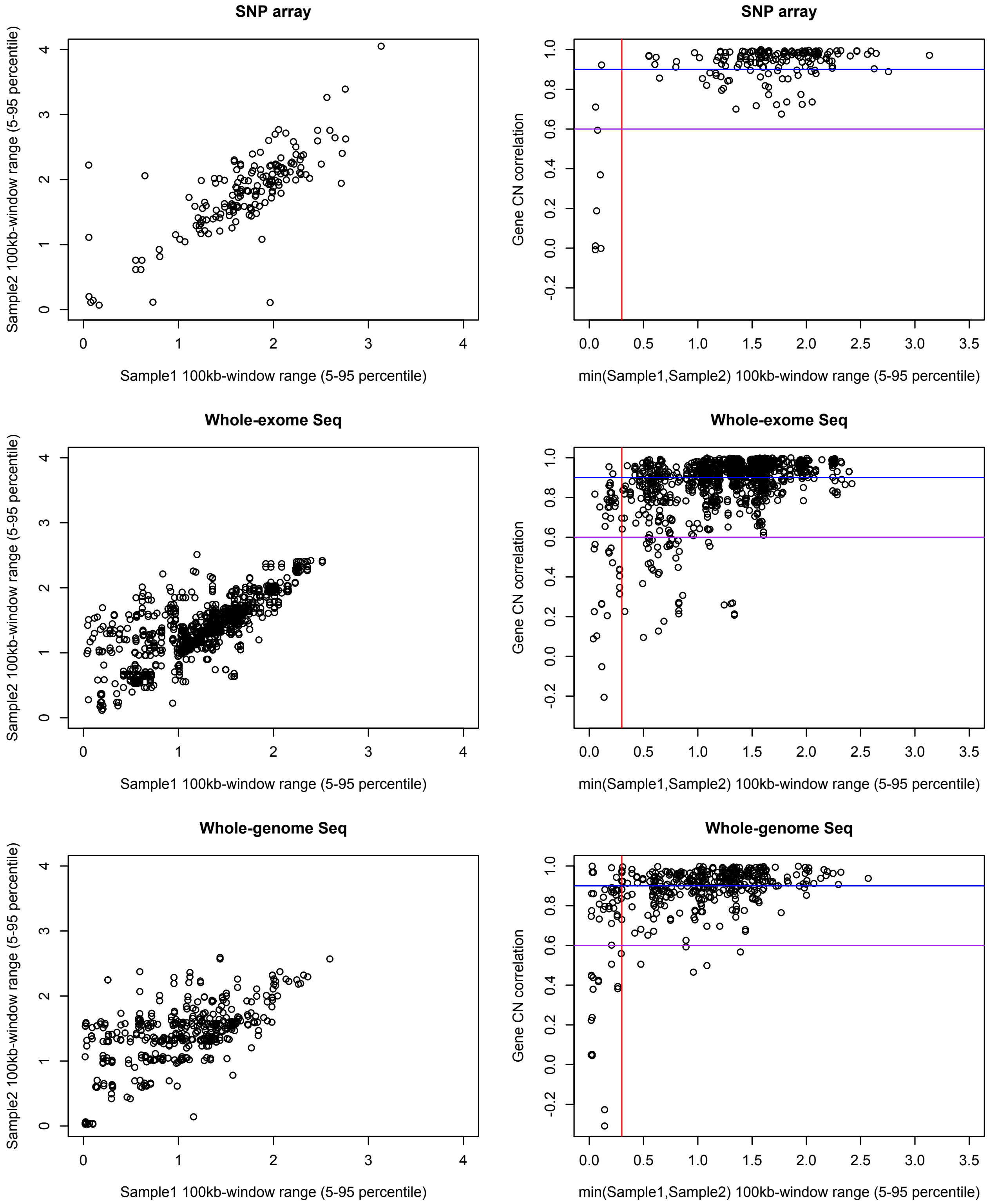

Supplementary Fig. 62: (Continue next page)

b

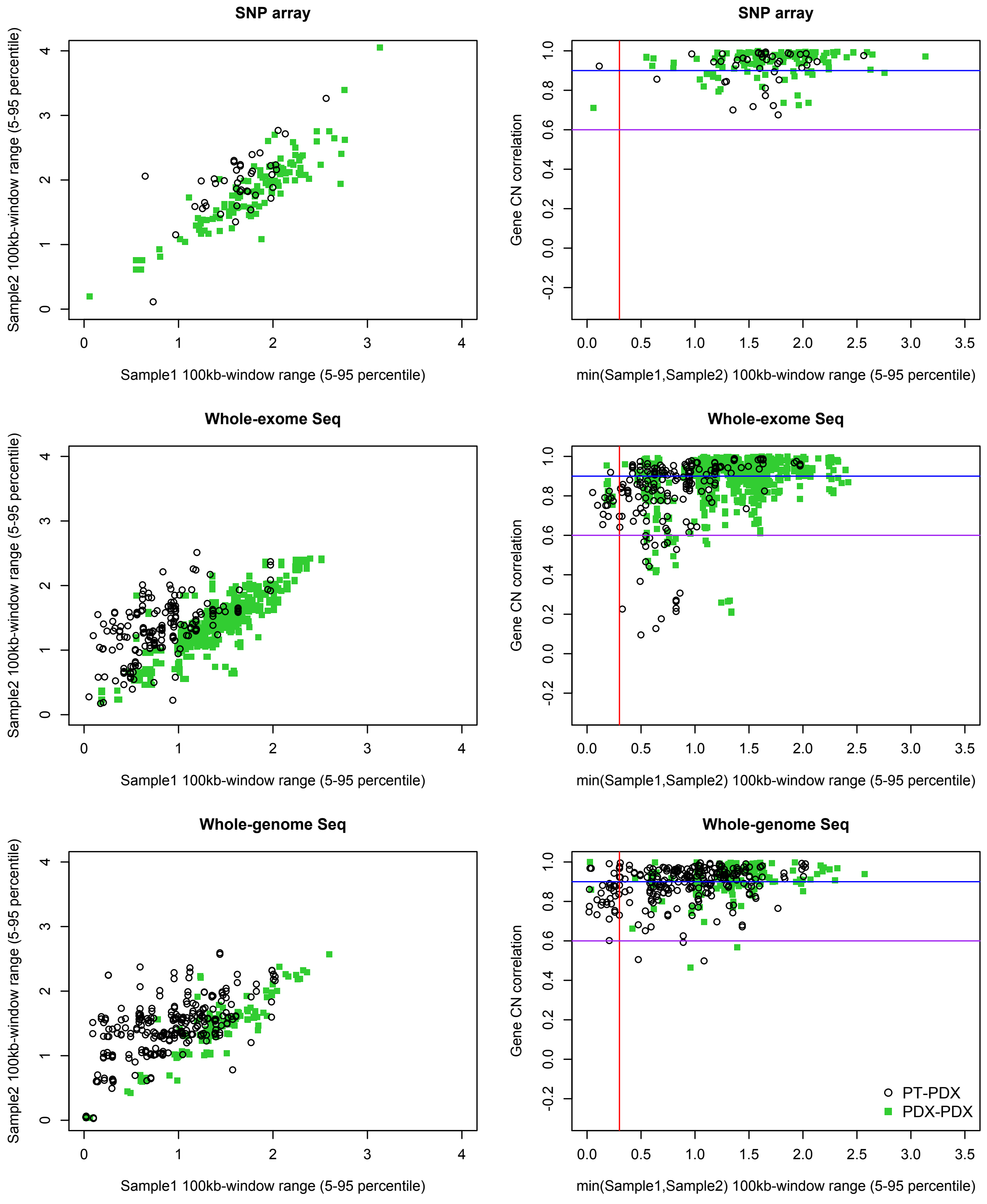

Supplementary Fig. 62: PT samples have a lower range of CNA values than PDX samples. Comparison of 5-95% inter-percentile range of log2(CN ratio) values of CNA in pairs of samples (PT or PDX) from the same model (left panel). Pearson correlation of the samples versus the minimum range of the two samples (right panel). Samples with lower range tend to have lower correlations with other samples. For a given sample, the 5-95% inter-percentile range of log2(CN ratio) values were calculated across all 100kb-windows binned from copy number segments of each sample. (**a**) All data; (**b**) After removing comparisons of low correlation (< 0.6) due to non-aberrant samples (range < 0.3). Sample 1: PT or lower passage PDX, Sample 2: later passage PDX or same passage PDX of different lineage.

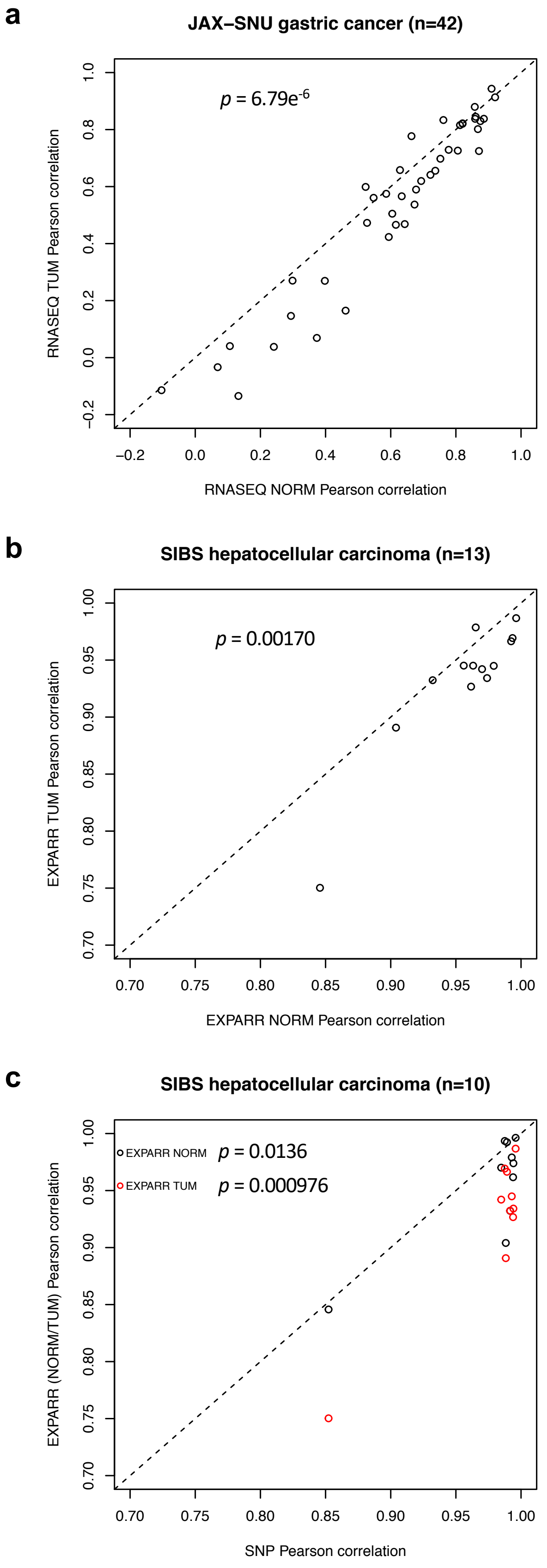

Supplementary Fig. 63: Scatter plots to compare Pearson correlation coefficients of gene-based copy number using SNP array, gene expression array normalized using median expression of normal (RNASEQ/EXPARR NORM) and tumor (RNASEQ/EXPARR TUM) samples. (**a**) RNASEQ NORM versus TUM, (**b**) EXPARR NORM versus TUM, and (**c**) SNP array versus EXPARR NORM/TUM. P-values were computed by one-sided Wilcoxon signed-rank test. In all plots, number of pairwise correlations are indicated in each plot title.

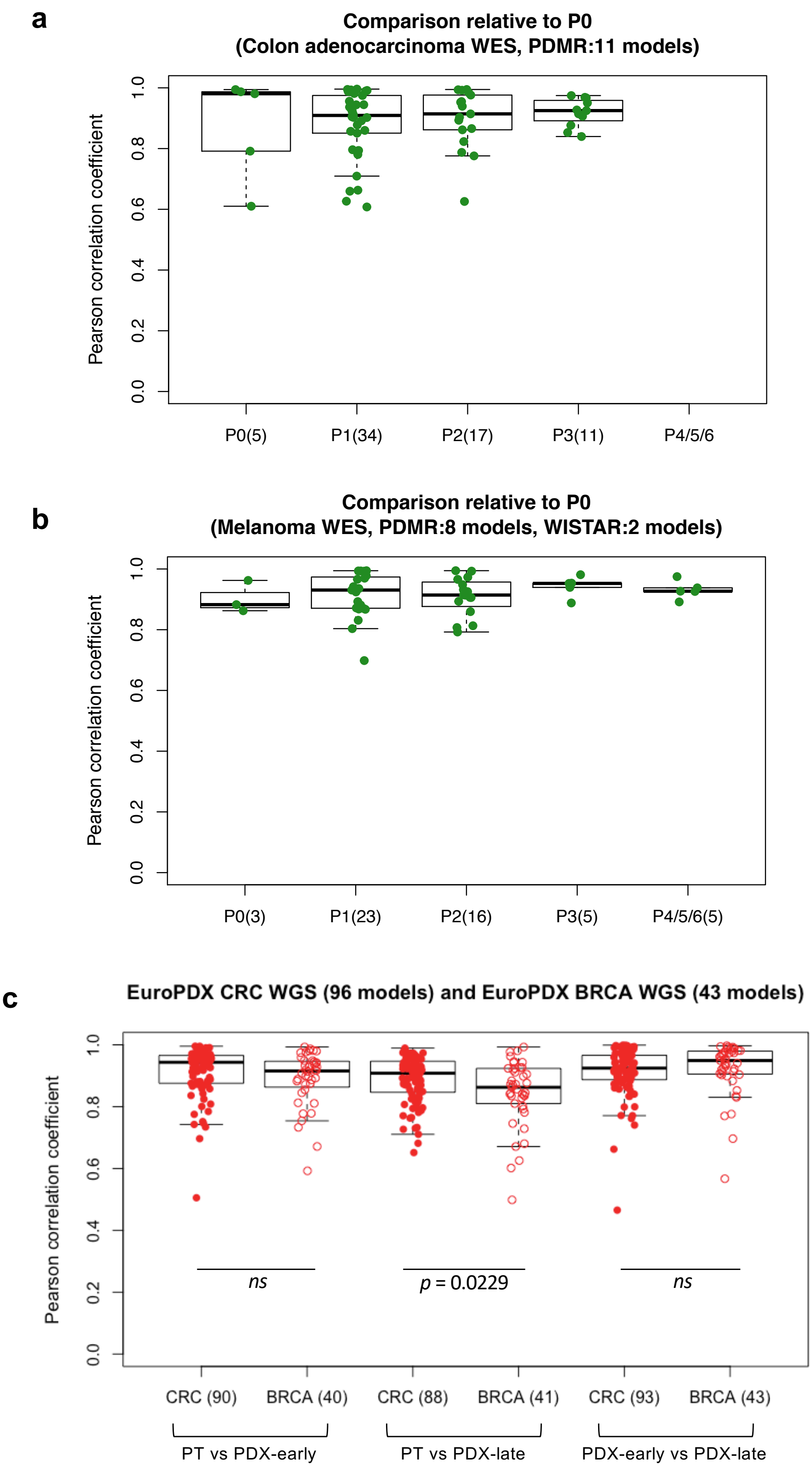

Supplementary Fig. 64: Distribution of Pearson correlation coefficients of gene-based copy number between different combinations of PT and PDX passages of the same model for specific tumor types, (**a**) PDMR colon carcinoma (WES), (**b**) PDMR and WISTAR melanoma (WES), (**c**) EuroPDX CRC and BRCA (WGS). PDX-early comprises of P0 to P1 for CRC and P0 to P2 for BRCA, and PDX-late comprises of P2 to P6 for CRC and P4 to P9 for BRCA. P-values were computed by two-sided Wilcoxon rank sum test (ns: non-significant, $p > 0.05$). In all boxplots, the center line is the median, box limits are the upper and lower quantiles, whiskers extend 1.5 × the interquartile range, dots represent the all data points. In all plots, number of models are indicated in each plot title, number of pairwise correlations per boxplot are indicated in the horizontal axis labels.

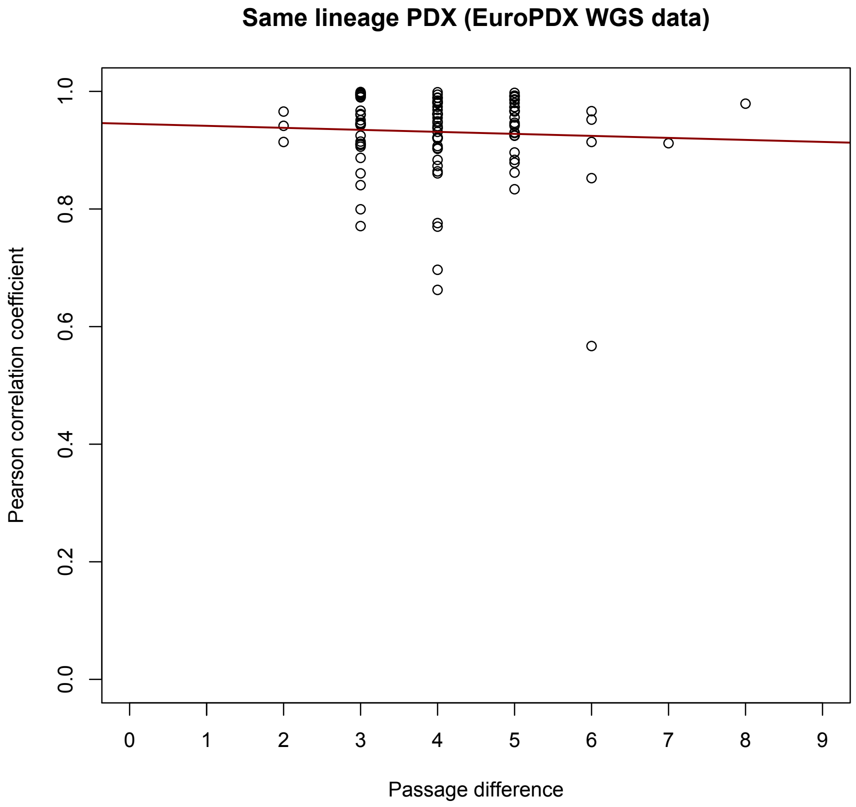

Supplementary Fig. 65: Scatter plot of Pearson correlation between samples of PDX-early and PDX-late versus the corresponding passage difference for same lineage samples.

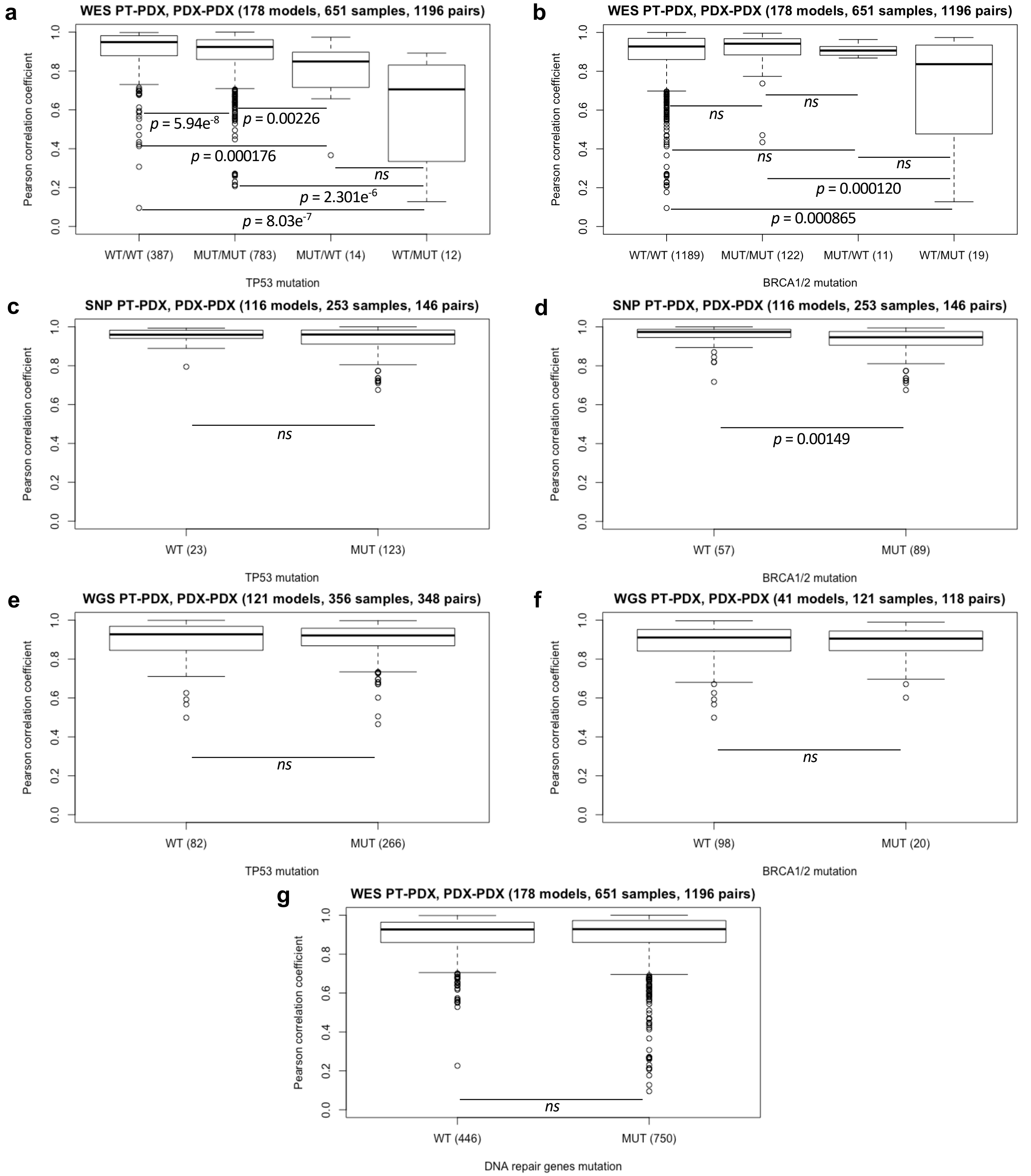

Supplementary Fig. 66: Distribution of Pearson correlation coefficients of gene-based copy number for mutational statuses (WT: wildtype, MUT: mutant) of (**a**) – (**f**) TP53 or BRCA1/BRCA2 of the samples or models for each correlation pair for, (**a**) and (**b**) WES, (**c**) and (**d**) SNP array, (**e**) and (**f**) WGS, and (**g**) DNA repair genes for WES. For SNP array and WGS data, only models with available mutational statuses are included. P-values were computed by one-sided Wilcoxon rank sum test (ns: non-significant, $p > 0.05$). In all boxplots, the center line is the median, box limits are the upper and lower quantiles, whiskers extend 1.5 × the interquartile range, dots represent outliers. In all plots, number of models, samples and pairwise correlations are indicated in each plot title, number of pairwise correlations per boxplot are indicated in the horizontal axis labels.

a

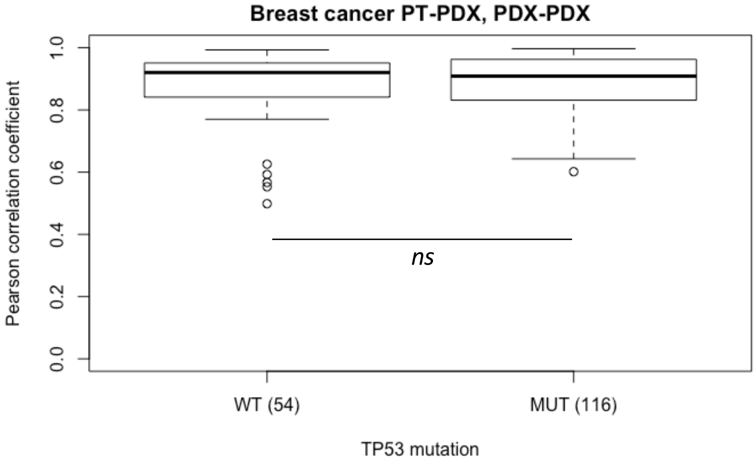

b

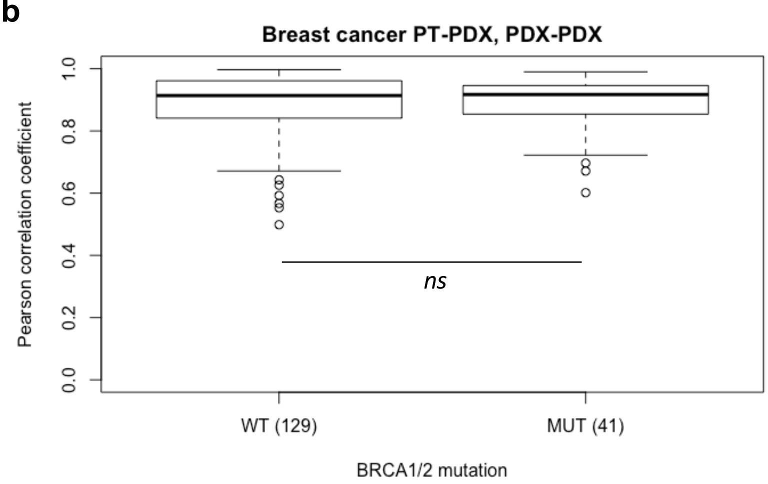

Supplementary Fig. 67: Distribution of Pearson correlation coefficients of gene-based copy number for mutational statuses (WT: wildtype, MUT: mutant) of (**a**) TP53 or (**b**) BRCA1/BRCA2 of the models for each correlation pair for all breast cancer models across all platforms, where mutational status is available. P-values were computed by one-sided Wilcoxon rank sum test (ns: non-significant, $p > 0.05$). In all boxplots, the center line is the median, box limits are the upper and lower quantiles, whiskers extend 1.5 × the interquartile range, dots represent outliers. In all plots, number of pairwise correlations per boxplot are indicated in the horizontal axis labels.

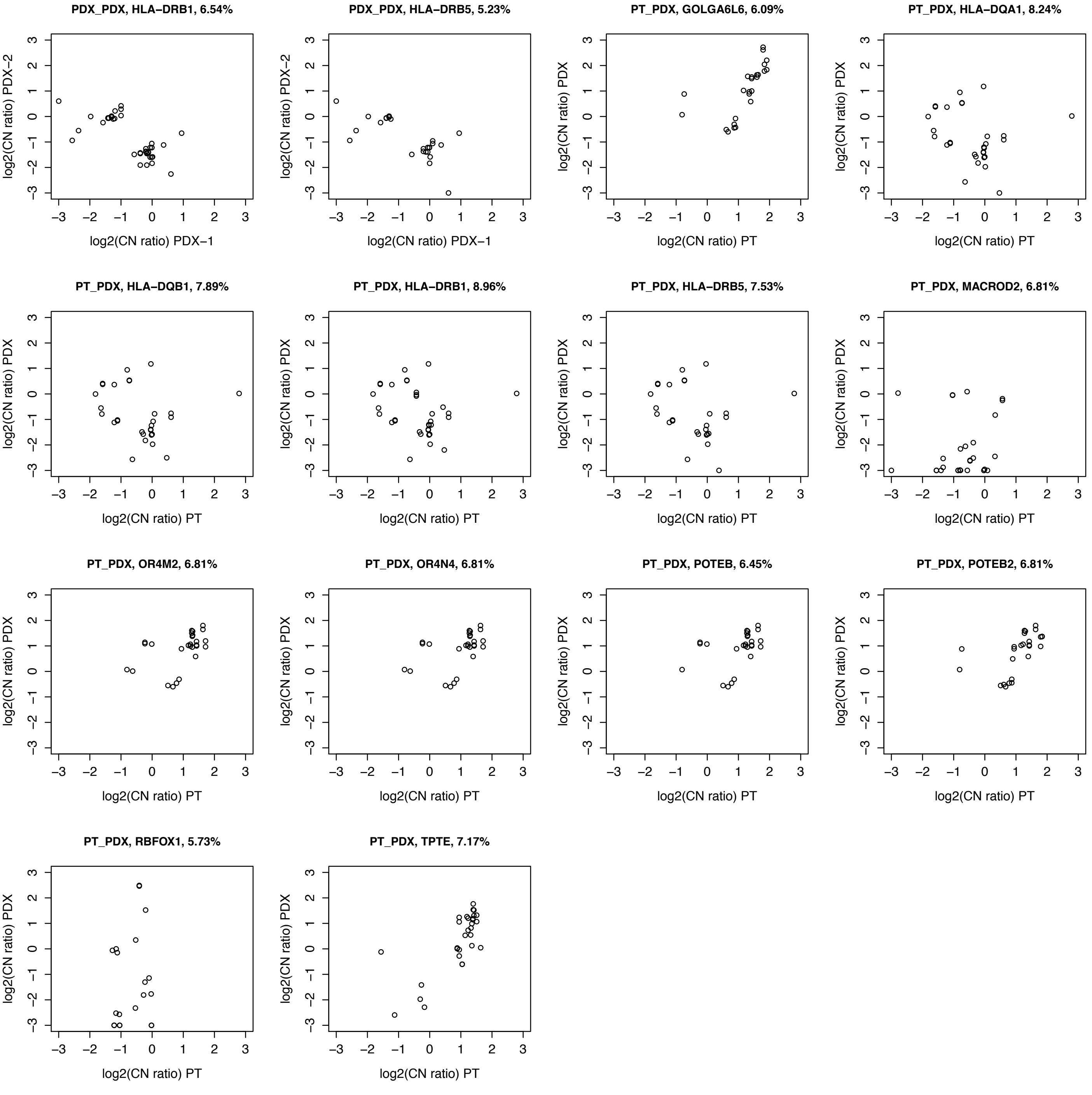

Supplementary Fig. 68: $\log_2$(CN ratio) values between each pair of samples of recurrent genes (see Supplementary Table 4). PDX-1: earlier passage, PDX-2: same passage but different lineage or later passage.

a

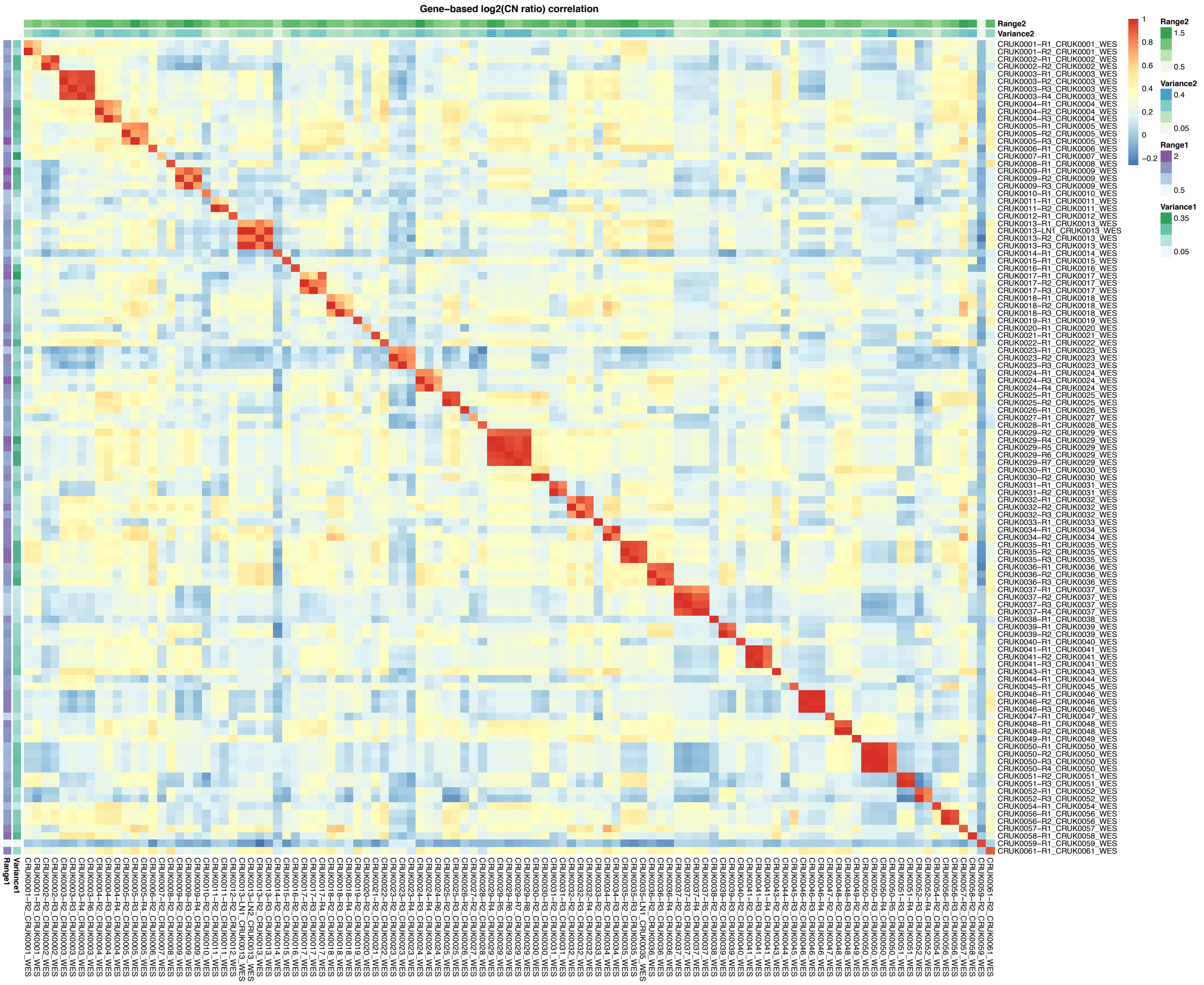

Supplementary Fig. 69: (Continue next page)

b

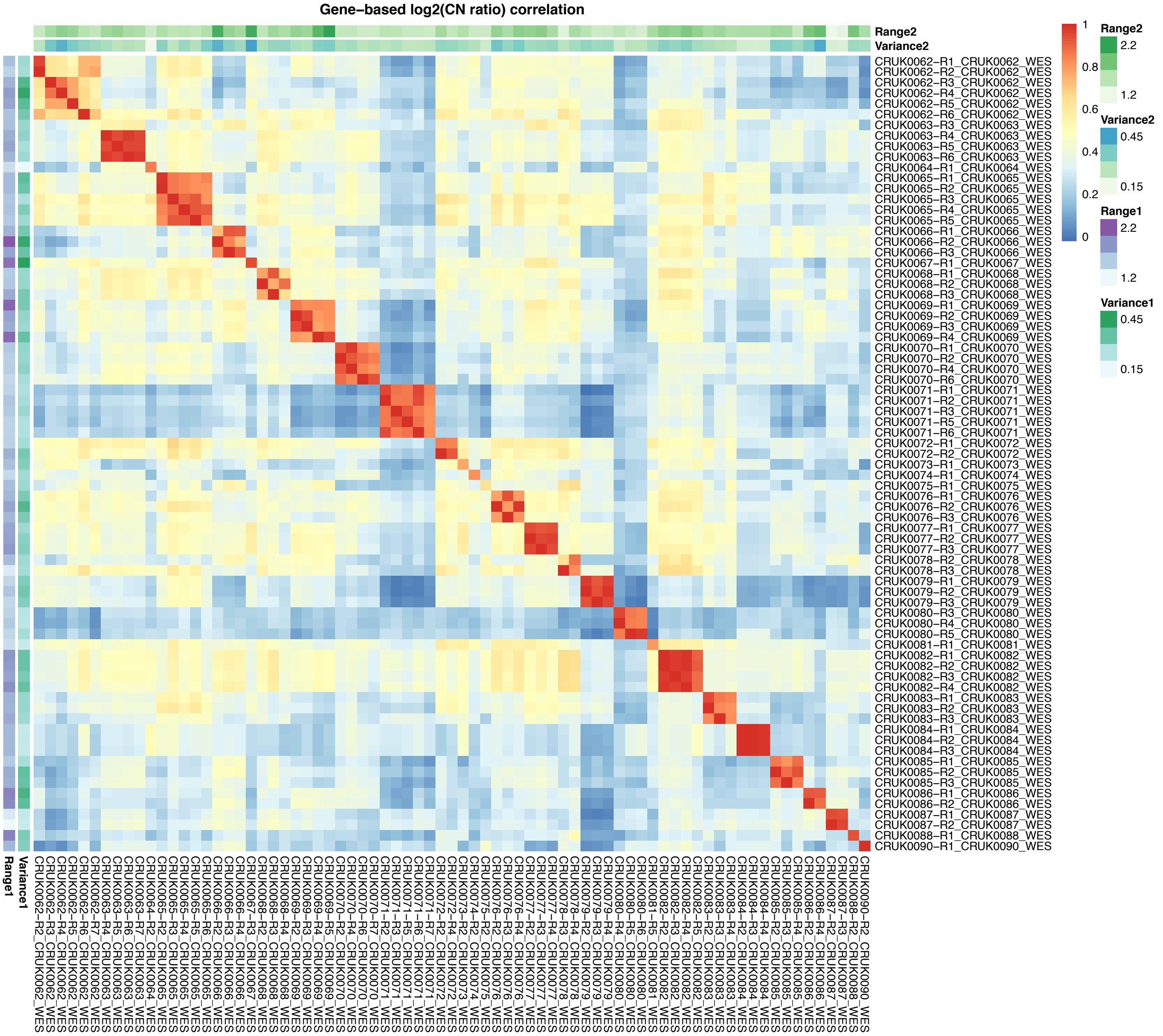

Supplementary Fig. 69: (Continue next page)

c

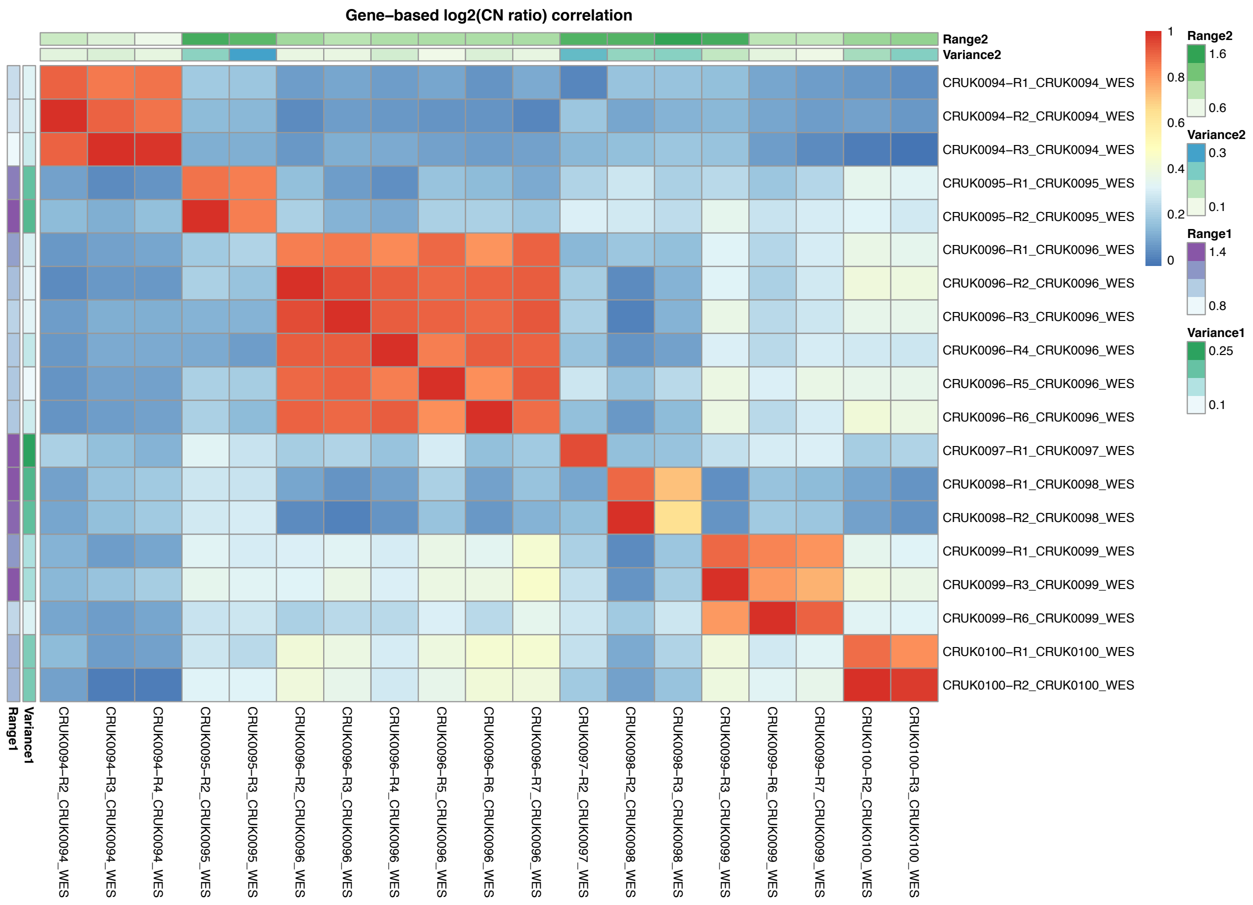

Supplementary Fig. 69: Correlation heatmap of gene-based copy number (log2(CN ratio), median centered) of multi-region samples of the same tumor from TRACERx (**a**) lung adenocarcinoma (LUAD), (**b**) lung squamous cell carcinoma (LUSC) and (**c**) other lung cancer subtypes.

a

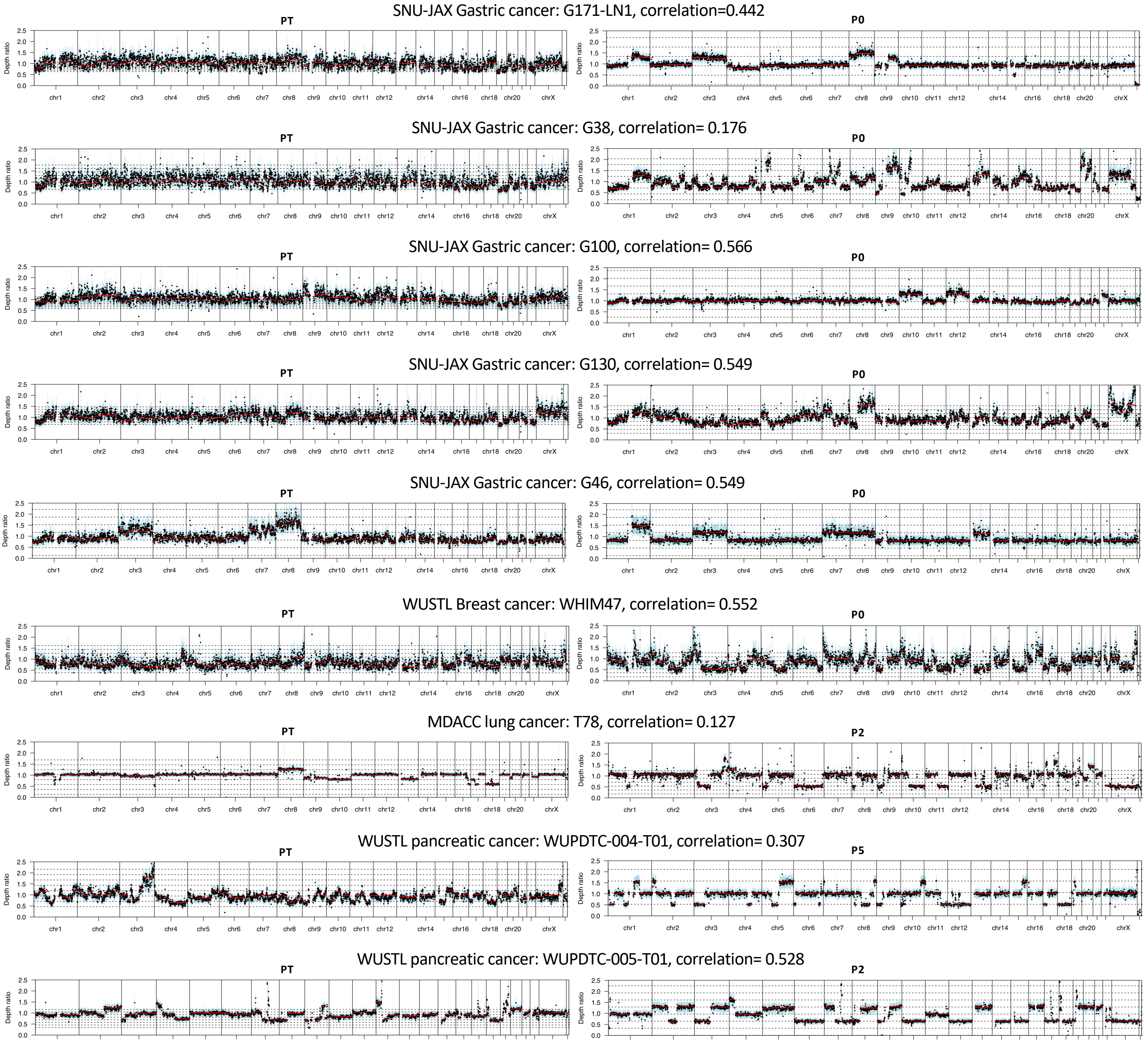

Supplementary Fig. 70: (Continue next page)

b

EuroPDX BRCA: BC291A
Correlation=
PT vs P1: 0.907
PT vs P7: 0.498
P1 vs P7: 0.566

EuroPDX BRCA: BC989
Correlation=
PT vs P1: 0.592
PT vs P5: 0.625
P1 vs P5: 0.769

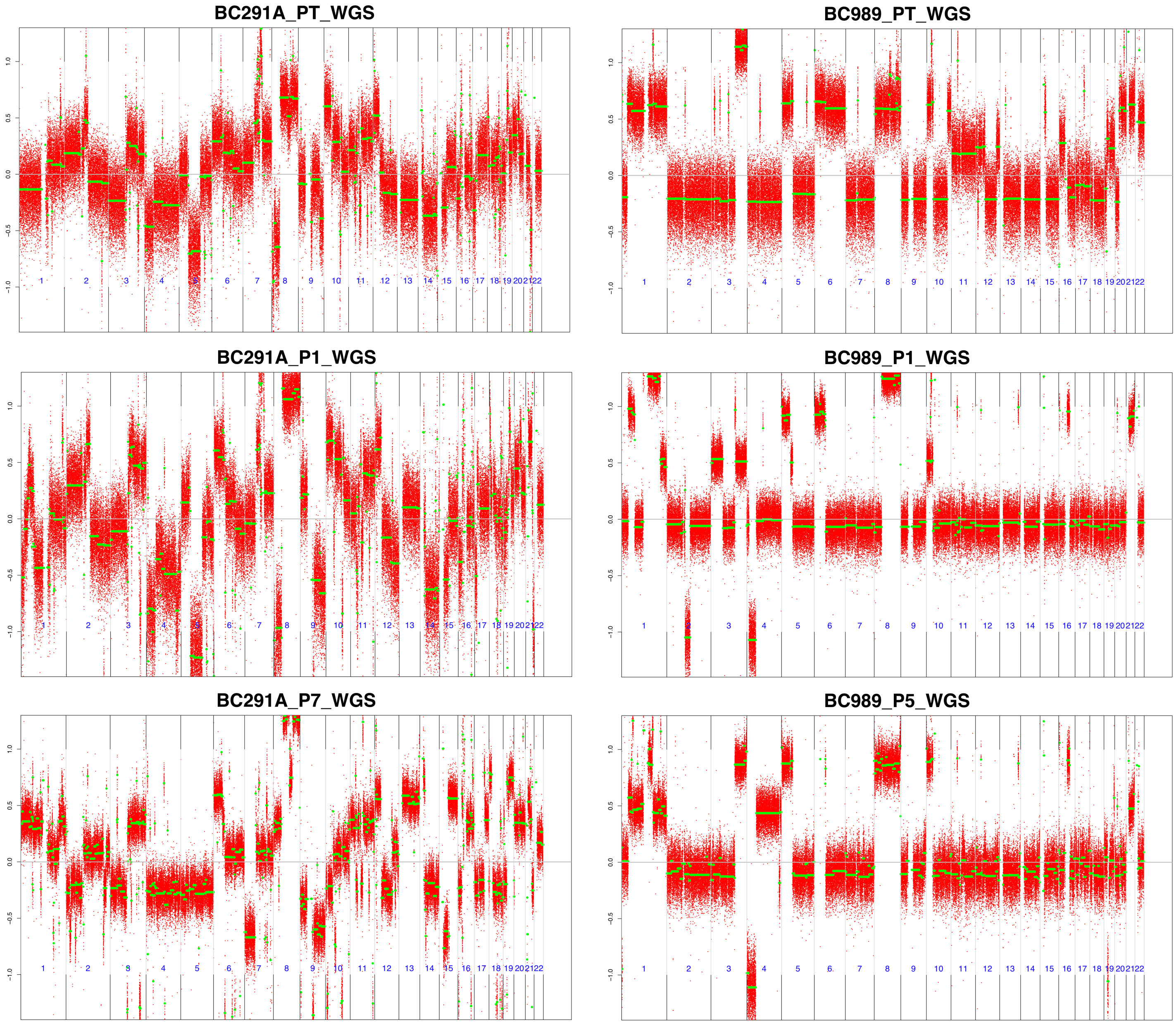

Supplementary Fig. 70: Window-based and segmented copy number estimated as (**a**) depth ratio by Sequenza for WES, and (**b**) $\log_2$ (ratio) by ASCAT for WGS, for same lineage samples identified to be aberrant (5-95% inter-percentile range of log2(CN ratio) values > 0.5 for both samples) and low correlation (Pearson correlation coefficient < 0.6) between the sample pairs

a

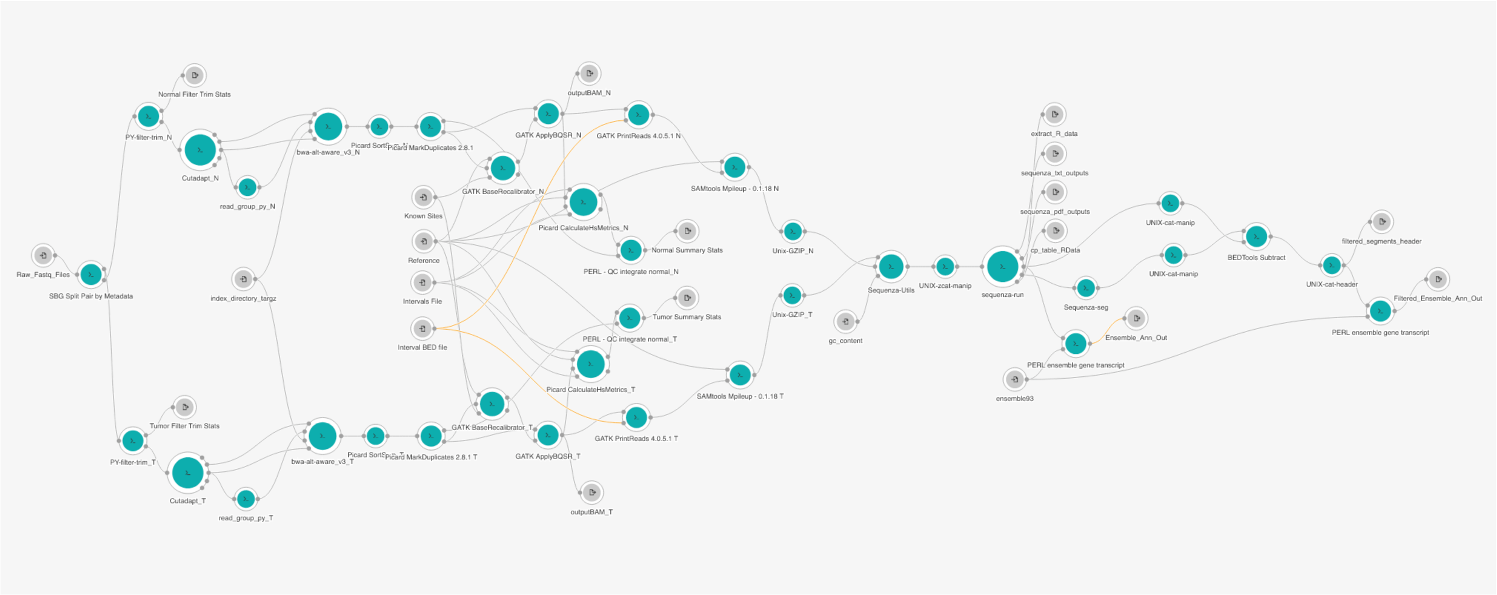

b

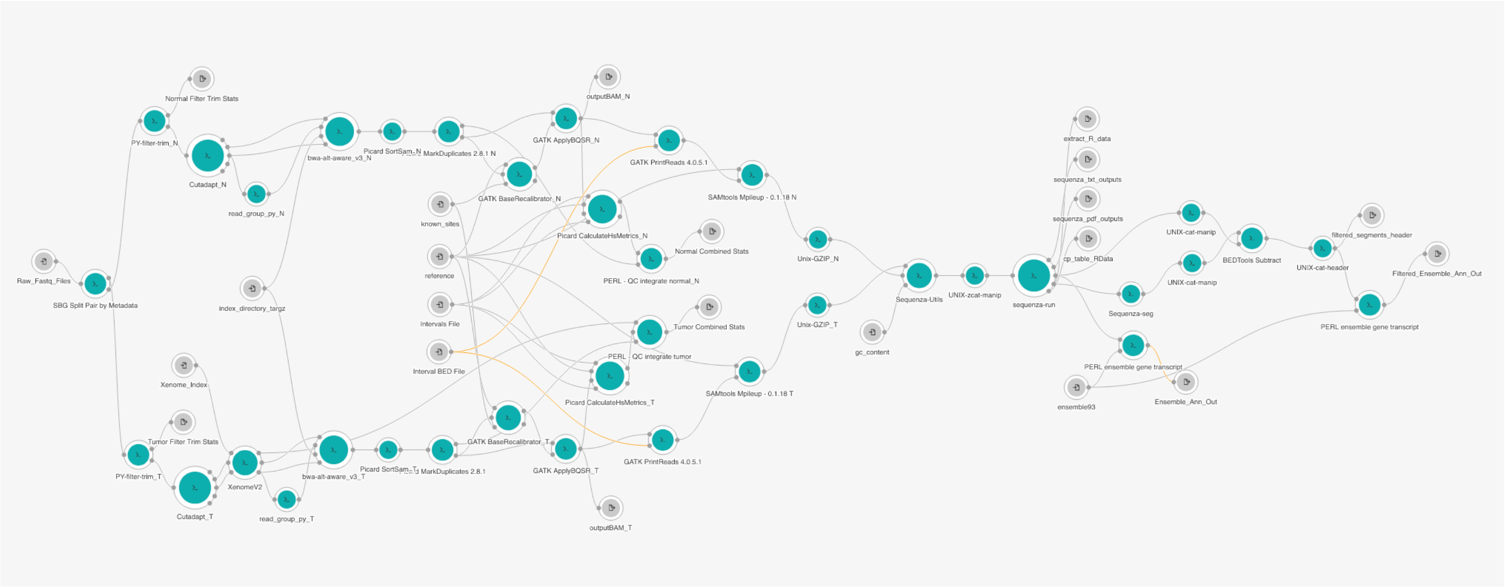

Supplementary Fig. 71: Workflow for copy number estimation by Sequenza from whole-exome sequencing data with paired-normal for (a) patient tumor (without Xenome) and (**b**) PDX tumor (with Xenome for mouse reads removal).

SUPPLEMENTARY TABLES

Data source	Number of unique models	SNP array (single tumor)			Whole-exome sequencing (tumor-normal)			Whole-genome sequencing (single tumor)			RNA-Seq or Microarray (tumor-normal)			Number of unique samples	
		Number of models	Number of patient tumor samples	Number of PDX samples	Number of models	Number of patient tumor samples	Number of PDX samples	Number of models	Number of patient tumor samples	Number of PDX samples	Number of models	Number of patient tumor samples	Number of PDX samples	PT	PDX
JAX PDX resource (various tumor types)	105	105	27	203	3	3	0							27	203
SNU-JAX gastric cancer	48				33	33	33				42	42	42	48	48
HCI breast cancer	18	13	12	16	10	9	9							16	25
BCM breast cancer	14	14	0	28										0	28
MDACC lung cancer	45				45	45	45							45	45
Wistar melanoma	10				10	8	9							8	9
NCI PDMR (various tumor types)	83				83	21	439							21	439
WUSTL pancreatic cancer	5				5	5	5							5	5
WUSTL breast cancer	13				13	13	13							13	13
SIBS hepatocellular carcinoma	28	28	2	33							28	3	34	3	34
EuroPDX colorectal cancer (liver metastasis)	97							97	95	192				95	192
EuroPDX breast cancer	43							43	43	86				43	86
Total	**509**	**160**	**41**	**280**	**202**	**137**	**553**	**140**	**138**	**278**	**70**	**45**	**76**	**324**	**1127**

Supplementary Table 1: Summary of datasets collected from various centers in the PDXNET consortium, EuroPDX consortium and published datasets.

Tumor type	Total models	Models with patient tumor samples	Models with multiple PDX samples	Number of PDX samples
Colorectal cancer	130	100	123	299
Breast cancer	96	74	69	167
Gastric cancer	48	48	0	48
Lung squamous cell carcinoma	34	19	17	69
Lung adenocarcinoma	33	23	11	59
Hepatocellular carcinoma	28	3	5	34
Skin melanoma and other skin cancers	28	13	17	79
Head and neck cancer	22	5	22	106
Sarcoma	19	7	15	73
Uninary Bladder cancer	16	6	15	61
Other lung cancers	11	7	5	16
Brain glioblastoma multiforme	10	9	2	13
Ovarian cancer and other female reproducitve organ cancers	10	1	10	31
Pancreatic cancer	10	6	5	27
Renal cell carcinoma and other kidney cancers	8	2	7	29
Other cancers	6	1	5	16
**Total**	**509**	**324**	**328**	**1127**

Supplementary Table 2: Summary of datasets by tumor type.

Comparison dataset		Data source	Number of PT samples	Number of PDX samples
SNP vs WES	Comparison of CNA profiles estiimated from SNP array and whole-exome sequencing	JAX PDX resource and HCI breast cancer	8	0
WES vs RNASEQ (NORM/TUM)	Comparison of CNA profiles estimated from whole-exome sequencing and RNA-sequencing, either normalized by median expression of normal samples of the same tumor type or median expression of the same set of tumor samples	SNU-JAX gastric cancer	27	27
SNP vs EXPARR (NORM/TUM)	Comparison of CNA profiles estimated from SNP array and gene expression array, either normalized by median expression of normal samples of the same tumor type or median expression of the same set of tumor samples	SIBS hepatocellular carcinoma	2	33
RNASEQ NORM vs TUM	Comparison of CNA profiles estimated from RNA-sequencing with different normalizations, by median expression of normal samples of the same tumor type or median expression of the same set of tumor samples	SNU-JAX gastric cancer	42	42
EXPARR NORM vs TUM	Comparison of CNA profiles estimated from gene expression array with different normalizations, by median expression of normal samples of the same tumor type or median expression of the same set of tumor samples	SIBS hepatocellular carcinoma	3	34

Supplementary Table 3: Validation dataset which comprises copy number alteration profiles estimated for matched samples assayed across multiple platforms

Genes with >5% recurrent frequency (|residual| > 1)	Gene location (GRCh38)	Recurrent frequency (%)	
		PT vs PDX	PDX vs PDX
GOLGA6L6	15q11.2	6.09	
HLA-DQA1	6p21.32	8.24	
HLA-DQB1	6p21.32	7.89	
HLA-DRB1	6p21.32	8.96	6.54
HLA-DRB5	6p21.32	7.53	5.23
MACROD2	20p12.1	6.81	
OR4M2	15q11.2	6.81	
OR4N4	15q11.2	6.81	
POTEB	15q11.2	6.45	
POTEB2	15q11.2	6.81	
RBFOX1	16p13.3	5.73	
TPTE	21p11.2	7.17	

Supplementary Table 4: Recurrent frequency (based on models) of genes with >5% recurrence with large copy number deviation (|residual| > 1) from linear regression model for PT-PDX (279 models) and PDX-PDX (306 models) comparisons.

Data source	Initial take rate
JAX PDX resource (various tumor types)	2 - 60%
SNU-JAX gastric cancer	NA
HCI breast cancer	28% [22]
BCM breast cancer	40 - 90% [41]
MDACC lung cancer	35% [25]
Wistar melanoma	100%
NCI PDMR (various tumor types)	(Evrard et al., In Preparation)
WUSTL pancreatic cancer	28%
WUSTL breast cancer	6 - 17%
SIBS hepatocellular carcinoma	NA
EuroPDX colorectal cancer (liver metastasis)	85% [30]
EuroPDX breast cancer	5-11%

Supplementary Table 10: Xenograft initial take rates of tumors from the various contributing centers.

SUPPLEMENTARY REFERENCES

1. Menghi, F. *et al.* The tandem duplicator phenotype as a distinct genomic configuration in cancer. *Proceedings of the National Academy of Sciences* **113**, E2373-E2382 (2016).
2. Stephens, P.J. *et al.* Massive Genomic Rearrangement Acquired in a Single Catastrophic Event during Cancer Development. *Cell* **144**, 27-40 (2011).
3. Notta, F. *et al.* A renewed model of pancreatic cancer evolution based on genomic rearrangement patterns. *Nature* **538**, 378-382 (2016).
4. Cortes-Ciriano, I. *et al.* Comprehensive analysis of chromothripsis in 2,658 human cancers using whole-genome sequencing. *Nat Genet* **52**, 331-341 (2020).
5. Schumacher, S.E. *et al.* Somatic copy number alterations in gastric adenocarcinomas among Asian and Western patients. *PLOS ONE* **12**, e0176045 (2017).
6. The Cancer Genome Atlas Research, N. *et al.* Comprehensive molecular characterization of gastric adenocarcinoma. *Nature* **513**, 202-209 (2014).
7. Ally, A. *et al.* Comprehensive and Integrative Genomic Characterization of Hepatocellular Carcinoma. *Cell* **169**, 1327-1341.e23 (2017).
8. Hastings, P.J., Lupski, J.R., Rosenberg, S.M. & Ira, G. Mechanisms of change in gene copy number. *Nature Reviews Genetics* **10**, 551-564 (2009).
9. Riaz, N. *et al.* Pan-cancer analysis of bi-allelic alterations in homologous recombination DNA repair genes. *Nature Communications* **8**, 857 (2017).
10. Broad Institute TCGA Genome Data Analysis Center: SNP6 Copy number analysis (GISTIC2). Broad Institute of MIT and Harvard. doi:10.7908/C1NP23RQ. (2016).
11. Broad Institute TCGA Genome Data Analysis Center: SNP6 Copy number analysis (GISTIC2). Broad Institute of MIT and Harvard. doi:10.7908/C1SJ1JZ8. (2016).
12. Zhang, X. *et al.* Evaluation of copy number variation detection for a SNP array platform. *BMC Bioinformatics* **15**, 50 (2014).
13. LaFramboise, T. Single nucleotide polymorphism arrays: a decade of biological, computational and technological advances. *Nucleic acids research* **37**, 4181-4193 (2009).
14. Van Loo, P. *et al.* Allele-specific copy number analysis of tumors. *Proceedings of the National Academy of Sciences* **107**, 16910-16915 (2010).
15. Miller, C.A. *et al.* SciClone: Inferring Clonal Architecture and Tracking the Spatial and Temporal Patterns of Tumor Evolution. *PLOS Computational Biology* **10**, e1003665 (2014).
16. Eirew, P. *et al.* Dynamics of genomic clones in breast cancer patient xenografts at single-cell resolution. *Nature* **518**, 422-426 (2014).
17. Ding, L. *et al.* Genome remodelling in a basal-like breast cancer metastasis and xenograft. *Nature* **464**, 999-1005 (2010).
18. Krupke, D.M. *et al.* The Mouse Tumor Biology Database: A Comprehensive Resource for Mouse Models of Human Cancer. *Cancer Res* **77**, e67-e70 (2017).
19. Conte, N. *et al.* PDX Finder: A portal for patient-derived tumor xenograft model discovery. *Nucleic Acids Res* **47**, D1073-D1079 (2019).
20. Woo, X.Y. *et al.* Genomic data analysis workflows for tumors from patient-derived xenografts (PDXs): challenges and guidelines. *BMC Medical Genomics* **12**, 92 (2019).
21. DeRose, Y.S. *et al.* Patient-derived models of human breast cancer: protocols for in vitro and in vivo applications in tumor biology and translational medicine. *Curr Protoc Pharmacol* **Chapter 14**, Unit14 23 (2013).
22. DeRose, Y.S. *et al.* Tumor grafts derived from women with breast cancer authentically reflect tumor pathology, growth, metastasis and disease outcomes. *Nature Medicine* **17**, 1514-1520 (2011).
23. Zhang, X.M. *et al.* A renewable tissue resource of phenotypically stable, biologically and ethnically diverse, patient-derived human breast cancer xenograft models. *Cancer Research* **73**, 4885-4897 (2013).

24. Li, S. *et al.* Endocrine-therapy-resistant ESR1 variants revealed by genomic characterization of breast-cancer-derived xenografts. *Cell Rep* **4**, 1116-1130 (2013).
25. Chen, Y. *et al.* Tumor characteristics associated with engraftment of patient-derived non-small cell lung cancer xenografts in immunocompromised mice. *Cancer* **125**, 3738-3748 (2019).
26. He, S. *et al.* PDXliver: a database of liver cancer patient derived xenograft mouse models. *BMC Cancer* **18**, 550 (2018).
27. Roth, R.B. *et al.* Gene expression analyses reveal molecular relationships among 20 regions of the human CNS. *Neurogenetics* **7**, 67-80 (2006).
28. Huang, Y. *et al.* Identification of a two-layer regulatory network of proliferation-related microRNAs in hepatoma cells. *Nucleic Acids Research* **40**, 10478-10493 (2012).
29. Malouf, G.G. *et al.* Transcriptional profiling of pure fibrolamellar hepatocellular carcinoma reveals an endocrine signature. *Hepatology* **59**, 2228-2237 (2014).
30. Bertotti, A. *et al.* A Molecularly Annotated Platform of Patient-Derived Xenografts ("Xenopatients") Identifies HER2 as an Effective Therapeutic Target in Cetuximab-Resistant Colorectal Cancer. *Cancer Discovery* **1**, 508-523 (2011).
31. Galimi, F. *et al.* Genetic and expression analysis of MET, MACC1, and HGF in metastatic colorectal cancer: response to met inhibition in patient xenografts and pathologic correlations. *Clin Cancer Res* **17**, 3146-3156 (2011).
32. Laird, P.W. *et al.* Simplified mammalian DNA isolation procedure. *Nucleic Acids Res* **19**, 4293 (1991).
33. Martin, M. Cutadapt removes adapter sequences from high-throughput sequencing reads. *2011* **17**, 10-12 (2011).
34. Li, H. & Durbin, R. Fast and accurate short read alignment with Burrows-Wheeler transform. *Bioinformatics* **25**, 1754-1760 (2009).
35. Conway, T. *et al.* Xenome--a tool for classifying reads from xenograft samples. *Bioinformatics* **28**, i172-i178 (2012).
36. McKenna, A. *et al.* The Genome Analysis Toolkit: a MapReduce framework for analyzing next-generation DNA sequencing data. *Genome Res* **20**, 1297-303 (2010).
37. DePristo, M.A. *et al.* A framework for variation discovery and genotyping using next-generation DNA sequencing data. *Nat Genet* **43**, 491-498 (2011).
38. Li, H. *et al.* The Sequence Alignment/Map format and SAMtools. *Bioinformatics* **25**, 2078-2079 (2009).
39. Kluin, R.J.C. *et al.* XenofilteR: computational deconvolution of mouse and human reads in tumor xenograft sequence data. *Bmc Bioinformatics* **19**(2018).
40. Li, B. & Dewey, C.N. RSEM: accurate transcript quantification from RNA-Seq data with or without a reference genome. *BMC Bioinformatics* **12**, 323 (2011).
41. Dobrolecki, L.E. *et al.* Patient-derived xenograft (PDX) models in basic and translational breast cancer research. *Cancer and Metastasis Reviews* **35**, 547-573 (2016).
