# Supplementary material for: Conservation of copy number profiles during engraftment and passaging of patient-derived cancer xenografts
Source: Nat Genet. 2021 Jan 7;53(1):86–99. doi: 10.1038/s41588-020-00750-6 (PMC7808565; doi:10.1038/s41588-020-00750-6)
Supplement: Supplementary file 2 — Reporting Summary [file 41588_2020_750_MOESM2_ESM.pdf]

nature research

Corresponding author(s): Enzo Medico, Jeffrey Chuang

Last updated by author(s): Oct 26, 2020

Reporting Summary

Nature Research wishes to improve the reproducibility of the work that we publish. This form provides structure for consistency and transparency in reporting. For further information on Nature Research policies, see Authors & Referees and the Editorial Policy Checklist.

Statistics

For all statistical analyses, confirm that the following items are present in the figure legend, table legend, main text, or Methods section.

n/a	Confirmed	
☐	☒	The exact sample size (*n*) for each experimental group/condition, given as a discrete number and unit of measurement
☒	☐	A statement on whether measurements were taken from distinct samples or whether the same sample was measured repeatedly
☐	☒	The statistical test(s) used AND whether they are one- or two-sided<br>*Only common tests should be described solely by name; describe more complex techniques in the Methods section.*
☐	☒	A description of all covariates tested
☐	☒	A description of any assumptions or corrections, such as tests of normality and adjustment for multiple comparisons
☐	☒	A full description of the statistical parameters including central tendency (e.g. means) or other basic estimates (e.g. regression coefficient) AND variation (e.g. standard deviation) or associated estimates of uncertainty (e.g. confidence intervals)
☐	☒	For null hypothesis testing, the test statistic (e.g. F, t, r) with confidence intervals, effect sizes, degrees of freedom and P value noted<br>*Give P values as exact values whenever suitable.*
☒	☐	For Bayesian analysis, information on the choice of priors and Markov chain Monte Carlo settings
☒	☐	For hierarchical and complex designs, identification of the appropriate level for tests and full reporting of outcomes
☐	☒	Estimates of effect sizes (e.g. Cohen's d, Pearson's r), indicating how they were calculated

Our web collection on statistics for biologists contains articles on many of the points above.

Software and code

Policy information about availability of computer code

Data collection	No specialized software was used for data collection.
Data analysis	We have used well-established computational sequence analysis and statistical analysis techniques, so no code is provided. Full descriptions of all analysis techniques are provided in the Methods. The implementation of the copy number estimation workflow from whole-exome sequencing data is deployed in the cancer genomics cloud at SevenBridges (https://cgc.sbgenomics.com/public/apps#pdxnet/pdx-wf-commit2/wes-cnv-tumor-normal-workflow/, https://cgc.sbgenomics.com/public/apps#pdxnet/pdx-wf-commit2/pdx-wes-cnv-xenome-tumor-normal-workflow/).<br>Publicly available algorithm/software used in the analyses include PennCNV-Affy, Affymetrix Power Tools v1.15.0, Illumina GenomeStudio, ASCAT (v2.0.7, v2.4.3, v2.5.1), cut-adapt v1.15, BWA (v0.7.12, v0.7.15), Xenome v1.0.0, Picard (v1.43 , v2.8.1), GATK 4.0.5.1, SnpEff v4.3, SAMTools v0.1.18, XenofilteR, Sequenza v2.1.2, QDNAseq v1.20, RSEM v1.3.1, CGH-Explorer, IGV v2.4.13, GenVisR v1.16.1, Bedtools v2.26.0, GISTIC 2 v6.15.28 and GSEA v3.0.

For manuscripts utilizing custom algorithms or software that are central to the research but not yet described in published literature, software must be made available to editors/reviewers. We strongly encourage code deposition in a community repository (e.g. GitHub). See the Nature Research guidelines for submitting code & software for further information.

Data

Policy information about availability of data

All manuscripts must include a data availability statement. This statement should provide the following information, where applicable:

- Accession codes, unique identifiers, or web links for publicly available datasets
- A list of figures that have associated raw data
- A description of any restrictions on data availability

Copy number calls from all datasets are available in Supplementary Data 1, and these are used for all figures. Raw sequence data for these calls are a combination of previously described sources (notably the publicly available NCI Patient Derived Models Repository, pdmr.cancer.gov) and newly sequenced data. New sequence

data from the PDXNet are being shared as part of the NCI Cancer Moonshot initiative through the Cancer Data Service. For further details, contact the authors. The SNP array data generated by The Jackson Laboratory can be requested via the Mouse Models of Human Cancer Database (tumor.informatics.jax.org). The whole genome sequencing data generated by EurOPDX can be made available by directly contacting the EurOPDX consortium (dataportal.europdx.eu). Other publicly available data used in the analyses include GSE90653, GSE3526, GSE33006 and E-MTAB-1503-3, MSigDB v6.2 and TRACERx NSCLC data (DOI: 10.1056/NEJMoa1616288).

Field-specific reporting

Please select the one below that is the best fit for your research. If you are not sure, read the appropriate sections before making your selection.

☒ Life sciences ☐ Behavioural & social sciences ☐ Ecological, evolutionary & environmental sciences

For a reference copy of the document with all sections, see nature.com/documents/nr-reporting-summary-flat.pdf

Life sciences study design

All studies must disclose on these points even when the disclosure is negative.

Sample size	This is an extensive meta-analysis of evolutionary behaviors using >1500 samples to consider a variety of hypotheses. Descriptions of sample size, data exclusions, and replicability are provided throughout the manuscript.
Data exclusions	See above
Replication	See above
Randomization	This is not a case/control study and randomization is not suited to the project design.
Blinding	This is not a case/control study and blinding is not suited to the project design.

Reporting for specific materials, systems and methods

We require information from authors about some types of materials, experimental systems and methods used in many studies. Here, indicate whether each material, system or method listed is relevant to your study. If you are not sure if a list item applies to your research, read the appropriate section before selecting a response.

Materials & experimental systems

n/a	Involved in the study	
☒	☐	Antibodies
☒	☐	Eukaryotic cell lines
☒	☐	Palaeontology
☐	☒	Animals and other organisms
☒	☐	Human research participants
☒	☐	Clinical data

Methods

n/a	Involved in the study	
☒	☐	ChIP-seq
☒	☐	Flow cytometry
☒	☐	MRI-based neuroimaging

Animals and other organisms

Policy information about studies involving animals; ARRIVE guidelines recommended for reporting animal research

Laboratory animals	Details on mouse strains used for xenografting studies are provided in the Methods and the references therein.
Wild animals	No wild animals
Field-collected samples	No field collected samples.
Ethics oversight	This study is a meta-analysis of data collected across a large number of consortium sites and does not follow a single study protocol. All studies were conducted in compliance with ethics regulations, as detailed in the Methods and the references therein.

Note that full information on the approval of the study protocol must also be provided in the manuscript.
